# Divergent IL18-STAT1 Immune Responses Underlie Differential Susceptibility to *Aeromonas hydrophila* in *Geoclemys hamiltonii* and *Trachemys scripta*: A Comparative Transcriptomic Perspective

**DOI:** 10.3390/genes17040436

**Published:** 2026-04-09

**Authors:** Wenxiu Dai, Zerui Li, Yuqing Liu, Yingwen Zhou, Yanan Gan, Yinzi Ye, Yi Mu

**Affiliations:** 1 College of Wildlife and Protected Area, Northeast Forestry University, Harbin 150040, China; growth_star123@163.com (W.D.); 18324699299@163.com (Z.L.); 18904860066@163.com (Y.L.); zhouyingwen@nefu.edu.cn (Y.Z.); n2793262900@163.com (Y.G.); yexinyinzi@foxmail.com (Y.Y.); 2 Heilongjiang Key Laboratory of Complex Traits and Protein Machines in Organisms, Harbin 150040, China

**Keywords:** *Geoclemys hamiltonii*, endangered turtles, *Aeromonas hydrophila*, splenic transcriptome, *IL18*, *IFN-γ* pathway, comparative immunology, transcriptomics

## Abstract

**Background/Objectives**:**** The IUCN endangered spotted pond turtle (*Geoclemys hamiltonii*) demonstrates markedly reduced resistance to *Aeromonas hydrophila*-induced infections compared to the red-eared slider (*Trachemys scripta*). This study investigates the immunological basis for this disparity by analyzing infection outcomes and splenic transcriptomes of both species post-pathogen challenge. **Methods:** In a preliminary experiment, 32 turtles (16 *G. hamiltonii* and 16 *T. scripta*) were exposed to *A. hydrophila*. **Results:** *G. hamiltonii* developed skin ulcer syndrome at a significantly higher incidence (81.25%) than *T. scripta* (12.5%) (*p* < 0.05). Comparative transcriptomic analysis identified 19 differentially expressed immune-related genes, with qPCR validation across five tissues (heart, liver, spleen, intestine, blood) revealing pronounced interspecies differences in *IL18*, *STAT1*, *IFIH1*, and *CD28* expression. Notably, *IL18* and its downstream effector *STAT1* were robustly upregulated in *T. scripta* but were considerably lower in *G. hamiltonii*, correlating with delayed *IFN-γ* pathway activation and impaired epidermal barrier repair. Concurrently, *CD28* upregulation in *T. scripta* facilitated rapid T-cell-mediated pathogen clearance, whereas its delayed induction in *G. hamiltonii* hindered adaptive immunity. These findings implicate dysregulated innate (*IL18/STAT1*) and adaptive (*CD28*) immune pathways as key determinants of *G. hamiltonii*’s susceptibility to bacterial infection. **Conclusions:** Despite the critical conservation status of *G. hamiltonii*, the immunological basis underlying its heightened susceptibility to bacterial infections remains largely unexplored; this study addresses this gap by comparing the splenic transcriptomes of *G. hamiltonii* and *T. scripta* following *A. hydrophila* challenge, identifying the dysregulated *IL18*-*STAT1* Immune Axis and *CD28*-mediated adaptive immunity as key determinants, thereby providing actionable immune targets for conservation breeding and susceptibility screening in this endangered species.

## 1. Introduction

The spotted pond turtle (*Geoclemys hamiltonii*) is listed in CITES Appendix I and classified as Endangered (EN) on the IUCN Red List [1]. Its distribution is largely confined to the floodplains of the Brahmaputra River in Pakistan, India, Bangladesh, and Nepal [2], with rare occurrences beyond these regions. Populations of *G. hamiltonii* have been nearly extirpated outside protected areas in Assam [3], and surveys indicate a dramatic decline in Bangladesh [4] and Pakistan [5] over the past two decades, resulting in local extinction across most habitats. As a commercially valuable ornamental species, *G. hamiltonii* faces intensified anthropogenic pressure due to its economic significance [6].

*Aeromonas hydrophila* is a Gram-negative primary opportunistic pathogen that is widely distributed in various natural water bodies [7]. It can infect hosts through water or food contamination [8] and employs multiple virulence factors [9], including adhesins, secretory proteins, secretion systems, quorum-sensing systems, and metal uptake systems [10], causing diseases such as skin ulcer syndrome, scab disease, perforation disease, and white-eye disease in turtles, with severe infections leading to mortality [11].

Turtles utilize both innate and adaptive immunity to defend against bacterial pathogen infections [12]. Pathogen-associated molecular patterns (*PAMPs*) on the surface of pathogenic microorganisms, such as lipopolysaccharides in Gram-negative bacterial cell walls and peptidoglycan in Gram-positive bacterial cell walls, are recognized by pattern recognition receptors (e.g., *TLRs*) on the immune cell surface [13], triggering downstream signaling cascades and activating innate immune responses. This process leads to the sequential expression of immune-related genes, including interferon genes α and β (*IFNA* and *IFNB*), tumor necrosis factor (*TNF*), interleukins (*IL*s), interferon regulatory factors (*IRF*), and interferon-stimulated genes (*ISGs*) [14]. Mechanistic target of rapamycin (*mTOR*) acts on the immune system by mediating T-cell activation [15] and can enhance innate immune responses. Additionally, antigens are presented to B- and T-cells via the major histocompatibility complex (*MHC*), activating adaptive immune pathways [16]. Current research on turtle immune signaling pathways primarily focuses on the *NF-κB*, *MAPK*, and *JAK-STAT* pathways involved in innate immunity [17]. For example, studies have shown that *TLR4* in Chinese soft-shelled turtles plays a crucial role in recognizing Gram-negative bacteria and activating inflammatory responses through the *NF-κB* pathway [18]. Overall, research on turtle immunity has predominantly focused on the sequences and functions of immune-related genes, with limited studies conducted on the immune mechanisms of different turtle species.

In our preliminary trials, we observed striking interspecific differences in susceptibility to *A. hydrophila* infection between turtle species challenged with the same bacterial dose: *G. hamiltonii* exhibited a skin ulcer syndrome incidence of 81.25%, whereas *Trachemys scripta* had an incidence of only 12.5% (*p* < 0.05). To elucidate the immunological mechanisms underlying this disparity and investigate the basis of *G. hamiltonii*’s heightened susceptibility, we performed comparative splenic transcriptome profiling of the two species following bacterial challenge to identify candidate immune genes. Subsequently, we validated the spatiotemporal expression patterns of these candidate genes across five tissues (heart, liver, spleen, intestine, and blood) over the infection time course using quantitative real-time PCR. Through this integrated approach—combining transcriptome-wide screening with tissue- and time-resolved expression validation—we ultimately identified dysregulation of the *IL18*-*STAT1* innate immune axis and *CD28*-mediated adaptive immunity as the most prominent molecular differences between the two species. We propose that these divergent immune responses are the key determinants of *G. hamiltonii*’s heightened susceptibility to *A. hydrophila* infection.

## 2. Materials and Methods

### 2.1. Turtle Collection and Acclimation

Newly hatched *G*. *hamiltonii* (straight-line carapace length, SLV: 70 ± 5 mm) and *T*. *scripta* (SLV: 50 ± 5 mm) were obtained from private hatcheries in Maoming, China (21°22′–22°42′ N, 110°19′–111°41′ E). The actual size difference between hatchlings within the same species is less than 5 mm, ensuring intra-species consistency. Although hatchlings’ body size differs between the two species as an inherent species characteristic, all hatchlings were collected within 3 days of hatching, ensuring they are at a comparable developmental stage and immune maturity. For each species, 50 individuals were housed separately in glass aquaria (500 × 350 × 200 mm^3^) under controlled conditions: water temperature, 28 °C; purified water depth, 50 mm (pH 6.9); and two synthetic resin basking platforms (200 × 100 × 50 mm^3^). After a 3-day acclimation period, turtles were fed a commercial hatchling diet (Lifeline^®^, Hangzhou, China) and their water was completely changed daily. Water quality was monitored via the plate streaking method, confirming the absence of *A. hydrophila*. All turtles underwent 15-day health screenings to exclude *A. hydrophila* infection symptoms (e.g., dermal yellow/white ulcers). The animal procedures were approved by the Northeast Forestry University Animal Care Committee (Approval No.: 2024021).

### 2.2. Bacterial Treatment and Tissue Collection

Following acclimation and health confirmation, 48 turtles per species were randomly assigned to three groups (*n* = 16/group): *G. hamiltonii*: Infected (Groups TH and THi), PBS control (Group CKH); *T. scripta*: Infected (Groups TB and TBi), PBS control (Group CKB). *A. hydrophila* (strain CGMCC 1801, China General Microbiological Culture Collection Center (CGMCC), Institute of Microbiology, Chinese Academy of Sciences, Beijing, China) was cultured in Luria–Bertani medium (Sigma-Aldrich, Saint Louis, MO, USA) at 28 °C for 24 h. Bacterial cells were harvested by centrifugation (3000× *g*, 12 min, 4 °C), washed thrice in sterile PBS, and resuspended to 1.9 × 108 colony-forming units (CFU) mL^−1^. Prior to inoculation, turtles received local anesthesia (1% lidocaine, Merck, Darmstadt, Germany), followed by a standardized dermal incision (2 mm length × 1 mm depth) on the left forelimb. Wounds were cleansed with PBS and inoculated with 1 µL bacterial suspension (infected groups) or PBS (controls). These parameters were selected based on previous studies [19] of bacterial infection models in turtles and the body size of the individuals, aiming to ensure localized bacterial exposure and consistent infection while minimizing tissue damage and inoculum leakage, thereby enhancing experimental reproducibility. The progression of the infection in Groups THi/TBi and CKH/CKB were monitored for 10 days post-injection (dpi), with ulcer incidence defined as: dermal yellow/white lesions with a necrotic odor and *A. hydrophila*-positive culture. For transcriptomic and RT-PCR analyses, three turtles per time point (0/3/5/10 dpi) from Groups TH/TB and CKH/CKB (day 0) were euthanized (MS-222, 200 mg/L). Heart, liver, spleen, intestine, and blood samples were flash-frozen in liquid nitrogen and stored at −80 °C.

To ensure that all sampled individuals were actively infected, prior to euthanasia at each designated time point, the wound site of each turtle was swabbed and cultured for re-isolation of *A. hydrophila*. Only turtles confirmed to be pathogen-positive were considered for sampling. From this pool of confirmed infected turtles, three individuals were randomly selected for tissue collection at 0, 3, 5, and 10 dpi. It is important to note that infection status was not determined solely by the presence of visible clinical signs; thus, turtles from both species, regardless of symptom severity, were included as long as bacterial re-isolation was successful. This approach ensured that the immune responses captured in the transcriptomic and qPCR analyses reflected active infection rather than differential symptom manifestation.

Due to the limited availability of *G. hamiltonii*, a CITES Appendix I-listed species, independent replicate infection experiments were not performed in this study. To maximize the reliability of the infection model under these constraints, the number of individuals showing clinical signs was recorded at each sampling time point within the infection cohort. The incidence trends observed in the sampling cohort were consistent with those in the infection group. Subsequent transcriptomic analyses (including three independent biological replicates) and qPCR data further supported the observed species-specific susceptibility patterns. Nevertheless, the absence of independent experimental replicates for the THi group *n* = 16 represents a limitation of this study.

### 2.3. Preparation for Spleen Transcriptome Sequencing

To precisely compare the core immune response differences between *G*. *hamiltonii* and *T*. *scripta* following pathogen challenge. Total RNA isolation was performed post-collection on 50 mg tissue specimens (both treated and control groups) employing TRIzol^®^ Reagent (Invitrogen, Waltham, MA, USA) following standard protocols. Total RNA was extracted using TRIzol reagent (Invitrogen). Briefly, samples were homogenized in 1 mL of pre-chilled TRIzol and incubated at room temperature for 5 min to dissociate nucleoprotein complexes. After adding 200 µL of chloroform, the mixture was vigorously vortexed for 20 s, incubated at room temperature for 3 min, and centrifuged at 12,000× *g* for 15 min at 4 °C. The upper aqueous phase was transferred to a new RNase-free tube, and RNA was precipitated with 500 µL of isopropanol. After centrifugation at 12,000× *g* for 10 min at 4 °C, the pellet was washed with 75% ethanol, air-dried briefly, and dissolved in RNase-free water. RNA quality verification was conducted using an Agilent 2100 Bioanalyzer (Agilent Technologies, Santa Clara, CA, USA), with splenic samples demonstrating an RIN (RNA integrity number) ≥7 qualifying for downstream processing. Library preparation was carried out using a TruseqTM RNA sample prep kit (Illumina, San Diego, CA, USA) according to established protocols. High-throughput sequencing was performed on Illumina platforms (including HiSeq™2000 systems), generating 150/125 bp paired-end sequencing fragments. Initial data filtration was implemented through proprietary bioinformatics pipelines. For each species and time point (0, 3, 5, 10 dpi), three independent biological replicates were included, each derived from a distinct individual. Technical replicates were not performed, as the sequencing depth (approximately 48 million clean reads per sample) was considered sufficient to ensure reliable quantification of gene expression levels.

### 2.4. Transcriptome Profiling and DEG Identification

High-throughput sequencing was performed on an Illumina NovaSeq 6000 system following the manufacturer’s standard protocols. Qualified libraries, with a main fragment peak ranging from 300 to 500 bp as assessed by the Agilent 2100 Bioanalyzer, were quantified using a Qubit Fluorometer v4. Libraries meeting quality standards were denatured and loaded onto the NovaSeq 6000 S4 flow cell, where clonal clusters were generated via onboard bridge amplification. Sequencing was conducted in paired-end 150 bp (PE150) mode using NovaSeq 6000 v1.5 sequencing reagents, based on Illumina sequencing-by-synthesis (SBS) technology, producing an average of 49.35 million raw reads per biological replicate. Raw image data were converted to FASTQ format via real-time base calling by the instrument’s built-in software. Subsequent demultiplexing and quality filtering were performed to remove low-quality reads, adapter sequences, and PCR duplicates. Initial quality control procedures were carried out using fastp v0.24 [20] for automated filtering of substandard sequences (Phred score < 20, length < 50 bp), resulting in the retention of 48.01 million high-confidence clean reads per sample. To enable cross-species differential expression analysis with mathematically valid normalization, clean reads from both species were ultimately aligned to the reference genome of *T. scripta* (GCF_013100865.1). For *G. hamiltonii*, a de novo transcriptome assembly was first generated (see Section 2.5) to capture species-specific transcripts; however, for comparative purposes, reads were also mapped to the *T. scripta* genome. Orthologous gene relationships between the two species were established using reciprocal best BLAST hits (RBH) with an E-value threshold of ≤1 × 10^−10^ and sequence coverage > 70%. Genome alignment was performed using HISAT2 v2.1.0 [21] with default parameters, and transcript abundance was quantified using HTSeq-count [22] to generate raw read counts. Multivariate analysis was conducted using R (v3.2.0) and included principal component analysis (PCA) for sample clustering validation. Differential expression screening was performed using DESeq2 v1.51.6 [23] with thresholds of |log2FC| ≥ 1 and FDR-adjusted *p* < 0.05. Hierarchical clustering and radar chart visualization (ggradar package) were employed to characterize expression patterns of the top 30 DEGs. Functional annotation incorporated four complementary databases (Gene Ontology (GO) [24], KEGG [25], Reactome, and WikiPathways), with enrichment significance evaluated through hypergeometric testing (FDR < 0.05). Data visualization workflows were implemented in R to generate column charts, chord diagrams, and bubble plots for pathway representation. Complementary Gene Set Enrichment Analysis (GSEA) was conducted to rank gene lists by differential-expression magnitude and detect predefined gene set enrichment at distribution extremes.

### 2.5. Transcript Assembly and Functional Annotation

Given the absence of a high-quality reference genome for *G*. *hamiltonii*, and to comprehensively capture species-specific novel transcripts that might be absent from the *T. scripta* annotation, a de novo transcriptome assembly was constructed. This approach mitigates potential mapping bias inherent in cross-species alignments and allows for the unbiased discovery of novel genetic features. The high genomic similarity (~96%) between *G. hamiltonii* and *T. scripta* [26], which supports the use of the latter’s genome for initial mapping, also ensured the feasibility of a robust de novo assembly. De novo transcriptome reconstruction was achieved through paired-end read splicing using Trinity v2.15.2 [27], followed by sequence consolidation using TGICL v2.1 [28] to generate non-redundant unigenes (minimum 90% identity, 80% coverage). To ensure the uniqueness of each gene locus, the following redundancy reduction pipeline was applied: raw reads in FASTQ format were first processed with Trimmomatic to remove adapters, poly-N-containing reads, and low-quality reads, yielding clean reads. These clean reads were then assembled into contigs using Trinity with default parameters. To reduce redundancy, the assembled transcripts were clustered based on sequence similarity and length using CD-HIT-EST (with a threshold of 95% identity). For each cluster, the longest transcript was selected as the representative unigene for subsequent analyses. This approach ensures that each unigene represents a unique gene locus, thereby minimizing redundancy. Notably, in de novo assembly using Trinity, alternative splicing variants are often represented as multiple transcripts derived from the same gene locus. However, in our pipeline, we selected the longest transcript from each cluster as the representative unigene to simplify downstream functional annotation and expression analysis. As a result, the current unigene set represents one representative transcript per gene locus, and alternative splicing variants are not retained as separate unigenes. Despite the fragmented nature of de novo assemblies, the high quality of our *G. hamiltonii* transcriptome (BUSCO completeness: 85.8% [73.6% single-copy and 12.2% duplicated], missing 2.0% of core genes; N50 = [2032]) provides confidence for subsequent functional annotation using BLASTx against the Nr database. Multidimensional functional annotation was conducted, which involved the alignment of BLASTX (E-value < 1 × 10^−10^) against three reference databases: NCBI Nr, Swiss-Prot, and KOG. KEGG pathway mapping was performed according to enzyme commission numbers. Annotation confidence was determined by identifying sequence similarity thresholds (>60% identity for Nr, >70% for Swiss-Prot), with priority given to top-hit proteins for functional assignment. To further ensure the reliability of the functional annotation, the assembled unigenes were re-evaluated against the Nr and Swiss-Prot databases, and the annotation results were confirmed and updated where necessary.

### 2.6. Identification of Transcriptional Variations and Functional Enrichment

Transcript abundance was quantified in *G. hamiltonii* and *T. scripta* specimens from the infection-challenged and control groups through alignment with transcriptomic references. Normalization of gene expression levels and detection of transcriptional differences were conducted using RPKM metrics (transcripts per million base pairs sequenced) as described in previous research [29]. Differential-expression patterns were identified through negative binomial distribution modeling implemented in the DESeq analytical package (Bioconductor release). A corrected significance threshold (Benjamini–Hochberg adjusted *p*-value < 0.05) served as the primary criterion for differential-expression screening. Transcripts that met the dual criteria of statistical significance (adjusted *p*-value < 0.05) and substantial expression variation (|log2 fold change| > 1) were classified as differentially expressed. Functionally significant gene sets were characterized through Gene Ontology (GO) term enrichment analysis complemented by pathway mapping against KEGG biochemical databases. Hypergeometric probability testing quantified the statistical significance of pathway associations. Metabolic pathway annotations for differentially expressed unigenes were performed using the KEGG Automatic Annotation Server (KAAS; http://www.genome.jp/kegg/kaas/ (accessed on 26 October 2024)). A subsequent interspecies comparative analysis of the expression levels of these immune-related DEGs was conducted using the R programming environment (v3.6.1) (Code S1). This analysis revealed genes with statistically significant expression differences (*p* < 0.05) between *T. scripta* and *G. hamiltonii* under infection conditions, ultimately identifying 19 key immune genes.

### 2.7. Quantitative Validation of Immune Gene Expression

Transcriptional validation studies were conducted using qRT-PCR to profile 19 immune-related targets across multiple tissues (cardiac, hepatic, splenic, intestinal, and hematopoietic systems) collected over 1–10 days post-infection. Candidate genes were selected based on transcriptomic evidence of differential regulation in immune pathways. Amplification reactions were conducted using SYBR Premix Ex Taq™ II chemistry (TaKaRa, DRR820A) in 50 µL volumes following the manufacturer’s specifications. To ensure accurate cross-species comparison of gene expression, all qPCR primers were meticulously designed as follows: First, the coding sequences (CDS) of target immune genes from *G. hamiltonii* and *T*. *scripta* were retrieved and aligned using Clustal Omega v1.2.4 to identify perfectly conserved regions (100% identity) (Figure S2). Primer pairs were then designed to span these conserved regions using Primer Premier 5.0 software, with amplicon lengths ranging between 80 and 150 bp. For each target gene, we designed at least three primer pairs during the preliminary experiment phase. Only primers with acceptable amplification efficiencies (90–105%) were used for subsequent detection. If all three primer pairs failed to meet the efficiency criterion, the design and validation step was repeated until qualified primers were obtained. The specificity of all primers was initially verified in silico by BLAST analysis against the NCBI database (see Table 1). All primers exhibited amplification efficiencies between 90 and 105%, which were consistent with the reference gene (β-actin), ensuring reliable quantification of target gene expression. The thermal cycling parameters consisted of initial denaturation (95 °C/5 min) followed by 40 amplification cycles (95 °C/5 s, 60 °C/30 s) with subsequent melt curve verification. β-actin was used as internal control for normalization, with all reactions performed in technical triplicate. To ensure the reliability of normalization under infectious conditions, we pre-validated the expression stability of β-actin: Using equal amounts of mRNA templates from both species and across different tissues (including the spleen), β-actin exhibited consistent Ct values of 22 ± 0.5 across three biological replicates, confirming that its expression remained stable following *A. hydrophila* challenge.

### 2.8. Computational Analysis of Experimental Data

Statistical evaluation was conducted using Statistica 6.0 (StatSoft, Hamburg, Germany), Fisher’s exact test was used for infection rate comparison and relative quantification of target gene expression was performed using the comparative Ct method (2^−ΔΔCT^) with *β-actin* normalization. Gene expression was normalized to the endogenous control *β-actin*. Inter-group variations in transcriptional profiles across temporal and treatment conditions were assessed through one-way ANOVA, and statistical significance (*p* < 0.05) was determined through a post hoc Tukey test following confirmation of variance homogeneity.

## 3. Results

### 3.1. Differences in the Infection Rates Between G. hamiltonii and T. scripta

**Figure 1:**
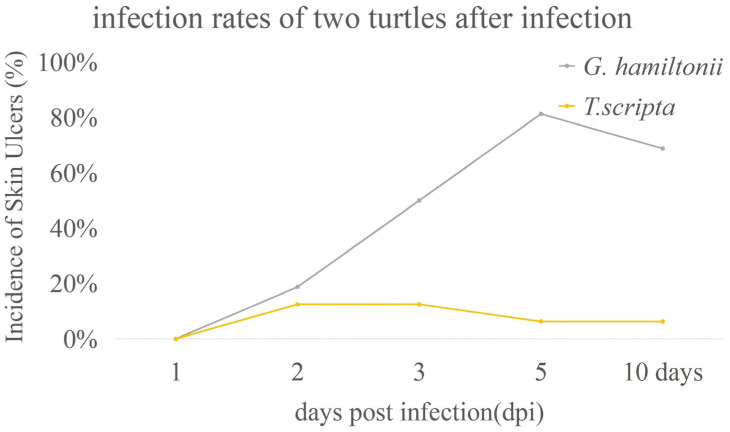
The infection rates (turtles show symptoms of skin ulcer syndrome) of *Geoclemys hamiltonii* and *Trachemys scripta* after pathogen challenge (*n* = 16, *p* = 0.02843). Hatchling *T. scripta* showed low infection rates (12.5%, *p* < 0.05) following challenge with *A. hydrophila*, whereas *G. hamiltonii* was highly susceptible (81.25%) 5 days post-infection.

The infection rates of *G. hamiltonii* and *T. scripta* differed after pathogen challenge. *T. scripta* showed significantly low (*p* < 0.05) infection rates (12.5%), while new *G. hamiltonii* hatchlings were susceptible (infection rate 81.25%) 5 days post-pathogen challenge (Figure 1).

### 3.2. High-Throughput Sequencing and Transcriptome Reconstruction

**Figure 2:**
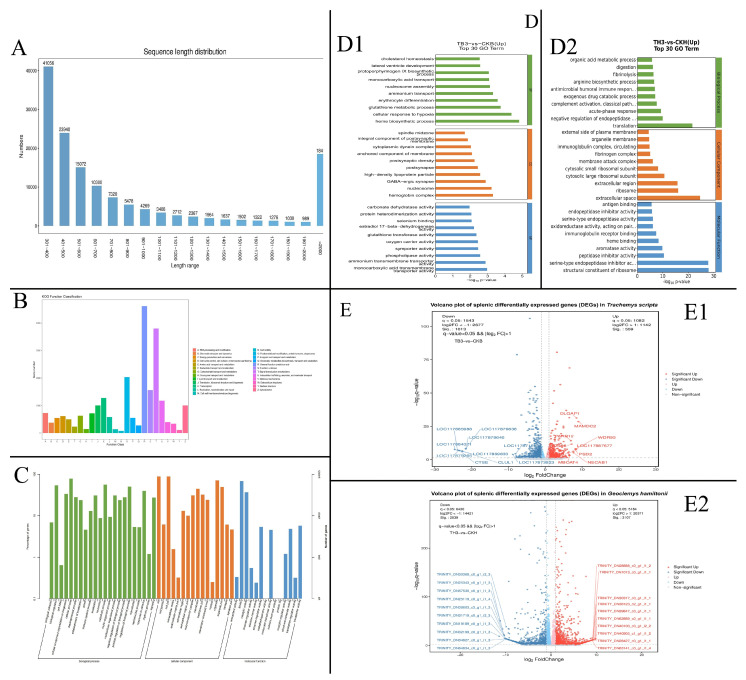
(**A**) A sequence length distribution graph, with 72.3% falling within the 1–200 nt range. (**B**) A total of 23,415 unigenes were finally mapped onto 25 different KOG categories. “General function prediction” (4607; 19.7%) was the largest KOG category, followed by “Signal transduction” (3799; 16.2%) and “Post-translational modification” (2031; 8.7%), while “Cellular motility” (80; 0.3%) was the smallest. (**C**) A total of 24,005 unigenes were mapped to 52 Gene Ontology (GO) terms, consisting of three major categories. (**D**) The top 30 GO enrichment subclasses. (**D1**) (TB3 vs CKB) shows enrichment in cholesterol/heme metabolism and synaptic function; (**D2**) (TH3 vs CKH) highlights immune response, organic acid metabolism and ribosome-related functions. (**E**) A volcano plot showing differentially expressed genes (red dots) and non-significantly differential genes (blue dots). (**E1**) shows DEGs between TB3 and CKB in *Trachemys scripta*, with 559 significantly upregulated and 1013 downregulated genes. (**E2**) displays DEGs between TH3 and CKH in *Geoclemys hamiltonii*, with 3107 upregulated and 2039 downregulated genes. Significance was defined as q < 0.05 and |log2 fold change| > 1.

cDNA specimens derived from all tissues of *A. hydrophila*-challenged *G. hamiltonii* and *T. scripta* underwent paired-end sequencing utilizing an Illumina NovaSeq 6000 instrument. Following quality filtration, the *G. hamiltonii* infection cohorts yielded 48.01 million clean reads (from 49.35 million raw reads), while the *T. scripta* samples produced 47.46 million clean reads (from 47.79 million raw reads). Transcriptomic reconstruction employing a comprehensive de novo assembly pipeline generated transcriptomic resources spanning 159,649,411 base pairs, comprising 144,127 non-redundant unigenes. Transcriptomic reconstruction employing a comprehensive de novo assembly pipeline generated transcriptomic resources spanning 159,649,411 base pairs, comprising 144,127 non-redundant unigenes with an N50 of 2032 bp. Quality assessment revealed that the GC content of the assembled unigenes was approximately 45.2%, consistent with values reported for other reptilian transcriptomes. Read duplication levels were low, indicating minimal PCR amplification bias. Transcript length distribution showed that the majority of unigenes (72.3%) fell within the 1–200 nt range, with a gradual decrease in frequency as length increased (Figure 2A). Collectively, these quality metrics support the reliability of the de novo assembly for downstream analyses.

### 3.3. Functional Characterization of Transcriptomic Elements

The 144,127 non-redundant unigenes identified in splenic tissues of *G. hamiltonii* and *T. scripta* underwent comprehensive functional annotation through BLASTX homology searches against multiple proteomic repositories (National Center for Biotechnology Information non-redundant [Nr], Swiss-Prot). Sequence homology analysis revealed 45,695 (31.70%) and 27,041 (18.76%) unigenes exhibiting significant matches (E-value < 1 × 10^−10^) in the Nr and Swiss-Prot databases, respectively (Table 2). Evolutionarily conserved protein domains were classified via Eukaryotic Orthologous Groups (KOG) analysis, with 23,415 unigenes systematically mapped to 25 functional clusters (Figure 2B). The predominant functional cluster encompassed “General functional prediction” (4607 unigenes; 19.7%), followed by “Signal transduction mechanisms” (3799; 16.2%) and “Post-translational regulation/proteostasis” (2031; 8.7%). In contrast, “Cellular motility” represented the smallest category (80 unigenes; 0.3%). Gene Ontology (GO) categorization assigned 24,005 unigenes to 52 functional terms across three ontological domains (Figure 2C): biological processes: 134,394 unigenes (22 subcategories); cellular components: 113,094 unigenes (16 subcategories); molecular functions: 30,892 unigenes (14 subcategories). Among the 144,127 non-redundant unigenes, 40,787 (28.30%) were annotated in the Nr database, 25,070 (17.39%) in Swiss-Prot, and 4300 (2.98%) in KEGG (Table 2). BLASTx annotation of all assembled transcripts did not identify any novel immune genes that are absent from the reference genome of *T. scripta* or other closely related species.

### 3.4. Immunological Transcript Profiling and Pathway Mapping

Transcriptomic profiling identified differentially expressed genes (DEGs) in response to *A. hydrophila* challenge compared to PBS controls within each species. In *G. hamiltonii*, 3666 genes were significantly upregulated, whereas in *T. scripta*, 3052 genes were significantly downregulated (FDR-adjusted *p* < 0.05, |log2FC| ≥ 1). These DEGs were functionally stratified into three GO domains, with the 30 most enriched subcategories visualized in Figure 2D. Comparative expression analysis was conducted implementing DESeq’s differential-expression algorithm (Benjamini–Hochberg adjusted (*p* < 0.05, |log2FC| > 1) to standardize inter-species variations. Transcriptomic divergences were graphically represented through scatterplot distributions and volcano plots highlighting replicate consistency (Figure 2E). To further characterize species-specific immune activation patterns, we compared the infection-induced transcriptional responses between the two species. These inter-species comparisons revealed both conserved and divergent immune signatures. While core immune pathways were commonly activated across species, the magnitude and direction of expression changes varied considerably. Notably, a strong upregulation bias was observed in *G. hamiltonii* (3107 upregulated vs. 2039 downregulated DEGs), suggesting species-specific differences in immune activation thresholds. KEGG pathway enrichment analysis prioritized 20 metabolic routes significantly modulated by infection (Table 3), with the most enriched pathways being arginine biosynthesis (enrichment score: 7.1); complement/coagulation cascades (6.8); and steroid hormone biosynthesis (5.4).

Further comparative analysis of KEGG pathway enrichment revealed species-specific immune activation patterns. In *T. scripta*, pathways associated with complement and coagulation cascades (ko04610) and arginine biosynthesis (ko00220) were among the most highly enriched, indicating a robust and rapidly engaged innate immune response conducive to effective pathogen clearance. In contrast, *G. hamiltonii* exhibited enrichment in steroid hormone biosynthesis (ko00140), a pathway linked to immunomodulation and anti-inflammatory responses, which may contribute to delayed or less effective antimicrobial defense. These distinct pathway activation profiles align with the differential expression of key immune genes (IL18, STAT1, CD28) and provide a mechanistic basis for the disparate infection outcomes between the two species.

### 3.5. Screening of 19 Crucial Genes Involved in Immunity Progress

**Figure 3:**
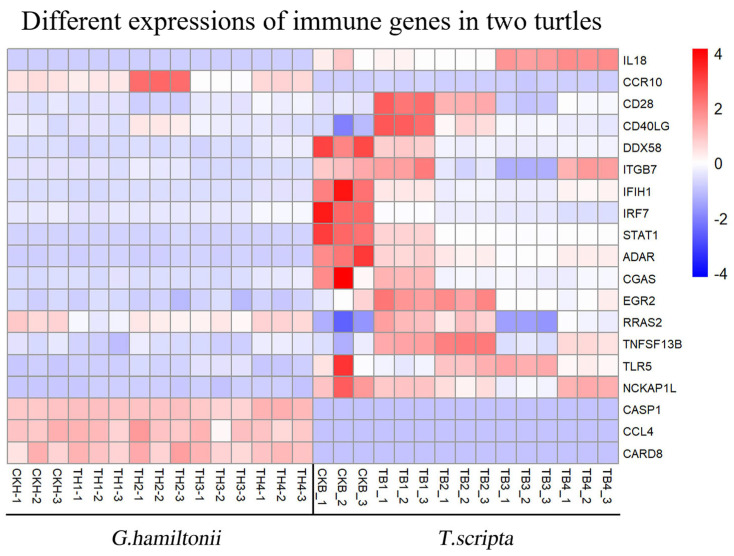
Heat map of deferentially expressed immune genes in the spleens of *Geoclemys hamiltonii* (H) and *Trachemys scripta* (B) after pathogen challenge. Prefixes: CK (PBS control), T (infected group). Species: H (*G. hamiltonii*), B (*T. scripta*). Time points: 1 (1 day), 2 (3 days), 3 (5 days), 4 (10 days). Biological replicates: 1, 2, 3. Example: TH4-3 represents replicate 3 of infected *G. hamiltonii* group on day 10.

To further filter the key genes involved in immunity progress, we employed R-studio to check their expression levels in the two species; genes which were deferentially up- or down-regulated not only on different dpi but also in the two turtles species were selected. As shown in Figure 3, 19 crucial genes involved in immunity progress were screened, systematically categorized into six functional groups based on their primary immunological roles: (1) Pathogen Recognition (*TLR5*, *DDX58*, *CGAS*, *IFIH1*), (2) Signal Transduction(*STAT1*, *RRAS2*, *IRF7*, *EGR2*), (3) Inflammasome & Pyroptosis (*CASP1*, *CARD8*), (4) Cytokines & Chemokines (*IL18*, *CCL4*, *TNFSF13B*), (5) Adaptive Immunity & Lymphocyte Regulation (*CD28*, *CD40LG*, *ITGB7*, *CCR10*), and (6) Immune Modulation (*ADAR*, *NCKAP1L*). This classification clearly distinguishes between components of the innate (Groups 1–4) and adaptive (Group 5) immune systems.

The Pathogen Recognition group included *TLR5* (Toll Like Receptor 5), which participates in the activation of innate immunity and inflammatory responses [18]; *DDX58* (RNA Sensor RIG-I), an innate immune receptor that senses cytoplasmic viral nucleic acids and activates a downstream signaling cascade, leading to the production of type I interferons and pro-inflammatory cytokines [30]; *CGAS* (Cyclic GMP-AMP Synthase), a nucleotidyltransferase that catalyzes the formation of cyclic GMP-AMP (2′,3′-cGAMP) from ATP and GTP and plays a key role in innate immunity [31]; *IFIH1* (Interferon Induced With Helicase C Domain 1), an innate immune receptor that acts as a cytoplasmic sensor of viral nucleic acids and plays a major role in sensing viral infection and activating a cascade of antiviral responses [32].

The Signal Transduction group comprised key signaling molecules like *STAT1* (Signal Transducer and Activator of Transcription 1), which transduces signals from the cytoplasmic domains of transmembrane receptors into the nucleus, where it regulates gene expression [33]; *RRAS2* (RAS Related 2), a member of the R-Ras subfamily of Ras-like low-molecular-weight GTPases, which is considered to regulate cell proliferation and differentiation via the *RAS*/*MAPK* signaling pathway [34]; *IRF7* (Interferon Regulatory Factor 7), a key transcriptional regulator of type I interferon (IFN)-dependent immune responses that plays a critical role in the innate immune response against DNA and RNA viruses [35]; *EGR2* (Early Growth Response 2), a transcription factor essential for the development and maintenance of myelin structure in the nervous system [36]. Beyond its neurodevelopmental role, EGR2 functions as a key regulator of immune responses, where it suppresses pro-inflammatory cytokine production (e.g., TNF-α, IL-6) and promotes anti-inflammatory macrophage polarization and regulatory T-cell differentiation. This immunomodulatory function is particularly relevant in bacterial infections, where EGR2 helps constrain excessive inflammation while influencing pathogen clearance efficiency. In the context of this study, differential EGR2 expression between the two species may contribute to the divergent inflammatory responses and infection outcomes observed.

Genes involved in Inflammasome & Pyroptosis included *CASP1* (Caspase-1), a cysteine protease that regulates inflammatory processes through its capacity to process and activate the interleukin-1-beta (*IL1B*), *IL18*, and *IL33* precursors [37]; *CARD8* (Caspase Recruitment Domain Family Member 8), an inflammasome sensor that mediates inflammasome activation in response to various pathogen-associated signals, leading to subsequent pyroptosis of CD4(+) T-cells and macrophages [38].

Furthermore, important Cytokines & Chemokines such as *IL18*(Interleukin 18), whose ability to potently enhance the production of interferon-γ indicates its crucial function as an immunomodulatory molecule [39]; *CCL4* (C-C Motif Chemokine Ligand 4), a monokine with inflammatory and chemokinetic properties [40]; *TNFSF13B* (TNF Superfamily Member 13b), which promotes B-cell maturation and antibody secretion [41].

The Adaptive Immunity group featured genes crucial for T-cell and B-cell function, including *CD28* (CD28 Molecule), which plays a crucial role in T-cell activation and signaling, promoting cell cycle entry and the production of various cytokines and chemokines, and also induces the expression of other costimulatory and regulatory molecules [42]; *CD40LG* (CD40 Ligand), which encodes a protein expressed on the surface of T-cells and regulates B-cell function by engaging *CD40* on the B-cell surface [43]; *ITGB7* (Integrin Subunit Beta 7), an adhesion molecule that mediates lymphocyte migration and homing to gut-associated lymphoid tissue [44]; *CCR10* (C-C Motif Chemokine Receptor 10), a receptor for chemokines *SCYA27* and *SCYA28*, which transduces signals by increasing intracellular calcium ion levels and stimulates chemotaxis in a pre-B-cell line [45].

Finally, the Immune Modulation category included regulators like *ADAR* (Adenosine Deaminase RNA-Specific), which catalyzes the hydrolytic deamination of adenosine to inosine in double-stranded RNA (dsRNA), referred to as A-to-I RNA editing [46]; *NCKAP1L* (NCK-Associated Protein 1 Like), which controls lymphocyte development, activation, proliferation, and homeostasis, as well as erythrocyte membrane stability, phagocytosis, and migration by neutrophils and macrophages [47].

The functional categories of these 19 genes (Table 4) highlight key components of both innate and adaptive immunity. The differential expression patterns between the two species—particularly the impaired upregulation of IL18, STAT1, and CD28 in *G. hamiltonii*—provide a mechanistic basis for its heightened susceptibility to *A. hydrophila* infection.

### 3.6. Immune Gene Expression Profiles in Different Tissues

**Figure 4:**
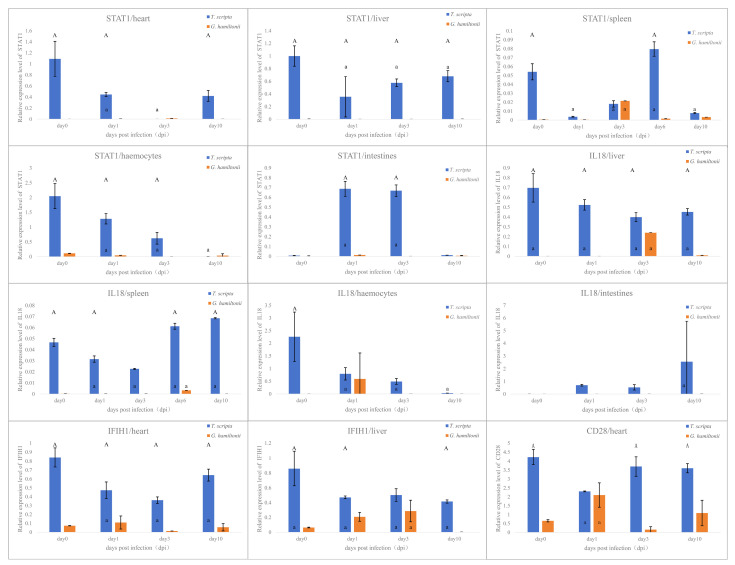
Temporal expression patterns of immune genes that exhibit significant differential expression in various tissues between *Geoclemys hamiltonii* and *Trachemys scripta* following *Aeromonas hydrophila* infection. Gene expression was normalized to the endogenous control *β-actin*. Data represent mean relative expression ± SE versus control (PBS-injected groups). Uppercase letters (A) above the bars indicate a significant difference between species at the same time point (*p* < 0.05). Lowercase letters (a) within the same species indicate a significant difference across time points compared to its own day 0 control (*p* < 0.05).

Expression level of all 19 genes was checked by RT-PCR at 0, 3, 5, and 10 days after bacterial challenge to assess differential immune responses in *G. hamiltonii* and *T. scripta*. The splenic expression profiles exhibited the highest concordance with the transcriptome findings, but in the other tissues, there were differences. Quantitative real-time PCR (qRT-PCR) analysis revealed that nine genes exhibited significant expression differences in the two turtle species after *A. hydrophila* exposure. A comparative analysis between species revealed that the expression levels of both *STAT1* and *IL18* were significantly higher in *T. scripta* than in *G. hamiltonii* across the majority of tissues examined (F(1, 4) = 62.69, *p* = 0.00138; F(1, 4) = 15.98, *p* = 0.0162). This relative upregulation in *T. scripta* was observed despite a temporal decrease in expression within *T. scripta* itself over the course of infection, potentially reflecting active consumption of these immune mediators during the robust defensive response. Additionally, *IFIH1* expression was significantly higher in the heart and liver of *T. scripta*, while *CD28* showed pronounced up-regulation specifically in the heart (F(1, 4) = 35.05, *p* = 0.00408; F(1, 4) = 114.7, *p* = 0.000431) (Figure 4), whereas *CASP1* and *TNFSF13B* were significantly suppressed in *T. scripta* (F(1, 4) = 1057, *p* = 5.34 × 10^−6^; F(1, 4) = 3,063,395, *p* = 6.39 × 10^−13^) compared to *G. hamiltonii*. This suppression reflects an adaptive immune strategy for *T. scripta*. As the core executor of inflammasome signaling, CASP1 drives pyroptosis and excessive inflammatory tissue damage when overactivated; TNFSF13B is a key regulator of B-cell maturation, whose aberrant upregulation triggers dysregulated humoral immunity. Given *T. scripta* has already established robust antimicrobial defense via the IL18-STAT1 axis and CD28-mediated adaptive immunity, this suppression balances efficient pathogen clearance and immune-mediated tissue injury, consistent with its low infection incidence. Additionally, *IRF7* and *ADAR* exhibited up-regulation in *T. scripta* hemocytes and intestinal tissues, but down-regulation in hepatic, cardiac, and splenic tissues. Conversely, *EGR2* and *DDX58* were up-regulated (F(1, 4) = 49.46, *p* = 0.00215) in *G. hamiltonii* post-infection. No significant differential expression was observed for the remaining genes in either species (Figure S1).

## 4. Discussion

Different turtle species may have evolved distinct immune response patterns due to variations in their native habitats and primary pathogens [48]. The significant differences in infection rates between *T. scripta* and *G. hamiltonii* infected with *A. hydrophila* suggest that they employ different immune response mechanisms. To elucidate the molecular basis of this differential susceptibility, we performed comparative splenic transcriptome analysis following bacterial challenge.

KEGG pathway enrichment analysis of the differentially expressed genes revealed species-specific immune activation patterns. In *T. scripta*, pathways associated with complement and coagulation cascades (ko04610) and arginine biosynthesis (ko00220) were among the most highly enriched. Complement activation facilitates direct pathogen opsonization and lysis, while arginine metabolism supports macrophage production of bactericidal nitric oxide; together, these pathways reflect a robust and rapidly engaged innate immune response conducive to effective pathogen clearance. In contrast, *G. hamiltonii* exhibited enrichment in steroid hormone biosynthesis (ko00140), a pathway linked to immunomodulation and anti-inflammatory responses. While such regulation may serve to limit excessive tissue damage, it may also delay or attenuate the initial antimicrobial defense, allowing *A. hydrophila* to proliferate and establish infection.

From the transcriptomic data, we identified 19 immune-related genes showing species-specific expression patterns, including *STAT1*, *IFIH1*, *RRAS2*, *CD28*, *IL18*, *CD40LG*, *CCR10*, *CASP1*, *ITGB7*, *CCL4*, *TLR5*, *EGR2*, *CGAS*, *DDX58*, *NCKAP1L*, *IRF7*, *ADAR*, *TNFSF13B*, and *CARD8* (Figure 3). These genes are involved in both innate and adaptive immunity. To further validate their expression dynamics, we performed qPCR analysis across five tissues (heart, liver, spleen, intestine, and blood) over the infection time course. The qPCR results confirmed that *IL18*, *STAT1*, and *CD28* exhibited the most pronounced differential expression between the two species, with *G. hamiltonii* showing impaired upregulation of these key immune genes. Notably, *IFIH1*, although primarily known as an antiviral gene, also contributes to NK cell activation [49], suggesting its potential involvement in the species-specific immune response. These distinct pathway activation profiles align with the differential expression of *IL18*, *STAT1*, and *CD28*, providing a mechanistic basis for the disparate infection outcomes between the two species. Collectively, our findings suggest that *G. hamiltonii*’s heightened susceptibility to *A. hydrophila* likely arises from impaired activation of early innate defense mechanisms.

*IL18* is a pro-inflammatory cytokine belonging to the *IL-*family [50]. It exists as an inactive precursor in the cytoplasm of various cells, such as macrophages and keratinocytes [51], and is activated by caspase-1 to stimulate interferon-γ [52] production and regulate Th1 and Th2 cell responses [52]. This gene also promotes epidermal-barrier repair [53]. Our results (Figure 4) showed that *IL18* and *STAT1* (downstream immune genes in the *IFN-γ* pathway which can mediate apoptosis activated by *MAPKs* [54]) were continuously highly expressed in tissues of *T. scripta*, such as the spleen and blood, which are the first to encounter the pathogen. We speculate that the sustained high expression of these genes helps the immune system rapidly and continuously respond to pathogen infection and initiate subsequent immune responses. In contrast, *IL18* expression remained unchanged in all tissues of *G. hamiltonii*, which also prevented its downstream genes in the IFN-γ pathway, such as *STAT1*, from being up-regulated. This provides more time for the pathogen to proliferate in the skin and internal tissues, causing severe damage in *G. hamiltonii*.

*CD28* is widely expressed on the surface of CD4+ and CD8+ T-cells and serves as a major co-stimulatory molecule for T-cells [55]. It participates in antigen presentations by binding to *CD80* (B7-1) and *CD86* (B7-2) on antigen-presenting cells (APCs), providing co-stimulatory signals that promote T-cell activation, proliferation, and synthesis of immune-related proteins [56]. Our results (Figure 3) showed that *CD28* was significantly up-regulated in the spleen and blood, and other tissues that may come into contact with the pathogen in *T. scripta*, starting from 1 day post-infection. This up-regulation likely enables *T. scripta* to initiate precise adaptive immune responses against *A. hydrophila*, leading to pathogen clearance and lower infection rates, as confirmed by our infection rate experiments (Figure 1). Conversely, in *G. hamiltonii*, *CD28* was not significantly up-regulated in the spleen, and although it was up-regulated in the blood, its peak expression level occurred later than in *T. scripta*. This delay prevents the rapid clearance of pathogens through T-cell activation, proliferation, antigen presentation, and antibody synthesis, resulting in higher infection rates.

Our findings indicate that compared to *T. scripta*, *G. hamiltonii* failed to rapidly initiate effective defense against *A. hydrophila* infection during both the early innate immune phase and the subsequent adaptive immune phase. However, *G. hamiltonii* can activate adaptive immune responses to resist pathogen infection after 3–4 days (Figure 4. *IFIH1*/hemocytes, *CD28*/heart, *CD28*/liver, *CD28*/intestines, *CD28*/hemocytes). This may be because, in their native habitats, *G. hamiltonii* are exposed to a higher abundance and diversity of pathogens. High expression of *IL18* could cause more tissue damage due to pro-inflammatory reactions [57], while a strategy focusing on long-term adaptive immune responses might enhance their fitness in the wild [58]. However, low *IL18* expression may slow the repair of the epidermal barrier [59], making *G. hamiltonii* more susceptible to skin ulcer disease.

In conclusion, our findings identify *IL18* as a primary contributor to the divergent immune trajectories between *G. hamiltonii* and *T. scripta*, with dysregulated *CD28* induction further compromising adaptive immunity in the endangered *G. hamiltonii*. The absence of *IL18*/*STAT1* signaling in *G. hamiltonii* correlates with delayed *IFN-γ* pathway activation, impaired epidermal-barrier restoration, and failure to initiate *CD28*-mediated T-cell responses. In future studies, we will explore therapeutic rescue strategies using *IL*18/*CD28* ectopic expression in *G. hamiltonii* primary cells and ex vivo tissue models. This approach may validate targets for enhancing *A. hydrophila* resistance in captive populations.

Compared with previous studies on immune responses in reptiles and other ectothermic vertebrates, our findings reveal both conserved and species-specific features. For example, in crocodilians and turtles, bacterial infections typically induce a strong inflammatory response characterized by the upregulation of cytokines such as IL-1β and TNF-α, refs [60,61,62]. similar to the pro-inflammatory signature observed in *G. hamiltonii* in the present study. However, unlike the robust antimicrobial peptide response reported in some ectothermic vertebrates (e.g., amphibians and fish) [63], our transcriptomic data did not show significant induction of known antimicrobial peptide genes in *G. hamiltonii* following bacterial challenge. This discrepancy may reflect lineage-specific evolutionary trajectories of innate immune components. Furthermore, compared with the delayed and less pronounced immune response described in certain reptile species under hypothermic conditions, the rapid transcriptional activation observed in our study under controlled thermal conditions highlights the importance of environmental temperature in modulating ectothermic immune function. Collectively, these comparisons underscore the need for more comparative studies across ectothermic lineages to better understand the evolution of vertebrate immunity.

Several limitations of this study should be acknowledged. First, independent replicate infection experiments were not conducted for the infection group, primarily due to the logistical and administrative challenges associated with obtaining sufficient numbers of newly hatched *G. hamiltonii*, a CITES Appendix I-listed species. To mitigate this constraint, we monitored clinical incidence trends across sampling time points and confirmed their consistency with the infection group. In addition, transcriptomic data from three independent biological replicates and qPCR results provided convergent evidence for the species-specific infection responses. Future studies with independent experimental replicates are warranted to further validate the robustness of the infection model. Second, our comparative analysis included only two species. Consequently, while we observed species-specific transcriptional differences following infection, we cannot infer the evolutionary directionality of these responses or determine which expression pattern represents the ancestral or derived state. Third, our conclusions are based solely on mRNA transcript levels measured by RNA-seq. We recognize that post-transcriptional regulation may lead to discordance between transcript abundance and functional protein levels, and future proteomic analyses would be valuable. Fourth, this study used a single dose and a single strain of the pathogen. It remains unknown whether the observed transcriptional responses are dose-dependent or strain-specific. Further experiments using a range of pathogen doses and multiple strains are required to generalize our findings.

Overall, this study elucidates divergent immune mechanisms in chelonians, while addressing a critical gap in conservation biology by defining the immunological basis of extinction vulnerability in the IUCN Endangered *G. hamiltonii*. Our findings demonstrate that divergence in the *IL18*-*STAT1* innate immune axis and reduced *CD28*-mediated adaptive immunity are core drivers of the species’ extreme susceptibility to *A. hydrophila* infection, a key threat driving its ongoing population collapse. These findings not only reveal immune fragility as an underappreciated intrinsic determinant of extinction risk in endangered vertebrates, but also provide actionable biomarkers (*IL18*/*CD28*) for susceptibility screening, and establish a foundational framework for immunotherapy-assisted conservation of this imperiled species and other threatened chelonians.

## Figures and Tables

**Table 1:** Sequences of primers used in this study. Primers were designed to target evolutionarily conserved sequences shared by both species.

Primers	Sequence (5′–3′)	Accession Number
β-actin F	TTGGGTATGGAATCCTGTGGC	FJ514826.1
β-actin R	AGGGCTGTGATCTCCTTCTGCA	FJ514826.1
IL18 F	GCAAACTACGCTCTACAGCACCA	XM_034754572.1
IL18 R	GATCTGTCATAGGCTCAAATACACTCA	XM_034754572.1
CCR10 F	TTTCCAAGGCAGCCAAGGG	XM_034756282.1
CCR10 R	TGTCCAGCAGCACCATCAGG	XM_034756282.1
CD28 F	CCATGGCAGATGCGGATACT	XM_034786510.1
CD28 R	ATTGCTGTGATGCCTCCTTTGT	XM_034786510.1
CD40LG F	GTGTTTACTGTGCCCTACAATCCA	XM_034782600.1
CD40LG R	TTCTTCATAGCAGGTCACTTCTCAATA	XM_034782600.1
DDX58 F	CCCAATAGATGCGTTGTCCC	XM_034774409.1
DDX58 R	ATTGTATTTCCGTAAGTGTTCAGTGTAA	XM_034774409.1
ITGB7 F	CAAGGAGAAGACGGACGACG	XM_034791704.1
ITGB7 R	CCCGATGACCACGATACCC	XM_034791704.1
IFIH1 F	ACAGAACAAGCCTACTCTACCTACACAG	XM_034786312.1
IFIH1 R	AAGAAAGTCTCAGCCAGCGAAA	XM_034786312.1
IRF7 F	CAGCAAGAGGGAGATGACGAGT	XM_034770403.1
IRF7 R	ACAAGGAGGAAGCAATCAGAGC	XM_034770403.1
STAT1 F	GAAAATGAATAATTCCCAGAGTAGCCT	XM_034785098.1
STAT1 R	CCCATCTGTCCTCTACCCTGTTG	XM_034785098.1
ADAR F	GGTCAGCATTGGCACGGGTA	XM_034792051.1
ADAR R	GCTCTGTTCTGTGGAGGAGGGAT	XM_034792051.1
CGAS F	CTTCCCTTCGCCCATTGACC	XM_034766204.1
CGAS R	CCCACGCCACCAGACTTGAC	XM_034766204.1
EGR2 F	GACATGAGTGACAAGAGGTCCCTG	XM_034777878.1
EGR2 R	AAGAGGCTGTGGAGGAAGAAGTG	XM_034777878.1
RRAS2 F	GGGTAGGACAATACAGGCAAACTT	XM_034770110.1
RRAS2 R	CCAAAACATAGCCAAGTACAAGAAGT	XM_034770110.1
TNFSF13B F	GCAATGCTCCTGTCCTCTTCTC	XM_034758181.1
TNFSF13B R	TCATCACCTTCACCTGCCTCA	XM_034758181.1
TLR5 F	CTCGTAGTTGGGAGGGTTGTTC	XM_034764647.1
TLR5 R	TTGTTAATTCCATTTGCTGGTGAC	XM_034764647.1
NCKAP1L F	GTTCTTCGGCAGTCTGAAAGGGTA	XM_034792820.1
NCKAP1L R	AGTTCACCTCGTCACGGCACA	XM_034792820.1
CASP1 F	TGGATGTGAAAGGGATGGAGAAG	XM_034752746.1
CASP1 R	AGCCCTGACGCCGTGAGA	XM_034752746.1
CCL4 F	GTCGCTGCCTTCTGCTCCC	XM_034752586.1
CCL4 R	TACTCCTGAACCCAGTCCTCTTTG	XM_034752586.1
CARD8 F	ACCTGACATCGTTGCGTTGAAA	XM_034768860.1
CARD8 R	GCTCTGGGTGTAAATCTCCGTGTAT	XM_034768860.1

**Table 2:** Annotation of *Geoclemys hamiltonii* and *Trachemys scripta* transcriptome unigenes; number of annotated unigenes in databases; percentages of annotated unigenes.

Database	Total Unigenes	Annotated Unigenes	Percentage
GO	144,127	22,225	15.42%
KEGG	144,127	4300	2.98%
NR	144,127	40,787	28.30%
PFAM	144,127	16,713	11.59%
KOG	144,127	18,639	12.95%
SWISSPROT	144,127	25,070	17.39%
EGGNOG	144,127	31,589	21.92%

**Table 3:** KEGG enrichment analysis during *Aeromonas hydrophila* infection and the top 20 pathways, with the most enriched pathways being arginine biosynthesis (enrichment score: 7.1); complement and coagulation cascades (6.8); and steroid hormone biosynthesis (5.4).

Pathway ID	Term	Enrichment_Score	ListHits
ko03010	Ribosome	4.5	79
ko04610	Complement and coagulation cascades	6.8	34
ko05150	Staphylococcus aureus infection	4.1	29
ko04145	Phagosome	2.6	42
ko05322	Systemic lupus erythematosus	3.3	29
ko04080	Neuroactive ligand-receptor interaction	3.3	29
ko00220	Arginine biosynthesis	7.1	11
ko05152	Tuberculosis	2.5	38
ko00140	Steroid hormone biosynthesis	5.4	12
ko05204	Chemical carcinogenesis	4.3	12
ko05323	Rheumatoid arthritis	2.5	25
ko05320	Autoimmune thyroid disease	2.5	21
ko05330	Allograft rejection	2.5	21
ko05310	Asthma	3.0	16
ko05143	African trypanosomiasis	2.9	17
ko05140	Leishmaniasis	2.4	22
ko00980	Metabolism of xenobiotics by cytochrome P450	4.2	9
ko05416	Viral myocarditis	2.2	23
ko05146	Amoebiasis	2.2	23
ko00830	Retinol metabolism	3.8	9

**Table 4:** Summary of the 19 immune-related genes differentially expressed between *Geoclemys hamiltonii* and *T. scripta* following *Aeromonas hydrophila* infection.

Functional Category	Gene	Immune Role	Key Reference(s)
Pathogen Recognition	*TLR5*	Recognizes bacterial flagellin; activates innate immunity and inflammatory responses	[18]
Pathogen Recognition	*DDX58*	Cytoplasmic viral RNA sensor; induces type I interferons and pro-inflammatory cytokines	[30]
Pathogen Recognition	*CGAS*	DNA sensor; produces cGAMP to activate STING-mediated innate immunity	[31]
Pathogen Recognition	*IFIH1*	Cytoplasmic viral RNA sensor; enhances NK cell activation and antiviral responses	[32]
Signal Transduction	*STAT1*	Mediates IFN-γ signaling; regulates apoptosis and antimicrobial gene expression	[33]
Signal Transduction	*RRAS2*	GTPase involved in RAS/MAPK signaling; regulates cell proliferation and differentiation	[34]
Signal Transduction	*IRF7*	Master regulator of type I interferon responses; critical for antiviral immunity	[35]
Signal Transduction	*EGR2*	Transcription factor; involved in immune regulation and myelin development	[36]
Inflammasome & Pyroptosis	*CASP1*	Activates IL-1β and IL-18; mediates pyroptosis and inflammatory responses	[37]
Inflammasome & Pyroptosis	*CARD8*	Inflammasome sensor; triggers pyroptosis in response to pathogen signals	[38]
Cytokines & Chemokines	*IL18*	Pro-inflammatory cytokine; induces IFN-γ production, promotes Th1 responses, and facilitates epidermal barrier repair	[39]
Cytokines & Chemokines	*CCL4*	Chemokine; recruits immune cells to infection sites	[40]
Cytokines & Chemokines	*TNFSF13B*	Promotes B-cell maturation and antibody secretion	[41]
Adaptive Immunity	*CD28*	Co-stimulatory molecule essential for T-cell activation, proliferation, and cytokine production	[42]
Adaptive Immunity	*CD40LG*	T-cell surface protein; regulates B-cell function via CD40 engagement	[43]
Adaptive Immunity	*ITGB7*	Integrin; mediates lymphocyte homing to gut-associated lymphoid tissue	[44]
Adaptive Immunity	*CCR10*	Chemokine receptor; directs lymphocyte migration to skin	[45]
Immune Modulation	*ADAR*	RNA editing enzyme; modulates immune responses by editing dsRNA	[46]
Immune Modulation	*NCKAP1L*	Regulates lymphocyte activation, proliferation, and phagocytosis in macrophages and neutrophils	[47]

## Data Availability

All raw sequencing data have been deposited in NCBI SRA under accession number [PRJNA1293693].

## Supplementary Materials

The following supporting information can be downloaded at: https://www.mdpi.com/article/10.3390/genes17040436/s1, Figure S1: Spatiotemporal Expression Patterns of 19 Immune Genes Across Multiple Tissues Data represent mean ± SE relative to the control. Different letters denote significant differences between treatment groups (*p* < 0.05), vertical bars indicate standard error (SE), Figure S2: Sequence alignment of 19 immune genes between *G. hamiltonii* and *T. scripta*. The query sequence (top) represents *G. hamiltonii*, and the sbjct sequence (bottom) represents *T. scripta*. Nucleotide identities are indicated by the vertical line (|) between sequences. The red boxes highlight the forward and reverse primer binding sites located within conserved regions; Code S1: The R code used for the analysis of the expression levels of these immune-related DEGs.
