# Supplementary material for: Divergent IL18-STAT1 Immune Responses Underlie Differential Susceptibility to Aeromonas hydrophila in Geoclemys hamiltonii and Trachemys scripta: A Comparative Transcriptomic Perspective
Source: Genes (Basel). 2026 Apr 9;17(4):436. doi: 10.3390/genes17040436 (PMC13116093; doi:10.3390/genes17040436)
Supplement: Supplementary file 1 [file genes-17-00436-s001.zip › Figure S2/ADAR.pdf]

PREDICTED: Trachemys scripta elegans adenosine deaminase RNA specific (ADAR), mRNA

Sequence ID: XM_034792051.1 **Length:** 6255 **Number of Matches:** 1

Range 1: 1 to 6255 GenBank Graphics ▼ Next Match ▲ Previous Match

Score	Expect	Identities	Gaps	Strand
11551 bits(6255)	0.0	6255/6255(100%)	0/6255(0%)	Plus/Plus

```
Query  1     TTAGTTTTTAAGGTGCCACCGGACTCCTCGTTGTTTTTGCGTAGTACGGTTTGGTAAAAA  60
Sbjct  1     TTAGTTTTTAAGGTGCCACCGGACTCCTCGTTGTTTTTGCGTAGTACGGTTTGGTAAAAA  60

Query  61    GTTTGTAAGTCTGTTTAAACCAGAGCCAGCTGCAGCAGGAGATGGGTAGAAGGGGGTGTA  120
Sbjct  61    GTTTGTAAGTCTGTTTAAACCAGAGCCAGCTGCAGCAGGAGATGGGTAGAAGGGGGTGTA  120

Query  121   TGTGTGAGCACTGCATGAAAGGGTTCCCTTTCTTTCCTAGAGGGATCCTGGAACAGACCC  180
Sbjct  121   TGTGTGAGCACTGCATGAAAGGGTTCCCTTTCTTTCCTAGAGGGATCCTGGAACAGACCC  180

Query  181   GCCCTGGTGAAACAGAACACCAGGCAAAACAGAGAAGTGAGAGTTAGCTGGGAGAAAATG  240
Sbjct  181   GCCCTGGTGAAACAGAACACCAGGCAAAACAGAGAAGTGAGAGTTAGCTGGGAGAAAATG  240

Query  241   AAACCGAAACTTAACTTGGAGCCATTTTAAGTTGATCGAGTGGATGATTACATTGTTAGC  300
Sbjct  241   AAACCGAAACTTAACTTGGAGCCATTTTAAGTTGATCGAGTGGATGATTACATTGTTAGC  300

Query  301   TCCCTGCTGGGCAGAGGGGAAGGGGTGACTAGCCTGTTTCTGTTGTTTGTAAAAAGTACC  360
Sbjct  301   TCCCTGCTGGGCAGAGGGGAAGGGGTGACTAGCCTGTTTCTGTTGTTTGTAAAAAGTACC  360

Query  361   GAGGTACCGAACTTTTCTGTATCTAGGAGCCATTTGTGAGCTCTGTCTTCAGCTGTTCGG  420
Sbjct  361   GAGGTACCGAACTTTTCTGTATCTAGGAGCCATTTGTGAGCTCTGTCTTCAGCTGTTCGG  420

Query  421   CTGGCACAGGGAGGAGCTGCTCCCGCTGGTGTTTCAGTTGCTTTCAGCCTCTCCTAGCAG  480
Sbjct  421   CTGGCACAGGGAGGAGCTGCTCCCGCTGGTGTTTCAGTTGCTTTCAGCCTCTCCTAGCAG  480

Query  481   TGTGAGTCTGTGGTTAGAGAGATGCGGTGGAGGAGGCCATAGATATCTTTTGTTGGACCA  540
Sbjct  481   TGTGAGTCTGTGGTTAGAGAGATGCGGTGGAGGAGGCCATAGATATCTTTTGTTGGACCA  540

Query  541   ACTTCTGTTGGCGGAGAGAGAGAAGCTTTTGAGTTACACAGATCTCTTCTTCAGGTCTGG  600
Sbjct  541   ACTTCTGTTGGCGGAGAGAGAGAAGCTTTTGAGTTACACAGATCTCTTCTTCAGGTCTGG  600

Query  601   GAAAGGCAGTAAGAGTGTCACAGCTAAGTACAAGATCAGACAGATAGTTTAGCATAAGTA  660
Sbjct  601   GAAAGGCAGTAAGAGTGTCACAGCTAAGTACAAGATCAGACAGATAGTTTAGCATAAGTA  660

Query  661   ATTAGCACTATTCTAAGGGACCATTCGAGGTGAAGTGGCGATAGGTTACAGCTTAGGAAC  720
Sbjct  661   ATTAGCACTATTCTAAGGGACCATTCGAGGTGAAGTGGCGATAGGTTACAGCTTAGGAAC  720

Query  721   AGTTCTTTACCTTCTGATCAATTAAATGAATTGTACTTGTCAGCTTGGGCAAAAACAAGG  780
Sbjct  721   AGTTCTTTACCTTCTGATCAATTAAATGAATTGTACTTGTCAGCTTGGGCAAAAACAAGG  780

Query  781   CCTCTGTGCAGCAATTGTGCGAGTGGAAAGCGAACCACAGCTGCCTCTCTTCTGGTGTGC  840
Sbjct  781   CCTCTGTGCAGCAATTGTGCGAGTGGAAAGCGAACCACAGCTGCCTCTCTTCTGGTGTGC  840

Query  841   AAGAGCCCCGATTCACACTTGCATTCCAGCAGCACGCTATGAACACAGGTTTTGGTCGAG  900
Sbjct  841   AAGAGCCCCGATTCACACTTGCATTCCAGCAGCACGCTATGAACACAGGTTTTGGTCGAG  900

Query  901   GCAAAGGCTCTTATCAAACACACCCAAAACATAGCTACTGCAGTCCTAATCCAGCCTTTT  960
Sbjct  901   GCAAAGGCTCTTATCAAACACACCCAAAACATAGCTACTGCAGTCCTAATCCAGCCTTTT  960

Query  961   TTAACAACTCTCTCATCCCACAGGGAAATAATCGCGACAGATTCAGAAACCAGCAGGTAC  1020
Sbjct  961   TTAACAACTCTCTCATCCCACAGGGAAATAATCGCGACAGATTCAGAAACCAGCAGGTAC  1020

Query  1021  AGTTCCTATTGGGACAAATCACTGAAGCTCCCCTGTACCCATTTTGGGAACAGAGGCCAC  1080
Sbjct  1021  AGTTCCTATTGGGACAAATCACTGAAGCTCCCCTGTACCCATTTTGGGAACAGAGGCCAC  1080

Query  1081  AAGTAGAGCAACACATCAGGGGTCCGCGAGCTGCTGTACCAATTCCTAGAGGGAGTGGGC  1140
Sbjct  1081  AAGTAGAGCAACACATCAGGGGTCCGCGAGCTGCTGTACCAATTCCTAGAGGGAGTGGGC  1140

Query  1141  ACAACAGCTGGGGCACAACAGCTGGGAGATTTAGGCCCCCCTATATCAGTCAGCAAACGC  1200
Sbjct  1141  ACAACAGCTGGGGCACAACAGCTGGGAGATTTAGGCCCCCCTATATCAGTCAGCAAACGC  1200

Query  1201  AAAGTTTACCGTCTTATTCCCCAACTCAGTACAACTACAGAAAATCAGAAAGGGAATTTG  1260
Sbjct  1201  AAAGTTTACCGTCTTATTCCCCAACTCAGTACAACTACAGAAAATCAGAAAGGGAATTTG  1260

Query  1261  ACCATTTCAACCTGAGTTTCCAGAGACTGACTGTTGCTGGGCAAAACAGGGAGCAAGAAA  1320
Sbjct  1261  ACCATTTCAACCTGAGTTTCCAGAGACTGACTGTTGCTGGGCAAAACAGGGAGCAAGAAA  1320

Query  1321  TTCTGACAATTTTAGGGCAGCTCAGGCAGGGGGAGTCCTGTACAGGTCGTGAACTCGCCC  1380
Sbjct  1321  TTCTGACAATTTTAGGGCAGCTCAGGCAGGGGGAGTCCTGTACAGGTCGTGAACTCGCCC  1380

Query  1381  ATAAACTTAAAACCCGAAAGAAAGAAGTCAATCATTATCTGTACAAACTTCTCCAGGAAG  1440
Sbjct  1381  ATAAACTTAAAACCCGAAAGAAAGAAGTCAATCATTATCTGTACAAACTTCTCCAGGAAG  1440

Query  1441  GTAAATTGCATAAAGAAGGAGAGAccccccccTTCTGGCGGATTGCGGACAAATCTGGCT  1500
Sbjct  1441  GTAAATTGCATAAAGAAGGAGAGACCCCCCCCTTCTGGCGGATTGCGGACAAATCTGGCT  1500

Query  1501  CTGTAGGGGCAGTGTGCGAAGGAAGTGCCACCACCGACGGGAGTCGTTGCAAAGACACAG  1560
Sbjct  1501  CTGTAGGGGCAGTGTGCGAAGGAAGTGCCACCACCGACGGGAGTCGTTGCAAAGACACAG  1560

Query  1561  CTTCTGAGAGTCAAGAAAAGGAAAGCCCCACTGTCGGCTCAGAAGACAGTACCGAGTCTC  1620
Sbjct  1561  CTTCTGAGAGTCAAGAAAAGGAAAGCCCCACTGTCGGCTCAGAAGACAGTACCGAGTCTC  1620

Query  1621  CCATCATGGCTGAAGTCAAGGAGAAAATCTGTAACTATTTGTTCAATGTCACAGACTCCA  1680
Sbjct  1621  CCATCATGGCTGAAGTCAAGGAGAAAATCTGTAACTATTTGTTCAATGTCACAGACTCCA  1680

Query  1681  CAGCATACAACCTTGCAAAAAAACATTGGTTTTTCAAAGGCCAAGGATGTTAACACCACTC  1740
Sbjct  1681  CAGCATACAACCTTGCAAAAAAACATTGGTTTTTCAAAGGCCAAGGATGTTAACACCACTC  1740

Query  1741  TCAGTGCCCTGGAAAAACTGGGAGAAGTCCACAAGGAGAACACAAACCCCCCAAAATGGT  1800
Sbjct  1741  TCAGTGCCCTGGAAAAACTGGGAGAAGTCCACAAGGAGAACACAAACCCCCCAAAATGGT  1800

Query  1801  CCCTCACTGAAAAGAAACGGGAGCGGATGCAGATCAAGTTAAAAGCCAATGAAGTAACGG  1860
Sbjct  1801  CCCTCACTGAAAAGAAACGGGAGCGGATGCAGATCAAGTTAAAAGCCAATGAAGTAACGG  1860

Query  1861  AAATGGCACCTCCCGCCCCAGAGCCAGAGTTTCCAGCTGCCTGCATAGAGCCGGATCCGC  1920
Sbjct  1861  AAATGGCACCTCCCGCCCCAGAGCCAGAGTTTCCAGCTGCCTGCATAGAGCCGGATCCGC  1920

Query  1921  AGGAGAGTGTGTTGCCCCTCACAGAGGTAAAGATGGAAGAAGAAGAAAACGTAAAGAATG  1980
Sbjct  1921  AGGAGAGTGTGTTGCCCCTCACAGAGGTAAAGATGGAAGAAGAAGAAAACGTAAAGAATG  1980

Query  1981  GACAGCAAGCCCCTGAGCAAACCGAACAGACTGACGCCAGTGCCCCTGACCCAGGCCTGC  2040
Sbjct  1981  GACAGCAAGCCCCTGAGCAAACCGAACAGACTGACGCCAGTGCCCCTGACCCAGGCCTGC  2040

Query  2041  GGGTCCGGAAGCCCAGATACCGCTTCATGAACTATGACAACTCTGAAAATGGCAAGTGGG  2100
Sbjct  2041  GGGTCCGGAAGCCCAGATACCGCTTCATGAACTATGACAACTCTGAAAATGGCAAGTGGG  2100

Query  2101  CCACCGATGACATTCCAGATGACTTGAATGCCATCAATAAGCAGGCGGATGAGTTGAGAT  2160
Sbjct  2101  CCACCGATGACATTCCAGATGACTTGAATGCCATCAATAAGCAGGCGGATGAGTTGAGAT  2160

Query  2161  GCATCATGGAATCCCCCTCATCTCCCAGCTACGCTGCCCAGTTTGATACGGCTTTCCAGT  2220
Sbjct  2161  GCATCATGGAATCCCCCTCATCTCCCAGCTACGCTGCCCAGTTTGATACGGCTTTCCAGT  2220

Query  2221  GTACGCCCTTAGAAAAGCTGATTGCCTGTCAGGAGAAGAATCCGGTCAGCGGCCTCATCG  2280
Sbjct  2221  GTACGCCCTTAGAAAAGCTGATTGCCTGTCAGGAGAAGAATCCGGTCAGCGGCCTCATCG  2280

Query  2281  AATATAGCCAGTACACTTACCAGCGCTGTGAATTTGCCCTTTTGGAGCAGAGCGGACCCT  2340
Sbjct  2281  AATATAGCCAGTACACTTACCAGCGCTGTGAATTTGCCCTTTTGGAGCAGAGCGGACCCT  2340

Query  2341  CGCATGAACCACGATTTAAGTTCCAGGCCGTGATAAACGGGCGCCGGTTCCCACCAGCGG  2400
Sbjct  2341  CGCATGAACCACGATTTAAGTTCCAGGCCGTGATAAACGGGCGCCGGTTCCCACCAGCGG  2400

Query  2401  AAGCAGGTAGCAAAAAACTGGCCAAGCAGGAGGCAGCTGCCAACGCCATGAAAATCCTGA  2460
Sbjct  2401  AAGCAGGTAGCAAAAAACTGGCCAAGCAGGAGGCAGCTGCCAACGCCATGAAAATCCTGA  2460

Query  2461  TGCGCGAAGCTGAGACCGAAGGGGAGGATGGCATGGAAGGGGAGGAATCATTCATCCCAG  2520
Sbjct  2461  TGCGCGAAGCTGAGACCGAAGGGGAGGATGGCATGGAAGGGGAGGAATCATTCATCCCAG  2520

Query  2521  ACAGCTCCGAAGCAGAGTTGCCTGCACGCCCAGAACCAGAGCCTCCATCTGCAGCGGCAC  2580
Sbjct  2521  ACAGCTCCGAAGCAGAGTTGCCTGCACGCCCAGAACCAGAGCCTCCATCTGCAGCGGCAC  2580

Query  2581  AGCTAAACTTGTCTTCCGGGAAGAACCCCATCAGCATATTAATGGAATATGGACAGAAAT  2640
Sbjct  2581  AGCTAAACTTGTCTTCCGGGAAGAACCCCATCAGCATATTAATGGAATATGGACAGAAAT  2640

Query  2641  CAGGGAGCACATGTGAATTCCAGCTGCTGTCTCAGGAGGGACCACCCCATGACCCCAAGT  2700
Sbjct  2641  CAGGGAGCACATGTGAATTCCAGCTGCTGTCTCAGGAGGGACCACCCCATGACCCCAAGT  2700

Query  2701  TCAAATACTGCGTGAAGGTGGGTGAGCAGACATTCCCTGCGGTGATAGCCAACAGCAAGA  2760
Sbjct  2701  TCAAATACTGCGTGAAGGTGGGTGAGCAGACATTCCCTGCGGTGATAGCCAACAGCAAGA  2760

Query  2761  AGGGAGCAAAGCAGATGGCAGCTGAGGTTGCTGTGAAGATCCTCCGTGGGGAGGCTGGGG  2820
Sbjct  2761  AGGGAGCAAAGCAGATGGCAGCTGAGGTTGCTGTGAAGATCCTCCGTGGGGAGGCTGGGG  2820

Query  2821  GGCAGTTCTTCCCAGAACAGCCCTCCGTAGAGGTCCCAAGCGAGCCCACCCTGGAGCCGC  2880
Sbjct  2821  GGCAGTTCTTCCCAGAACAGCCCTCCGTAGAGGTCCCAAGCGAGCCCACCCTGGAGCCGC  2880

Query  2881  CTGTTGCCCAGCCCCTGAATCCGGATGAGTCAAAGGCAGCAAAAGCCAAGGGCATCGGGG  2940
Sbjct  2881  CTGTTGCCCAGCCCCTGAATCCGGATGAGTCAAAGGCAGCAAAAGCCAAGGGCATCGGGG  2940

Query  2941  AGCTGATCAAATACCTCAATGCCAATCCCGTCAGTGGCCTGCTGGAATACGCCCGTTCCA  3000
Sbjct  2941  AGCTGATCAAATACCTCAATGCCAATCCCGTCAGTGGCCTGCTGGAATACGCCCGTTCCA  3000

Query  3001  ACGGGTTTGCAGCAGAGTTCAAAATGATCGATCAGACGGGACCACCTCACGATCCGAAGT  3060
Sbjct  3001  ACGGGTTTGCAGCAGAGTTCAAAATGATCGATCAGACGGGACCACCTCACGATCCGAAGT  3060

Query  3061  TTGTCTACCAGGCCAAGGTGGGAGGCCGCTGGTTCCCAGCCGTGACTGCACACAACAAAA  3120
Sbjct  3061  TTGTCTACCAGGCCAAGGTGGGAGGCCGCTGGTTCCCAGCCGTGACTGCACACAACAAAA  3120

Query  3121  AGCAAGGCAAGCAGGAAGCGGCCGACGCAGCGCTCAGAGTCCTGATTGGGGAAACAGAGA  3180
Sbjct  3121  AGCAAGGCAAGCAGGAAGCGGCCGACGCAGCGCTCAGAGTCCTGATTGGGGAAACAGAGA  3180

Query  3181  AGGCTGAGCGCACCGAAGGACTGAGCATCGCAGAGCTCCCTGTGAGCGGCAGTACCCTGC  3240
Sbjct  3181  AGGCTGAGCGCACCGAAGGACTGAGCATCGCAGAGCTCCCTGTGAGCGGCAGTACCCTGC  3240

Query  3241  ATGATCAGATAGCTATGCTCAGCCACCAGCGCTTCAATGCCCTCACTGCTCGCATCCAGC  3300
Sbjct  3241  ATGATCAGATAGCTATGCTCAGCCACCAGCGCTTCAATGCCCTCACTGCTCGCATCCAGC  3300

Query  3301  ACAGCCTGCTCGGACGGAAGATCCTGGCTGCAATCATCATGAGGCGAGGAGAAGAGGGCC  3360
Sbjct  3301  ACAGCCTGCTCGGACGGAAGATCCTGGCTGCAATCATCATGAGGCGAGGAGAAGAGGGCC  3360

Query  3361  TGGGAGTTGTGGTCAGCATTGGCACGGGTAATCGCTGCGTGAAGGGGGAGGAGCTGAGCC  3420
Sbjct  3361  TGGGAGTTGTGGTCAGCATTGGCACGGGTAATCGCTGCGTGAAGGGGGAGGAGCTGAGCC  3420

Query  3421  TGAAGGGCGAGACTGTGAATGACTGCCATGCAGAAATCATTTCCCGACGAGGCTTCGTGA  3480
Sbjct  3421  TGAAGGGCGAGACTGTGAATGACTGCCATGCAGAAATCATTTCCCGACGAGGCTTCGTGA  3480

Query  3481  GGTTTCTCTACAGCGAGCTGATGAAGTACAATCCCGCCGATCCCTCCTCCACAGAACAGA  3540
Sbjct  3481  GGTTTCTCTACAGCGAGCTGATGAAGTACAATCCCGCCGATCCCTCCTCCACAGAACAGA  3540

Query  3541  GCATATTTGAGTCAGCAGGAGAAAACAGGCTCAAAATAAAAGACGACGTTACCTTTCACC  3600
Sbjct  3541  GCATATTTGAGTCAGCAGGAGAAAACAGGCTCAAAATAAAAGACGACGTTACCTTTCACC  3600

Query  3601  TCTACGTCAGCACGGCGCCGTGTGGAGACGGGGCCCTCTTCGATAAATCCTGCAGTGACC  3660
Sbjct  3601  TCTACGTCAGCACGGCGCCGTGTGGAGACGGGGCCCTCTTCGATAAATCCTGCAGTGACC  3660

Query  3661  AGGCAAGCACAGCGGGGCAGAGCCAGCATCAGCCTCTCTTTGAGAATCCCAAGCAGGGTA  3720
Sbjct  3661  AGGCAAGCACAGCGGGGCAGAGCCAGCATCAGCCTCTCTTTGAGAATCCCAAGCAGGGTA  3720

Query  3721  AACTGCGCACCAAGGTGGAGAATGGGGAAGGCACCATTCCCGTGGAGTCGAGTGACATTG  3780
Sbjct  3721  AACTGCGCACCAAGGTGGAGAATGGGGAAGGCACCATTCCCGTGGAGTCGAGTGACATTG  3780

Query  3781  TGCCCACGTGGGATGGGATCCAGCATGGGGAGAGGCTGCGCACCATGTCCTGCAGCGACA  3840
Sbjct  3781  TGCCCACGTGGGATGGGATCCAGCATGGGGAGAGGCTGCGCACCATGTCCTGCAGCGACA  3840

Query  3841  AAATCCTGCGCTGGAATGTGCTTGGCCTGCAGGGGGCGCTGCTGTCGCATTTCATGCAGC  3900
Sbjct  3841  AAATCCTGCGCTGGAATGTGCTTGGCCTGCAGGGGGCGCTGCTGTCGCATTTCATGCAGC  3900

Query  3901  CAGTGTACCTCAGCTCCGTTACGCTCGGTTACCTATTCAGCCAGGGTCACCTGACACGTG  3960
Sbjct  3901  CAGTGTACCTCAGCTCCGTTACGCTCGGTTACCTATTCAGCCAGGGTCACCTGACACGTG  3960

Query  3961  CAATCTGCTGCCGCATGTCGAGAGACGGGAGCACGTTTGAAGCAGGTCTCCAGGCTCCGT  4020
Sbjct  3961  CAATCTGCTGCCGCATGTCGAGAGACGGGAGCACGTTTGAAGCAGGTCTCCAGGCTCCGT  4020

Query  4021  ATCGTGTTAAACATCCCGAGGTGGGGAGAGTTAGCGTGTACGACTCCGCCAGGCAGACGG  4080
Sbjct  4021  ATCGTGTTAAACATCCCGAGGTGGGGAGAGTTAGCGTGTACGACTCCGCCAGGCAGACGG  4080

Query  4081  GCAAGACCAAGGAGTCGAGTGTGAACTGGTGTCTTGCCGACGAAAGCGAGGTGGAAGTCC  4140
Sbjct  4081  GCAAGACCAAGGAGTCGAGTGTGAACTGGTGTCTTGCCGACGAAAGCGAGGTGGAAGTCC  4140

Query  4141  TGGATGGCACAAAAGGCAAAGTAGACGGACCAAAGCTAGAGGTGTCCCGCGTGTCCAAGA  4200
Sbjct  4141  TGGATGGCACAAAAGGCAAAGTAGACGGACCAAAGCTAGAGGTGTCCCGCGTGTCCAAGA  4200

Query  4201  GGAGCATGTTCGCTCTGTTCCAGCAGCTCTGTGCCAAGGCTGACCGCAAAGACCTGCAGA  4260
Sbjct  4201  GGAGCATGTTCGCTCTGTTCCAGCAGCTCTGTGCCAAGGCTGACCGCAAAGACCTGCAGA  4260

Query  4261  GCCTTGCGCTGTACTCAGATGCCAAGGAGGCAGCCGGGGCCTACCAGGGAGCCAAGCGGC  4320
Sbjct  4261  GCCTTGCGCTGTACTCAGATGCCAAGGAGGCAGCCGGGGCCTACCAGGGAGCCAAGCGGC  4320

Query  4321  ACTTCTTCAGGGCGCTGGAGGAGATGAGCTATGGGAGTTGGATCTGCAAACCCCAGGAAG  4380
Sbjct  4321  ACTTCTTCAGGGCGCTGGAGGAGATGAGCTATGGGAGTTGGATCTGCAAACCCCAGGAAG  4380

Query  4381  AGGAGAACTTCTCTCTCGCCGAGGCGTAACAAGCTGCCCTCtgtgtgtgtgtctgtgtgt  4440
Sbjct  4381  AGGAGAACTTCTCTCTCGCCGAGGCGTAACAAGCTGCCCTCTGTGTGTGTGTCTGTGTGT  4440

Query  4441  TCCTTTGGttttctttttttgatttttttAAATGGAGCCAGGAATGTTGAGTAGGATTGT  4500
Sbjct  4441  TCCTTTGGTTTTCTTTTTTTGATTTTTTTAAATGGAGCCAGGAATGTTGAGTAGGATTGT  4500

Query  4501  GGATTTCCCCTGGGTCTCTCCTCCTTTTGGTTATCCTGGCAGTACACGTTGATTCTGCTG  4560
Sbjct  4501  GGATTTCCCCTGGGTCTCTCCTCCTTTTGGTTATCCTGGCAGTACACGTTGATTCTGCTG  4560

Query  4561  GGAGGGACTCCCGTGTTCAGATTGCATTCTTTCTACGTGAGTGTACACTCAGCACACAAG  4620
Sbjct  4561  GGAGGGACTCCCGTGTTCAGATTGCATTCTTTCTACGTGAGTGTACACTCAGCACACAAG  4620

Query  4621  AGAATCCACACTGTCCACACGTTTAAATGAAACCACAGGCGAGCCCGCTCCCGTGTCCCT  4680
Sbjct  4621  AGAATCCACACTGTCCACACGTTTAAATGAAACCACAGGCGAGCCCGCTCCCGTGTCCCT  4680

Query  4681  TCATCACAGCAGTTGGGGGGAACGCAGCTTTTCGCTTGTCAGATGCATTGTCTTGATTGG  4740
Sbjct  4681  TCATCACAGCAGTTGGGGGGAACGCAGCTTTTCGCTTGTCAGATGCATTGTCTTGATTGG  4740

Query  4741  ATCCCTTTAAACAGTATGTGTGTACTTCCCAGCGTCTTACCCTCTCCCGAAACACTTCCC  4800
Sbjct  4741  ATCCCTTTAAACAGTATGTGTGTACTTCCCAGCGTCTTACCCTCTCCCGAAACACTTCCC  4800

Query  4801  TCTGATTTGCAAGATGATTCCTGCAGCTATTGGCAAAACTCCCCGGGACTTTTCCTTCCC  4860
Sbjct  4801  TCTGATTTGCAAGATGATTCCTGCAGCTATTGGCAAAACTCCCCGGGACTTTTCCTTCCC  4860

Query  4861  AGCAGTCCTGCTGGGTGGGCACAAATCGGTTTGAAGACAAGGTGATCTCTTGCAGCTGCT  4920
Sbjct  4861  AGCAGTCCTGCTGGGTGGGCACAAATCGGTTTGAAGACAAGGTGATCTCTTGCAGCTGCT  4920

Query  4921  ATTTAATTCTGGGATCTGCCTGTAGGGCATAGCAGAAGCCGGGTGGGGTTCAGGGCTAGT  4980
Sbjct  4921  ATTTAATTCTGGGATCTGCCTGTAGGGCATAGCAGAAGCCGGGTGGGGTTCAGGGCTAGT  4980

Query  4981  GGCAGATTGCATGTACTACCTGATCTAGATCCCCATAGTTAGCAGGCATATTCCTGGCTT  5040
Sbjct  4981  GGCAGATTGCATGTACTACCTGATCTAGATCCCCATAGTTAGCAGGCATATTCCTGGCTT  5040

Query  5041  TGGCGTGGTAGCCTCGTCTGACATACCCTGTTGATTATGAGGGTTGGGGCTTGATTTCTG  5100
Sbjct  5041  TGGCGTGGTAGCCTCGTCTGACATACCCTGTTGATTATGAGGGTTGGGGCTTGATTTCTG  5100

Query  5101  GAGTCCTGCTGAGATGGGCCATTCTAAAACCCTGCCTGACACTTCCCCCTGCGAGGGGGG  5160
Sbjct  5101  GAGTCCTGCTGAGATGGGCCATTCTAAAACCCTGCCTGACACTTCCCCCTGCGAGGGGGG  5160

Query  5161  CGAGTGGCACCCAAGGGTGGGGTTCCCCAGACTTGCCAGGTAGGGTGACACCACTTTAAG  5220
Sbjct  5161  CGAGTGGCACCCAAGGGTGGGGTTCCCCAGACTTGCCAGGTAGGGTGACACCACTTTAAG  5220

Query  5221  ATGCACGTCAGTGAAGTGGAGCGTTTCTTCCTCACCCCAGACACCCCTTCGGACCAGTTT  5280
Sbjct  5221  ATGCACGTCAGTGAAGTGGAGCGTTTCTTCCTCACCCCAGACACCCCTTCGGACCAGTTT  5280

Query  5281  CATGACTAGCCCATGGCTGGTTCGGCCGCAGCACAGCCCCTTTTACTGAAATGGGACCAG  5340
Sbjct  5281  CATGACTAGCCCATGGCTGGTTCGGCCGCAGCACAGCCCCTTTTACTGAAATGGGACCAG  5340

Query  5341  GGATCTTTTCGAGCCTGGCCTCTGTTTTTAAACCGTGCCTTGAAACGGTCATGAATTTTA  5400
Sbjct  5341  GGATCTTTTCGAGCCTGGCCTCTGTTTTTAAACCGTGCCTTGAAACGGTCATGAATTTTA  5400

Query  5401  TCTTGTCTACAGGCCTCAGGCCCAGCCAGCCTCTCCTCTTGGTTGTTTGCAGGAATCTAA  5460
Sbjct  5401  TCTTGTCTACAGGCCTCAGGCCCAGCCAGCCTCTCCTCTTGGTTGTTTGCAGGAATCTAA  5460

Query  5461  GACGCCTTCGTGGGTGATGGGATCTAAGGCAGGTTCTGTCTGTGTTGGGAACGCAGATTG  5520
Sbjct  5461  GACGCCTTCGTGGGTGATGGGATCTAAGGCAGGTTCTGTCTGTGTTGGGAACGCAGATTG  5520

Query  5521  GTGGCTGTGCTCCCGCCTGCCCTCTCTGCAGGCCTGTGTCTGGGCAGCCCTGGGGAATGG  5580
Sbjct  5521  GTGGCTGTGCTCCCGCCTGCCCTCTCTGCAGGCCTGTGTCTGGGCAGCCCTGGGGAATGG  5580

Query  5581  GGGTCGTGGGGCTGCCTGCCGGCACTGGGCTATCAGGGCCAGCAAATCCCATCCAGCCTG  5640
Sbjct  5581  GGGTCGTGGGGCTGCCTGCCGGCACTGGGCTATCAGGGCCAGCAAATCCCATCCAGCCTG  5640

Query  5641  CCTAGAATCCCAGGCCCCGGGTTACAGGATCAGCTTGCTCCGGAGGTCGGTTGCGTTTGG  5700
Sbjct  5641  CCTAGAATCCCAGGCCCCGGGTTACAGGATCAGCTTGCTCCGGAGGTCGGTTGCGTTTGG  5700

Query  5701  CCTGAGCTGGCCTGGGCTTTGTCAGAAGCTAGTCTGGGTTAGCCTGGCCCTGGGGGAAGG  5760
Sbjct  5701  CCTGAGCTGGCCTGGGCTTTGTCAGAAGCTAGTCTGGGTTAGCCTGGCCCTGGGGGAAGG  5760

Query  5761  GGCGAATTGAGTGGCGCTGTGAGCAGTCAAGTGCTGCACGGGGTGTACGTTGCAGTGCGG  5820
Sbjct  5761  GGCGAATTGAGTGGCGCTGTGAGCAGTCAAGTGCTGCACGGGGTGTACGTTGCAGTGCGG  5820

Query  5821  GAGGCTGGGGGCAAGGTCAGGGGGCGGGCTGCCCCTCTGAAGTCATTAAAGAGAAACCCA  5880
Sbjct  5821  GAGGCTGGGGGCAAGGTCAGGGGGCGGGCTGCCCCTCTGAAGTCATTAAAGAGAAACCCA  5880

Query  5881  GACCTGTTCCTACTCCACAGTTCTGGCATCTCCTTGTGCCGTCTCCACCCCGCCCCCATT  5940
Sbjct  5881  GACCTGTTCCTACTCCACAGTTCTGGCATCTCCTTGTGCCGTCTCCACCCCGCCCCCATT  5940

Query  5941  TATTTTATTTGTAATTCGTTGCACTGTCAGCGCGTCGGAGCAATTCCCGCCGTGCATCAT  6000
Sbjct  5941  TATTTTATTTGTAATTCGTTGCACTGTCAGCGCGTCGGAGCAATTCCCGCCGTGCATCAT  6000

Query  6001  TTGGGGACTGTCTCCTGCAAACTCGTCCTACCCCCGCCCCCTTTCTTTTCTGTCATGTCG  6060
Sbjct  6001  TTGGGGACTGTCTCCTGCAAACTCGTCCTACCCCCGCCCCCTTTCTTTTCTGTCATGTCG  6060

Query  6061  GATTCCTTCTCTTGTCGCACTGGCTCTGGAGAGCTGAATGGAAACAAATGTAAATTTATT  6120
Sbjct  6061  GATTCCTTCTCTTGTCGCACTGGCTCTGGAGAGCTGAATGGAAACAAATGTAAATTTATT  6120

Query  6121  CATGGGAGAACTGTAACCCTCCtttttattttatttaatttttGTAAGTCTGCTTTTAA  6180
Sbjct  6121  CATGGGAGAACTGTAACCCTCCTTTTTATTTTATTTAATTTTTGTAAGTCTGCTTTTAA  6180

Query  6181  GTGCCCCTCGATATCGATGAAATGAAGTCCTAACATTCCGCATaaaaataaaaaaaaaTT  6240
Sbjct  6181  GTGCCCCTCGATATCGATGAAATGAAGTCCTAACATTCCGCATAAAAATAAAAAAAAATT  6240

Query  6241  CTGTATTGTTAGACA  6255
Sbjct  6241  CTGTATTGTTAGACA  6255
```
