# Supplementary material for: Divergent IL18-STAT1 Immune Responses Underlie Differential Susceptibility to Aeromonas hydrophila in Geoclemys hamiltonii and Trachemys scripta: A Comparative Transcriptomic Perspective
Source: Genes (Basel). 2026 Apr 9;17(4):436. doi: 10.3390/genes17040436 (PMC13116093; doi:10.3390/genes17040436)
Supplement: Supplementary file 1 [file genes-17-00436-s001.zip › Figure S2/CARD8.pdf]

PREDICTED: Trachemys scripta elegans caspase recruitment domain-containing protein 8-like (LOC117876608), partial mRNA

Sequence ID: XM_034768860.1 Length: 1137 Number of Matches: 1

Range 1: 26 to 959 GenBank Graphics ▼ Next Match ▲ Previous Match

Score	Expect	Identities	Gaps	Strand
1162 bits(629)	0.0	834/935(89%)	5/935(0%)	Plus/Plus

```
Query  1193  GAGTGAGACGCAGAAGTGAAGATGCCACTTCAGACTCCTGGGGAAAGGACAGATGTGACT  1252
Sbjct  26    GAGTGAGACACAGGAGTGAAGATGCCACTTCAGACTCCTGGGGAAAGGACAGATGTGACT  85

Query  1253  TGTGTCGCAGAGCGAAGGGTTTTGCAGAAGTACAACCAGAGATTTTGCCAGGCC---TGG  1309
Sbjct  86    TGTGTCACAGAGAGAAGGGTTTTGCAGAAGTACAACCAGAGATTTTGCCAGGCCCAGAGG  145

Query  1310  AGAACCAGAAAACGTACAGGGTTCACTTCCCCCAGGCAGGCTCCTTCAGATGCTCTGAAA  1369
Sbjct  146   AGAACCAGAAAACGTACAGGGTTCACTTCCCCCAGGCAGGCTCCTTCAGATGCTCTGAAA  205

Query  1370  CTGAACTGGGGTTCGAGGTGAGGGCAGCCGTGACTCTCAAATACGAATATGAATCCTGGC  1429
Sbjct  206   CTGAACTGGGGTTCGAAGTCAGGGCAGCCGTGACTCTCAAATACAACTATGAATCCTGGC  265

Query  1430  GTTGTCATCAGACTGAACTGGAAAGGCAGCAGTGGATGATCGCCGGCCCTTTGTTCAACA  1489
Sbjct  266   GTTGTTGTCAGACAGAACTGGACATGCAGCAGTGGATGATCGCTGGCCCTTTGTTCAACA  325

Query  1490  TCTGGGCGGAACCAGCTGGGGCTGTGGCAGCTGTGCACCTCCCCCACTTCATGTGCCTGG  1549
Sbjct  326   TCTGGGCGGAACCGGCCGGGGCTGTGGCAGCTCTGCACCTCCCCCACTTCATGTGCCTCA  385

Query  1550  CAGGGGGAGAGGCTGACAGCTCCCAGATGCGAATCGCCCATTTCGTTGACGGGAGGATGA  1609
Sbjct  386   CAGGGGGAGAGGCTGGCGTCTCCCAGATGCGAATGGCCCATTTTGTTGATGGGAGGCTGA  445

Query  1610  CGCTGGAGGAGCCAATGAGAGTGATGCCTTTTCATGCAGTGCTGGAGAACCCCCGGTTCT  1669
Sbjct  446   CACTGGAGGAGCCAACGCGAGTGATGCCTTTCCACGCTGTGCTGGAGAACCCCCGTTTCT  505

Query  1670  CCCTTTGGGGAGTTATTTGGAAAC-GGAAAGAGTCTAAGAAAGATCCTCCACGCCACAGT  1728
Sbjct  506   CCTTTTGGGGAACTGTTTTGAAAAAAGGAATA-TCTATGCTATCTAGTCCAATCCACGGT  564

Query  1729  GTCACATTGCTCTACCGGGCTCTCGGGGCTCAACATATAACTCTCCACCTCTACCTGATA  1788
Sbjct  565   ATCACACTGCTTTACCGGGCACTCAGGGCTGAAAATATAACTCTCCACCTCTACTTGATA  624

Query  1789  CCTGACATCGTTGCGTTGAAAACGGTCATTGATGACAATGAAAGAAAGGACAAATCGATA  1848
Sbjct  625   CCTGACATCCTTCCATTGAGAAAGGTAATTGATGACAATGAAAGAAAGTACAAATCAATA  684

Query  1849  CGTGTGCGCAAACCTCCTACAACCCAGTCATTGTCCTATGGCTCCCATTATGCAGTATCC  1908
Sbjct  685   CGTGTGCGCAAACCTCCTACAACTAAACCATTGACCTATGGCTCCCATTATGCTGTATCT  744

Query  1909  AGCACATCAGATGTAGAGATAACACCTAACGAGTTGGAGTTCTGTTATGTAGATCCCCAG  1968
Sbjct  745   AGCACATCAGATGTAGAGATAACACCTGAGGAGCTGGAGTTCTGTTATGTAGATCCCCAG  804

Query  1969  GATGAACATCCATACACGGAGATTTACACCCAGAGCTTGGTGGATAGACTGGAGCTCAAC  2028
Sbjct  805   GATGAACATCCATACATGGAGATTTACACCCAGGACTTGGTGGACAAACTGGAGCTCCAC  864

Query  2029  GTAGTGAAGCAAAGCGATAGGCAAGTGATCTGGAAAGCCACTGTGAGACCAGGTGACATT  2088
Sbjct  865   TTGGTGAAGCAAAGTGATAGGCAACTGATCTGGAAGGCCCTTGTGAGACCAGGTGACATT  924

Query  2089  GAGCACAGTTCACCATCAGCAGAACGCCACACAGG  2123
Sbjct  925   ACGCACAGTTCACCATCAACAGAATGCCACACAGG  959
```

Related Information

Gene - associated gene det

Genome Data Viewer - align

genomic context
