# Supplementary material for: Divergent IL18-STAT1 Immune Responses Underlie Differential Susceptibility to Aeromonas hydrophila in Geoclemys hamiltonii and Trachemys scripta: A Comparative Transcriptomic Perspective
Source: Genes (Basel). 2026 Apr 9;17(4):436. doi: 10.3390/genes17040436 (PMC13116093; doi:10.3390/genes17040436)
Supplement: Supplementary file 1 [file genes-17-00436-s001.zip › Figure S2/CASP1.pdf]

PREDICTED: Trachemys scripta elegans caspase-1-like (LOC117867574), mRNA

Sequence ID: XM_034752746.1 **Length:** 4890 **Number of Matches:** 2

Range 1: 491 to 1254 GenBank Graphics ▼ Next Match ▲ Previous Match

Score	Expect	Identities	Gaps	Strand
1112 bits(602)	0.0	710/764(93%)	0/764(0%)	Plus/Plus

```
Query  1886  AGATCTATTCCATCCGGGACAAGGCGACGCGAACCCGGCTGGCCCTCATCATCTGTAATG  1945
Sbjct  491   AGATCTATTCCATCCTGGACAAGGCGACGCGAACCCGCCTGGCCCTCATCATCTGTAATG  550

Query  1946  TGGAGTTTGAGCATCTCCGCAGGAGGGATGGGGCTGACGTGGATGTGAAAGGGATGGAGA  2005
Sbjct  551   TTGAGTTTGAGCATCTCCGCAGGAGGGACGGGGCTGACGTGGACATGAATGGGATGAAGA  610

Query  2006  AGCTTCTAGAAGGGCTGGGATACAAAGTGGAAACCCACTGCAACTTAAACGCCCAGGCCA  2065
Sbjct  611   GGCTTCTAGAAGGGCTGGGATACAAGGTGGAAATTCACTGCAACTTGAATGCCCAGGCCA  670

Query  2066  TGGCGGAGACCCTGAAGCAGTTTGCTGCTCGGAACGAGCACCAGACCTCGGACAGCACCT  2125
Sbjct  671   TGGTGGAGACTCTGAAGCAGTTCGCTGCTCGGAATGAGCACCGGACCTCGGATAGCACCT  730

Query  2126  TCCTCGTGCTCATGTCTCACGGCGTCAGGGCTGGCCTGTGTGGGACAAATTGCCAAGACG  2185
Sbjct  731   TCCTCGTGCTCATGTCTCATGGCGTCAGGGCTGGCCTGTGCGGGACAAAGAGCCAAGGCG  790

Query  2186  AGTCCACAGACATCCTTCCTATTGACACCATCTACAGCACCTTCAACAACAAGAACTGCC  2245
Sbjct  791   AGTCCACAGACATCCTTCCCATTGACACCATCTACAGCACCTTCAACAACAAGAATTGCC  850

Query  2246  AGGCGCTGCTGGGAAAACCCAAAGTGATCATCATTCAGGCCTGCCGTGGGGAGAACCGCG  2305
Sbjct  851   AGACACTGCTGGGAAAACCCAAAGTGATCATCATTCAGGCCTGCCGTGGGGAGAACCGCG  910

Query  2306  GACAGGTGTGGGTGAGTGACTCTGCAGAGCTCCCTGGAGACGGCTCCAGCCTTGCTCCGC  2365
Sbjct  911   GACAGGTGTTGGTGAGTGACTCTGCAGAGCTCCCTGGAGACGGCTCCAGCCTCGCTCCGC  970

Query  2366  TGCCTCCTCAAGGATTAGAAGATGATGCAAGTCGCCGAATCCACGTGGAGACCGATTTCA  2425
Sbjct  971   TGCCCCCTGAAGAGTTAGAAGATGATGCAAGTCACCAAATCCACGTGGAGAGCGATTTCA  1030

Query  2426  TCTGTATGCATGCTACAACGCCAGACACTTTGTCCTGGAGATCTCCAAAAACTGGCTCCG  2485
Sbjct  1031  TCGGTTTCCACGCTACAACGCCAGACACTTTGTCCTGGAGATGTCCAAAAACTGGGTCCG  1090

Query  2486  TCTTCATCACCCGTCTGATAGAGAAGCTCCGAACCAATGCCTGCCGCTTCCCCTTGGAGG  2545
Sbjct  1091  TCTTCATCACCCGTCTGATAGAGCAGCTTCGAACCAATGCCTGCCGCTTCCCCTTGGAGG  1150

Query  2546  AGATCTTCCGACAGGTCCAGTTCTCCTTTCAAGATTTTCCTGATCAGATGCCCACCAAGG  2605
Sbjct  1151  AGATCTTCCGAAAGGTCCAGCTCTCCTTTCAAAATTTTCCTCGTCAGATGCCCACCAAGG  1210

Query  2606  AAAGAACTACCATGATAAAAAAGTTCTACCTGTTCCCAGGTCAT  2649
Sbjct  1211  AAAGAACCACCATGATAAAAAAGTTCTATCTGTTCCCAGGCCAT  1254
```

Range 2: 121 to 492 GenBank Graphics ▼ Next Match ▲ Previous Match First Match

Score	Expect	Identities	Gaps	Strand
599 bits(324)	2e-169	356/372(96%)	0/372(0%)	Plus/Plus

```
Query  7    GATCGGCAGTTAAGCGACGTGCGGATCAGGTTTGTGGAGAGCGTGAGCAAGGCCGTGATC  66
Sbjct  121  GATCGGAAGTTAAGCGACGTGCGGATCAGGTTTGTGGAGCGCGTGAGCAAGGCAGTGATC  180

Query  67   AGCACCCTCCTGGACGACCTGCTGGAGAGACGGGTGCTGAACGAGGAGGAAGTGGAGGAG  126
Sbjct  181  AGCAATCTCCTGGACGACCTGCTGGAGAGACGGGTGCTGAACGAGGAGGAAGTGGAGGAG  240

Query  127  GTGAAGGAGAGCTACAGTAAGAAGAGTGACCAAGCCAGGTGTCTGATTGATGGGGTGAGG  186
Sbjct  241  GTGAGGGAGAGCTACAGTAAGAAGAGTGACCAAGCCAGGTGCCTGATTGATGGGGTGAGG  300

Query  187  AAGAAGGGTGCCAAAGCCAGCGAAATATTCATCGAGCGCCTCCGCGTCAGAGATGTCCAC  246
Sbjct  301  AAAAAGGGTGCCAGAGCCAGCGAAATATTCATCGAGCGCCTCTGCGTCAGAGATGTCCAC  360

Query  247  CTGGCCACGGAGCTGGGGCTCGGCGCCCCCTCGGGGTCTGCTGCAGAAACCCAGCAAGTG  306
Sbjct  361  CTGGCCACAGAGCTGGGGCTCGGAGCCCCCTCAGGGACTGCTGCAGAAACCCAGCCAGCG  420

Query  307  CCATCTCAGGAATGGATCCAGCCCTGCCCCCGGGAATTCGTCCAGCGGATCCAGAAGGAG  366
Sbjct  421  CCATCTCAGGAATGGATCCAGCCCTGCCCCCGGGAATTCGTCCAGCGGATCCAGAAGGAG  480

Query  367  GAAGCAAAGGAG  378
Sbjct  481  GAAGCAAAGGAG  492
```
