# Supplementary material for: Divergent IL18-STAT1 Immune Responses Underlie Differential Susceptibility to Aeromonas hydrophila in Geoclemys hamiltonii and Trachemys scripta: A Comparative Transcriptomic Perspective
Source: Genes (Basel). 2026 Apr 9;17(4):436. doi: 10.3390/genes17040436 (PMC13116093; doi:10.3390/genes17040436)
Supplement: Supplementary file 1 [file genes-17-00436-s001.zip › Figure S2/CCL4.pdf]

PREDICTED: Trachemys scripta elegans C-C motif chemokine 4-like (LOC117867492), mRNA

Sequence ID: XM_034752586.1 **Length:** 963 **Number of Matches:** 1

Range 1: 303 to 574 GenBank Graphics ▼ Next Match ▲ Previous Match

Score	Expect	Identities	Gaps	Strand
420 bits(227)	3e-116	257/272(94%)	0/272(0%)	Plus/Plus

```
Query  1    ATGAAGGTCTCCATGGCTGCCCTCGCCGTTCTCCTCGTCGCTGCCTTCTGCTCCCTGGGC  60
            |||||||||||| ||||||||||||||||||||||| |||||||||||||||| | || |
Sbjct  303  ATGAAGGTCTCCGTGGCTGCCCTCGCCGTTCTCCTCATCGCTGCCTTCTGCTCGCAGGCC  362

Query  61   TCCTCTGCGCCAATTGGCTCCGATCCCCCGACTGCCTGCTGCTTTACCTACACATCTCGG  120
            |||||||| ||||||||||||||||||||||||||||||||||||| ||||||||| |||
Sbjct  363  TCCTCTGCCCCAATTGGCTCCGATCCCCCGACTGCCTGCTGCTTTAGCTACACATCCCGG  422

Query  121  AAGATCCCACGCAGCTTGGTGGTAGATTATTACGACACCAACAGCATGTGCTCCCAGACG  180
            |||||||||||| |||||||||| |||||||| |||||||||||||||||||||||||||
Sbjct  423  AAGATCCCACGCGGCTTGGTGGTGGATTATTATGACACCAACAGCATGTGCTCCCAGACG  482

Query  181  GCCATAGTATTTATCACCAAGAAGGGCCGTGAAGTCTGTGCTAACCCCAAAGAGGACTGG  240
            ||||||||||||||||||||||||||||||||||||||||||||||||||||||||||||
Sbjct  483  GCCATAGTATTTATCACCAAGAAGGGCCGTGAAGTCTGTGCTAACCCCAAAGAGGACTGG  542

Query  241  GTTCAGGAGTACATGACTCAGCTGGAACTGAA  272
            |||||||||||| |||| || |||||| ||||
Sbjct  543  GTTCAGGAGTACGTGACCCATCTGGAAATGAA  574
```
