# Supplementary material for: Divergent IL18-STAT1 Immune Responses Underlie Differential Susceptibility to Aeromonas hydrophila in Geoclemys hamiltonii and Trachemys scripta: A Comparative Transcriptomic Perspective
Source: Genes (Basel). 2026 Apr 9;17(4):436. doi: 10.3390/genes17040436 (PMC13116093; doi:10.3390/genes17040436)
Supplement: Supplementary file 1 [file genes-17-00436-s001.zip › Figure S2/CCR10.pdf]

**PREDICTED: Trachemys scripta elegans C-C motif chemokine receptor 10 (CCR10), mRNA**

Sequence ID: XM_034756282.1 Length: 1441 Number of Matches: 1

Range 1: 1 to 1441 GenBank Graphics ▼ Next Match ▲ Previous Match

| Score | Expect | Identities | Gaps | Strand |
|---|---|---|---|---|
| 2662 bits(1441) | 0.0 | 1441/1441(100%) | 0/1441(0%) | Plus/Plus |

```
Query  1     ATGGACCCTGAGAGCGGAGGCCGGGGAGCAGGGGTGGAGCCGGGGGCCAGGACCCGAGTC  60
             ||||||||||||||||||||||||||||||||||||||||||||||||||||||||||||
Sbjct  1     ATGGACCCTGAGAGCGGAGGCCGGGGAGCAGGGGTGGAGCCGGGGGCCAGGACCCGAGTC  60

Query  61    CCAGTGTTCATATACTGTGCTGTATTGCActgctgtgttgtgttaatatcttgttgcaat  120
             ||||||||||||||||||||||||||||||||||||||||||||||||||||||||||||
Sbjct  61    CCAGTGTTCATATACTGTGCTGTATTGCACTGCTGTGTTGTGTTAATATCTTGTTGCAAT  120

Query  121   gctgtgttgtgtagctgcggtgttgtgttgctgtgtagctatattgtgttgctgtgtggt  180
             ||||||||||||||||||||||||||||||||||||||||||||||||||||||||||||
Sbjct  121   GCTGTGTTGTGTAGCTGCGGTGTTGTGTTGCTGTGTAGCTATATTGTGTTGCTGTGTGGT  180

Query  181   gtaactgtggtgctgcgttgtgttgtgtgctCGAAGCTCCTTGCTAATGATAACGATGAT  240
             ||||||||||||||||||||||||||||||||||||||||||||||||||||||||||||
Sbjct  181   GTAACTGTGGTGCTGCGTTGTGTTGTGTGCTCGAAGCTCCTTGCTAATGATAACGATGAT  240

Query  241   GTTCCGCCCTCACAGACTTTCAGCCCCTGGGCGGCCGAGCCCCCGGTCACCACCGACTAC  300
             ||||||||||||||||||||||||||||||||||||||||||||||||||||||||||||
Sbjct  241   GTTCCGCCCTCACAGACTTTCAGCCCCTGGGCGGCCGAGCCCCCGGTCACCACCGACTAC  300

Query  301   TACGACGAGGATGAGCAGTACCCGGAGTGGGTCAGCTCCCTGCCCGAGCTGTGTGAGAAG  360
             ||||||||||||||||||||||||||||||||||||||||||||||||||||||||||||
Sbjct  301   TACGACGAGGATGAGCAGTACCCGGAGTGGGTCAGCTCCCTGCCCGAGCTGTGTGAGAAG  360

Query  361   GGGTCTGTGCAGAGCTTTGCCCGCACCTACCAGACGGTTGTCTACCTGCTGCTGTCAGCG  420
             ||||||||||||||||||||||||||||||||||||||||||||||||||||||||||||
Sbjct  361   GGGTCTGTGCAGAGCTTTGCCCGCACCTACCAGACGGTTGTCTACCTGCTGCTGTCAGCG  420

Query  421   CTGGGCGTGGCGGGGAACGGACTGGTGCTGCTCATCCACGCCCGCTACCGCCAGGCCCAC  480
             ||||||||||||||||||||||||||||||||||||||||||||||||||||||||||||
Sbjct  421   CTGGGCGTGGCGGGGAACGGACTGGTGCTGCTCATCCACGCCCGCTACCGCCAGGCCCAC  480

Query  481   TCCGTGACCGACGTctgcctgctgcacctggccatggccgacctgctgctgctgctgacg  540
             ||||||||||||||||||||||||||||||||||||||||||||||||||||||||||||
Sbjct  481   TCCGTGACCGACGTCTGCCTGCTGCACCTGGCCATGGCCGACCTGCTGCTGCTGCTGACG  540

Query  541   ctgccctttgccgtggcgggcgcgctgcagggctggctgctgggcACCGACGCCTGCCGG  600
             ||||||||||||||||||||||||||||||||||||||||||||||||||||||||||||
Sbjct  541   CTGCCCTTTGCCGTGGCGGGCGCGCTGCAGGGCTGGCTGCTGGGCACCGACGCCTGCCGG  600

Query  601   ACCATGCAGGGCTTCTACGCCCTCAACTTCTACAGCGGCTTCCTCTTCCTGACCTGCATC  660
             ||||||||||||||||||||||||||||||||||||||||||||||||||||||||||||
Sbjct  601   ACCATGCAGGGCTTCTACGCCCTCAACTTCTACAGCGGCTTCCTCTTCCTGACCTGCATC  660

Query  661   AGCGTGGACCGCTACATGGCCATCGTCCGGGCGCCTGCTGCCTACCGCCTGCACCCCCGG  720
             ||||||||||||||||||||||||||||||||||||||||||||||||||||||||||||
Sbjct  661   AGCGTGGACCGCTACATGGCCATCGTCCGGGCGCCTGCTGCCTACCGCCTGCACCCCCGG  720

Query  721   GCCCGCTGCTACGGCTGGCTCGCCGCCGGGCTGGCCTGGCTGCTCTCCACCCTGCTGGCA  780
             ||||||||||||||||||||||||||||||||||||||||||||||||||||||||||||
Sbjct  721   GCCCGCTGCTACGGCTGGCTCGCCGCCGGGCTGGCCTGGCTGCTCTCCACCCTGCTGGCA  780

Query  781   CTGCCCCAGTTCGTGTACAGCCGGGCCGAGGGTCACCAGGAACACCTGCTCTGCATGGTG  840
             ||||||||||||||||||||||||||||||||||||||||||||||||||||||||||||
Sbjct  781   CTGCCCCAGTTCGTGTACAGCCGGGCCGAGGGTCACCAGGAACACCTGCTCTGCATGGTG  840

Query  841   CTCTTCCCTGCCGGCGTTTCCAAGGCAGCCAAGGGAGCCACCAACCTGGCCCAGGTCATC  900
             ||||||||||||||||||||||||||||||||||||||||||||||||||||||||||||
Sbjct  841   CTCTTCCCTGCCGGCGTTTCCAAGGCAGCCAAGGGAGCCACCAACCTGGCCCAGGTCATC  900

Query  901   CTGGGCTTCGTGCTGCCCTTCCTGGTCATGGCCTCCTGCTACACGGCCATGGCCCGCACC  960
             ||||||||||||||||||||||||||||||||||||||||||||||||||||||||||||
Sbjct  901   CTGGGCTTCGTGCTGCCCTTCCTGGTCATGGCCTCCTGCTACACGGCCATGGCCCGCACC  960

Query  961   CTGCTGGCTGCCCGCAGCTTCCAGCGGCACAAGGCCCTACGCCTCATCCTGGCCCTGGTG  1020
             ||||||||||||||||||||||||||||||||||||||||||||||||||||||||||||
Sbjct  961   CTGCTGGCTGCCCGCAGCTTCCAGCGGCACAAGGCCCTACGCCTCATCCTGGCCCTGGTG  1020

Query  1021  CTTTTCTTCATGGCCCTCGAGCTTCCCCACAGCCTGATGGTGCTGCTGGACACGGCCGAC  1080
             ||||||||||||||||||||||||||||||||||||||||||||||||||||||||||||
Sbjct  1021  CTTTTCTTCATGGCCCTCGAGCTTCCCCACAGCCTGATGGTGCTGCTGGACACGGCCGAC  1080

Query  1081  ATCCTGGGCAGCCGGGAGATGAGCTGCGCCCAGAGCCGCCGCAAGGACCTGGCTCTGGTG  1140
             ||||||||||||||||||||||||||||||||||||||||||||||||||||||||||||
Sbjct  1081  ATCCTGGGCAGCCGGGAGATGAGCTGCGCCCAGAGCCGCCGCAAGGACCTGGCTCTGGTG  1140

Query  1141  GTGATCAGCGGCCTGGCCTTCGCCCGCTGCTGCCTCAACCCCGTGCTCTACGCCTTCATG  1200
             ||||||||||||||||||||||||||||||||||||||||||||||||||||||||||||
Sbjct  1141  GTGATCAGCGGCCTGGCCTTCGCCCGCTGCTGCCTCAACCCCGTGCTCTACGCCTTCATG  1200

Query  1201  GGCGTGCGCTTCCGGAAGGAGCTGCGGCTCCTGGCCAGCGATGTTGGCTGCGTGGGCCGG  1260
             ||||||||||||||||||||||||||||||||||||||||||||||||||||||||||||
Sbjct  1201  GGCGTGCGCTTCCGGAAGGAGCTGCGGCTCCTGGCCAGCGATGTTGGCTGCGTGGGCCGG  1260

Query  1261  GCACAGGATGGGCAGCACCCCAGCCCCAGGTGCCGGTCACAACTCTCCACCTGCCTGGAC  1320
             ||||||||||||||||||||||||||||||||||||||||||||||||||||||||||||
Sbjct  1261  GCACAGGATGGGCAGCACCCCAGCCCCAGGTGCCGGTCACAACTCTCCACCTGCCTGGAC  1320

Query  1321  ATGGTGTAGGAGGCGGCGCTGACCGGGCTCAGCCCTCTCCCTGCCAGCCAGGAGGCCAGG  1380
             ||||||||||||||||||||||||||||||||||||||||||||||||||||||||||||
Sbjct  1321  ATGGTGTAGGAGGCGGCGCTGACCGGGCTCAGCCCTCTCCCTGCCAGCCAGGAGGCCAGG  1380

Query  1381  GCTCCCCTCCCCGGCTGGCAGGGGGCTGCTGGGGCAGGCTCATGGGAGGAGGCAGTGTCA  1440
             ||||||||||||||||||||||||||||||||||||||||||||||||||||||||||||
Sbjct  1381  GCTCCCCTCCCCGGCTGGCAGGGGGCTGCTGGGGCAGGCTCATGGGAGGAGGCAGTGTCA  1440

Query  1441  T  1441
             |
Sbjct  1441  T  1441
```
