# Supplementary material for: Divergent IL18-STAT1 Immune Responses Underlie Differential Susceptibility to Aeromonas hydrophila in Geoclemys hamiltonii and Trachemys scripta: A Comparative Transcriptomic Perspective
Source: Genes (Basel). 2026 Apr 9;17(4):436. doi: 10.3390/genes17040436 (PMC13116093; doi:10.3390/genes17040436)
Supplement: Supplementary file 1 [file genes-17-00436-s001.zip › Figure S2/CD28.pdf]

PREDICTED: Trachemys scripta elegans CD28 molecule (CD28), mRNA

Sequence ID: XM_034786510.1 Length: 4437 Number of Matches: 1

Range 1: 1 to 4437 GenBank Graphics ▼ Next Match ▲ Previous Match

Score	Expect	Identities	Gaps	Strand
8194 bits(4437)	0.0	4437/4437(100%)	0/4437(0%)	Plus/Plus

```
Query  1     AAGgagaggagagagagaATGTGTGACTGACTGAACAGTCTAGCCCCACCAGGTTGGCTT  60
             ||||||||||||||||||||||||||||||||||||||||||||||||||||||||||||
Sbjct  1     AAGGAGAGGAGAGAGAGAATGTGTGACTGACTGAACAGTCTAGCCCCACCAGGTTGGCTT  60

Query  61    GTGGTTCAGTCTTCAGATTTCTTTGTGGGGGTGGCATCTACATTTGCAGCCCACCATTAT  120
             ||||||||||||||||||||||||||||||||||||||||||||||||||||||||||||
Sbjct  61    GTGGTTCAGTCTTCAGATTTCTTTGTGGGGGTGGCATCTACATTTGCAGCCCACCATTAT  120

Query  121   CATCATGATATTCTGGATACTCGCTGCTCTCAGCTTCATTCAAGCTGCTGAACTAACAGA  180
             ||||||||||||||||||||||||||||||||||||||||||||||||||||||||||||
Sbjct  121   CATCATGATATTCTGGATACTCGCTGCTCTCAGCTTCATTCAAGCTGCTGAACTAACAGA  180

Query  181   AAACAAGATTTTAGTGGAACAGCCACCTCGAATCACGGCATCTGATGAAAATGCTACCTT  240
             ||||||||||||||||||||||||||||||||||||||||||||||||||||||||||||
Sbjct  181   AAACAAGATTTTAGTGGAACAGCCACCTCGAATCACGGCATCTGATGAAAATGCTACCTT  240

Query  241   GGCTTGCAAATATGCCTATAATGGAACTGGGAATGAATTCAGAGCATCACTGAATAAAGG  300
             ||||||||||||||||||||||||||||||||||||||||||||||||||||||||||||
Sbjct  241   GGCTTGCAAATATGCCTATAATGGAACTGGGAATGAATTCAGAGCATCACTGAATAAAGG  300

Query  301   AGCAGACAGAGCAGTTGAAGTCTGCTCTGTTTCCTGGAATGGTTCCTTCCATAACAATCA  360
             ||||||||||||||||||||||||||||||||||||||||||||||||||||||||||||
Sbjct  301   AGCAGACAGAGCAGTTGAAGTCTGCTCTGTTTCCTGGAATGGTTCCTTCCATAACAATCA  360

Query  361   TTCAAATAAAGATTTCAACTGCCTTGTTGCAGTTGATAGCCATAAAAAGGAAGTGAATTT  420
             ||||||||||||||||||||||||||||||||||||||||||||||||||||||||||||
Sbjct  361   TTCAAATAAAGATTTCAACTGCCTTGTTGCAGTTGATAGCCATAAAAAGGAAGTGAATTT  420

Query  421   CAGTCTTTGGAAGCTGAATACTGACCAAACAGATATTTACTTCTGCAAAATTGAGGTCAT  480
             ||||||||||||||||||||||||||||||||||||||||||||||||||||||||||||
Sbjct  421   CAGTCTTTGGAAGCTGAATACTGACCAAACAGATATTTACTTCTGCAAAATTGAGGTCAT  480

Query  481   GTTTCCACCTCCTTACATCTCCAACGACAAGAGCAATGGGACTGTCATTCATGTGAAAGA  540
             ||||||||||||||||||||||||||||||||||||||||||||||||||||||||||||
Sbjct  481   GTTTCCACCTCCTTACATCTCCAACGACAAGAGCAATGGGACTGTCATTCATGTGAAAGA  540

Query  541   GATGGTCCGCCAACCACCACCATTTCAATCATCCCTTTGGGCTCTGATGGTGACTCTTGG  600
             ||||||||||||||||||||||||||||||||||||||||||||||||||||||||||||
Sbjct  541   GATGGTCCGCCAACCACCACCATTTCAATCATCCCTTTGGGCTCTGATGGTGACTCTTGG  600

Query  601   ATTTCTGGCTTTCTACAGTGTGCTAATAACTGTGGTGTTTGTTATCTGCTGGTGGAAAAA  660
             ||||||||||||||||||||||||||||||||||||||||||||||||||||||||||||
Sbjct  601   ATTTCTGGCTTTCTACAGTGTGCTAATAACTGTGGTGTTTGTTATCTGCTGGTGGAAAAA  660

Query  661   CAAGAAGAACAGAATCTTGACGAGTGACTACATGAACATGACGCCACGCCATCCTCCTGG  720
             ||||||||||||||||||||||||||||||||||||||||||||||||||||||||||||
Sbjct  661   CAAGAAGAACAGAATCTTGACGAGTGACTACATGAACATGACGCCACGCCATCCTCCTGG  720

Query  721   CCCAAAGAACAAGCATTACCAACCCTACGCACCAACTCGGATACACACGGAATACCGCTC  780
             ||||||||||||||||||||||||||||||||||||||||||||||||||||||||||||
Sbjct  721   CCCAAAGAACAAGCATTACCAACCCTACGCACCAACTCGGATACACACGGAATACCGCTC  780

Query  781   TTGGGAACCATGACTATCTGCTTACCAAGCTGCTAATACCCCTGGTGTGCTTTTACTCTG  840
             ||||||||||||||||||||||||||||||||||||||||||||||||||||||||||||
Sbjct  781   TTGGGAACCATGACTATCTGCTTACCAAGCTGCTAATACCCCTGGTGTGCTTTTACTCTG  840

Query  841   CCACGTGCACTTGGACGTGAAAGGACAGCCTGCCATTAGTTTGTCATATCTGTTATATTT  900
             ||||||||||||||||||||||||||||||||||||||||||||||||||||||||||||
Sbjct  841   CCACGTGCACTTGGACGTGAAAGGACAGCCTGCCATTAGTTTGTCATATCTGTTATATTT  900

Query  901   GATGACATCATAGCAAGTACCGGTCAAACTTTAATTGATGCTGTGTAAAAGTAGGTTGTT  960
             ||||||||||||||||||||||||||||||||||||||||||||||||||||||||||||
Sbjct  901   GATGACATCATAGCAAGTACCGGTCAAACTTTAATTGATGCTGTGTAAAAGTAGGTTGTT  960

Query  961   TTCTGTGATGGCTGAAAACTAGCTACGACTCATGTTCAAGTTTGATGGTGGGTAAGGGGG  1020
             ||||||||||||||||||||||||||||||||||||||||||||||||||||||||||||
Sbjct  961   TTCTGTGATGGCTGAAAACTAGCTACGACTCATGTTCAAGTTTGATGGTGGGTAAGGGGG  1020

Query  1021  AAAGATAACACTGGATCCAATACAGGGTATATTGTTCACTGTTGCAACTACCGATCTTTC  1080
             ||||||||||||||||||||||||||||||||||||||||||||||||||||||||||||
Sbjct  1021  AAAGATAACACTGGATCCAATACAGGGTATATTGTTCACTGTTGCAACTACCGATCTTTC  1080

Query  1081  CCAATCACTTTTGCCTTATATTGAATATTAATTTCTAGTTAGAAGTATGTTTAACTTGAA  1140
             ||||||||||||||||||||||||||||||||||||||||||||||||||||||||||||
Sbjct  1081  CCAATCACTTTTGCCTTATATTGAATATTAATTTCTAGTTAGAAGTATGTTTAACTTGAA  1140

Query  1141  GAATTATAAATCTCTGAAATACAGCCCTAATTGGTTACCATTAAATATGGTACCAAGCAA  1200
             ||||||||||||||||||||||||||||||||||||||||||||||||||||||||||||
Sbjct  1141  GAATTATAAATCTCTGAAATACAGCCCTAATTGGTTACCATTAAATATGGTACCAAGCAA  1200

Query  1201  ACTGTTGATGCAAAAATTTGTCCCCTCTCACTTCAAAGAGGAGAACCCAGTTTCAAGTTC  1260
             ||||||||||||||||||||||||||||||||||||||||||||||||||||||||||||
Sbjct  1201  ACTGTTGATGCAAAAATTTGTCCCCTCTCACTTCAAAGAGGAGAACCCAGTTTCAAGTTC  1260

Query  1261  CAAACATATGCTTAAAAGAATAGAAGAGGGTTTCAGCTGTAGGGAAAAGTCAGCAGTGAG  1320
             ||||||||||||||||||||||||||||||||||||||||||||||||||||||||||||
Sbjct  1261  CAAACATATGCTTAAAAGAATAGAAGAGGGTTTCAGCTGTAGGGAAAAGTCAGCAGTGAG  1320

Query  1321  CCATGTGGACTATTAGATTATTCCACAGATGGGCTGTAATGACAGATCCAGGACACTCAA  1380
             ||||||||||||||||||||||||||||||||||||||||||||||||||||||||||||
Sbjct  1321  CCATGTGGACTATTAGATTATTCCACAGATGGGCTGTAATGACAGATCCAGGACACTCAA  1380

Query  1381  AATCCACTATACATTAAATGTGCGATTCTTAAGTTCTTAAATTGGTAACTGTGTAATATG  1440
             ||||||||||||||||||||||||||||||||||||||||||||||||||||||||||||
Sbjct  1381  AATCCACTATACATTAAATGTGCGATTCTTAAGTTCTTAAATTGGTAACTGTGTAATATG  1440

Query  1441  TGTAGGGGGAGAGGACCCCCCTATGAAACAATTTCATACAGTGAGAAAGCAATCAATCTG  1500
             ||||||||||||||||||||||||||||||||||||||||||||||||||||||||||||
Sbjct  1441  TGTAGGGGGAGAGGACCCCCCTATGAAACAATTTCATACAGTGAGAAAGCAATCAATCTG  1500

Query  1501  CAAGGCAGGTCTGGAACAACAGGGGATGAAGAAAGATCTGCAGTCATCACGAAGAGAATC  1560
             ||||||||||||||||||||||||||||||||||||||||||||||||||||||||||||
Sbjct  1501  CAAGGCAGGTCTGGAACAACAGGGGATGAAGAAAGATCTGCAGTCATCACGAAGAGAATC  1560

Query  1561  CTTTTGAATTATGTTGGCCTACAACAAGGAAACACCAGGACTTGGCCAAAGTCATAGGAG  1620
             ||||||||||||||||||||||||||||||||||||||||||||||||||||||||||||
Sbjct  1561  CTTTTGAATTATGTTGGCCTACAACAAGGAAACACCAGGACTTGGCCAAAGTCATAGGAG  1620

Query  1621  AAGCAGGTGAGAAAATCCTCATTAGAGCTTATTGAGGGCAGATTTTCAAAAGTGGGCCAG  1680
             ||||||||||||||||||||||||||||||||||||||||||||||||||||||||||||
Sbjct  1621  AAGCAGGTGAGAAAATCCTCATTAGAGCTTATTGAGGGCAGATTTTCAAAAGTGGGCCAG  1680

Query  1681  ATTTTTGAGTGCTCAGCACACACAGTCGGGGCCAGATTTTTAGCTGCGCTGAGAACCCAG  1740
             ||||||||||||||||||||||||||||||||||||||||||||||||||||||||||||
Sbjct  1681  ATTTTTGAGTGCTCAGCACACACAGTCGGGGCCAGATTTTTAGCTGCGCTGAGAACCCAG  1740

Query  1741  TGTGCATACAGCCTGCTGAGCTCTCCTGAAAATCTGTCCATGTAGTCTGGTGCTTTTTTC  1800
             ||||||||||||||||||||||||||||||||||||||||||||||||||||||||||||
Sbjct  1741  TGTGCATACAGCCTGCTGAGCTCTCCTGAAAATCTGTCCATGTAGTCTGGTGCTTTTTTC  1800

Query  1801  AAAATCTGGTCACAGTGTTGAGTGATCAGATCATTTGAAAATTTGCTCCTAAACACCCTA  1860
             ||||||||||||||||||||||||||||||||||||||||||||||||||||||||||||
Sbjct  1801  AAAATCTGGTCACAGTGTTGAGTGATCAGATCATTTGAAAATTTGCTCCTAAACACCCTA  1860

Query  1861  TCATTGGAGAGGCTGAGTGACCTCAACTCCCATTGCAGTCAAGAGGAACTGAAAACTCTC  1920
             ||||||||||||||||||||||||||||||||||||||||||||||||||||||||||||
Sbjct  1861  TCATTGGAGAGGCTGAGTGACCTCAACTCCCATTGCAGTCAAGAGGAACTGAAAACTCTC  1920

Query  1921  AGCACTTTGCAGGCGATGTTCAGTACCTTGAAGGATCAGGCTCTAACTGGGGCTTTCTGT  1980
             ||||||||||||||||||||||||||||||||||||||||||||||||||||||||||||
Sbjct  1921  AGCACTTTGCAGGCGATGTTCAGTACCTTGAAGGATCAGGCTCTAACTGGGGCTTTCTGT  1980

Query  1981  TCTTATATAGACTTTTCTCTAGGTATGTTCCATGGAGGAGTAAAACAAGGGCTAGAATAA  2040
             ||||||||||||||||||||||||||||||||||||||||||||||||||||||||||||
Sbjct  1981  TCTTATATAGACTTTTCTCTAGGTATGTTCCATGGAGGAGTAAAACAAGGGCTAGAATAA  2040

Query  2041  AACTCGTTCAGTGATTTTCATGTGGCATGTTTCTTTTAATGATGATTCCAAAACTGCAGT  2100
             ||||||||||||||||||||||||||||||||||||||||||||||||||||||||||||
Sbjct  2041  AACTCGTTCAGTGATTTTCATGTGGCATGTTTCTTTTAATGATGATTCCAAAACTGCAGT  2100

Query  2101  AGGGTCAGCTCAACACAAAAGCTATTCCCAAGCTTAGGAGAAGAGGTTCCTTTATCGGTT  2160
             ||||||||||||||||||||||||||||||||||||||||||||||||||||||||||||
Sbjct  2101  AGGGTCAGCTCAACACAAAAGCTATTCCCAAGCTTAGGAGAAGAGGTTCCTTTATCGGTT  2160

Query  2161  CCCTCCAATCCTTCTAGAGCTGAGCAAAAGACAGAATTTCTGTCCGTGCGAAAATTCTGA  2220
             ||||||||||||||||||||||||||||||||||||||||||||||||||||||||||||
Sbjct  2161  CCCTCCAATCCTTCTAGAGCTGAGCAAAAGACAGAATTTCTGTCCGTGCGAAAATTCTGA  2220

Query  2221  TAATTCTACATTTGTTTTTATCTTGAACCAGAACAAAAAGATCCCTGGCTTCGAACTTCC  2280
             ||||||||||||||||||||||||||||||||||||||||||||||||||||||||||||
Sbjct  2221  TAATTCTACATTTGTTTTTATCTTGAACCAGAACAAAAAGATCCCTGGCTTCGAACTTCC  2280

Query  2281  ACGAAGCTTATGGGCAACCCTAAGCCACCTCCACACAATCCATGGCAGATGCGGATACTT  2340
             ||||||||||||||||||||||||||||||||||||||||||||||||||||||||||||
Sbjct  2281  ACGAAGCTTATGGGCAACCCTAAGCCACCTCCACACAATCCATGGCAGATGCGGATACTT  2340

Query  2341  GATGCACAAATGGAACATTAAAAACTCTCCAGTGTGCGACTGTGGTCACCCAGAACAGAC  2400
             ||||||||||||||||||||||||||||||||||||||||||||||||||||||||||||
Sbjct  2341  GATGCACAAATGGAACATTAAAAACTCTCCAGTGTGCGACTGTGGTCACCCAGAACAGAC  2400

Query  2401  CATGGAATATATAACAACTCAATGTCTGATTCAGAAATACAAAGGAGGCATCACAGCAAT  2460
             ||||||||||||||||||||||||||||||||||||||||||||||||||||||||||||
Sbjct  2401  CATGGAATATATAACAACTCAATGTCTGATTCAGAAATACAAAGGAGGCATCACAGCAAT  2460

Query  2461  ATACCCCGCCACTCTTGATCCAATTACCTGACAACCACCTAAATGTAAAATTATAGTTGT  2520
             ||||||||||||||||||||||||||||||||||||||||||||||||||||||||||||
Sbjct  2461  ATACCCCGCCACTCTTGATCCAATTACCTGACAACCACCTAAATGTAAAATTATAGTTGT  2520

Query  2521  TGCTCTATAGCTACACACCAGCCATATAAGAAGGaaaaaaaaCTGAAATATCAAATTTTT  2580
             ||||||||||||||||||||||||||||||||||||||||||||||||||||||||||||
Sbjct  2521  TGCTCTATAGCTACACACCAGCCATATAAGAAGGAAAAAAAACTGAAATATCAAATTTTT  2580

Query  2581  TCACAGAACAGAAATTCCaaaaaaaTTTGATTCAGAAATGGAGAAACAGTCCAAGTCAAC  2640
             ||||||||||||||||||||||||||||||||||||||||||||||||||||||||||||
Sbjct  2581  TCACAGAACAGAAATTCCAAAAAAATTTGATTCAGAAATGGAGAAACAGTCCAAGTCAAC  2640

Query  2641  ATTTTCAATAGAAATGCAATGTTTTGACATTCCTGACTGAAAATGTTTCATTTAATTTTG  2700
             ||||||||||||||||||||||||||||||||||||||||||||||||||||||||||||
Sbjct  2641  ATTTTCAATAGAAATGCAATGTTTTGACATTCCTGACTGAAAATGTTTCATTTAATTTTG  2700

Query  2701  TTGATCTGACTGGAAATGCATAGTTTTAAATCAGTTCAGCATTAAACTGCATTTCCTTAC  2760
             ||||||||||||||||||||||||||||||||||||||||||||||||||||||||||||
Sbjct  2701  TTGATCTGACTGGAAATGCATAGTTTTAAATCAGTTCAGCATTAAACTGCATTTCCTTAC  2760

Query  2761  AGAGCCACATTGCCTCATTGGAATTCAGTTCAGGAGTCTGATGCCCTTGTTCTCAACTAT  2820
             ||||||||||||||||||||||||||||||||||||||||||||||||||||||||||||
Sbjct  2761  AGAGCCACATTGCCTCATTGGAATTCAGTTCAGGAGTCTGATGCCCTTGTTCTCAACTAT  2820

Query  2821  GGACCAGGCACCCTGGCTGGACTATATCTCCCATGATACAACAGCAGTAATGTGACATGA  2880
             ||||||||||||||||||||||||||||||||||||||||||||||||||||||||||||
Sbjct  2821  GGACCAGGCACCCTGGCTGGACTATATCTCCCATGATACAACAGCAGTAATGTGACATGA  2880

Query  2881  CAGACTACTCAGCTTGGTCAGAGAGGAGACAGTGTTGCATCATGGGAAATATAGTCAGGC  2940
             ||||||||||||||||||||||||||||||||||||||||||||||||||||||||||||
Sbjct  2881  CAGACTACTCAGCTTGGTCAGAGAGGAGACAGTGTTGCATCATGGGAAATATAGTCAGGC  2940

Query  2941  CAGGGAGTCCAACCCATGCGGGGGAGGTCGGGGGCTGGAGGCAAACGCTGGGTCCCATAA  3000
             ||||||||||||||||||||||||||||||||||||||||||||||||||||||||||||
Sbjct  2941  CAGGGAGTCCAACCCATGCGGGGGAGGTCGGGGGCTGGAGGCAAACGCTGGGTCCCATAA  3000

Query  3001  GCCAATGCAGCAAAACAGGGAAATGCAGTTTAATGTTGAACTGACGCAAAATGAAACATT  3060
             ||||||||||||||||||||||||||||||||||||||||||||||||||||||||||||
Sbjct  3001  GCCAATGCAGCAAAACAGGGAAATGCAGTTTAATGTTGAACTGACGCAAAATGAAACATT  3060

Query  3061  TCTGGTCTGTTTAATAAATAGAAATATTCCAGTTTAGGTTGAACCAAATCTAAATATTTT  3120
             ||||||||||||||||||||||||||||||||||||||||||||||||||||||||||||
Sbjct  3061  TCTGGTCTGTTTAATAAATAGAAATATTCCAGTTTAGGTTGAACCAAATCTAAATATTTT  3120

Query  3121  GATTTGGGTTTCCAAAGGGAAGGaaaaaaatcagaaattcagcaaaattgaaattttcct  3180
             ||||||||||||||||||||||||||||||||||||||||||||||||||||||||||||
Sbjct  3121  GATTTGGGTTTCCAAAGGGAAGGAAAAAAATCAGAAATTCAGCAAAATTGAAATTTTCCT  3180

Query  3181  acaaaaaatattttttttttGCAGAAATTGCATTTCCTGTCAGAAAATCATTCTGACAGA  3240
             ||||||||||||||||||||||||||||||||||||||||||||||||||||||||||||
Sbjct  3181  ACAAAAAATATTTTTTTTTTGCAGAAATTGCATTTCCTGTCAGAAAATCATTCTGACAGA  3240

Query  3241  AGATTCCTGATCAGACTTAATTCCATCCTGTCTAATTTAAAACTATACAGGGTTAAGTGC  3300
             ||||||||||||||||||||||||||||||||||||||||||||||||||||||||||||
Sbjct  3241  AGATTCCTGATCAGACTTAATTCCATCCTGTCTAATTTAAAACTATACAGGGTTAAGTGC  3300

Query  3301  TTGAATATACCCCAAGGAATAATTTTGTGCTAAATTCCTAAATTTTGTGTTAATAAGGGA  3360
             ||||||||||||||||||||||||||||||||||||||||||||||||||||||||||||
Sbjct  3301  TTGAATATACCCCAAGGAATAATTTTGTGCTAAATTCCTAAATTTTGTGTTAATAAGGGA  3360

Query  3361  GTGCATAGGAAGACCCAATGGGTCTTTTCCTTCTTCCCTTCCTGGGCTGGATTGTATCCA  3420
             ||||||||||||||||||||||||||||||||||||||||||||||||||||||||||||
Sbjct  3361  GTGCATAGGAAGACCCAATGGGTCTTTTCCTTCTTCCCTTCCTGGGCTGGATTGTATCCA  3420

Query  3421  TGGCAGGAGGCACGTAACTCCATTGTCCCAGGAGCAAGGCAAGGCAGGAACTGTGCTGAG  3480
             ||||||||||||||||||||||||||||||||||||||||||||||||||||||||||||
Sbjct  3421  TGGCAGGAGGCACGTAACTCCATTGTCCCAGGAGCAAGGCAAGGCAGGAACTGTGCTGAG  3480

Query  3481  ATGCTGAGAGTCACAGTCCGGTCCCTGCTTCTTCCTTGTGCCATGGGAGAAGTGAGCTGT  3540
             ||||||||||||||||||||||||||||||||||||||||||||||||||||||||||||
Sbjct  3481  ATGCTGAGAGTCACAGTCCGGTCCCTGCTTCTTCCTTGTGCCATGGGAGAAGTGAGCTGT  3540

Query  3541  GCCACTCCTTTTGCTGCCGCAGCCTACTTTCTCTCTCTGAACTAGCTCCAGTGCACTGGC  3600
             ||||||||||||||||||||||||||||||||||||||||||||||||||||||||||||
Sbjct  3541  GCCACTCCTTTTGCTGCCGCAGCCTACTTTCTCTCTCTGAACTAGCTCCAGTGCACTGGC  3600

Query  3601  TGGTACCTTGGGAGGGGTGGAGCCACACCTTGCCTCTGCCTTCGCAGCCTGACCACTCCC  3660
             ||||||||||||||||||||||||||||||||||||||||||||||||||||||||||||
Sbjct  3601  TGGTACCTTGGGAGGGGTGGAGCCACACCTTGCCTCTGCCTTCGCAGCCTGACCACTCCC  3660

Query  3661  TGCCTACCTACATGGATGGGCCTAGCAATTTGGGTGGCCTATGCAGCGGAATAAGGGGAT  3720
             ||||||||||||||||||||||||||||||||||||||||||||||||||||||||||||
Sbjct  3661  TGCCTACCTACATGGATGGGCCTAGCAATTTGGGTGGCCTATGCAGCGGAATAAGGGGAT  3720

Query  3721  TCCTTACTCCCCTGCAGCAGAACTCATGAGTGAGTCCTCCATGGTAATTTTATGGATATT  3780
             ||||||||||||||||||||||||||||||||||||||||||||||||||||||||||||
Sbjct  3721  TCCTTACTCCCCTGCAGCAGAACTCATGAGTGAGTCCTCCATGGTAATTTTATGGATATT  3780

Query  3781  ATCATATAATCACTGACTGCATCTTCTTCCTGGCCTTCTGAGCCACAAGTCACTGCCAAG  3840
             ||||||||||||||||||||||||||||||||||||||||||||||||||||||||||||
Sbjct  3781  ATCATATAATCACTGACTGCATCTTCTTCCTGGCCTTCTGAGCCACAAGTCACTGCCAAG  3840

Query  3841  TACTTCATCCTGTAATTTAACAGAGTGCTGCAATTAGAATATACTGTGAAAAGAATGTCT  3900
             ||||||||||||||||||||||||||||||||||||||||||||||||||||||||||||
Sbjct  3841  TACTTCATCCTGTAATTTAACAGAGTGCTGCAATTAGAATATACTGTGAAAAGAATGTCT  3900

Query  3901  TATACTCTGGCTCAAGTATAGCAGACAAATATGTACAGAAAGCAATAATACACAGGGGCA  3960
             ||||||||||||||||||||||||||||||||||||||||||||||||||||||||||||
Sbjct  3901  TATACTCTGGCTCAAGTATAGCAGACAAATATGTACAGAAAGCAATAATACACAGGGGCA  3960

Query  3961  AAAATTTCTGGTTTAAAACTGCATATGCATTTCCAGACGTGAGAAACGTGTATATTTCAT  4020
             ||||||||||||||||||||||||||||||||||||||||||||||||||||||||||||
Sbjct  3961  AAAATTTCTGGTTTAAAACTGCATATGCATTTCCAGACGTGAGAAACGTGTATATTTCAT  4020

Query  4021  TGTGTCCACATGCCTCAGAGTTTTTATTGTACTTTTATAATCTTCTTTTAAAACTGTTAA  4080
             ||||||||||||||||||||||||||||||||||||||||||||||||||||||||||||
Sbjct  4021  TGTGTCCACATGCCTCAGAGTTTTTATTGTACTTTTATAATCTTCTTTTAAAACTGTTAA  4080

Query  4081  ATAGACGCTGTAAATTAAACTTTGAATACTTTGTAAGGCCTGACAGTAATAGACAAGTAT  4140
             ||||||||||||||||||||||||||||||||||||||||||||||||||||||||||||
Sbjct  4081  ATAGACGCTGTAAATTAAACTTTGAATACTTTGTAAGGCCTGACAGTAATAGACAAGTAT  4140

Query  4141  TACTTTTTAATGCAATTGTTAATATTATTTGTTTTCTCTCCCTTAACACTTCATTGGGTT  4200
             ||||||||||||||||||||||||||||||||||||||||||||||||||||||||||||
Sbjct  4141  TACTTTTTAATGCAATTGTTAATATTATTTGTTTTCTCTCCCTTAACACTTCATTGGGTT  4200

Query  4201  TCAGTTTACATATACAGACAGTAGCAGCGTAGTATTCTAATGCATAGTGCTTTGTAGTTC  4260
             ||||||||||||||||||||||||||||||||||||||||||||||||||||||||||||
Sbjct  4201  TCAGTTTACATATACAGACAGTAGCAGCGTAGTATTCTAATGCATAGTGCTTTGTAGTTC  4260

Query  4261  ATATATTCGGTAAGTATTTGATAAACAAGTGAACACATACTCAATATTTACACACTGGAA  4320
             ||||||||||||||||||||||||||||||||||||||||||||||||||||||||||||
Sbjct  4261  ATATATTCGGTAAGTATTTGATAAACAAGTGAACACATACTCAATATTTACACACTGGAA  4320

Query  4321  TGCTATGGGCCCGTTTCCAAACAAACGTTAATTTGTGTGGGTGACCAAAAAAGGGGCACA  4380
             ||||||||||||||||||||||||||||||||||||||||||||||||||||||||||||
Sbjct  4321  TGCTATGGGCCCGTTTCCAAACAAACGTTAATTTGTGTGGGTGACCAAAAAAGGGGCACA  4380

Query  4381  AATAAGGAGGATGCAACAAAAGTGTACATGTGTGGGGAGGAGCAGGAAGGAAAGTGC  4437
             |||||||||||||||||||||||||||||||||||||||||||||||||||||||||
Sbjct  4381  AATAAGGAGGATGCAACAAAAGTGTACATGTGTGGGGAGGAGCAGGAAGGAAAGTGC  4437
```
