# Supplementary material for: Divergent IL18-STAT1 Immune Responses Underlie Differential Susceptibility to Aeromonas hydrophila in Geoclemys hamiltonii and Trachemys scripta: A Comparative Transcriptomic Perspective
Source: Genes (Basel). 2026 Apr 9;17(4):436. doi: 10.3390/genes17040436 (PMC13116093; doi:10.3390/genes17040436)
Supplement: Supplementary file 1 [file genes-17-00436-s001.zip › Figure S2/CD40LG.pdf]

PREDICTED: Trachemys scripta elegans CD40 ligand (CD40LG), mRNA

Sequence ID: XM_034782600.1 Length: 3642 Number of Matches: 1

Range 1: 1 to 3642 GenBank Graphics ▼ Next Match ▲ Previous Match

Score	Expect	Identities	Gaps	Strand
6726 bits(3642)	0.0	3642/3642(100%)	0/3642(0%)	Plus/Plus

```
Query  1     GTAGCCCACGAAAGCTTATGCTCAAATAAAGTTGTTAGTCTCTAAGGTGCCACAAGGACT  60
             ||||||||||||||||||||||||||||||||||||||||||||||||||||||||||||
Sbjct  1     GTAGCCCACGAAAGCTTATGCTCAAATAAAGTTGTTAGTCTCTAAGGTGCCACAAGGACT  60

Query  61    CCTGTTCTTTTAGAAGATTTACTGAAAACTTGGCTATACAAGCAAATAGATACTAGAAGA  120
             ||||||||||||||||||||||||||||||||||||||||||||||||||||||||||||
Sbjct  61    CCTGTTCTTTTAGAAGATTTACTGAAAACTTGGCTATACAAGCAAATAGATACTAGAAGA  120

Query  121   TGTAGTACCCAAGACTATGGCTGTTGATATGATCGCACATAAAAGCCCCAGCCAAGCCTG  180
             ||||||||||||||||||||||||||||||||||||||||||||||||||||||||||||
Sbjct  121   TGTAGTACCCAAGACTATGGCTGTTGATATGATCGCACATAAAAGCCCCAGCCAAGCCTG  180

Query  181   GCAGTCCATTTTGCTAGGTGCTGTACAAACACAGGGTAAGAGACATTCCTTGCCCTAAAG  240
             ||||||||||||||||||||||||||||||||||||||||||||||||||||||||||||
Sbjct  181   GCAGTCCATTTTGCTAGGTGCTGTACAAACACAGGGTAAGAGACATTCCTTGCCCTAAAG  240

Query  241   AGAGACCATCAAAGCAGACACAAGAAGTATTATTAcccccccTTTTCACATGCGGTGAGG  300
             ||||||||||||||||||||||||||||||||||||||||||||||||||||||||||||
Sbjct  241   AGAGACCATCAAAGCAGACACAAGAAGTATTATTACCCCCCCTTTTCACATGCGGTGAGG  300

Query  301   TCAGGCATAGAGAGATTACGCAACTTGCCCATGGTCACAAAGGGGGAGACTGTGATAAAG  360
             ||||||||||||||||||||||||||||||||||||||||||||||||||||||||||||
Sbjct  301   TCAGGCATAGAGAGATTACGCAACTTGCCCATGGTCACAAAGGGGGAGACTGTGATAAAG  360

Query  361   CCAGGAACTGAATTCGCATCTCCTGAGTCCTATCTAGGTCCTTAATTACAAAACCATCTT  420
             ||||||||||||||||||||||||||||||||||||||||||||||||||||||||||||
Sbjct  361   CCAGGAACTGAATTCGCATCTCCTGAGTCCTATCTAGGTCCTTAATTACAAAACCATCTT  420

Query  421   TCCTCACTGAGGAAAACAAAAATTAAAGTAAGACTTTTTGGGAAATAGTAAAAACCAACA  480
             ||||||||||||||||||||||||||||||||||||||||||||||||||||||||||||
Sbjct  421   TCCTCACTGAGGAAAACAAAAATTAAAGTAAGACTTTTTGGGAAATAGTAAAAACCAACA  480

Query  481   CACACAATCTTTTACTCTCCACATTGAAATTCAACAGCTTTGCTTGTGGATTTGTTTTCC  540
             ||||||||||||||||||||||||||||||||||||||||||||||||||||||||||||
Sbjct  481   CACACAATCTTTTACTCTCCACATTGAAATTCAACAGCTTTGCTTGTGGATTTGTTTTCC  540

Query  541   TCATTTCTTTGAAGCCATCGGCCATGAAATGTTTGTCCTCAGTCGGaaaaaaaTAAAGTT  600
             ||||||||||||||||||||||||||||||||||||||||||||||||||||||||||||
Sbjct  541   TCATTTCTTTGAAGCCATCGGCCATGAAATGTTTGTCCTCAGTCGGAAAAAAATAAAGTT  600

Query  601   ATTTTCTGGAGCACTGCTAGGCAAGTATGAATACATGGAATAAGAACATTCAACTGACTT  660
             ||||||||||||||||||||||||||||||||||||||||||||||||||||||||||||
Sbjct  601   ATTTTCTGGAGCACTGCTAGGCAAGTATGAATACATGGAATAAGAACATTCAACTGACTT  660

Query  661   CTGATAGAACAATAGGAAAAAGTATGGAAGTGAATGAAAGAAGAAAGTTCAAGTCACTTT  720
             ||||||||||||||||||||||||||||||||||||||||||||||||||||||||||||
Sbjct  661   CTGATAGAACAATAGGAAAAAGTATGGAAGTGAATGAAAGAAGAAAGTTCAAGTCACTTT  720

Query  721   TTGTTGTGGGAGATGGCAATGAACAAACCTCCCCACTACGAAGGGTTTTTTCCAGGAAGT  780
             ||||||||||||||||||||||||||||||||||||||||||||||||||||||||||||
Sbjct  721   TTGTTGTGGGAGATGGCAATGAACAAACCTCCCCACTACGAAGGGTTTTTTCCAGGAAGT  780

Query  781   GTAGGCTGTCACATCTATCTGTCCCTTAACTATCTTGAGTAGTGTGCTAGCGAGGCAGTT  840
             ||||||||||||||||||||||||||||||||||||||||||||||||||||||||||||
Sbjct  781   GTAGGCTGTCACATCTATCTGTCCCTTAACTATCTTGAGTAGTGTGCTAGCGAGGCAGTT  840

Query  841   CTCATGCTGCTCCTGCACCGTGCTTTTGAAGGGGAGGCCACAGCCACTTGAACACAGAAT  900
             ||||||||||||||||||||||||||||||||||||||||||||||||||||||||||||
Sbjct  841   CTCATGCTGCTCCTGCACCGTGCTTTTGAAGGGGAGGCCACAGCCACTTGAACACAGAAT  900

Query  901   GAACGAACCTTATAGTCCAACGACGCCTCGACCTAGCAGTACCAGCTCACCTAACACCAT  960
             ||||||||||||||||||||||||||||||||||||||||||||||||||||||||||||
Sbjct  901   GAACGAACCTTATAGTCCAACGACGCCTCGACCTAGCAGTACCAGCTCACCTAACACCAT  960

Query  961   GAAAATTTTTATGTGTTTTCTTATTGTATTTATTATAGCACAGACTATTGGGACTGTACT  1020
             ||||||||||||||||||||||||||||||||||||||||||||||||||||||||||||
Sbjct  961   GAAAATTTTTATGTGTTTTCTTATTGTATTTATTATAGCACAGACTATTGGGACTGTACT  1020

Query  1021  TTTTTGCTTATATCTTCACATGAAGCTGGATAAGTTGGAACAGGAGTTGAGCTTACAGGA  1080
             ||||||||||||||||||||||||||||||||||||||||||||||||||||||||||||
Sbjct  1021  TTTTTGCTTATATCTTCACATGAAGCTGGATAAGTTGGAACAGGAGTTGAGCTTACAGGA  1080

Query  1081  AGATTATTTGTTCCTCCGAAGAATACAAAAATGTCGGAAACCAGAAGGTGCGGGCTCATC  1140
             ||||||||||||||||||||||||||||||||||||||||||||||||||||||||||||
Sbjct  1081  AGATTATTTGTTCCTCCGAAGAATACAAAAATGTCGGAAACCAGAAGGTGCGGGCTCATC  1140

Query  1141  ATTATTGGACTGTAAGGAGATCATAAACCGATTCCAGGATCTGCTAGTCAAAGATCCAGA  1200
             ||||||||||||||||||||||||||||||||||||||||||||||||||||||||||||
Sbjct  1141  ATTATTGGACTGTAAGGAGATCATAAACCGATTCCAGGATCTGCTAGTCAAAGATCCAGA  1200

Query  1201  AGTCAGCAAGGATGATGCAAAATTTGAAATGCAAAAAGATGACAGGCAGCAACCAATTTC  1260
             ||||||||||||||||||||||||||||||||||||||||||||||||||||||||||||
Sbjct  1201  AGTCAGCAAGGATGATGCAAAATTTGAAATGCAAAAAGATGACAGGCAGCAACCAATTTC  1260

Query  1261  AGCTCACCTAATGGGGTTCAAGAACAGCACCAAGAAAGTATCAGTTTTACAATGGCAGAA  1320
             ||||||||||||||||||||||||||||||||||||||||||||||||||||||||||||
Sbjct  1261  AGCTCACCTAATGGGGTTCAAGAACAGCACCAAGAAAGTATCAGTTTTACAATGGCAGAA  1320

Query  1321  AACGGGGTATGCCCCTATGAGCAACCTGATATCCTACAAAGGAGGGAAATTGAAGGTGGA  1380
             ||||||||||||||||||||||||||||||||||||||||||||||||||||||||||||
Sbjct  1321  AACGGGGTATGCCCCTATGAGCAACCTGATATCCTACAAAGGAGGGAAATTGAAGGTGGA  1380

Query  1381  AAAAGAAGGGCTCTACTACATCTACTCCCAGGTCAGCTTCTGCACCAAGACAGCTCCTGG  1440
             ||||||||||||||||||||||||||||||||||||||||||||||||||||||||||||
Sbjct  1381  AAAAGAAGGGCTCTACTACATCTACTCCCAGGTCAGCTTCTGCACCAAGACAGCTCCTGG  1440

Query  1441  GGCGCCATTTACGGTGTTTATTTATTTGAACCTACCATCGGAATCTGATCGGCTCTTATT  1500
             ||||||||||||||||||||||||||||||||||||||||||||||||||||||||||||
Sbjct  1441  GGCGCCATTTACGGTGTTTATTTATTTGAACCTACCATCGGAATCTGATCGGCTCTTATT  1500

Query  1501  AAAGGGACAGGATACGCACAGCTCATCCAGTGTTTACTGTGCCCTACAATCCACTCACCT  1560
             ||||||||||||||||||||||||||||||||||||||||||||||||||||||||||||
Sbjct  1501  AAAGGGACAGGATACGCACAGCTCATCCAGTGTTTACTGTGCCCTACAATCCACTCACCT  1560

Query  1561  GGGAGGAGTGTTTGAACTCCGGAAAGGTGATGTGGTGTTTGTTAATGTGACAGATTCGAC  1620
             ||||||||||||||||||||||||||||||||||||||||||||||||||||||||||||
Sbjct  1561  GGGAGGAGTGTTTGAACTCCGGAAAGGTGATGTGGTGTTTGTTAATGTGACAGATTCGAC  1620

Query  1621  TCAAGTGAACTATGATCATGGAAACACATACTTTGGTATGTTTAAACTCTATTGAGAAGT  1680
             ||||||||||||||||||||||||||||||||||||||||||||||||||||||||||||
Sbjct  1621  TCAAGTGAACTATGATCATGGAAACACATACTTTGGTATGTTTAAACTCTATTGAGAAGT  1680

Query  1681  GACCTGCTATGAAGAACAGAGCTGCAGGAGGCCCAATCCCAACTTTGTTGAACAAAGTGA  1740
             ||||||||||||||||||||||||||||||||||||||||||||||||||||||||||||
Sbjct  1681  GACCTGCTATGAAGAACAGAGCTGCAGGAGGCCCAATCCCAACTTTGTTGAACAAAGTGA  1740

Query  1741  ATCCTGGTTTCCAATATTTATGTTCCTCTGCATCTTTAGTTTTCTTGTATCTAGTTCTGT  1800
             ||||||||||||||||||||||||||||||||||||||||||||||||||||||||||||
Sbjct  1741  ATCCTGGTTTCCAATATTTATGTTCCTCTGCATCTTTAGTTTTCTTGTATCTAGTTCTGT  1800

Query  1801  ATATTTTGTTATTTATTAACGGGGGCAGAATGAGCCCTTAGAACGGAAACAGAACAAAGT  1860
             ||||||||||||||||||||||||||||||||||||||||||||||||||||||||||||
Sbjct  1801  ATATTTTGTTATTTATTAACGGGGGCAGAATGAGCCCTTAGAACGGAAACAGAACAAAGT  1860

Query  1861  TTGGAGCTGTTACTGTGTGCACTTTGTTCTCACCTTTACTCATTGTGGAATGTCTGTACA  1920
             ||||||||||||||||||||||||||||||||||||||||||||||||||||||||||||
Sbjct  1861  TTGGAGCTGTTACTGTGTGCACTTTGTTCTCACCTTTACTCATTGTGGAATGTCTGTACA  1920

Query  1921  CATCTGTATCATATGTATTTAAGCCCATGCCCTGTGTGCTATCACTTGTCTGACAGGGTG  1980
             ||||||||||||||||||||||||||||||||||||||||||||||||||||||||||||
Sbjct  1921  CATCTGTATCATATGTATTTAAGCCCATGCCCTGTGTGCTATCACTTGTCTGACAGGGTG  1980

Query  1981  GATATGCTTATTTAGTAGAACATAATATGAGTGTTTCTTTCCAGACAACATTACGTATCT  2040
             ||||||||||||||||||||||||||||||||||||||||||||||||||||||||||||
Sbjct  1981  GATATGCTTATTTAGTAGAACATAATATGAGTGTTTCTTTCCAGACAACATTACGTATCT  2040

Query  2041  CTCAGGGGACTCCAGTCAGATAGATTATATGTACCTCTTTTTCAATCTGCTTATAAAGTA  2100
             ||||||||||||||||||||||||||||||||||||||||||||||||||||||||||||
Sbjct  2041  CTCAGGGGACTCCAGTCAGATAGATTATATGTACCTCTTTTTCAATCTGCTTATAAAGTA  2100

Query  2101  ACAATGGAAAAACTTTAATGGTTTAGAAATATCTTCTCTAGGAAAAAAGCAATCAGCAAA  2160
             ||||||||||||||||||||||||||||||||||||||||||||||||||||||||||||
Sbjct  2101  ACAATGGAAAAACTTTAATGGTTTAGAAATATCTTCTCTAGGAAAAAAGCAATCAGCAAA  2160

Query  2161  TTGCCATCCTTAAGCGGTAACTGCTAATGACACATATTTACCATTTAAATCGTATATTTC  2220
             ||||||||||||||||||||||||||||||||||||||||||||||||||||||||||||
Sbjct  2161  TTGCCATCCTTAAGCGGTAACTGCTAATGACACATATTTACCATTTAAATCGTATATTTC  2220

Query  2221  ACCTGAAGTCTCCCTTCTTTTCATCTCTGTATATGTACAATATACAATCAGCAGAACCTT  2280
             ||||||||||||||||||||||||||||||||||||||||||||||||||||||||||||
Sbjct  2221  ACCTGAAGTCTCCCTTCTTTTCATCTCTGTATATGTACAATATACAATCAGCAGAACCTT  2280

Query  2281  GTACCTTTTATAATCATGTGATATTTGCTGAGGCTTGTTCACACGGGATGAAATTCACTC  2340
             ||||||||||||||||||||||||||||||||||||||||||||||||||||||||||||
Sbjct  2281  GTACCTTTTATAATCATGTGATATTTGCTGAGGCTTGTTCACACGGGATGAAATTCACTC  2340

Query  2341  TGGAGCAGAAGGGCCTGCACAAGGTCCATGTGCCACTCGTGTCCTCAGAACAGGGCTTCA  2400
             ||||||||||||||||||||||||||||||||||||||||||||||||||||||||||||
Sbjct  2341  TGGAGCAGAAGGGCCTGCACAAGGTCCATGTGCCACTCGTGTCCTCAGAACAGGGCTTCA  2400

Query  2401  GTGAGATGTAAATAGTCCTCTGCCCCAGGGTGAATTTCATCCACAGGCCTAGACTGTCCT  2460
             ||||||||||||||||||||||||||||||||||||||||||||||||||||||||||||
Sbjct  2401  GTGAGATGTAAATAGTCCTCTGCCCCAGGGTGAATTTCATCCACAGGCCTAGACTGTCCT  2460

Query  2461  GCATAGATTTGTGAATGAAGAGGACACTGCTCATGGAACAACCCTCACCCTGTGGAAGGG  2520
             ||||||||||||||||||||||||||||||||||||||||||||||||||||||||||||
Sbjct  2461  GCATAGATTTGTGAATGAAGAGGACACTGCTCATGGAACAACCCTCACCCTGTGGAAGGG  2520

Query  2521  AGCCACCCTCCCCGTACCACGACACCTTCTCTCCAGGCCCCCTGGACAACCCTCTTCCAT  2580
             ||||||||||||||||||||||||||||||||||||||||||||||||||||||||||||
Sbjct  2521  AGCCACCCTCCCCGTACCACGACACCTTCTCTCCAGGCCCCCTGGACAACCCTCTTCCAT  2580

Query  2581  GGGCCTTTATTCCACATTGGGGAAGAAATGAGAAAGCAGAAGGCTGGACCAGGCAGGCTC  2640
             ||||||||||||||||||||||||||||||||||||||||||||||||||||||||||||
Sbjct  2581  GGGCCTTTATTCCACATTGGGGAAGAAATGAGAAAGCAGAAGGCTGGACCAGGCAGGCTC  2640

Query  2641  CCTTGTCCAGTCCTATGATACACCTCCAAGCAGTATTATCTATTGTGTAGAGCCTCCCCT  2700
             ||||||||||||||||||||||||||||||||||||||||||||||||||||||||||||
Sbjct  2641  CCTTGTCCAGTCCTATGATACACCTCCAAGCAGTATTATCTATTGTGTAGAGCCTCCCCT  2700

Query  2701  GGGCATTTTGGGGCTACCACTGCCAGGGTAGGTCCTGTTAGAGCGGCTTCATTGGGGTCA  2760
             ||||||||||||||||||||||||||||||||||||||||||||||||||||||||||||
Sbjct  2701  GGGCATTTTGGGGCTACCACTGCCAGGGTAGGTCCTGTTAGAGCGGCTTCATTGGGGTCA  2760

Query  2761  CTAGGGCCATAGCTGGTGCAGAGGGACTGGACCTCTAAGGCCCCAGAGCCTTTTCCATGT  2820
             ||||||||||||||||||||||||||||||||||||||||||||||||||||||||||||
Sbjct  2761  CTAGGGCCATAGCTGGTGCAGAGGGACTGGACCTCTAAGGCCCCAGAGCCTTTTCCATGT  2820

Query  2821  GTGAACAATGGGTAATATGGGGATGTCCTTGCTAAGATTTTTCCCCCCAGACCTTCCTCC  2880
             ||||||||||||||||||||||||||||||||||||||||||||||||||||||||||||
Sbjct  2821  GTGAACAATGGGTAATATGGGGATGTCCTTGCTAAGATTTTTCCCCCCAGACCTTCCTCC  2880

Query  2881  CATGGACTGTGTGAGAGGGTGATCCGGGCCATAATAAATTAAGACAGTTACATAGCACCT  2940
             ||||||||||||||||||||||||||||||||||||||||||||||||||||||||||||
Sbjct  2881  CATGGACTGTGTGAGAGGGTGATCCGGGCCATAATAAATTAAGACAGTTACATAGCACCT  2940

Query  2941  TGCACCCAAAGATCCCAAATACCAAGAGACTTTTCAGGGTCCTGATCCTGGAACTCTGAC  3000
             ||||||||||||||||||||||||||||||||||||||||||||||||||||||||||||
Sbjct  2941  TGCACCCAAAGATCCCAAATACCAAGAGACTTTTCAGGGTCCTGATCCTGGAACTCTGAC  3000

Query  3001  TCACTCAAGTAGTGCTCACTCATGGGAGTAGCTCTGTTGAAGTAAATAAGATTGCTTGTA  3060
             ||||||||||||||||||||||||||||||||||||||||||||||||||||||||||||
Sbjct  3001  TCACTCAAGTAGTGCTCACTCATGGGAGTAGCTCTGTTGAAGTAAATAAGATTGCTTGTA  3060

Query  3061  TAAGGGTTGCAGGATTGGGACATAGGATTTTTGCCTGATTAAAGGTTGCAAGATTGGCCC  3120
             ||||||||||||||||||||||||||||||||||||||||||||||||||||||||||||
Sbjct  3061  TAAGGGTTGCAGGATTGGGACATAGGATTTTTGCCTGATTAAAGGTTGCAAGATTGGCCC  3120

Query  3121  ATATAGCATAGCATGGTCACTTGCCCCATCTTCAATATGCAGCCACCTCTGGGGTGGAAA  3180
             ||||||||||||||||||||||||||||||||||||||||||||||||||||||||||||
Sbjct  3121  ATATAGCATAGCATGGTCACTTGCCCCATCTTCAATATGCAGCCACCTCTGGGGTGGAAA  3180

Query  3181  GTAGTAGTCTTAACACGCAGAACACACCAATACCAGTGACTGTGGATTTAAGGCCTGATG  3240
             ||||||||||||||||||||||||||||||||||||||||||||||||||||||||||||
Sbjct  3181  GTAGTAGTCTTAACACGCAGAACACACCAATACCAGTGACTGTGGATTTAAGGCCTGATG  3240

Query  3241  CTGCACCTATGGCTATCAATGGGAGGCATGCTCAGAATCTGCCCGTCTAATTTTACTATA  3300
             ||||||||||||||||||||||||||||||||||||||||||||||||||||||||||||
Sbjct  3241  CTGCACCTATGGCTATCAATGGGAGGCATGCTCAGAATCTGCCCGTCTAATTTTACTATA  3300

Query  3301  ATTTAATCTGTAGTGTTGGTGGCATAACTTTATTAGAGAATGGAGTCAGTGAAATATTTT  3360
             ||||||||||||||||||||||||||||||||||||||||||||||||||||||||||||
Sbjct  3301  ATTTAATCTGTAGTGTTGGTGGCATAACTTTATTAGAGAATGGAGTCAGTGAAATATTTT  3360

Query  3361  TGTTTGATGCATTGTGTAAAAATTATTTATTTAATTAAAGCTGTATTATATTCATGGACA  3420
             ||||||||||||||||||||||||||||||||||||||||||||||||||||||||||||
Sbjct  3361  TGTTTGATGCATTGTGTAAAAATTATTTATTTAATTAAAGCTGTATTATATTCATGGACA  3420

Query  3421  AAGGGAAGGTGCATTGAAAAATAGAAAGTGTGCAAGGAATAAATATGACTGTTTGTGCAG  3480
             ||||||||||||||||||||||||||||||||||||||||||||||||||||||||||||
Sbjct  3421  AAGGGAAGGTGCATTGAAAAATAGAAAGTGTGCAAGGAATAAATATGACTGTTTGTGCAG  3480

Query  3481  TAATGTTGGATCCCCAGTATTGAATGTTGAGTTCTGAAGTGCCAAACAGGAAGTGCTGTG  3540
             ||||||||||||||||||||||||||||||||||||||||||||||||||||||||||||
Sbjct  3481  TAATGTTGGATCCCCAGTATTGAATGTTGAGTTCTGAAGTGCCAAACAGGAAGTGCTGTG  3540

Query  3541  TTGCTTACACATCACAGCACTGAGCTAATTTGCAGTCTCGCTACAACAATTGGTGTAGAA  3600
             ||||||||||||||||||||||||||||||||||||||||||||||||||||||||||||
Sbjct  3541  TTGCTTACACATCACAGCACTGAGCTAATTTGCAGTCTCGCTACAACAATTGGTGTAGAA  3600

Query  3601  TTCTCCATACTGTAGTGTTACCAATAACATACCATTTATAAA  3642
             ||||||||||||||||||||||||||||||||||||||||||
Sbjct  3601  TTCTCCATACTGTAGTGTTACCAATAACATACCATTTATAAA  3642
```
