# Supplementary material for: Divergent IL18-STAT1 Immune Responses Underlie Differential Susceptibility to Aeromonas hydrophila in Geoclemys hamiltonii and Trachemys scripta: A Comparative Transcriptomic Perspective
Source: Genes (Basel). 2026 Apr 9;17(4):436. doi: 10.3390/genes17040436 (PMC13116093; doi:10.3390/genes17040436)
Supplement: Supplementary file 1 [file genes-17-00436-s001.zip › Figure S2/CGAS.pdf]

PREDICTED: Trachemys scripta elegans cyclic GMP-AMP synthase (CGAS), mRNA

Sequence ID: XM_034766204.1 **Length:** 2118 **Number of Matches:** 1

Range 1: 1 to 2118 GenBank Graphics ▼ Next Match ▲ Previous Match

Score	Expect	Identities	Gaps	Strand
3912 bits(2118)	0.0	2118/2118(100%)	0/2118(0%)	Plus/Plus

```
Query  1     GGGGCTTGGGATGCAGGAAGAGGCTCTGGGCTGGAGGTTGGGGCCAAGGGTTCGGAATGG  60
             ||||||||||||||||||||||||||||||||||||||||||||||||||||||||||||
Sbjct  1     GGGGCTTGGGATGCAGGAAGAGGCTCTGGGCTGGAGGTTGGGGCCAAGGGTTCGGAATGG  60

Query  61    CCGGCTTTTACAAACTCCCCTCAGTCACAACACCTTGCCCCGCGGCTGGCCTCGCTGCTA  120
             ||||||||||||||||||||||||||||||||||||||||||||||||||||||||||||
Sbjct  61    CCGGCTTTTACAAACTCCCCTCAGTCACAACACCTTGCCCCGCGGCTGGCCTCGCTGCTA  120

Query  121   CTGCACCCGGGTTGTGGCCCAGCGGGAGGGAACAGGGTCATGCCCGTCCCTGAGTTTCGT  180
             ||||||||||||||||||||||||||||||||||||||||||||||||||||||||||||
Sbjct  121   CTGCACCCGGGTTGTGGCCCAGCGGGAGGGAACAGGGTCATGCCCGTCCCTGAGTTTCGT  180

Query  181   TTCTCCAGCTGCGCGCCCTGGTCCCGGAAAGGAAGTTGCCTGAGCCTGGTTTCGCGCTTA  240
             ||||||||||||||||||||||||||||||||||||||||||||||||||||||||||||
Sbjct  181   TTCTCCAGCTGCGCGCCCTGGTCCCGGAAAGGAAGTTGCCTGAGCCTGGTTTCGCGCTTA  240

Query  241   GCGCCTCAGTGCCCGGAGCCAGGGAGGCGCGGAAGGCGGTGGGAGCTGAGCCATGGGGGA  300
             ||||||||||||||||||||||||||||||||||||||||||||||||||||||||||||
Sbjct  241   GCGCCTCAGTGCCCGGAGCCAGGGAGGCGCGGAAGGCGGTGGGAGCTGAGCCATGGGGGA  300

Query  301   CCAGTGCGCTGCAGCCAGGAGAGCGAGTGGGTCGAGAAAGACACCGCGTCAGAGCAGCGC  360
             ||||||||||||||||||||||||||||||||||||||||||||||||||||||||||||
Sbjct  301   CCAGTGCGCTGCAGCCAGGAGAGCGAGTGGGTCGAGAAAGACACCGCGTCAGAGCAGCGC  360

Query  361   TCCTGAGCCCCCTCGGAGCCATAGCGCCGCCGCCAAGCCCCCTGCCCGCGGCGGGGGGAG  420
             ||||||||||||||||||||||||||||||||||||||||||||||||||||||||||||
Sbjct  361   TCCTGAGCCCCCTCGGAGCCATAGCGCCGCCGCCAAGCCCCCTGCCCGCGGCGGGGGGAG  420

Query  421   AGCTGGGGCAGGAAGAGGGGACGCTCGGTCTCCTGGTGAGCATGCTAACGCCATTAACCC  480
             ||||||||||||||||||||||||||||||||||||||||||||||||||||||||||||
Sbjct  421   AGCTGGGGCAGGAAGAGGGGACGCTCGGTCTCCTGGTGAGCATGCTAACGCCATTAACCC  480

Query  481   CCGGGACTTCCCTTCGCCCATTGACCCAAAGGGCGCCACAGCTGCCAGACCCCGCACAGA  540
             ||||||||||||||||||||||||||||||||||||||||||||||||||||||||||||
Sbjct  481   CCGGGACTTCCCTTCGCCCATTGACCCAAAGGGCGCCACAGCTGCCAGACCCCGCACAGA  540

Query  541   GAAGGGACCCGCTCCCAGCGAGAAGGGATCTGCCCCGGGAGAGAGTAATGCTGTCCCCAG  600
             ||||||||||||||||||||||||||||||||||||||||||||||||||||||||||||
Sbjct  541   GAAGGGACCCGCTCCCAGCGAGAAGGGATCTGCCCCGGGAGAGAGTAATGCTGTCCCCAG  600

Query  601   TAGGAAGGGAGCAGTCTCCACTCGGAAAGACTCTGCCCGCAGAGAGAAGGGAGCTGTCCC  660
             ||||||||||||||||||||||||||||||||||||||||||||||||||||||||||||
Sbjct  601   TAGGAAGGGAGCAGTCTCCACTCGGAAAGACTCTGCCCGCAGAGAGAAGGGAGCTGTCCC  660

Query  661   GAAAACAAAAGAGTCTCCCGCCAGAGGGCCGCCCGCTGTAGCTTCCTACTGCTGCACGGG  720
             ||||||||||||||||||||||||||||||||||||||||||||||||||||||||||||
Sbjct  661   GAAAACAAAAGAGTCTCCCGCCAGAGGGCCGCCCGCTGTAGCTTCCTACTGCTGCACGGG  720

Query  721   GACAGAGAGCCGCGTCGGGGTCAAGTCTGGTGGCGTGGGAGCCCGGCGGCTCAGGGATGT  780
             ||||||||||||||||||||||||||||||||||||||||||||||||||||||||||||
Sbjct  721   GACAGAGAGCCGCGTCGGGGTCAAGTCTGGTGGCGTGGGAGCCCGGCGGCTCAGGGATGT  780

Query  781   GCTGAAGACACTGAGCCTGGGCCGGCAGGACACGTCCGAGGCCTCGGAGAATGTGAACCA  840
             ||||||||||||||||||||||||||||||||||||||||||||||||||||||||||||
Sbjct  781   GCTGAAGACACTGAGCCTGGGCCGGCAGGACACGTCCGAGGCCTCGGAGAATGTGAACCA  840

Query  841   GCTGATCCGCACGTTGGTGTCAGCGATCAGAGCCCGGGAGAGCAGTTTCAGCTCAATTGA  900
             ||||||||||||||||||||||||||||||||||||||||||||||||||||||||||||
Sbjct  841   GCTGATCCGCACGTTGGTGTCAGCGATCAGAGCCCGGGAGAGCAGTTTCAGCTCAATTGA  900

Query  901   TATTCTGGGCACCGGTAGCTACTACGAGCATGTCAAGATTTCTGCACCAAATGAGTTTGA  960
             ||||||||||||||||||||||||||||||||||||||||||||||||||||||||||||
Sbjct  901   TATTCTGGGCACCGGTAGCTACTACGAGCATGTCAAGATTTCTGCACCAAATGAGTTTGA  960

Query  961   TATCATGTTTAAGATGCCAGCTCCTAGAGTTGAACTGGAACAATGTGATAGCTCTGGTGC  1020
             ||||||||||||||||||||||||||||||||||||||||||||||||||||||||||||
Sbjct  961   TATCATGTTTAAGATGCCAGCTCCTAGAGTTGAACTGGAACAATGTGATAGCTCTGGTGC  1020

Query  1021  CTTTTATTATGTGAGACTTAAAAGAAATCCTCAAGGAAACAGTCTGGACAAATTTTTACA  1080
             ||||||||||||||||||||||||||||||||||||||||||||||||||||||||||||
Sbjct  1021  CTTTTATTATGTGAGACTTAAAAGAAATCCTCAAGGAAACAGTCTGGACAAATTTTTACA  1080

Query  1081  AGAAGATGGAACATTAGCAGCCTGTAAGATGCTTTTTGCCCTGAGGAACATTATTAAAGA  1140
             ||||||||||||||||||||||||||||||||||||||||||||||||||||||||||||
Sbjct  1081  AGAAGATGGAACATTAGCAGCCTGTAAGATGCTTTTTGCCCTGAGGAACATTATTAAAGA  1140

Query  1141  AATTGTAAAGAGCATGACACGAACAGAAATGAAAGTGACTGTGGATaaaaaaaaGGCTGG  1200
             ||||||||||||||||||||||||||||||||||||||||||||||||||||||||||||
Sbjct  1141  AATTGTAAAGAGCATGACACGAACAGAAATGAAAGTGACTGTGGATAAAAAAAAGGCTGG  1200

Query  1201  AAGCCCTGCAATAACACTTCGCATTGGGAATCCTCCAATGGAGATATCAGTGGATATAAT  1260
             ||||||||||||||||||||||||||||||||||||||||||||||||||||||||||||
Sbjct  1201  AAGCCCTGCAATAACACTTCGCATTGGGAATCCTCCAATGGAGATATCAGTGGATATAAT  1260

Query  1261  CTTGGCTTTGGAAGTTCGAAGTCAGAGCTGGCCTGCCAGTACACAGGAGGGCCTAAAAAT  1320
             ||||||||||||||||||||||||||||||||||||||||||||||||||||||||||||
Sbjct  1261  CTTGGCTTTGGAAGTTCGAAGTCAGAGCTGGCCTGCCAGTACACAGGAGGGCCTAAAAAT  1320

Query  1321  TGAAAAATGGCTAGGAAGCAAAGTCAAACAAGAATATAAATGGAAGCCAATATACCTAGT  1380
             ||||||||||||||||||||||||||||||||||||||||||||||||||||||||||||
Sbjct  1321  TGAAAAATGGCTAGGAAGCAAAGTCAAACAAGAATATAAATGGAAGCCAATATACCTAGT  1380

Query  1381  ACCCAAACATGCCAAGGATGGAAGAGTGCTAAAAGAAGACACCTGGCGACTCTCTTTCTC  1440
             ||||||||||||||||||||||||||||||||||||||||||||||||||||||||||||
Sbjct  1381  ACCCAAACATGCCAAGGATGGAAGAGTGCTAAAAGAAGACACCTGGCGACTCTCTTTCTC  1440

Query  1441  ACACATTGAAAAGGACATGATAAAGAACCATGGCAACACAAAGACATGTTGTGAATCTAA  1500
             ||||||||||||||||||||||||||||||||||||||||||||||||||||||||||||
Sbjct  1441  ACACATTGAAAAGGACATGATAAAGAACCATGGCAACACAAAGACATGTTGTGAATCTAA  1500

Query  1501  GGGAGTAAAGTGTTGTAGGAAAAGCTGTCTGAAACTTCTGAAGCATCTTCTGGATCAGCT  1560
             ||||||||||||||||||||||||||||||||||||||||||||||||||||||||||||
Sbjct  1501  GGGAGTAAAGTGTTGTAGGAAAAGCTGTCTGAAACTTCTGAAGCATCTTCTGGATCAGCT  1560

Query  1561  TAAAACAAAAGATGGAAACAGACGGGGGCTGGACAAATTCTGTTCCTACCATGCCAAAAC  1620
             ||||||||||||||||||||||||||||||||||||||||||||||||||||||||||||
Sbjct  1561  TAAAACAAAAGATGGAAACAGACGGGGGCTGGACAAATTCTGTTCCTACCATGCCAAAAC  1620

Query  1621  TGCCTTTTTCCAAGCATGTGTCCTTTGGCCAGATGACAAACAATGGCTGTTCACAGACCT  1680
             ||||||||||||||||||||||||||||||||||||||||||||||||||||||||||||
Sbjct  1621  TGCCTTTTTCCAAGCATGTGTCCTTTGGCCAGATGACAAACAATGGCTGTTCACAGACCT  1680

Query  1681  TGAGAGCTGTTTTCAAAAATTTTTGGATTACTTTCTGGATTGCCTCAACAACGCATACCT  1740
             ||||||||||||||||||||||||||||||||||||||||||||||||||||||||||||
Sbjct  1681  TGAGAGCTGTTTTCAAAAATTTTTGGATTACTTTCTGGATTGCCTCAACAACGCATACCT  1740

Query  1741  TCCACACTTTTTTATTCCTACACACAACCTTTTTAGTAGACCACTGATTGATAAGGCAAG  1800
             ||||||||||||||||||||||||||||||||||||||||||||||||||||||||||||
Sbjct  1741  TCCACACTTTTTTATTCCTACACACAACCTTTTTAGTAGACCACTGATTGATAAGGCAAG  1800

Query  1801  CAGTGATTTCCTTTCAAAGGAAATTAAATATGAAATAAACAATAGATTTCCAATATTTGA  1860
             ||||||||||||||||||||||||||||||||||||||||||||||||||||||||||||
Sbjct  1801  CAGTGATTTCCTTTCAAAGGAAATTAAATATGAAATAAACAATAGATTTCCAATATTTGA  1860

Query  1861  ACTGCAGAATTAAGGAAATATTCTATGATGTTTGTCAAAATTTTCATTTACATTTTATAG  1920
             ||||||||||||||||||||||||||||||||||||||||||||||||||||||||||||
Sbjct  1861  ACTGCAGAATTAAGGAAATATTCTATGATGTTTGTCAAAATTTTCATTTACATTTTATAG  1920

Query  1921  ATCAAAATATCTAATAAAGAATCAACATTTGAAGTTATTCCTTGGGAATTTCTGGTGCTA  1980
             ||||||||||||||||||||||||||||||||||||||||||||||||||||||||||||
Sbjct  1921  ATCAAAATATCTAATAAAGAATCAACATTTGAAGTTATTCCTTGGGAATTTCTGGTGCTA  1980

Query  1981  TGAAAAGTGGAAGTAAAGTAATTGTGGTCAATAAATGAGACTTACACATTTAAATTAAAT  2040
             ||||||||||||||||||||||||||||||||||||||||||||||||||||||||||||
Sbjct  1981  TGAAAAGTGGAAGTAAAGTAATTGTGGTCAATAAATGAGACTTACACATTTAAATTAAAT  2040

Query  2041  TATGCTTCTTAGTGGTAGAGGAGAAGGGATATCAGGACAAGTTTTCACAATTTTAAGTC  2100
             ||||||||||||||||||||||||||||||||||||||||||||||||||||||||||||
Sbjct  2041  TATGCTTCTTAGTGGTAGAGGAGAAGGGATATCAGGACAAGTTTTCACAATTTTAAGTC  2100

Query  2101  TGATTGGTGCCAGGCTAC  2118
             ||||||||||||||||||
Sbjct  2101  TGATTGGTGCCAGGCTAC  2118
```
