# Supplementary material for: Divergent IL18-STAT1 Immune Responses Underlie Differential Susceptibility to Aeromonas hydrophila in Geoclemys hamiltonii and Trachemys scripta: A Comparative Transcriptomic Perspective
Source: Genes (Basel). 2026 Apr 9;17(4):436. doi: 10.3390/genes17040436 (PMC13116093; doi:10.3390/genes17040436)
Supplement: Supplementary file 1 [file genes-17-00436-s001.zip › Figure S2/DDX58.pdf]

PREDICTED: Trachemys scripta elegans DExD/H-box helicase 58 (DDX58), transcript variant X1, mRNA

Sequence ID: XM_034774409.1 Length: 3728 Number of Matches: 1

Range 1: 329 to 3124 GenBank Graphics

▼ Next Match ▲ Previous Match

Score	Expect	Identities	Gaps	Strand
4455 bits(2412)	0.0	2668/2796(95%)	0/2796(0%)	Plus/Plus

```
Query  1     ATGACTGCGGAGCAGAAGGAGAGCCTGCGGTGCTACAGGCAGTACATTGAGAAGACCCTG  60
Sbjct  329   ATGACTGCGGAGCAGAAAGAGACCCTGCGGTGCTACAGGCAGTACATTGAGAAGATACTG  388

Query  61    AATCCTGTCTATGTGCTGGGCAACATGAAGGAGTGGCTCTCTGACGATGCCAGAGAGAGA  120
Sbjct  389   AATCCTGCCTATGTGCTGGGCAACATGAAGGACTGGCTCTCTGATGGTGCCAGAGAGAGA  448

Query  121   ATTCAGACTGAGGAACAGAAGAAGGGGCTGACTGCTGCAGCGTCTCTCTTTGTTGACTCT  180
Sbjct  449   ATTCAGATTGAGGAACAGAAGGAGGGGCTGACTGCTGCTGCGGCTCTCTTTGTTGACTCT  508

Query  181   ATACTGCAGCTCGAATCAGAAGGATGGTTTCGGGGATTTCTAGATGCACTGAATGAAGCA  240
Sbjct  509   ATACTGCAGCTCGAATCAGAAGGATGGTTTCGGGGATTTCTAGATGCACTGAATGAAGCA  568

Query  241   GGTTACACTGGTCTGGGGGAAGCAATTGAAAACTGGGATTTCCAAAAACTTGAAAGCCTG  300
Sbjct  569   GGTTACACTGGTCTGAGGGAAGCAATTGAAAACTCGGATTTTCAAAAACTTGAAAGCCTG  628

Query  301   GAGGTGCATCGACAGCTGCTGAAACGTATAGAAGCTACAATGAGAGACATTGATGCAGAA  360
Sbjct  629   GAGGTGCATCGGCAGCTGCTGAAACGTATAGAAGCTACGATGAGAGACATTGATGCAGAA  688

Query  361   CAGATAATTCCTTATTTAAACACTTGTCTCATAGACCGAGAATGTGAAGAGATTCAGCAG  420
Sbjct  689   CAGATAATTCCTTACTTAAACACTTGTCTCATAGACCGAGAATGCGAAGAGATTCAGCAG  748

Query  421   GTCAAAGAACAGAAAGGCAGAATGGCTGGTGCAGTGAAGCTCATTGAATGCCTTGCTAGG  480
Sbjct  749   GTCAAAGAACTGAAAGGCAGAATGGCTGGTGCTGTAAAGCTCATTGAATGCCTTTCTAGG  808

Query  481   TCAGACAAGGAAAACTGGCCCAAAACCTTTCATCTGGCTCTAGAACGAGCAGGATATGAC  540
Sbjct  809   TCAGACAAGGAAAACTGGCCCAAAACCTTTCATCTGGCTCTAGAACAAGCAGGATATGAC  868

Query  541   CCAGAAAGCAAACTCTGGAATATGAAAGAAGGTAACGACAAAGAGATGGATGTTGAAATG  600
Sbjct  869   CGAGCAAGCAAACTGTGGAATATGAAAGAAGGTGACAATAAAGAGATGGATGTTGAAATG  928

Query  601   ATGGAGGACCAGAATGGGAACAGTAGTGTCTTAGACATAAATGTACAGTATTCCGAAGAA  660
Sbjct  929   ATGGAGGACCAGAATGGGACCAGTAGTGCCTTCGACATAAATGTACAGTATTCCGAAGAA  988

Query  661   GCAGAAGTTCACAATTTCAGTGAAAGTCCGTGTTCTCCTTCAGAAGTTTCTCAGCAATCT  720
Sbjct  989   GCAGAAGTTCACAATTTCAGTGAAAGTCCGTGTTCTCCTTCAGAAGTGTCTCAACAACCT  1048

Query  721   ACTTATGGACCAAAGAAGGCTAGAAGTTATCAGATTGAGCTTGCCGGGCCTGCTGTCAGT  780
Sbjct  1049  ACTTATGGACCAAAGAAGGCTAGAAGTTATCAGATTGAGCTTGCCCGGCCTGCTGTCAGT  1108

Query  781   GGGAAAAATACAATCATATGTGCTCCTACTGGATCTGGGAAAACCTTCGTGGCACTTCTG  840
Sbjct  1109  GGGAAAAATACAATCATATGTGCTCCTACTGGATCTGGGAAAACCTTTGTGGCACTTCTG  1168

Query  841   ATTTGTGACAATCATCTTCAAAACATGCCCAAGGAACAGAAAGGGAAGATTGTCTTTCTA  900
Sbjct  1169  ATTTGTGACGATCATCTCCAAAACATGCCCAAGGAACAGAAAGGGAAGATTGTCTTTCTA  1228

Query  901   GCTACTAAAGTTCCAGTATATGAACAACAGAAAAAAGTCTTCACACAACACTTTGAAAGA  960
Sbjct  1229  GCTACTAAAGTTCCAGTATATGAACAACAGAAAAAAGTCTTCACGCAACACTTTGAAAGA  1288

Query  961   ACTAGGTACAAAGTTGCAGGGATTTCTGGGGAAACAGCTGGGGATGTCTCCTCAGCAAGT  1020
Sbjct  1289  ACTAGGTACGAAGTTGCGGGGATTTCTGGGGAAACGGCTGGGGATGTCTCTTCAGCAAGC  1348

Query  1021  GTCATTGAAGGGAATGATATCATTGTCTTGACGCCTCAGATCCTCGTGAATGGCCTCAGA  1080
Sbjct  1349  GTCATTGAAGGGAATGATATCATTGTCTTGACACCTCAGATCCTTGTGAATGCTCTCAAA  1408

Query  1081  GATGGAACTGTTCCTTCCCTTGCCATTTTCACATTGATTATATTTGATGAGTGCCACAAC  1140
Sbjct  1409  GATGGAACTGTTCCTTCCCTTGCCATTTTCACTTTGATTATATTTGATGAGTGCCACAAC  1468

Query  1141  ACCACTGGGAATCACCCCTATAATGTGTTAATGACCAGCTACTTGAATCTTAAATTTGAC  1200
Sbjct  1469  ACCACTGGGAATCACCCCTATAATGTGTTAATGACCAGCTACTTGAATCTTAAATTTGAC  1528

Query  1201  TCCGCTACGAAACAGCTGCCTCAGATTGTTGGCTTAACAGCCTCGGTCGGAGTGGGCAGT  1260
Sbjct  1529  TCCGCTGTGAAACAGCTGCCTCAGATTGTTGGCTTAACAGCCTCGGTCGGCGTGGGCAGT  1588

Query  1261  GCCAAGACCCTTGAGGAGACGATAGAATACATCTGTACCCTCTGCGCCTGCCTCGACATA  1320
Sbjct  1589  GCCAAGACCCTCGAGGAGACGATAGAATACATCTGTACCCTCTGCGCCTGCCTCGACATA  1648

Query  1321  CAAGTCATATCTACCATCAGAGAGAACACACAAGAACTGGAGGAGATTGTATACAGGCCC  1380
Sbjct  1649  CAAGTCATATCTACCATCAGAGAGAACACACAAGAACTGGAGGAGATTGTATACAGGCCC  1708

Query  1381  CAAAAATCTTTCAGGCTAGTCGGGAAGCGCCCCAAAAACCACTTTGTGGACATTATCTCA  1440
Sbjct  1709  CAAAAATCTTTCAGACTAGTCGGGAAGCGCCCCAAAAACCACTTTATGGCCATTATCTCA  1768

Query  1441  GTCCTGATGTCTGAAACTGAGACCCTGGCAAGCAAGATTTACCCAATAGATGCGTTGTCC  1500
Sbjct  1769  GTCCTGATGTCTGAAACTGAGGCCCTGGCAAGCAAGATTTACCCAATAGATGCGTTGTCC  1828

Query  1501  CACATTAAGAATAAACGTTTTGGAACGCAGTGCTATGAACAGTGGATTGTTGACACTCAG  1560
Sbjct  1829  CACATTAAGAGTAAACGTTTCGGAACGCAATGCTATGAACAGTGGATCGTTGACACTCAG  1888

Query  1561  AAGAAGTGCAGAATGTTGCAACTGCTGGATAAGGAAGAAGAGAAGAGGATTTGTAGAGCT  1620
Sbjct  1889  AAGAAGTGCAGAATGTTGCAACTGCCGGACAAGGAAGAAGAGAGGAGGATTTGTAGAGCT  1948

Query  1621  CTTTTATTTACACTGAACACTTACGGAAATACAATGATGCCCTCATCATCAACGAAGAT  1680
Sbjct  1949  CTCTTTATTTATACTGAACACTTACGGAAATACAATGATGCCCTCATCATCAATGAAGAT  2008

Query  1681  GCCCGGACTCAGGATGCATTGGCCTACTTGACTGAGTTTTTCAATGACATTAGAAGTGGA  1740
Sbjct  2009  GCACGGACTCAGGATGCGTTGGCCTACTTGACTGAATTTTTCAATGACCTTAGAAGTGGA  2068

Query  1741  GGGTTTGATGAGATAGATCAACAGTTAACAGCGAACTTTGAAGCTAAACAACAGGAACTG  1800
Sbjct  2069  GGGTTTGATGAGATAGATCAACAGTTAACAGCGAACTTTGAAGCTAAACAACAGGAACTG  2128

Query  1801  AGAGAGGCCTCAGTAGATGAACTGAATGAGAATCCCAAACTGGAGGAGCTCACCTTCATC  1860
Sbjct  2129  AGAGAGGCCGCAGTAGATGAGCTGAATGAGAATCCCAAACTGGAGGAGCTCACCTTCATC  2188

Query  1861  CTGAATGAAGAATACCGCCTAAAGCCAGAGACCCGCACCCTTCTCTTTGTTACCACAAGA  1920
Sbjct  2189  CTGAATGAAGAATACCGTCTAAAGCCAGAGACCCGCACTCTGCTCTTTGTTACCACAAGA  2248

Query  1921  GCTCTTGTGTCTGCTTTGAAGAAATGGATTGATGAAAATCCTACACTCAGCTACCTAAAA  1980
Sbjct  2249  GCGCTAGTGTCCGCTTTGAAGAAATGGATTGATGAAAATCCTACACTCAGCTACCTAAAA  2308

Query  1981  CCAGATGCATTGATGGGGCGTAGCAAAAGAAACCAGCAGACAGGGATGACACTCTCAAAT  2040
Sbjct  2309  CCGGATGTGTTGATGGGGCGTAACAAAAGAAACCAGCAGACAGGGATGACGCTCTCAAAT  2368

Query  2041  CAAAAGGGCGTACTGGACTCGTTCAAAACCAACAGCGACAGTAAGATACTAATAACCACA  2100
Sbjct  2369  CAAAAGGACGTACTGGACTCATTCAAAACCAACAGCGACAGTAAGATACTAATAACTACA  2428

Query  2101  GCTGTCGCAGATGAAGGGATTGATATTGCTCAGTGCAACCTGGTTCTCCTCTATGAATAC  2160
Sbjct  2429  GCAGTTGCAGATGAAGGGATTGATATTGCTCAGTGCAACCTGGTTCTCCTCTATGAATAC  2488

Query  2161  TCTGGCAATGTCACCAAAATGATCCAAGTCCGAGGTCGCGGAAGGGCAAAAGGCAGCAAG  2220
Sbjct  2489  TCTGGCAACGTCACCAAAATGATCCAAGTCCGAGGTCGTGGAAGGGCAAAAGACAGCAAG  2548

Query  2221  TGCATCCTTGTGACAAGCAAAAGCGAAGTGGCTGAGAATGAGAGAAACAATATCTATAAG  2280
Sbjct  2549  TGCATCCTTGTGACAAGCAAAAGCGAAGTGGCTGAGAATGAGAGAAACAATATCTATAAG  2608

Query  2281  GAGGAGATGATGAACAAGGCCATCAAGCAGCTGCAGGAGTGGGATGAGGAAAAGTTCGCA  2340
Sbjct  2609  GAGGAGATGATGAACAAAGCCATCAAGCAGCTGCAGGAGTGGGATGAAGAAAAGTTCGCA  2668

Query  2341  AGGAAGATAAATGACCTGCAAAATAAGGAAAAGACACTGCGAGATTCCAGAAAGAGAGAA  2400
Sbjct  2669  AGGAAGATAAATGACCTGCAAAACAAGGAAAAGACCCTGCGAGATTCCAGAAAGAGAGAA  2728

Query  2401  ATAAAACAAAAGCCTCTGGAGGGCAAGAGACAGTTGCTCTGTGGGAAGTGCAGAGCATAT  2460
Sbjct  2729  ATTAAACAAAAACCCCTGGAGGGCAATAGACAGTTGCTCTGTGGGAAGTGCAAAGCATAT  2788

Query  2461  GCCTGTAATACAGAAGACATCAGGGTAATAGAGAAGTCTCACCACACAGTCTTAGATGCC  2520
Sbjct  2789  GCCTGTAACACAGAAGACATCCGGGTAATAGAGAAGGCTCACCACACCGTCTTAGGTGCC  2848

Query  2521  CTGTTTCAGGAACGTTTTCTAACAAAGCCTCACACGAAACCAAACCGCTATGACCACTTT  2580
Sbjct  2849  CTGTTTCGGGAACGTTTTCTAACAAAGCCTCACAAAAAACCAAGCCGCTATGACCACTTT  2908

Query  2581  GAGAAGAAATGCAAAATGTACTGCCGGGACGCCAAGTGCCAGCACGACTGGGGAATCACA  2640
Sbjct  2909  GAGAAGAAATGTAAAATGTCCTGCCGGGACTCCAAGTGCCAGCATGATTGGGGCATCACA  2968

Query  2641  GTGAGGTACAAGGCATTCGATGACCTCCCCGTGATCAAAATTGAAAGCTTTGTGGTGAAG  2700
Sbjct  2969  GTGAGGTACAAGGCATTCGATGACCTCCCAGTGATCAAAATTGAAAGCTTTGTGGTGAAG  3028

Query  2701  GATGTTACTACTAGTAAACTTGCAGTTTTCCGGAAATGGAGAGAGGTGGATTTTGCAATG  2760
Sbjct  3029  GATATTACAACTGGGAAACTCGCAGTTTTCCGGAAATGGAGAGAGGTGGATTTCGCAATG  3088

Query  2761  AGGGAATTTGACATAAATGAAATGTCCAGCACAGAG  2796
Sbjct  3089  AAGGAATTTGACATTAATGAAATGTCCAGCACAGAG  3124
```
