# Supplementary material for: Divergent IL18-STAT1 Immune Responses Underlie Differential Susceptibility to Aeromonas hydrophila in Geoclemys hamiltonii and Trachemys scripta: A Comparative Transcriptomic Perspective
Source: Genes (Basel). 2026 Apr 9;17(4):436. doi: 10.3390/genes17040436 (PMC13116093; doi:10.3390/genes17040436)
Supplement: Supplementary file 1 [file genes-17-00436-s001.zip › Figure S2/EGR2.pdf]

PREDICTED: Trachemys scripta elegans early growth response 2 (EGR2), mRNA

Sequence ID: XM_034777878.1 **Length:** 1792 **Number of Matches:** 1

Range 1: 1 to 1792 GenBank Graphics ▼ Next Match ▲ Previous Match

Score	Expect	Identities	Gaps	Strand
3310 bits(1792)	0.0	1792/1792(100%)	0/1792(0%)	Plus/Plus

```
Query  1     CGGCTCGCCCTTCCCTGTTCCTCAGTCCTTATATGGGCAGTGACGTCCTGGGCATTCAGG  60
             ||||||||||||||||||||||||||||||||||||||||||||||||||||||||||||
Sbjct  1     CGGCTCGCCCTTCCCTGTTCCTCAGTCCTTATATGGGCAGTGACGTCCTGGGCATTCAGG  60

Query  61    GCCTCCCCATAAATACTTACTGCAACACTTTCCATCTACTGAGAGCTGCAAGTGATTAAC  120
             ||||||||||||||||||||||||||||||||||||||||||||||||||||||||||||
Sbjct  61    GCCTCCCCATAAATACTTACTGCAACACTTTCCATCTACTGAGAGCTGCAAGTGATTAAC  120

Query  121   AGCTCTGCAAGGGACACTGACTGTTACAATAACACTACAGCAACTACTGGGCTTTTACAG  180
             ||||||||||||||||||||||||||||||||||||||||||||||||||||||||||||
Sbjct  121   AGCTCTGCAAGGGACACTGACTGTTACAATAACACTACAGCAACTACTGGGCTTTTACAG  180

Query  181   AGCAACACAGAGGCAGTAACAGACTTTAAGTATAAATTTACTTGAttttttttAGCGTGG  240
             ||||||||||||||||||||||||||||||||||||||||||||||||||||||||||||
Sbjct  181   AGCAACACAGAGGCAGTAACAGACTTTAAGTATAAATTTACTTGATTTTTTTTAGCGTGG  240

Query  241   AAGAAACAAATAACCCTACAGTATTAACACTACAAGTAGTGAAAGTTGTTTTCAAGGTTG  300
             ||||||||||||||||||||||||||||||||||||||||||||||||||||||||||||
Sbjct  241   AAGAAACAAATAACCCTACAGTATTAACACTACAAGTAGTGAAAGTTGTTTTCAAGGTTG  300

Query  301   TTTATTGACCAAATGATGACTGCCAAGACCGTAGACAAAATTCCAGTAACTCTCAGTGGT  360
             ||||||||||||||||||||||||||||||||||||||||||||||||||||||||||||
Sbjct  301   TTTATTGACCAAATGATGACTGCCAAGACCGTAGACAAAATTCCAGTAACTCTCAGTGGT  360

Query  361   TTTGTGCATCAGCTATCCGAAAACATTTACCCTGTGGATGACATCGCTACCACATTGCCA  420
             ||||||||||||||||||||||||||||||||||||||||||||||||||||||||||||
Sbjct  361   TTTGTGCATCAGCTATCCGAAAACATTTACCCTGTGGATGACATCGCTACCACATTGCCA  420

Query  421   ACTTCGGTCACAATCTTCCCCAATGCTGACTTAGGAGGACCGTTTGACCAGATGAGCAGT  480
             ||||||||||||||||||||||||||||||||||||||||||||||||||||||||||||
Sbjct  421   ACTTCGGTCACAATCTTCCCCAATGCTGACTTAGGAGGACCGTTTGACCAGATGAGCAGT  480

Query  481   GTGACAGGAGATGGAATGATCAATATTGACATGAGTGACAAGAGGTCCCTGGATCTGCCT  540
             ||||||||||||||||||||||||||||||||||||||||||||||||||||||||||||
Sbjct  481   GTGACAGGAGATGGAATGATCAATATTGACATGAGTGACAAGAGGTCCCTGGATCTGCCT  540

Query  541   TATGCCAGCAGCTTTGCCCCAGCAGTTTCTGCTTCCCGCAATCAGACTTTTACCTACATG  600
             ||||||||||||||||||||||||||||||||||||||||||||||||||||||||||||
Sbjct  541   TATGCCAGCAGCTTTGCCCCAGCAGTTTCTGCTTCCCGCAATCAGACTTTTACCTACATG  600

Query  601   GGCAAATTCTCCATCGACCCTCAGTACCCAGGAGCCGGCTGCTATCCGGAGGGGATCATT  660
             ||||||||||||||||||||||||||||||||||||||||||||||||||||||||||||
Sbjct  601   GGCAAATTCTCCATCGACCCTCAGTACCCAGGAGCCGGCTGCTATCCGGAGGGGATCATT  660

Query  661   AACATCGTGAGCGCTGGGATCCTGCAGGGGGTCAGCACCCCTTCTTCATCGGCCACTTCT  720
             ||||||||||||||||||||||||||||||||||||||||||||||||||||||||||||
Sbjct  661   AACATCGTGAGCGCTGGGATCCTGCAGGGGGTCAGCACCCCTTCTTCATCGGCCACTTCT  720

Query  721   TCCTCCACAGCCTCTTCTGCCTCCCCCAACCCGCTGGCCAGCAGCGCTCTGAGCTGCAGC  780
             ||||||||||||||||||||||||||||||||||||||||||||||||||||||||||||
Sbjct  721   TCCTCCACAGCCTCTTCTGCCTCCCCCAACCCGCTGGCCAGCAGCGCTCTGAGCTGCAGC  780

Query  781   ATGGCTCAGAACCAGCCCGGAGACCTGGAGCACCTGTACTCACCTCCGCCTCCCTACTCG  840
             ||||||||||||||||||||||||||||||||||||||||||||||||||||||||||||
Sbjct  781   ATGGCTCAGAACCAGCCCGGAGACCTGGAGCACCTGTACTCACCTCCGCCTCCCTACTCG  840

Query  841   GGCTGCGGGGAGCTCTACCAGCAGGACCCCTCCTCTGCTTTCCTGCCCGCCTCGGCCGGG  900
             ||||||||||||||||||||||||||||||||||||||||||||||||||||||||||||
Sbjct  841   GGCTGCGGGGAGCTCTACCAGCAGGACCCCTCCTCTGCTTTCCTGCCCGCCTCGGCCGGG  900

Query  901   GGCTCTCTTCCCTACCACCCGCCCCCGTCCTACCCTTCCCCCAAAGCGGCCGCGGACGGC  960
             ||||||||||||||||||||||||||||||||||||||||||||||||||||||||||||
Sbjct  901   GGCTCTCTTCCCTACCACCCGCCCCCGTCCTACCCTTCCCCCAAAGCGGCCGCGGACGGC  960

Query  961   GGGATCTTCTCTATGATCCCGGATTACCCGGGTTTCTTCCCGCCCGCTCAGTGCCAGCGG  1020
             ||||||||||||||||||||||||||||||||||||||||||||||||||||||||||||
Sbjct  961   GGGATCTTCTCTATGATCCCGGATTACCCGGGTTTCTTCCCGCCCGCTCAGTGCCAGCGG  1020

Query  1021  GAGCTGCACGCCCCGCCCGAGCGCAAGCCCTTCCCGTGCCCCCTGGACTCCCTCAGGGTC  1080
             ||||||||||||||||||||||||||||||||||||||||||||||||||||||||||||
Sbjct  1021  GAGCTGCACGCCCCGCCCGAGCGCAAGCCCTTCCCGTGCCCCCTGGACTCCCTCAGGGTC  1080

Query  1081  CCGCCGCCGCTCACGCCGCTCTCCACCATCCGCAACTTCACCCTgggcgggccgggggcc  1140
             ||||||||||||||||||||||||||||||||||||||||||||||||||||||||||||
Sbjct  1081  CCGCCGCCGCTCACGCCGCTCTCCACCATCCGCAACTTCACCCTGGGCGGGCCGGGGGCC  1140

Query  1141  ggagcagcagggggaagcggggaaggcggccggcTGCCCGCCAGCGCCTACAGCCCGCAC  1200
             ||||||||||||||||||||||||||||||||||||||||||||||||||||||||||||
Sbjct  1141  GGAGCAGCAGGGGGAAGCGGGGAAGGCGGCCGGCTGCCCGCCAGCGCCTACAGCCCGCAC  1200

Query  1201  AACTTGCCCCTGCGGCCCATCCTGCGGCCCCGCAAGTACCCGAACCGGCCCAGCAAGACG  1260
             ||||||||||||||||||||||||||||||||||||||||||||||||||||||||||||
Sbjct  1201  AACTTGCCCCTGCGGCCCATCCTGCGGCCCCGCAAGTACCCGAACCGGCCCAGCAAGACG  1260

Query  1261  CCGGTGCACGAGCGGCCCTACCCGTGCCCGGCAGAGGGCTGCGACCGCCGCTTCTCCCGC  1320
             ||||||||||||||||||||||||||||||||||||||||||||||||||||||||||||
Sbjct  1261  CCGGTGCACGAGCGGCCCTACCCGTGCCCGGCAGAGGGCTGCGACCGCCGCTTCTCCCGC  1320

Query  1321  TCGGACGAGCTAACGCGGCACATCCGCATCCACACCGGCCACAAGCCCTTCCAGTGCCGC  1380
             ||||||||||||||||||||||||||||||||||||||||||||||||||||||||||||
Sbjct  1321  TCGGACGAGCTAACGCGGCACATCCGCATCCACACCGGCCACAAGCCCTTCCAGTGCCGC  1380

Query  1381  ATCTGCATGCGCAACTTCAGCCGCAGCGACCACCTCACCACCCACATCCGCACGCACACG  1440
             ||||||||||||||||||||||||||||||||||||||||||||||||||||||||||||
Sbjct  1381  ATCTGCATGCGCAACTTCAGCCGCAGCGACCACCTCACCACCCACATCCGCACGCACACG  1440

Query  1441  GGCGAGAAGCCCTTCGCCTGCGACTTCTGCGGCCGGAAGTTCGCCCGCAGCGACGAGAGA  1500
             ||||||||||||||||||||||||||||||||||||||||||||||||||||||||||||
Sbjct  1441  GGCGAGAAGCCCTTCGCCTGCGACTTCTGCGGCCGGAAGTTCGCCCGCAGCGACGAGAGA  1500

Query  1501  AAGCGCCACACCAAGATCCACCTGCGCCAGAAGGAGAGGAAGGGCGCCGCCGCCCCCTCC  1560
             ||||||||||||||||||||||||||||||||||||||||||||||||||||||||||||
Sbjct  1501  AAGCGCCACACCAAGATCCACCTGCGCCAGAAGGAGAGGAAGGGCGCCGCCGCCCCCTCC  1560

Query  1561  TCCTCCAGCGGGACCAGCGCCCCGGCCGCGCCCCCGCCAGGTGCCGTGTGCAGCAGCAGC  1620
             ||||||||||||||||||||||||||||||||||||||||||||||||||||||||||||
Sbjct  1561  TCCTCCAGCGGGACCAGCGCCCCGGCCGCGCCCCCGCCAGGTGCCGTGTGCAGCAGCAGC  1620

Query  1621  GCCGTCGGCTCGGGGAGCCTGGCTGCCTGCGCCTCCAGGACCAGGACGCCCTGAGGCAGC  1680
             ||||||||||||||||||||||||||||||||||||||||||||||||||||||||||||
Sbjct  1621  GCCGTCGGCTCGGGGAGCCTGGCTGCCTGCGCCTCCAGGACCAGGACGCCCTGAGGCAGC  1680

Query  1681  CCCCCTGCGCGCGCACTATCGCCTGCTCCCCGGGCGCCACGGCGTAGCGCCGAGCGCTGC  1740
             ||||||||||||||||||||||||||||||||||||||||||||||||||||||||||||
Sbjct  1681  CCCCCTGCGCGCGCACTATCGCCTGCTCCCCGGGCGCCACGGCGTAGCGCCGAGCGCTGC  1740

Query  1741  CCGGCCAGGCCCGGCCCGCCCTCACACCTCCCGCCCCGGCCGCCGGAGAGGG  1792
             ||||||||||||||||||||||||||||||||||||||||||||||||||||
Sbjct  1741  CCGGCCAGGCCCGGCCCGCCCTCACACCTCCCGCCCCGGCCGCCGGAGAGGG  1792
```
