# Supplementary material for: Divergent IL18-STAT1 Immune Responses Underlie Differential Susceptibility to Aeromonas hydrophila in Geoclemys hamiltonii and Trachemys scripta: A Comparative Transcriptomic Perspective
Source: Genes (Basel). 2026 Apr 9;17(4):436. doi: 10.3390/genes17040436 (PMC13116093; doi:10.3390/genes17040436)
Supplement: Supplementary file 1 [file genes-17-00436-s001.zip › Figure S2/IL18.pdf]

# PREDICTED: Trachemys scripta elegans interleukin 18 (IL18), mRNA

**Sequence ID:** XM_034754572.1 **Length:** 1133 **Number of Matches:** 1

**Range 1: 1 to 1133** GenBank Graphics ▼ Next Match ▲ Previou

| Score | Expect | Identities | Gaps | Strand |
|---|---|---|---|---|
| 2093 bits(1133) | 0.0 | 1133/1133(100%) | 0/1133(0%) | Plus/Plus |

```
Query  1     CCGCCCCCGTCCCGCGAGCAGTCAGCGTGCGCTCCGCTCTCCCCGCCTCCAGCCGGGCTG  60
             ||||||||||||||||||||||||||||||||||||||||||||||||||||||||||||
Sbjct  1     CCGCCCCCGTCCCGCGAGCAGTCAGCGTGCGCTCCGCTCTCCCCGCCTCCAGCCGGGCTG  60

Query  61    CCCTTCCCTCCCCGCCTCTCGCTGAGCATTGCGCTCCGGACTCGGCTGCAGGAGGCAGAG  120
             ||||||||||||||||||||||||||||||||||||||||||||||||||||||||||||
Sbjct  61    CCCTTCCCTCCCCGCCTCTCGCTGAGCATTGCGCTCCGGACTCGGCTGCAGGAGGCAGAG  120

Query  121   TGAAGATGAGCTGTGAAGAGATCCGCGTGTTTGCAGTGACATTTGGGCAAGATTCTGACC  180
             ||||||||||||||||||||||||||||||||||||||||||||||||||||||||||||
Sbjct  121   TGAAGATGAGCTGTGAAGAGATCCGCGTGTTTGCAGTGACATTTGGGCAAGATTCTGACC  180

Query  181   TCTATTTTGCAGATGATGAAGGTCTGGAGTGCGACTACTGGAAGAAGGCAAACTACGCTC  240
             ||||||||||||||||||||||||||||||||||||||||||||||||||||||||||||
Sbjct  181   TCTATTTTGCAGATGATGAAGGTCTGGAGTGCGACTACTGGAAGAAGGCAAACTACGCTC  240

Query  241   TACAGCACCAGATCTTTCGAAACATATCTAATCAGGTACTCATGGTACGGCCAGGACCAG  300
             ||||||||||||||||||||||||||||||||||||||||||||||||||||||||||||
Sbjct  241   TACAGCACCAGATCTTTCGAAACATATCTAATCAGGTACTCATGGTACGGCCAGGACCAG  300

Query  301   AGAATGCTGTCATGAGTGTATTTGAGCCTATGACAGATCAAGAGATGACACCTGGATGCG  360
             ||||||||||||||||||||||||||||||||||||||||||||||||||||||||||||
Sbjct  301   AGAATGCTGTCATGAGTGTATTTGAGCCTATGACAGATCAAGAGATGACACCTGGATGCG  360

Query  361   GAGTGCACTTCACCATCCACTATTATGAAGACAACAGGTTTGAGGAGGGGATGTCCGTGG  420
             ||||||||||||||||||||||||||||||||||||||||||||||||||||||||||||
Sbjct  361   GAGTGCACTTCACCATCCACTATTATGAAGACAACAGGTTTGAGGAGGGGATGTCCGTGG  420

Query  421   CATTCAGCATCCTGGTAGAAAACAAGACTTACTGTATGTACTGCACACATGAAGGTGGGG  480
             ||||||||||||||||||||||||||||||||||||||||||||||||||||||||||||
Sbjct  421   CATTCAGCATCCTGGTAGAAAACAAGACTTACTGTATGTACTGCACACATGAAGGTGGGG  480

Query  481   AAAAGACTGTTCGATTTAGGGAAGAAGAAGTTCCCAGAGAAATTCTGGAAAACAGCAGTG  540
             ||||||||||||||||||||||||||||||||||||||||||||||||||||||||||||
Sbjct  481   AAAAGACTGTTCGATTTAGGGAAGAAGAAGTTCCCAGAGAAATTCTGGAAAACAGCAGTG  540

Query  541   ACATCATCTTCATCCAAAAGTCAGTTTCACCAACAGACACACAAGCATTCAAATTTGAAT  600
             ||||||||||||||||||||||||||||||||||||||||||||||||||||||||||||
Sbjct  541   ACATCATCTTCATCCAAAAGTCAGTTTCACCAACAGACACACAAGCATTCAAATTTGAAT  600

Query  601   CTTCACTAATGCGAGGATACTTCTTGGCATTTCAGAAAGTAGAGAATTTAAGCAAACTGA  660
             ||||||||||||||||||||||||||||||||||||||||||||||||||||||||||||
Sbjct  601   CTTCACTAATGCGAGGATACTTCTTGGCATTTCAGAAAGTAGAGAATTTAAGCAAACTGA  660

Query  661   TTTTAAAGCGATGTGATGAAGACTACGTGGATGAATCCACACATATTATTGTCCCCTAAA  720
             ||||||||||||||||||||||||||||||||||||||||||||||||||||||||||||
Sbjct  661   TTTTAAAGCGATGTGATGAAGACTACGTGGATGAATCCACACATATTATTGTCCCCTAAA  720

Query  721   TAAACTGATTTGATAATGTATCATACACAAGCAATTTCTTGGCCATTTTTATACAACTTT  780
             ||||||||||||||||||||||||||||||||||||||||||||||||||||||||||||
Sbjct  721   TAAACTGATTTGATAATGTATCATACACAAGCAATTTCTTGGCCATTTTTATACAACTTT  780

Query  781   TATTACTATCTCTATGCTAAACTGTGTAACCCTTCTCTGTAACCACTCAAACACACAGCC  840
             ||||||||||||||||||||||||||||||||||||||||||||||||||||||||||||
Sbjct  781   TATTACTATCTCTATGCTAAACTGTGTAACCCTTCTCTGTAACCACTCAAACACACAGCC  840

Query  841   TCTGGACACTGAGTTAAGTTCCTCAGGGTCTGTGCTGAGTTTATTGGCTCCAGGATTGAT  900
             ||||||||||||||||||||||||||||||||||||||||||||||||||||||||||||
Sbjct  841   TCTGGACACTGAGTTAAGTTCCTCAGGGTCTGTGCTGAGTTTATTGGCTCCAGGATTGAT  900

Query  901   ACTTTTCAATTCTTAGCTGCAGCTGAGAGCAAATATAGAAACAAAATAAATTGGTGGACA  960
             ||||||||||||||||||||||||||||||||||||||||||||||||||||||||||||
Sbjct  901   ACTTTTCAATTCTTAGCTGCAGCTGAGAGCAAATATAGAAACAAAATAAATTGGTGGACA  960

Query  961   TGGAGCTTAGGAATTCTATAATCAGAGAGATTAATGTCTGTTACTAGTTACAGCAGGGCT  1020
             ||||||||||||||||||||||||||||||||||||||||||||||||||||||||||||
Sbjct  961   TGGAGCTTAGGAATTCTATAATCAGAGAGATTAATGTCTGTTACTAGTTACAGCAGGGCT  1020

Query  1021  TCTATGGTAAGGCTGCTATTTACCACAATGCCTTCTCCaaaaaaaGCTAGTCTTTGCATA  1080
             ||||||||||||||||||||||||||||||||||||||||||||||||||||||||||||
Sbjct  1021  TCTATGGTAAGGCTGCTATTTACCACAATGCCTTCTCCAAAAAAAGCTAGTCTTTGCATA  1080

Query  1081  AGATTGTCATTGCCAACTTTTTGAATTAAGTATTAAATTTGGAATTAAATGAA  1133
             |||||||||||||||||||||||||||||||||||||||||||||||||||||
Sbjct  1081  AGATTGTCATTGCCAACTTTTTGAATTAAGTATTAAATTTGGAATTAAATGAA  1133
```
