# Supplementary material for: Divergent IL18-STAT1 Immune Responses Underlie Differential Susceptibility to Aeromonas hydrophila in Geoclemys hamiltonii and Trachemys scripta: A Comparative Transcriptomic Perspective
Source: Genes (Basel). 2026 Apr 9;17(4):436. doi: 10.3390/genes17040436 (PMC13116093; doi:10.3390/genes17040436)
Supplement: Supplementary file 1 [file genes-17-00436-s001.zip › Figure S2/IRF7.pdf]

PREDICTED: Trachemys scripta elegans interferon regulatory factor 7 (IRF7), transcript variant X3, mRNA

Sequence ID: XM_034770403.1 Length: 7885 Number of Matches: 1

Range 1: 1 to 7885 GenBank Graphics ▼ Next Match ▲ Previous Match

Score	Expect	Identities	Gaps	Strand
14534 bits(7870)	0.0	7885/7891(99%)	6/7891(0%)	Plus/Plus

```
Query  1     ACGACACGTTCCAGAAGAGTATAAAAGGGAGACTCCTTGCTGGAAATACTTTGATATTTC  60
             ||||||||||||||||||||||||||||||||||||||||||||||||||||||||||||
Sbjct  1     ACGACACGTTCCAGAAGAGTATAAAAGGGAGACTCCTTGCTGGAAATACTTTGATATTTC  60

Query  61    AACTATTCACAAAATCCCTGGTAACATCGTCTGGTAACAGCAAAGGTCCTGCTTGGAGGT  120
             ||||||||||||||||||||||||||||||||||||||||||||||||||||||||||||
Sbjct  61    AACTATTCACAAAATCCCTGGTAACATCGTCTGGTAACAGCAAAGGTCCTGCTTGGAGGT  120

Query  121   TTCCAATTCATCCACAAGAATTCCGTTTGGTAGCAAAGGAGTGAACTCCCCAATCCTTCC  180
             ||||||||||||||||||||||||||||||||||||||||||||||||||||||||||||
Sbjct  121   TTCCAATTCATCCACAAGAATTCCGTTTGGTAGCAAAGGAGTGAACTCCCCAATCCTTCC  180

Query  181   TTCCGCAATTAACCTGCCACTGGCTGCGTGATGGCTGCATCCGAGAGCGAGGGGGGTTCT  240
             ||||||||||||||||||||||||||||||||||||||||||||||||||||||||||||
Sbjct  181   TTCCGCAATTAACCTGCCACTGGCTGCGTGATGGCTGCATCCGAGAGCGAGGGGGGTTCT  240

Query  241   CAAAAATTACGATTTGCCTTCTGGCTGATTAAGCAGATCAACAGCGAAAACTACGAGGGG  300
             ||||||||||||||||||||||||||||||||||||||||||||||||||||||||||||
Sbjct  241   CAAAAATTACGATTTGCCTTCTGGCTGATTAAGCAGATCAACAGCGAAAACTACGAGGGG  300

Query  301   CTCCGCTGGGTAGACAGGGACCACACTCAGTTTCGCATCCCCTGGAAACACACTTCCAGG  360
             ||||||||||||||||||||||||||||||||||||||||||||||||||||||||||||
Sbjct  301   CTCCGCTGGGTAGACAGGGACCACACTCAGTTTCGCATCCCCTGGAAACACACTTCCAGG  360

Query  361   AAGGATATCAACCCTAACGATTACAAGATTTTCAAGGCCTGGGCTATAACCAGCGGAAAG  420
             ||||||||||||||||||||||||||||||||||||||||||||||||||||||||||||
Sbjct  361   AAGGATATCAACCCTAACGATTACAAGATTTTCAAGGCCTGGGCTATAACCAGCGGAAAG  420

Query  421   TACAACGAAAAGCTGAAAGATCTAGCCAAGTGGAAAACCAACTTCCGCTGTGCCATGAGA  480
             ||||||||||||||||||||||||||||||||||||||||||||||||||||||||||||
Sbjct  421   TACAACGAAAAGCTGAAAGATCTAGCCAAGTGGAAAACCAACTTCCGCTGTGCCATGAGA  480

Query  481   AGCACAAACATGTTCCTGATGGTGCAGGATAACTCAAAGACCTCTGAGGACCCTCACAAA  540
             ||||||||||||||||||||||||||||||||||||||||||||||||||||||||||||
Sbjct  481   AGCACAAACATGTTCCTGATGGTGCAGGATAACTCAAAGACCTCTGAGGACCCTCACAAA  540

Query  541   GTGTATCAATACATCAGCCCAAGCCCCGATGCTGTGGCAGCTGCTAATGCCCCGACTACT  600
             ||||||||||||||||||||||||||||||||||||||||||||||||||||||||||||
Sbjct  541   GTGTATCAATACATCAGCCCAAGCCCCGATGCTGTGGCAGCTGCTAATGCCCCGACTACT  600

Query  601   AAAAACAATAACCTGCGTGCCAGTAAAGAAGACATCAGCCCCCATTCTGAAGAAGCCCCT  660
             ||||||||||||||||||||||||||||||||||||||||||||||||||||||||||||
Sbjct  601   AAAAACAATAACCTGCGTGCCAGTAAAGAAGACATCAGCCCCCATTCTGAAGAAGCCCCT  660

Query  661   CATCTGCACCACCATATGCCACAGCAACTGCAGCCGCAGATAGAATTAGATTTGGAGATG  720
             ||||||||||||||||||||||||||||||||||||||||||||||||||||||||||||
Sbjct  661   CATCTGCACCACCATATGCCACAGCAACTGCAGCCGCAGATAGAATTAGATTTGGAGATG  720

Query  721   TTGTCGCTGGAAAACCCTCCCCAAGAGATGGATAGCGTGCAGAACGAAAGGCCAGTCTAC  780
             ||||||||||||||||||||||||||||||||||||||||||||||||||||||||||||
Sbjct  721   TTGTCGCTGGAAAACCCTCCCCAAGAGATGGATAGCGTGCAGAACGAAAGGCCAGTCTAC  780

Query  781   GACCCTGGCAGGAATCCTCCCCAGGACTACACCACCAATAATTCGGCCCCAAATGGCTAC  840
             ||||||||||||||||||||||||||||||||||||||||||||||||||||||||||||
Sbjct  781   GACCCTGGCAGGAATCCTCCCCAGGACTACACCACCAATAATTCGGCCCCAAATGGCTAC  840

Query  841   CAGTGCAGCCCCAACACACTCCACTGGATTCTGCAGCAGTGCAACTTAACCCAGAATGGC  900
             ||||||||||||||||||||||||||||||||||||||||||||||||||||||||||||
Sbjct  841   CAGTGCAGCCCCAACACACTCCACTGGATTCTGCAGCAGTGCAACTTAACCCAGAATGGC  900

Query  901   TGCAGCCCACAGCAGCTGCCGTGGGCTCTGGAGGGAGGTTTAGATCACGGTTCAGGCTAT  960
             ||||||||||||||||||||||||||||||||||||||||||||||||||||||||||||
Sbjct  901   TGCAGCCCACAGCAGCTGCCGTGGGCTCTGGAGGGAGGTTTAGATCACGGTTCAGGCTAT  960

Query  961   CCAAACCAACATATTGGCCAAAATAGCTACCCACAGCAGGGGAGCGGGGTGGCAAATGGA  1020
             ||||||||||||||||||||||||||||||||||||||||||||||||||||||||||||
Sbjct  961   CCAAACCAACATATTGGCCAAAATAGCTACCCACAGCAGGGGAGCGGGGTGGCAAATGGA  1020

Query  1021  CCAGTGGAGGACTATCACCAATCAATTGGCCAGTGGGTGACCCAAACCATGGCAGAGACC  1080
             ||||||||||||||||||||||||||||||||||||||||||||||||||||||||||||
Sbjct  1021  CCAGTGGAGGACTATCACCAATCAATTGGCCAGTGGGTGACCCAAACCATGGCAGAGACC  1080

Query  1081  CAAACCATGCAAAATGCTTATGGCCCTTCTGCTGCCTCACTTCTCCAGCAGCCCCTCCCA  1140
             ||||||||||||||||||||||||||||||||||||||||||||||||||||||||||||
Sbjct  1081  CAAACCATGCAAAATGCTTATGGCCCTTCTGCTGCCTCACTTCTCCAGCAGCCCCTCCCA  1140

Query  1141  TCCAGCACAGTTCCTGGCACGAGTCAGCTTCTTAATAACGCAGCGGAAGTCCTGTTTGAG  1200
             |||||||||||||||||||||||||||||||||||||||||      |||||||||||||
Sbjct  1141  TCCAGCACAGTTCCTGGCACGAGTCAGCTTCTTAATAACGC------AGTCCTGTTTGAG  1194

Query  1201  GGGAGACCTCACCTCAATAACCAAAGCACTTTCCTGAGTGAAGCACCTGCAGGAAACGCA  1260
             ||||||||||||||||||||||||||||||||||||||||||||||||||||||||||||
Sbjct  1195  GGGAGACCTCACCTCAATAACCAAAGCACTTTCCTGAGTGAAGCACCTGCAGGAAACGCA  1254

Query  1261  CTGATCTATCCAACACTGAATGGCCCGGAAGAGAACAGTTATCACCAGCTGGCAAATCAG  1320
             ||||||||||||||||||||||||||||||||||||||||||||||||||||||||||||
Sbjct  1255  CTGATCTATCCAACACTGAATGGCCCGGAAGAGAACAGTTATCACCAGCTGGCAAATCAG  1314

Query  1321  TGGGCAGCCCCAGCAGTGGTGGAGCAGAACGGGTTCACCCAGCCTGCTGCCCCTCTCCCA  1380
             ||||||||||||||||||||||||||||||||||||||||||||||||||||||||||||
Sbjct  1315  TGGGCAGCCCCAGCAGTGGTGGAGCAGAACGGGTTCACCCAGCCTGCTGCCCCTCTCCCA  1374

Query  1381  GAGCAGCATCCTCCTCCCCACACAGCGCCATCGCGGAACAATACAGAAACCATTCCTCCC  1440
             ||||||||||||||||||||||||||||||||||||||||||||||||||||||||||||
Sbjct  1375  GAGCAGCATCCTCCTCCCCACACAGCGCCATCGCGGAACAATACAGAAACCATTCCTCCC  1434

Query  1441  AATATGGACGTCACCATCTATTACCGAGGGAAGCCACTCCACGAGGTGCAGGTGGCCACC  1500
             ||||||||||||||||||||||||||||||||||||||||||||||||||||||||||||
Sbjct  1435  AATATGGACGTCACCATCTATTACCGAGGGAAGCCACTCCACGAGGTGCAGGTGGCCACC  1494

Query  1501  AGCAGCTTCCTGTTCACGTACAATAATGAAGACACCACCGTTGTCCCGGGCTGCGCGCAG  1560
             ||||||||||||||||||||||||||||||||||||||||||||||||||||||||||||
Sbjct  1495  AGCAGCTTCCTGTTCACGTACAATAATGAAGACACCACCGTTGTCCCGGGCTGCGCGCAG  1554

Query  1561  GTGGTCCAATTCCCAAGCCCAGATGAGCTGCCTGACCAGAAACAGGTTCAGTTCACCAGG  1620
             ||||||||||||||||||||||||||||||||||||||||||||||||||||||||||||
Sbjct  1555  GTGGTCCAATTCCCAAGCCCAGATGAGCTGCCTGACCAGAAACAGGTTCAGTTCACCAGG  1614

Query  1621  CAGCTGCTCCTGAACACAGGGCTGCTGCTGGAGCAGAAGCACAAGAAGATCTATGCCAAG  1680
             ||||||||||||||||||||||||||||||||||||||||||||||||||||||||||||
Sbjct  1615  CAGCTGCTCCTGAACACAGGGCTGCTGCTGGAGCAGAAGCACAAGAAGATCTATGCCAAG  1674

Query  1681  CGCCTGAATAAATGCAAGGTCTTCTGGGCCCTCTCCAGGCAACTGGCGAACATGGCTCAG  1740
             ||||||||||||||||||||||||||||||||||||||||||||||||||||||||||||
Sbjct  1675  CGCCTGAATAAATGCAAGGTCTTCTGGGCCCTCTCCAGGCAACTGGCGAACATGGCTCAG  1734

Query  1741  AACCCTGAGCCCAAGCTGCTGCCACGTGGCACAGAGACGGAGATCTTCGACTATGAGGTG  1800
             ||||||||||||||||||||||||||||||||||||||||||||||||||||||||||||
Sbjct  1735  AACCCTGAGCCCAAGCTGCTGCCACGTGGCACAGAGACGGAGATCTTCGACTATGAGGTG  1794

Query  1801  TTCTCAAAGGAGCTGAAGGATTTTAAGGAACATCAAAGGGCTGCATCTCCCGACTTCACC  1860
             ||||||||||||||||||||||||||||||||||||||||||||||||||||||||||||
Sbjct  1795  TTCTCAAAGGAGCTGAAGGATTTTAAGGAACATCAAAGGGCTGCATCTCCCGACTTCACC  1854

Query  1861  ATCTACCTTTGCTTTGGGCAGTGCTTCTCCAAATCCAAGCCCAAGGAGACCATGTTAATC  1920
             ||||||||||||||||||||||||||||||||||||||||||||||||||||||||||||
Sbjct  1855  ATCTACCTTTGCTTTGGGCAGTGCTTCTCCAAATCCAAGCCCAAGGAGACCATGTTAATC  1914

Query  1921  CTGGTGAAGCTGGTGCCTAAGTTCTGTGAGTATTTCCACGACATGGTGCTGCGGGAGGGA  1980
             ||||||||||||||||||||||||||||||||||||||||||||||||||||||||||||
Sbjct  1915  CTGGTGAAGCTGGTGCCTAAGTTCTGTGAGTATTTCCACGACATGGTGCTGCGGGAGGGA  1974

Query  1981  GCCTCGTCCCTGAACAGCGGGAACGTGAGCCTCCAGCTGTCCAGCTGCTTCAACAGCTTG  2040
             ||||||||||||||||||||||||||||||||||||||||||||||||||||||||||||
Sbjct  1975  GCCTCGTCCCTGAACAGCGGGAACGTGAGCCTCCAGCTGTCCAGCTGCTTCAACAGCTTG  2034

Query  2041  TATGACCTCATTGAGCAGTTCAACATGCAGATTGACTGAACCTTCTCCCCTCCCTAGGAC  2100
             ||||||||||||||||||||||||||||||||||||||||||||||||||||||||||||
Sbjct  2035  TATGACCTCATTGAGCAGTTCAACATGCAGATTGACTGAACCTTCTCCCCTCCCTAGGAC  2094

Query  2101  CCGATCTGCATGTGCATTGGCCAGTCCTTGTCTAGGTCTCTGCCTGACTGGCCTGCCTTT  2160
             ||||||||||||||||||||||||||||||||||||||||||||||||||||||||||||
Sbjct  2095  CCGATCTGCATGTGCATTGGCCAGTCCTTGTCTAGGTCTCTGCCTGACTGGCCTGCCTTT  2154

Query  2161  GAAAACACTTCTGCCTTTCCCTCACCCACTTGCATCAGTTTGCTCCCATCAGAAAACATG  2220
             ||||||||||||||||||||||||||||||||||||||||||||||||||||||||||||
Sbjct  2155  GAAAACACTTCTGCCTTTCCCTCACCCACTTGCATCAGTTTGCTCCCATCAGAAAACATG  2214

Query  2221  CGGGTGAGATGATGGGTGGCCTTGTGGCTTAGCCACATCACTGGGACTCAGTAAAGCTGG  2280
             ||||||||||||||||||||||||||||||||||||||||||||||||||||||||||||
Sbjct  2215  CGGGTGAGATGATGGGTGGCCTTGTGGCTTAGCCACATCACTGGGACTCAGTAAAGCTGG  2274

Query  2281  GTTCAATCTCTGCTGTGCCACTGGCCTTCTTAGTGACCTTGGGCAAGTCACTGCATCTGG  2340
             ||||||||||||||||||||||||||||||||||||||||||||||||||||||||||||
Sbjct  2275  GTTCAATCTCTGCTGTGCCACTGGCCTTCTTAGTGACCTTGGGCAAGTCACTGCATCTGG  2334

Query  2341  GCCTCACTTTCCCCATCTGTAAAATGGGAATAATTCCTTACCTCACAGGGTTGTTGTGAG  2400
             ||||||||||||||||||||||||||||||||||||||||||||||||||||||||||||
Sbjct  2335  GCCTCACTTTCCCCATCTGTAAAATGGGAATAATTCCTTACCTCACAGGGTTGTTGTGAG  2394

Query  2401  GAGAAATGCATGTGAAGTGCTCAGATATTATGGCAATAGACAACTCAGCCTTCCCCACTC  2460
             ||||||||||||||||||||||||||||||||||||||||||||||||||||||||||||
Sbjct  2395  GAGAAATGCATGTGAAGTGCTCAGATATTATGGCAATAGACAACTCAGCCTTCCCCACTC  2454

Query  2461  TGGGCCACCAATTGAAAATAGAGAttttttttCCCCCCAGCTGAAACAAACAGACTACCA  2520
             ||||||||||||||||||||||||||||||||||||||||||||||||||||||||||||
Sbjct  2455  TGGGCCACCAATTGAAAATAGAGATTTTTTTTCCCCCCAGCTGAAACAAACAGACTACCA  2514

Query  2521  GACACTGTGCTCTGAAGGTAGAAGCAAGAGAATCTTCCTCTGTTTCAGGATGTCAGCAAG  2580
             ||||||||||||||||||||||||||||||||||||||||||||||||||||||||||||
Sbjct  2515  GACACTGTGCTCTGAAGGTAGAAGCAAGAGAATCTTCCTCTGTTTCAGGATGTCAGCAAG  2574

Query  2581  AAAGTCAGGGTGTGGGGGTGTGGGGGAAGAGGAGGCAGGAAGGGGAATACTCTTCTACAG  2640
             ||||||||||||||||||||||||||||||||||||||||||||||||||||||||||||
Sbjct  2575  AAAGTCAGGGTGTGGGGGTGTGGGGGAAGAGGAGGCAGGAAGGGGAATACTCTTCTACAG  2634

Query  2641  CACTGCCATGTAATGATCTTGTGGAGCTGGCTGGATTTTAAGCTGGGTGAGGGttttttt  2700
             ||||||||||||||||||||||||||||||||||||||||||||||||||||||||||||
Sbjct  2635  CACTGCCATGTAATGATCTTGTGGAGCTGGCTGGATTTTAAGCTGGGTGAGGGTTTTTTT  2694

Query  2701  tttttttttttAAGAAATGGGGATGTTGACGCTgggggcgggggggAATCCTTACCACCC  2760
             ||||||||||||||||||||||||||||||||||||||||||||||||||||||||||||
Sbjct  2695  TTTTTTTTTTTAAGAAATGGGGATGTTGACGCTGGGGGCGGGGGGGAATCCTTACCACCC  2754

Query  2761  GTTGCTGAAGTGAGCGAAGCAGGAACCCTTGGGGTGGATTGTACAATAGCCCTGGCCAGC  2820
             ||||||||||||||||||||||||||||||||||||||||||||||||||||||||||||
Sbjct  2755  GTTGCTGAAGTGAGCGAAGCAGGAACCCTTGGGGTGGATTGTACAATAGCCCTGGCCAGC  2814

Query  2821  TCTCCTGGGATCACAGGGTTAGTACAAGGTGAGCTAATCTGTCGGACAGAGGCTAAGGCC  2880
             ||||||||||||||||||||||||||||||||||||||||||||||||||||||||||||
Sbjct  2815  TCTCCTGGGATCACAGGGTTAGTACAAGGTGAGCTAATCTGTCGGACAGAGGCTAAGGCC  2874

Query  2881  CAGCTCCTAACGTCCGTTGATTTGAATGGAAGTTCAGAGCCTAAATACTTGGGGAGCTGG  2940
             ||||||||||||||||||||||||||||||||||||||||||||||||||||||||||||
Sbjct  2875  CAGCTCCTAACGTCCGTTGATTTGAATGGAAGTTCAGAGCCTAAATACTTGGGGAGCTGG  2934

Query  2941  GCTTTAATCAGTTGATAAACCATTATGGAAAAATCTCCACCCAGGACTAGGAACAGGGGA  3000
             ||||||||||||||||||||||||||||||||||||||||||||||||||||||||||||
Sbjct  2935  GCTTTAATCAGTTGATAAACCATTATGGAAAAATCTCCACCCAGGACTAGGAACAGGGGA  2994

Query  3001  GGGACCCCTATAGAAGAGAGAGACTCCATGTTCTCCCCAGGCTCTGTCCCTTTCAAGCGC  3060
             ||||||||||||||||||||||||||||||||||||||||||||||||||||||||||||
Sbjct  2995  GGGACCCCTATAGAAGAGAGAGACTCCATGTTCTCCCCAGGCTCTGTCCCTTTCAAGCGC  3054

Query  3061  TCGCCTGTCAGAGGTCTGTGAAATGGAAGCACCCATGGTCACGTCGATCTGCGAGTGACT  3120
             ||||||||||||||||||||||||||||||||||||||||||||||||||||||||||||
Sbjct  3055  TCGCCTGTCAGAGGTCTGTGAAATGGAAGCACCCATGGTCACGTCGATCTGCGAGTGACT  3114

Query  3121  GCAGCTGTGTGCAAGGAGTGTACAGAAGGCTGAAGTAAGCAAGCTCTAGGAGGGCTGCTT  3180
             ||||||||||||||||||||||||||||||||||||||||||||||||||||||||||||
Sbjct  3115  GCAGCTGTGTGCAAGGAGTGTACAGAAGGCTGAAGTAAGCAAGCTCTAGGAGGGCTGCTT  3174

Query  3181  TAAACAGCTGGCCCATGCCTCAAGCCCTCCCCTTACCACAATGCGAGGAGAGGGGAAAGA  3240
             ||||||||||||||||||||||||||||||||||||||||||||||||||||||||||||
Sbjct  3175  TAAACAGCTGGCCCATGCCTCAAGCCCTCCCCTTACCACAATGCGAGGAGAGGGGAAAGA  3234

Query  3241  AGGAGAATGAGTAACACCCTGAACAGTGTAGCAGAGGGGAGGCCCGGAAGCCACCACAGA  3300
             ||||||||||||||||||||||||||||||||||||||||||||||||||||||||||||
Sbjct  3235  AGGAGAATGAGTAACACCCTGAACAGTGTAGCAGAGGGGAGGCCCGGAAGCCACCACAGA  3294

Query  3301  GGCTGGTCCTGCTCCCAATGGAATTTTGCCATTGACTTTCCTGGGAGCAGGATCAGTCCT  3360
             ||||||||||||||||||||||||||||||||||||||||||||||||||||||||||||
Sbjct  3295  GGCTGGTCCTGCTCCCAATGGAATTTTGCCATTGACTTTCCTGGGAGCAGGATCAGTCCT  3354

Query  3361  ATTCCAAAGTAGCTTGCGCCCCCAAATGTGGCTGGCTAATAAATTAAAGTTTATAGCTCA  3420
             ||||||||||||||||||||||||||||||||||||||||||||||||||||||||||||
Sbjct  3355  ATTCCAAAGTAGCTTGCGCCCCCAAATGTGGCTGGCTAATAAATTAAAGTTTATAGCTCA  3414

Query  3421  ACCCTGTATTTTAACCTCTCCACACTCTGCCTGAGGTCAGTCTGCAGGCCCAGCTGCGCT  3480
             ||||||||||||||||||||||||||||||||||||||||||||||||||||||||||||
Sbjct  3415  ACCCTGTATTTTAACCTCTCCACACTCTGCCTGAGGTCAGTCTGCAGGCCCAGCTGCGCT  3474

Query  3481  CAGGCCTTGTGCATGTTGTATGAGCCCATGCGCCCTACAACTCCTTGTCATCCTTGAGCA  3540
             ||||||||||||||||||||||||||||||||||||||||||||||||||||||||||||
Sbjct  3475  CAGGCCTTGTGCATGTTGTATGAGCCCATGCGCCCTACAACTCCTTGTCATCCTTGAGCA  3534

Query  3541  CGGAGCTGCATCTGCCCGAACCAAGTCACACCGACAACACCCCACATCATTCTTTGCCAG  3600
             ||||||||||||||||||||||||||||||||||||||||||||||||||||||||||||
Sbjct  3535  CGGAGCTGCATCTGCCCGAACCAAGTCACACCGACAACACCCCACATCATTCTTTGCCAG  3594

Query  3601  CTTCTTGCCCCATAAAGCACATTCACACAAGAGAAATGCACTCCATGTTGATGTAGGATA  3660
             ||||||||||||||||||||||||||||||||||||||||||||||||||||||||||||
Sbjct  3595  CTTCTTGCCCCATAAAGCACATTCACACAAGAGAAATGCACTCCATGTTGATGTAGGATA  3654

Query  3661  ATCCACCATACCGCCTCCCTATCCCTTTTTCTGTCCCACTTCCCTCGGACACCACAGCAC  3720
             ||||||||||||||||||||||||||||||||||||||||||||||||||||||||||||
Sbjct  3655  ATCCACCATACCGCCTCCCTATCCCTTTTTCTGTCCCACTTCCCTCGGACACCACAGCAC  3714

Query  3721  GCACGTGCTGGCTTGCAGTCATCTGATGAGCACACAGCATATATAGGCACAGATCCATAA  3780
             ||||||||||||||||||||||||||||||||||||||||||||||||||||||||||||
Sbjct  3715  GCACGTGCTGGCTTGCAGTCATCTGATGAGCACACAGCATATATAGGCACAGATCCATAA  3774

Query  3781  AGCTGGCACTTTCCTAACCATACATGCTCAGGGTAACCAGTTGTTTTAATGGGCATAGGC  3840
             ||||||||||||||||||||||||||||||||||||||||||||||||||||||||||||
Sbjct  3775  AGCTGGCACTTTCCTAACCATACATGCTCAGGGTAACCAGTTGTTTTAATGGGCATAGGC  3834

Query  3841  CAGCAATTCTTAAACTATGGTTCACGCTTTAtttttttAACTGCAGCGTAGCCAAAATCT  3900
             ||||||||||||||||||||||||||||||||||||||||||||||||||||||||||||
Sbjct  3835  CAGCAATTCTTAAACTATGGTTCACGCTTTATTTTTTTAACTGCAGCGTAGCCAAAATCT  3894

Query  3901  CAATTCATTAATTGCCTTTCAAACAGCTGCACAGGCACTAAGTACCGCCGGTGCTTTCCC  3960
             ||||||||||||||||||||||||||||||||||||||||||||||||||||||||||||
Sbjct  3895  CAATTCATTAATTGCCTTTCAAACAGCTGCACAGGCACTAAGTACCGCCGGTGCTTTCCC  3954

Query  3961  CATGTTAACAATTGCGCTAGTTGCCACCATGATGTTGTGCAATCACAGCAACGAAGCCTG  4020
             ||||||||||||||||||||||||||||||||||||||||||||||||||||||||||||
Sbjct  3955  CATGTTAACAATTGCGCTAGTTGCCACCATGATGTTGTGCAATCACAGCAACGAAGCCTG  4014

Query  4021  TGCAGCGGGATGGGTCATAAGACATTAGGTCATGACCAACACTGGATCAGTATGGGAAGG  4080
             ||||||||||||||||||||||||||||||||||||||||||||||||||||||||||||
Sbjct  4015  TGCAGCGGGATGGGTCATAAGACATTAGGTCATGACCAACACTGGATCAGTATGGGAAGG  4074

Query  4081  CCTACTGCTGTAGGCTACTGTTACGCAAGCAGTTTGGACTCTGACAGGAGACCTGTATGA  4140
             ||||||||||||||||||||||||||||||||||||||||||||||||||||||||||||
Sbjct  4075  CCTACTGCTGTAGGCTACTGTTACGCAAGCAGTTTGGACTCTGACAGGAGACCTGTATGA  4134

Query  4141  AACTGACATCACACACTGGGCATTATTTTTTCCTCCAAAAGCAACTACAGATGTTAGCTT  4200
             ||||||||||||||||||||||||||||||||||||||||||||||||||||||||||||
Sbjct  4135  AACTGACATCACACACTGGGCATTATTTTTTCCTCCAAAAGCAACTACAGATGTTAGCTT  4194

Query  4201  TAGGACCCTTCCATGTCCTAGCTTAGCTCTGTTATTGCTAAGACACCTGAGTATCACCAT  4260
             ||||||||||||||||||||||||||||||||||||||||||||||||||||||||||||
Sbjct  4195  TAGGACCCTTCCATGTCCTAGCTTAGCTCTGTTATTGCTAAGACACCTGAGTATCACCAT  4254

Query  4261  GGTATCTGAGCACTGAGCATCTGTGGAAAGCAGCAAGAGGGAGATGACGAGTGTATCTTC  4320
             ||||||||||||||||||||||||||||||||||||||||||||||||||||||||||||
Sbjct  4255  GGTATCTGAGCACTGAGCATCTGTGGAAAGCAGCAAGAGGGAGATGACGAGTGTATCTTC  4314

Query  4321  CTCTTTTTGCCACCCCACAATTCTGAGCTGTCTATATTTATTGTGTTAACGCGGTTCTGG  4380
             ||||||||||||||||||||||||||||||||||||||||||||||||||||||||||||
Sbjct  4315  CTCTTTTTGCCACCCCACAATTCTGAGCTGTCTATATTTATTGTGTTAACGCGGTTCTGG  4374

Query  4381  GTAGTTTCAATTATATTAATTTTAAATTTCTTGCATTCCATGAGCTTAGTAACTGAGGCA  4440
             ||||||||||||||||||||||||||||||||||||||||||||||||||||||||||||
Sbjct  4375  GTAGTTTCAATTATATTAATTTTAAATTTCTTGCATTCCATGAGCTTAGTAACTGAGGCA  4434

Query  4441  GTAAATGAGTGCCAATTCGCTTGGTCATTGCAATGTGTGTGTGTATTacacacacacacG  4500
             ||||||||||||||||||||||||||||||||||||||||||||||||||||||||||||
Sbjct  4435  GTAAATGAGTGCCAATTCGCTTGGTCATTGCAATGTGTGTGTGTATTACACACACACACG  4494

Query  4501  AAGGTTGGCATCCACACCAGCAGCTCTGATTGCTTCCTCCTTGTCCTTTAAAGGCTGCTT  4560
             ||||||||||||||||||||||||||||||||||||||||||||||||||||||||||||
Sbjct  4495  AAGGTTGGCATCCACACCAGCAGCTCTGATTGCTTCCTCCTTGTCCTTTAAAGGCTGCTT  4554

Query  4561  AGATCTTTATACATGTCATCCTGACTGAGCTGTGAGGAACAAAAATGCAAGTGTCTCAAG  4620
             ||||||||||||||||||||||||||||||||||||||||||||||||||||||||||||
Sbjct  4555  AGATCTTTATACATGTCATCCTGACTGAGCTGTGAGGAACAAAAATGCAAGTGTCTCAAG  4614

Query  4621  GTCTGATGGCTAAAAGTTTGATTTAAATAGAAAAATGAATAGATTCCAAGAAGTCTCATC  4680
             ||||||||||||||||||||||||||||||||||||||||||||||||||||||||||||
Sbjct  4615  GTCTGATGGCTAAAAGTTTGATTTAAATAGAAAAATGAATAGATTCCAAGAAGTCTCATC  4674

Query  4681  CTCTGCCCTTCCTTTGACCAGCCAAGGGCATTTTAAGAAAAAGGGGTGGGGGATAGAAAT  4740
             ||||||||||||||||||||||||||||||||||||||||||||||||||||||||||||
Sbjct  4675  CTCTGCCCTTCCTTTGACCAGCCAAGGGCATTTTAAGAAAAAGGGGTGGGGGATAGAAAT  4734

Query  4741  CAGGGAAGCAGGTCTTCCCAAACCTATGCACTGATGGAGCCCAACATGCCTTACAAAGGG  4800
             ||||||||||||||||||||||||||||||||||||||||||||||||||||||||||||
Sbjct  4735  CAGGGAAGCAGGTCTTCCCAAACCTATGCACTGATGGAGCCCAACATGCCTTACAAAGGG  4794

Query  4801  TGAGTGAAATCCTGGCCCTGCTGAATCAATGGGCGTCCTGCCATTGACATCAGAGGGGCC  4860
             ||||||||||||||||||||||||||||||||||||||||||||||||||||||||||||
Sbjct  4795  TGAGTGAAATCCTGGCCCTGCTGAATCAATGGGCGTCCTGCCATTGACATCAGAGGGGCC  4854

Query  4861  TGGATTTCACCTGGTGTGTCTGGTAGAATGTTAAGCACTAATATTAAAGTATAATTTATA  4920
             ||||||||||||||||||||||||||||||||||||||||||||||||||||||||||||
Sbjct  4855  TGGATTTCACCTGGTGTGTCTGGTAGAATGTTAAGCACTAATATTAAAGTATAATTTATA  4914

Query  4921  GAACACATGCCCCGCTGTGGTACTTTCCCTAGGTTTTCTTTATTGCTTAGGTTGCATAAG  4980
             ||||||||||||||||||||||||||||||||||||||||||||||||||||||||||||
Sbjct  4915  GAACACATGCCCCGCTGTGGTACTTTCCCTAGGTTTTCTTTATTGCTTAGGTTGCATAAG  4974

Query  4981  TAAGTTGTGCATGTTTGGTTTTTAGCTTCCTTTTATTAGCACGGGGGCTCTGGGTTTCAT  5040
             ||||||||||||||||||||||||||||||||||||||||||||||||||||||||||||
Sbjct  4975  TAAGTTGTGCATGTTTGGTTTTTAGCTTCCTTTTATTAGCACGGGGGCTCTGGGTTTCAT  5034

Query  5041  CCAAATGGATTTTGACTTAAATTTTCAAAAGTGATTTTGGGGGAAATTTTTTAAAAGGGC  5100
             ||||||||||||||||||||||||||||||||||||||||||||||||||||||||||||
Sbjct  5035  CCAAATGGATTTTGACTTAAATTTTCAAAAGTGATTTTGGGGGAAATTTTTTAAAAGGGC  5094

Query  5101  CTGACTGTCAGAACATGCAGAGCACCTGCTCTCTCAAAGGCTGTCAAGCCAGCACCCACA  5160
             ||||||||||||||||||||||||||||||||||||||||||||||||||||||||||||
Sbjct  5095  CTGACTGTCAGAACATGCAGAGCACCTGCTCTCTCAAAGGCTGTCAAGCCAGCACCCACA  5154

Query  5161  AACTGAGCCCACCCCAcccccccACAAATCACTAGTAAATGGGTATGTGCACAACTGCCT  5220
             ||||||||||||||||||||||||||||||||||||||||||||||||||||||||||||
Sbjct  5155  AACTGAGCCCACCCCACCCCCCCACAAATCACTAGTAAATGGGTATGTGCACAACTGCCT  5214

Query  5221  GCTCCTAGGTGAAATGTCACCTGTTTGACAGGTTCCCATCAGGGGTTAACCTAGATGACC  5280
             ||||||||||||||||||||||||||||||||||||||||||||||||||||||||||||
Sbjct  5215  GCTCCTAGGTGAAATGTCACCTGTTTGACAGGTTCCCATCAGGGGTTAACCTAGATGACC  5274

Query  5281  AGCACCTGAGCAGCCGGAGAGGCCTTTGGCTTTGCAGCTGTACTGCAACTTCCAAATAGA  5340
             ||||||||||||||||||||||||||||||||||||||||||||||||||||||||||||
Sbjct  5275  AGCACCTGAGCAGCCGGAGAGGCCTTTGGCTTTGCAGCTGTACTGCAACTTCCAAATAGA  5334

Query  5341  AGCAAATCCCTTTACATGGGCTAGGGTTTAATTCAGTTGGTGTCAGTCCAGCCCTCTCCC  5400
             ||||||||||||||||||||||||||||||||||||||||||||||||||||||||||||
Sbjct  5335  AGCAAATCCCTTTACATGGGCTAGGGTTTAATTCAGTTGGTGTCAGTCCAGCCCTCTCCC  5394

Query  5401  GGCCAGCCTGAGGCTGGAGAGGGGCTCTCCTCCTGACATCTTGTACTGGAGTTCATGTTT  5460
             ||||||||||||||||||||||||||||||||||||||||||||||||||||||||||||
Sbjct  5395  GGCCAGCCTGAGGCTGGAGAGGGGCTCTCCTCCTGACATCTTGTACTGGAGTTCATGTTT  5454

Query  5461  ACACAAGGGGGTTGGTCTTTGCTGCTGCTGGCCTCACAGAAGTAGTTCCAGCACTAGCAG  5520
             ||||||||||||||||||||||||||||||||||||||||||||||||||||||||||||
Sbjct  5455  ACACAAGGGGGTTGGTCTTTGCTGCTGCTGGCCTCACAGAAGTAGTTCCAGCACTAGCAG  5514

Query  5521  AAAAGCTTTAGGGCTCTGATCTGGCTCCAGTTAAATTCAGTGGCCAAAGTGTGTGTTTTC  5580
             ||||||||||||||||||||||||||||||||||||||||||||||||||||||||||||
Sbjct  5515  AAAAGCTTTAGGGCTCTGATCTGGCTCCAGTTAAATTCAGTGGCCAAAGTGTGTGTTTTC  5574

Query  5581  AGGGGGAGGTGAATTAGCCTCAAGAGGCTGCTTGCCTATAGCATGAGGAGGAGATGGACA  5640
             ||||||||||||||||||||||||||||||||||||||||||||||||||||||||||||
Sbjct  5575  AGGGGGAGGTGAATTAGCCTCAAGAGGCTGCTTGCCTATAGCATGAGGAGGAGATGGACA  5634

Query  5641  AGGGCAAGAGAAGGGATTggggggcagggggAAGACATGAAAGCAGCAGGTCAAGGTGAG  5700
             ||||||||||||||||||||||||||||||||||||||||||||||||||||||||||||
Sbjct  5635  AGGGCAAGAGAAGGGATTGGGGGGCAGGGGGAAGACATGAAAGCAGCAGGTCAAGGTGAG  5694

Query  5701  CAGGGGTGAAAAGAGCCCCACACACAACTGGCCCCAAGCCCCTTTTCCCTCTTCTCTATA  5760
             ||||||||||||||||||||||||||||||||||||||||||||||||||||||||||||
Sbjct  5695  CAGGGGTGAAAAGAGCCCCACACACAACTGGCCCCAAGCCCCTTTTCCCTCTTCTCTATA  5754

Query  5761  GAGCAGCAGCAGATCTGGTGCCTCCCAGAGCTGTCTTCTAGCTCAGGTGTTGAAGACTTT  5820
             ||||||||||||||||||||||||||||||||||||||||||||||||||||||||||||
Sbjct  5755  GAGCAGCAGCAGATCTGGTGCCTCCCAGAGCTGTCTTCTAGCTCAGGTGTTGAAGACTTT  5814

Query  5821  AAGTAACATTTGACTTTATTAATATGCCTTTTTCTAATGTCATGTCTCATGCAGCCCCAG  5880
             ||||||||||||||||||||||||||||||||||||||||||||||||||||||||||||
Sbjct  5815  AAGTAACATTTGACTTTATTAATATGCCTTTTTCTAATGTCATGTCTCATGCAGCCCCAG  5874

Query  5881  GGTGTTTGCACTCTCTGTAGAGCAGGCATTGCATTATCTGGGAGAGGATTGCACAGCCTG  5940
             ||||||||||||||||||||||||||||||||||||||||||||||||||||||||||||
Sbjct  5875  GGTGTTTGCACTCTCTGTAGAGCAGGCATTGCATTATCTGGGAGAGGATTGCACAGCCTG  5934

Query  5941  AGAACGCTCCCTGGTTAAGGAGATGGTAGCCTGACACGGCGTAACTTGTGTAGCAATGTT  6000
             ||||||||||||||||||||||||||||||||||||||||||||||||||||||||||||
Sbjct  5935  AGAACGCTCCCTGGTTAAGGAGATGGTAGCCTGACACGGCGTAACTTGTGTAGCAATGTT  5994

Query  6001  AGTGCAGTGTGTGGGTGGGATGGTTGAGTCACAAGCAGTGTCGTGGATTTTATGGAGCGT  6060
             ||||||||||||||||||||||||||||||||||||||||||||||||||||||||||||
Sbjct  5995  AGTGCAGTGTGTGGGTGGGATGGTTGAGTCACAAGCAGTGTCGTGGATTTTATGGAGCGT  6054

Query  6061  GTCTAGACATTGCAAAGGAGTCAGAGTCTCGCTCATGTGAGCATGTGTAGCTCCAGCCAA  6120
             ||||||||||||||||||||||||||||||||||||||||||||||||||||||||||||
Sbjct  6055  GTCTAGACATTGCAAAGGAGTCAGAGTCTCGCTCATGTGAGCATGTGTAGCTCCAGCCAA  6114

Query  6121  GTCAATGCAGCTCCAGAGCCGGCCCACTGAATTTAATTTAGGCAGAGAGCGAAACATCTG  6180
             ||||||||||||||||||||||||||||||||||||||||||||||||||||||||||||
Sbjct  6115  GTCAATGCAGCTCCAGAGCCGGCCCACTGAATTTAATTTAGGCAGAGAGCGAAACATCTG  6174

Query  6181  GGTTCTCAAACTGGGGGTCGTGACCCCTCCGGGGGGTCACAAGATTATTTCGGGGGGCCG  6240
             ||||||||||||||||||||||||||||||||||||||||||||||||||||||||||||
Sbjct  6175  GGTTCTCAAACTGGGGGTCGTGACCCCTCCGGGGGGTCACAAGATTATTTCGGGGGGCCG  6234

Query  6241  TTGCGAGCTGTCAGCCTCACACATGTCGGGGCTCCAGCTATCAACCCCCAGGTGTGCTAG  6300
             ||||||||||||||||||||||||||||||||||||||||||||||||||||||||||||
Sbjct  6235  TTGCGAGCTGTCAGCCTCACACATGTCGGGGCTCCAGCTATCAACCCCCAGGTGTGCTAG  6294

Query  6301  GGCTCCGGCTGTCAGCCCACGCCCTTTACAGCTGGGTGGCTGGAGAGCAGGGGCTGCTGG  6360
             ||||||||||||||||||||||||||||||||||||||||||||||||||||||||||||
Sbjct  6295  GGCTCCGGCTGTCAGCCCACGCCCTTTACAGCTGGGTGGCTGGAGAGCAGGGGCTGCTGG  6354

Query  6361  CCAGGCTCGCAGCATTGAAGGCAGCAGCTGCGCAGAAGTAAGGGTGGCATAGTATGGTAA  6420
             ||||||||||||||||||||||||||||||||||||||||||||||||||||||||||||
Sbjct  6355  CCAGGCTCGCAGCATTGAAGGCAGCAGCTGCGCAGAAGTAAGGGTGGCATAGTATGGTAA  6414

Query  6421  GGAGGGGGGTCGTCACTTTTTTggggggggggggTCATCACAGTCTGAAATATTTTCAAA  6480
             ||||||||||||||||||||||||||||||||||||||||||||||||||||||||||||
Sbjct  6415  GGAGGGGGGTCGTCACTTTTTTGGGGGGGGGGGGTCATCACAGTCTGAAATATTTTCAAA  6474

Query  6481  GAGGGTCCCAGCaaaaaaaaGTTTGAGAATCCCTGAGCTTGAGAATGTGCTGGCAGGGAA  6540
             ||||||||||||||||||||||||||||||||||||||||||||||||||||||||||||
Sbjct  6475  GAGGGTCCCAGCAAAAAAAAGTTTGAGAATCCCTGAGCTTGAGAATGTGCTGGCAGGGAA  6534

Query  6541  CTGAGCAAGAGAccccaccccccccaccccATCTACAGTGCAGCTCCTATCCAAGCACAA  6600
             ||||||||||||||||||||||||||||||||||||||||||||||||||||||||||||
Sbjct  6535  CTGAGCAAGAGACCCCACCCCCCCCACCCCATCTACAGTGCAGCTCCTATCCAAGCACAA  6594

Query  6601  CTGTATGTCGGTGTTGGTCTGACTGCACATGCCATGCTGCATAGTCATGTTTGTAGCCGT  6660
             ||||||||||||||||||||||||||||||||||||||||||||||||||||||||||||
Sbjct  6595  CTGTATGTCGGTGTTGGTCTGACTGCACATGCCATGCTGCATAGTCATGTTTGTAGCCGT  6654

Query  6661  ACGTTATGGACAGCTATCCCACTCTGACTCAGCTGCTGGGGGAGAGGGGCAGAAGATTGT  6720
             ||||||||||||||||||||||||||||||||||||||||||||||||||||||||||||
Sbjct  6655  ACGTTATGGACAGCTATCCCACTCTGACTCAGCTGCTGGGGGAGAGGGGCAGAAGATTGT  6714

Query  6721  GGGTGTTTAATAAGGCCCAATGAGCTTTAGGGCAGGCTGACCTCCCACTCACACAGCGAG  6780
             ||||||||||||||||||||||||||||||||||||||||||||||||||||||||||||
Sbjct  6715  GGGTGTTTAATAAGGCCCAATGAGCTTTAGGGCAGGCTGACCTCCCACTCACACAGCGAG  6774

Query  6781  GAGGCAGGAGAACTGGCTGGCATTGCACACACGGTTAGGTGGTTATCTGCTGTAATGAGG  6840
             ||||||||||||||||||||||||||||||||||||||||||||||||||||||||||||
Sbjct  6775  GAGGCAGGAGAACTGGCTGGCATTGCACACACGGTTAGGTGGTTATCTGCTGTAATGAGG  6834

Query  6841  AAGGACCATTTAGAACCTAGTTACAGCCTAATAATGATGTTCATGAGAAAAATCAAAGCA  6900
             ||||||||||||||||||||||||||||||||||||||||||||||||||||||||||||
Sbjct  6835  AAGGACCATTTAGAACCTAGTTACAGCCTAATAATGATGTTCATGAGAAAAATCAAAGCA  6894

Query  6901  TGGTGTATCCAACATGATTACGTTGCCAACAGCGTGTGTGGACATGAACTGGGTAGGGAG  6960
             ||||||||||||||||||||||||||||||||||||||||||||||||||||||||||||
Sbjct  6895  TGGTGTATCCAACATGATTACGTTGCCAACAGCGTGTGTGGACATGAACTGGGTAGGGAG  6954

Query  6961  GCAGCCGCAGTACAAGCTGTACGGTAGAAGGGCCTCTCGCTATTGCCATGAAATGCCAAG  7020
             ||||||||||||||||||||||||||||||||||||||||||||||||||||||||||||
Sbjct  6955  GCAGCCGCAGTACAAGCTGTACGGTAGAAGGGCCTCTCGCTATTGCCATGAAATGCCAAG  7014

Query  7021  CTGATCCGAGTTGGGTGCTACAGATTATTCTGCCTTTTTAGTAACATGACCCTTGTGATT  7080
             ||||||||||||||||||||||||||||||||||||||||||||||||||||||||||||
Sbjct  7015  CTGATCCGAGTTGGGTGCTACAGATTATTCTGCCTTTTTAGTAACATGACCCTTGTGATT  7074

Query  7081  TGATTTTAAAATGCTTAACTCCCATCAATCACAGATTCAGGGGGGATTTATTGTGTTCTC  7140
             ||||||||||||||||||||||||||||||||||||||||||||||||||||||||||||
Sbjct  7075  TGATTTTAAAATGCTTAACTCCCATCAATCACAGATTCAGGGGGGATTTATTGTGTTCTC  7134

Query  7141  TCACACCCCAGTTGTGATCATGGGAGTTCCACATAAAGCAACACGTTAAAGCCTTTCATT  7200
             ||||||||||||||||||||||||||||||||||||||||||||||||||||||||||||
Sbjct  7135  TCACACCCCAGTTGTGATCATGGGAGTTCCACATAAAGCAACACGTTAAAGCCTTTCATT  7194

Query  7201  TTCCTTCTCTCCAAAACTTGGGCAGCAGAAGGAAGTAGGAAATAAAAGGACACTAATGAC  7260
             ||||||||||||||||||||||||||||||||||||||||||||||||||||||||||||
Sbjct  7195  TTCCTTCTCTCCAAAACTTGGGCAGCAGAAGGAAGTAGGAAATAAAAGGACACTAATGAC  7254

Query  7261  CAGCTTCTTCATGCTAAGTCAGAAAATCAAATTTCATGGCTCAGCATCTGCACTGCACCT  7320
             ||||||||||||||||||||||||||||||||||||||||||||||||||||||||||||
Sbjct  7255  CAGCTTCTTCATGCTAAGTCAGAAAATCAAATTTCATGGCTCAGCATCTGCACTGCACCT  7314

Query  7321  TTGGGAGGCAATCAATGTGGGTGTGTTAATTAAATATGGATTTTTGTAGTGTACTTTTTG  7380
             ||||||||||||||||||||||||||||||||||||||||||||||||||||||||||||
Sbjct  7315  TTGGGAGGCAATCAATGTGGGTGTGTTAATTAAATATGGATTTTTGTAGTGTACTTTTTG  7374

Query  7381  ATACTTGGTTAATTACTGCTCCTTCCCATATGATGGAATAAACGGGCTGCTTCCCCAGAG  7440
             ||||||||||||||||||||||||||||||||||||||||||||||||||||||||||||
Sbjct  7375  ATACTTGGTTAATTACTGCTCCTTCCCATATGATGGAATAAACGGGCTGCTTCCCCAGAG  7434

Query  7441  GTTGTAAACTGGCATAGCTCTCCTGACACAAATAATGCTTTCAGCAAATCCGAAGACCCG  7500
             ||||||||||||||||||||||||||||||||||||||||||||||||||||||||||||
Sbjct  7435  GTTGTAAACTGGCATAGCTCTCCTGACACAAATAATGCTTTCAGCAAATCCGAAGACCCG  7494

Query  7501  GAAGTTCTGGTTCTTCATTAACAGGCGTGAGGTGAATAATGTTTCTTAGTGAGGAAACAG  7560
             ||||||||||||||||||||||||||||||||||||||||||||||||||||||||||||
Sbjct  7495  GAAGTTCTGGTTCTTCATTAACAGGCGTGAGGTGAATAATGTTTCTTAGTGAGGAAACAG  7554

Query  7561  GGCCTTGATCCGAAGCTTAACAGGAGCCTTTCCAAAGGCTTTCACTGGGCTTTGGATCCA  7620
             ||||||||||||||||||||||||||||||||||||||||||||||||||||||||||||
Sbjct  7555  GGCCTTGATCCGAAGCTTAACAGGAGCCTTTCCAAAGGCTTTCACTGGGCTTTGGATCCA  7614

Query  7621  GCTGCTAATGTATCACAAAATCTGAAAGCTTCTTTCCATCTTGATTTAAGAAATGTTTCT  7680
             ||||||||||||||||||||||||||||||||||||||||||||||||||||||||||||
Sbjct  7615  GCTGCTAATGTATCACAAAATCTGAAAGCTTCTTTCCATCTTGATTTAAGAAATGTTTCT  7674

Query  7681  TAGAACCGTAACTGAGGTAAACTGTAATTCTGTTCTAACGCCAGGTCAGTTTTCATATTT  7740
             ||||||||||||||||||||||||||||||||||||||||||||||||||||||||||||
Sbjct  7675  TAGAACCGTAACTGAGGTAAACTGTAATTCTGTTCTAACGCCAGGTCAGTTTTCATATTT  7734

Query  7741  TGTGCCCATTTCTTGTGTGTATTCTGAGTAGTGCAAACCTTTCAGCTACTGATGTGAGCG  7800
             ||||||||||||||||||||||||||||||||||||||||||||||||||||||||||||
Sbjct  7735  TGTGCCCATTTCTTGTGTGTATTCTGAGTAGTGCAAACCTTTCAGCTACTGATGTGAGCG  7794

Query  7801  AAGCTCTCTGTTCACATTGCCTGTGCCGAAGCATAGATAAGTGCCCTTTTCCCCTCTTGG  7860
             ||||||||||||||||||||||||||||||||||||||||||||||||||||||||||||
Sbjct  7795  AAGCTCTCTGTTCACATTGCCTGTGCCGAAGCATAGATAAGTGCCCTTTTCCCCTCTTGG  7854

Query  7861  GTCTGTGTAAATAAACTGACATTCCACTGTC  7891
             |||||||||||||||||||||||||||||||
Sbjct  7855  GTCTGTGTAAATAAACTGACATTCCACTGTC  7885
```
