# Supplementary material for: Divergent IL18-STAT1 Immune Responses Underlie Differential Susceptibility to Aeromonas hydrophila in Geoclemys hamiltonii and Trachemys scripta: A Comparative Transcriptomic Perspective
Source: Genes (Basel). 2026 Apr 9;17(4):436. doi: 10.3390/genes17040436 (PMC13116093; doi:10.3390/genes17040436)
Supplement: Supplementary file 1 [file genes-17-00436-s001.zip › Figure S2/ITGB7.pdf]

PREDICTED: Trachemys scripta elegans integrin subunit beta 7 (ITGB7), mRNA

Sequence ID: XM_034791704.1 Length: 2907 Number of Matches: 1

Range 1: 817 to 2907 GenBank Graphics ▼ Next Match ▲ Previous Match

Score	Expect	Identities	Gaps	Strand
3862 bits(2091)	0.0	2091/2091(100%)	0/2091(0%)	Plus/Plus

```
Query  1     GAGCAGATTGGCTGGCGGAACGTGACACGTCTGCTGGTCTTCACGTCAGATGACGTCTTC  60
             ||||||||||||||||||||||||||||||||||||||||||||||||||||||||||||
Sbjct  817   GAGCAGATTGGCTGGCGGAACGTGACACGTCTGCTGGTCTTCACGTCAGATGACGTCTTC  876

Query  61    CACACGGCAGGGGATGGCAAGCTGGGTGGCATCTACCTCCCGAATGACAACCAGTGCCAC  120
             ||||||||||||||||||||||||||||||||||||||||||||||||||||||||||||
Sbjct  877   CACACGGCAGGGGATGGCAAGCTGGGTGGCATCTACCTCCCGAATGACAACCAGTGCCAC  936

Query  121   CTCGACACTGACGGCCTCTACAGCAAGAGCCACATCTACGATTACCCCTCGGTCGGGCAC  180
             ||||||||||||||||||||||||||||||||||||||||||||||||||||||||||||
Sbjct  937   CTCGACACTGACGGCCTCTACAGCAAGAGCCACATCTACGATTACCCCTCGGTCGGGCAC  996

Query  181   TTGGCCCAGGTGCTCTCAGCCTCTAACATCCAGCCCATCTTCGCTGTCACTGGCTCCACG  240
             ||||||||||||||||||||||||||||||||||||||||||||||||||||||||||||
Sbjct  997   TTGGCCCAGGTGCTCTCAGCCTCTAACATCCAGCCCATCTTCGCTGTCACTGGCTCCACG  1056

Query  241   CTGCCCGTGTACCAGGAGCTGAGTAGGCTGATCCCCAAGTCGGTGGTGGGGGAGCTGAAG  300
             ||||||||||||||||||||||||||||||||||||||||||||||||||||||||||||
Sbjct  1057  CTGCCCGTGTACCAGGAGCTGAGTAGGCTGATCCCCAAGTCGGTGGTGGGGGAGCTGAAG  1116

Query  301   GAGGATTCCAGCAACGTGGTGCAGCTCATCTCTGATGCCTACAATAGCCTGTCGTCCACG  360
             ||||||||||||||||||||||||||||||||||||||||||||||||||||||||||||
Sbjct  1117  GAGGATTCCAGCAACGTGGTGCAGCTCATCTCTGATGCCTACAATAGCCTGTCGTCCACG  1176

Query  361   GTGAACCTGGAGCACTTCCAGCTCCCGCCCGGCGTGAGCGTGGCCTACAAGTCGCACTGC  420
             ||||||||||||||||||||||||||||||||||||||||||||||||||||||||||||
Sbjct  1177  GTGAACCTGGAGCACTTCCAGCTCCCGCCCGGCGTGAGCGTGGCCTACAAGTCGCACTGC  1236

Query  421   AAGGACGCCACCGACTCCCTCGGGATCCACGGTGGGGTCTGCTCCGGTGTCCACATCAAC  480
             ||||||||||||||||||||||||||||||||||||||||||||||||||||||||||||
Sbjct  1237  AAGGACGCCACCGACTCCCTCGGGATCCACGGTGGGGTCTGCTCCGGTGTCCACATCAAC  1296

Query  481   CAGCTGGTGAGTTTCACGGTGACGGTGCAGGCCGACGCCTGCCTGGAGGGGCCGCACACC  540
             ||||||||||||||||||||||||||||||||||||||||||||||||||||||||||||
Sbjct  1297  CAGCTGGTGAGTTTCACGGTGACGGTGCAGGCCGACGCCTGCCTGGAGGGGCCGCACACC  1356

Query  541   TTCGCGCTGCGGGTGCTGGGCTTCACCGAGGAGGTGCGTGTGGAGCTGCAGACGCTGTGC  600
             ||||||||||||||||||||||||||||||||||||||||||||||||||||||||||||
Sbjct  1357  TTCGCGCTGCGGGTGCTGGGCTTCACCGAGGAGGTGCGTGTGGAGCTGCAGACGCTGTGC  1416

Query  601   GAGTGCCCCTGCAGCCAGCCTGAGCCCAACGCTACCCACTGCAGCGGGGGCCACGGCACC  660
             ||||||||||||||||||||||||||||||||||||||||||||||||||||||||||||
Sbjct  1417  GAGTGCCCCTGCAGCCAGCCTGAGCCCAACGCTACCCACTGCAGCGGGGGCCACGGCACC  1476

Query  661   CTCACCTGCGGGGTCTGCAGCTGCAGCCCTGGCCGCGTAGGCAAGCTGTGTGAGTGCGAG  720
             ||||||||||||||||||||||||||||||||||||||||||||||||||||||||||||
Sbjct  1477  CTCACCTGCGGGGTCTGCAGCTGCAGCCCTGGCCGCGTAGGCAAGCTGTGTGAGTGCGAG  1536

Query  721   CTGGCGGAGGCCACGGACCTGGATGCGGGGTGCCGGGGCCGGAACGGCACGGGCCCCACG  780
             ||||||||||||||||||||||||||||||||||||||||||||||||||||||||||||
Sbjct  1537  CTGGCGGAGGCCACGGACCTGGATGCGGGGTGCCGGGGCCGGAACGGCACGGGCCCCACG  1596

Query  781   TGCAGCGGGAAGGGGCAGTGCGTGTGCGGGCAGTGCCAGTGCAACAGCAACGTGCGAGGG  840
             ||||||||||||||||||||||||||||||||||||||||||||||||||||||||||||
Sbjct  1597  TGCAGCGGGAAGGGGCAGTGCGTGTGCGGGCAGTGCCAGTGCAACAGCAACGTGCGAGGG  1656

Query  841   CAGCACTGCGAGTGCGACGACACCAGCTGCGAGCGGCACGACGGGCAGCTCTGCGCAGGC  900
             ||||||||||||||||||||||||||||||||||||||||||||||||||||||||||||
Sbjct  1657  CAGCACTGCGAGTGCGACGACACCAGCTGCGAGCGGCACGACGGGCAGCTCTGCGCAGGC  1716

Query  901   CAGGGTCGGTGCCAGTGTGGGAGTTGCATGTGCAACGAGGGCTACACGGGCAGCGCCTGT  960
             ||||||||||||||||||||||||||||||||||||||||||||||||||||||||||||
Sbjct  1717  CAGGGTCGGTGCCAGTGTGGGAGTTGCATGTGCAACGAGGGCTACACGGGCAGCGCCTGT  1776

Query  961   GACTGCAGCCTGGACACCCGCGCGTGCCTGCAGGACGGCGTGGAGTGCAGCGGGCACGGG  1020
             ||||||||||||||||||||||||||||||||||||||||||||||||||||||||||||
Sbjct  1777  GACTGCAGCCTGGACACCCGCGCGTGCCTGCAGGACGGCGTGGAGTGCAGCGGGCACGGG  1836

Query  1021  CGCTGCGTCTGCAACAAATGTCAGTGCCAGCCGGGCTACTTCGACCGGCTCTGCAGCCGG  1080
             ||||||||||||||||||||||||||||||||||||||||||||||||||||||||||||
Sbjct  1837  CGCTGCGTCTGCAACAAATGTCAGTGCCAGCCGGGCTACTTCGACCGGCTCTGCAGCCGG  1896

Query  1081  TGCACGAACTGCCGGACCCCCTGCGAGGAGCACCGGGACTGTGCCGACTGCCAGGCCTTC  1140
             ||||||||||||||||||||||||||||||||||||||||||||||||||||||||||||
Sbjct  1897  TGCACGAACTGCCGGACCCCCTGCGAGGAGCACCGGGACTGTGCCGACTGCCAGGCCTTC  1956

Query  1141  AGGATGGGCCCGCTGAGCCAGAACTGCAGCATCGCCTGCAACCACACCGTGGCCACAGTG  1200
             ||||||||||||||||||||||||||||||||||||||||||||||||||||||||||||
Sbjct  1957  AGGATGGGCCCGCTGAGCCAGAACTGCAGCATCGCCTGCAACCACACCGTGGCCACAGTG  2016

Query  1201  GTGCCTGAGGCCACCGTGAACGAGCAGTGGTGCAAGGAGAAGACGGACGACGAGCGGATC  1260
             ||||||||||||||||||||||||||||||||||||||||||||||||||||||||||||
Sbjct  2017  GTGCCTGAGGCCACCGTGAACGAGCAGTGGTGCAAGGAGAAGACGGACGACGAGCGGATC  2076

Query  1261  CTGATCTTCCTGATTGAGGGCACGGAGGGGGGCAAAGTTGCCCTGAAGGTGAAAGACAAG  1320
             ||||||||||||||||||||||||||||||||||||||||||||||||||||||||||||
Sbjct  2077  CTGATCTTCCTGATTGAGGGCACGGAGGGGGGCAAAGTTGCCCTGAAGGTGAAAGACAAG  2136

Query  1321  GACGCTGTCACTGACCAGACCTCCATGATCGTGCTGGGCTCGGTCCTGGGTATCGTGGTC  1380
             ||||||||||||||||||||||||||||||||||||||||||||||||||||||||||||
Sbjct  2137  GACGCTGTCACTGACCAGACCTCCATGATCGTGCTGGGCTCGGTCCTGGGTATCGTGGTC  2196

Query  1381  ATCGGGCTGCTGCTCGTCATCGCCTACCGCATCTCCGTGGAGATCCTGGACCGTCGGGAG  1440
             ||||||||||||||||||||||||||||||||||||||||||||||||||||||||||||
Sbjct  2197  ATCGGGCTGCTGCTCGTCATCGCCTACCGCATCTCCGTGGAGATCCTGGACCGTCGGGAG  2256

Query  1441  TACATGCGCTTCGAGAAGGAGCGGGAGCGGGCCAAGGGGAACGAGGTCAACAACCCACTG  1500
             ||||||||||||||||||||||||||||||||||||||||||||||||||||||||||||
Sbjct  2257  TACATGCGCTTCGAGAAGGAGCGGGAGCGGGCCAAGGGGAACGAGGTCAACAACCCACTG  2316

Query  1501  TTCCAGAGTGCCACCACCACCGTCATCAACCCCAGGTACAACGAGGACTGAGGCAGCATG  1560
             ||||||||||||||||||||||||||||||||||||||||||||||||||||||||||||
Sbjct  2317  TTCCAGAGTGCCACCACCACCGTCATCAACCCCAGGTACAACGAGGACTGAGGCAGCATG  2376

Query  1561  GATGGCCGCCCCGGCGCCAGCCCAGCTACCAACGGCACGGGCCCCCGGTGCCACGCTCGC  1620
             ||||||||||||||||||||||||||||||||||||||||||||||||||||||||||||
Sbjct  2377  GATGGCCGCCCCGGCGCCAGCCCAGCTACCAACGGCACGGGCCCCCGGTGCCACGCTCGC  2436

Query  1621  TACAGCAGCCTCCCCCCGAGCTCTCCTGGAAGGGAAGGCGCAGGCAGAGGGCCTGCTGCT  1680
             ||||||||||||||||||||||||||||||||||||||||||||||||||||||||||||
Sbjct  2437  TACAGCAGCCTCCCCCCGAGCTCTCCTGGAAGGGAAGGCGCAGGCAGAGGGCCTGCTGCT  2496

Query  1681  CGGTGGAATCCCACGCTGCTACCCGCTGCCAAATAGGCCCACGGGGACGTGCCTGGTTGT  1740
             ||||||||||||||||||||||||||||||||||||||||||||||||||||||||||||
Sbjct  2497  CGGTGGAATCCCACGCTGCTACCCGCTGCCAAATAGGCCCACGGGGACGTGCCTGGTTGT  2556

Query  1741  GCTGCCAGGCGCGCCCACTTCTTACTGGAGGAGCAGGATGCAGCCCTGGGGCTCTTCCCA  1800
             ||||||||||||||||||||||||||||||||||||||||||||||||||||||||||||
Sbjct  2557  GCTGCCAGGCGCGCCCACTTCTTACTGGAGGAGCAGGATGCAGCCCTGGGGCTCTTCCCA  2616

Query  1801  GCCCAAAGCCTCCCTGCTTAAGTAGCCCCACCCTGTCCTCCCCTCGCAGCACTAAGTCCC  1860
             ||||||||||||||||||||||||||||||||||||||||||||||||||||||||||||
Sbjct  2617  GCCCAAAGCCTCCCTGCTTAAGTAGCCCCACCCTGTCCTCCCCTCGCAGCACTAAGTCCC  2676

Query  1861  GGAGCCTGCTGCTTCTCTGCCCCCTGCAGCGCATCAGTCAGCGACCCCCAGCACCAGGAG  1920
             ||||||||||||||||||||||||||||||||||||||||||||||||||||||||||||
Sbjct  2677  GGAGCCTGCTGCTTCTCTGCCCCCTGCAGCGCATCAGTCAGCGACCCCCAGCACCAGGAG  2736

Query  1921  AGCACGAGCTGCTGTGCACGCCCAGCAGGAGAGCTCTGGGGTCAGAGACTAGCAGGAAGG  1980
             ||||||||||||||||||||||||||||||||||||||||||||||||||||||||||||
Sbjct  2737  AGCACGAGCTGCTGTGCACGCCCAGCAGGAGAGCTCTGGGGTCAGAGACTAGCAGGAAGG  2796

Query  1981  GCCGGGCTACGGAGCACCAAAGCTCCACTTTTGGGGGGCTTCTCTCCAAGGCACTGTAGG  2040
             ||||||||||||||||||||||||||||||||||||||||||||||||||||||||||||
Sbjct  2797  GCCGGGCTACGGAGCACCAAAGCTCCACTTTTGGGGGGCTTCTCTCCAAGGCACTGTAGG  2856

Query  2041  GCAGAGTAGAGCCAGGGGAAACTCATGGCCAACCATGGGGGCGGGGCGGGG  2091
             |||||||||||||||||||||||||||||||||||||||||||||||||||
Sbjct  2857  GCAGAGTAGAGCCAGGGGAAACTCATGGCCAACCATGGGGGCGGGGCGGGG  2907
```
