# Supplementary material for: Divergent IL18-STAT1 Immune Responses Underlie Differential Susceptibility to Aeromonas hydrophila in Geoclemys hamiltonii and Trachemys scripta: A Comparative Transcriptomic Perspective
Source: Genes (Basel). 2026 Apr 9;17(4):436. doi: 10.3390/genes17040436 (PMC13116093; doi:10.3390/genes17040436)
Supplement: Supplementary file 1 [file genes-17-00436-s001.zip › Figure S2/NCKAP1L.pdf]

PREDICTED: Trachemys scripta elegans NCK associated protein 1 like (NCKAP1L), mRNA

Sequence ID: XM_034792820.1 **Length:** 2850 **Number of Matches:** 1

Range 1: 1 to 2850 GenBank Graphics ▼ Next Match ▲ Previous Match

Score	Expect	Identities	Gaps	Strand
5264 bits(2850)	0.0	2850/2850(100%)	0/2850(0%)	Plus/Plus

```
Query  1     GGAAGCACCAGTTGAAGGAGCGGCAGCAACGCAGCCCCATATCCCCTCGCGCAGACGTGC  60
             ||||||||||||||||||||||||||||||||||||||||||||||||||||||||||||
Sbjct  1     GGAAGCACCAGTTGAAGGAGCGGCAGCAACGCAGCCCCATATCCCCTCGCGCAGACGTGC  60

Query  61    CATTTTGGTGACTTGGTGCCTGTTGGGTGTGTAGCCGTTGGGAGGCCTGGCCTTTTCCCT  120
             ||||||||||||||||||||||||||||||||||||||||||||||||||||||||||||
Sbjct  61    CATTTTGGTGACTTGGTGCCTGTTGGGTGTGTAGCCGTTGGGAGGCCTGGCCTTTTCCCT  120

Query  121   CCAGCGGGTAATTGGGTGCGGAgggggggCGAGATCTCACCTGGAGAGATGTCTCTGCCC  180
             ||||||||||||||||||||||||||||||||||||||||||||||||||||||||||||
Sbjct  121   CCAGCGGGTAATTGGGTGCGGAGGGGGGGCGAGATCTCACCTGGAGAGATGTCTCTGCCC  180

Query  181   TCCATCTATCAGCACAAGTTCGCGGAGAAGCTGACGATCCTCAATGACAGGGGGAAGGGC  240
             ||||||||||||||||||||||||||||||||||||||||||||||||||||||||||||
Sbjct  181   TCCATCTATCAGCACAAGTTCGCGGAGAAGCTGACGATCCTCAATGACAGGGGGAAGGGC  240

Query  241   GTCCTCATCCGCATGTACAACATCAAGAAGACGTGCTCAGACCCCAGGTCCAAACCCCCC  300
             ||||||||||||||||||||||||||||||||||||||||||||||||||||||||||||
Sbjct  241   GTCCTCATCCGCATGTACAACATCAAGAAGACGTGCTCAGACCCCAGGTCCAAACCCCCC  300

Query  301   TTCTTCACCGAGAAGACCATGGAGCCGTCCATCAAATACATCAACAAGAAGTTCCCAAAT  360
             ||||||||||||||||||||||||||||||||||||||||||||||||||||||||||||
Sbjct  301   TTCTTCACCGAGAAGACCATGGAGCCGTCCATCAAATACATCAACAAGAAGTTCCCAAAT  360

Query  361   GTGGACGCACGGAGCAGCACGCAACACCTGGGCCCGGTGCACAAGGAGAAGGCAGAGATC  420
             ||||||||||||||||||||||||||||||||||||||||||||||||||||||||||||
Sbjct  361   GTGGACGCACGGAGCAGCACGCAACACCTGGGCCCGGTGCACAAGGAGAAGGCAGAGATC  420

Query  421   ATCAAAGTGCTCAATAGCTACTACCAGTCCTTCGTGGACGTGATGGAGTTCCGGGACCAT  480
             ||||||||||||||||||||||||||||||||||||||||||||||||||||||||||||
Sbjct  421   ATCAAAGTGCTCAATAGCTACTACCAGTCCTTCGTGGACGTGATGGAGTTCCGGGACCAT  480

Query  481   GTGTACGAGCTGCTGAACACCATCGATGCCAGCCAGTGCTACTTCGACATCCATGTGAAC  540
             ||||||||||||||||||||||||||||||||||||||||||||||||||||||||||||
Sbjct  481   GTGTACGAGCTGCTGAACACCATCGATGCCAGCCAGTGCTACTTCGACATCCATGTGAAC  540

Query  541   TACGACCTCACCAAGAACTACCTGGACCTGGTAGTGACCTACACGTCTGTCATCCTGTTG  600
             ||||||||||||||||||||||||||||||||||||||||||||||||||||||||||||
Sbjct  541   TACGACCTCACCAAGAACTACCTGGACCTGGTAGTGACCTACACGTCTGTCATCCTGTTG  600

Query  601   CTGTCGCGCATCGAGGACCGCAAAGCCCTGATTGGGATGTACAACTGCGCCCACGAGATG  660
             ||||||||||||||||||||||||||||||||||||||||||||||||||||||||||||
Sbjct  601   CTGTCGCGCATCGAGGACCGCAAAGCCCTGATTGGGATGTACAACTGCGCCCACGAGATG  660

Query  661   ATCCAGGGCAGCGGCGACCCCAGCTATGCCCGGCTGGCACAGATGGTGCTGGAGTACGAT  720
             ||||||||||||||||||||||||||||||||||||||||||||||||||||||||||||
Sbjct  661   ATCCAGGGCAGCGGCGACCCCAGCTATGCCCGGCTGGCACAGATGGTGCTGGAGTACGAT  720

Query  721   GCCCCACTCAAGAAACTCACAGAGGAGTTCGGGCCTCACACCAAGGCCGTGACCAGCGCC  780
             ||||||||||||||||||||||||||||||||||||||||||||||||||||||||||||
Sbjct  721   GCCCCACTCAAGAAACTCACAGAGGAGTTCGGGCCTCACACCAAGGCCGTGACCAGCGCC  780

Query  781   CTCCTGTCTCTCCATTTCCTCTTTGCCCGGAGGAACCAGTCGGCCGAGCAGTGGCGCAGT  840
             ||||||||||||||||||||||||||||||||||||||||||||||||||||||||||||
Sbjct  781   CTCCTGTCTCTCCATTTCCTCTTTGCCCGGAGGAACCAGTCGGCCGAGCAGTGGCGCAGT  840

Query  841   GACCAGCTCCTGAGCCTGATCAGCAACTCGGCCGCCATGCTGAGCCCGGCCAGCTCGGAC  900
             ||||||||||||||||||||||||||||||||||||||||||||||||||||||||||||
Sbjct  841   GACCAGCTCCTGAGCCTGATCAGCAACTCGGCCGCCATGCTGAGCCCGGCCAGCTCGGAC  900

Query  901   GTCATGGCCTGCGAGTACCTGTCGCTGGAGGTGATCGAGCGCTGGATACTGATTGGCTTC  960
             ||||||||||||||||||||||||||||||||||||||||||||||||||||||||||||
Sbjct  901   GTCATGGCCTGCGAGTACCTGTCGCTGGAGGTGATCGAGCGCTGGATACTGATTGGCTTC  960

Query  961   CTTCTGTGCCACGGCTGCCTGAGCACGAACCCGCAGTGCCTGGAGCTGTGGAAGCTGGGC  1020
             ||||||||||||||||||||||||||||||||||||||||||||||||||||||||||||
Sbjct  961   CTTCTGTGCCACGGCTGCCTGAGCACGAACCCGCAGTGCCTGGAGCTGTGGAAGCTGGGC  1020

Query  1021  CTGCAGGGCTCGCTCTACATCAGCCTCATCCGCGACGACGCCCTGCAGATCCACAAGGTC  1080
             ||||||||||||||||||||||||||||||||||||||||||||||||||||||||||||
Sbjct  1021  CTGCAGGGCTCGCTCTACATCAGCCTCATCCGCGACGACGCCCTGCAGATCCACAAGGTC  1080

Query  1081  ACTGAGGAGTTCTTCGGCAGTCTGAAAGGGTATGGGAAGCGGGTGGCTGATATCAAGGAG  1140
             ||||||||||||||||||||||||||||||||||||||||||||||||||||||||||||
Sbjct  1081  ACTGAGGAGTTCTTCGGCAGTCTGAAAGGGTATGGGAAGCGGGTGGCTGATATCAAGGAG  1140

Query  1141  TGCAAGGAGCATGCTGTGGCGCACAGTGGCCAGCTGCACCGGAACCGGCGGGCTTTCCTA  1200
             ||||||||||||||||||||||||||||||||||||||||||||||||||||||||||||
Sbjct  1141  TGCAAGGAGCATGCTGTGGCGCACAGTGGCCAGCTGCACCGGAACCGGCGGGCTTTCCTA  1200

Query  1201  CGCAACGCGGTGCGGGAGCTGGAGGCGCTGCTGAGCGACCAGCCCGGGCTGCTGGGCCCC  1260
             ||||||||||||||||||||||||||||||||||||||||||||||||||||||||||||
Sbjct  1201  CGCAACGCGGTGCGGGAGCTGGAGGCGCTGCTGAGCGACCAGCCCGGGCTGCTGGGCCCC  1260

Query  1261  AAGGCTCTCTGCGTCTTCATGTCGCTCTCCTTGTGCCGTGACGAGGTGAACTGGCTGGTG  1320
             ||||||||||||||||||||||||||||||||||||||||||||||||||||||||||||
Sbjct  1261  AAGGCTCTCTGCGTCTTCATGTCGCTCTCCTTGTGCCGTGACGAGGTGAACTGGCTGGTG  1320

Query  1321  CGTCATGCTGAGCATGTCACCAAGACCAAGACCCCTGAGGACTACGTTGACAGTCACATC  1380
             ||||||||||||||||||||||||||||||||||||||||||||||||||||||||||||
Sbjct  1321  CGTCATGCTGAGCATGTCACCAAGACCAAGACCCCTGAGGACTACGTTGACAGTCACATC  1380

Query  1381  GCCGAGCTGCTCTTCCTCATGGAGCAGCTGCGTACCCTCGTGCACAGGCATGGCCCAGTG  1440
             ||||||||||||||||||||||||||||||||||||||||||||||||||||||||||||
Sbjct  1381  GCCGAGCTGCTCTTCCTCATGGAGCAGCTGCGTACCCTCGTGCACAGGCATGGCCCAGTG  1440

Query  1441  ATCCAGCGCTACCACGTCCAGTACCTGGCTCGCTTTGACGCCCTGCTGCTCAGCGATATC  1500
             ||||||||||||||||||||||||||||||||||||||||||||||||||||||||||||
Sbjct  1441  ATCCAGCGCTACCACGTCCAGTACCTGGCTCGCTTTGACGCCCTGCTGCTCAGCGATATC  1500

Query  1501  ATCCAGAATCTGACCGTCTGCCCCGAGGAGAAGTCCATCATCATGTCGTCCTTCGTCAGC  1560
             ||||||||||||||||||||||||||||||||||||||||||||||||||||||||||||
Sbjct  1501  ATCCAGAATCTGACCGTCTGCCCCGAGGAGAAGTCCATCATCATGTCGTCCTTCGTCAGC  1560

Query  1561  ACCCTGTCGTCTCTCACTCTGAAACAAGTGGACAAAAAGGAGAAGTTCGACTTCTCTGGG  1620
             ||||||||||||||||||||||||||||||||||||||||||||||||||||||||||||
Sbjct  1561  ACCCTGTCGTCTCTCACTCTGAAACAAGTGGACAAAAAGGAGAAGTTCGACTTCTCTGGG  1620

Query  1621  CTTCGCCTGGACTGGTTCCGGCTGCAGGCTTACACCAGTGTGGCCAAGGCCCCCATGCAG  1680
             ||||||||||||||||||||||||||||||||||||||||||||||||||||||||||||
Sbjct  1621  CTTCGCCTGGACTGGTTCCGGCTGCAGGCTTACACCAGTGTGGCCAAGGCCCCCATGCAG  1680

Query  1681  CTGCGCGAGAGCACGGATGTGGGCCGTGTGATGAACCTCATCATGTTCCACTCCAAGATG  1740
             ||||||||||||||||||||||||||||||||||||||||||||||||||||||||||||
Sbjct  1681  CTGCGCGAGAGCACGGATGTGGGCCGTGTGATGAACCTCATCATGTTCCACTCCAAGATG  1740

Query  1741  CTGGACTCACTGGAGGAGTTGGTAGTGGAGACTTCGGACCTGTCGGCCTTGTGTTTCTAC  1800
             ||||||||||||||||||||||||||||||||||||||||||||||||||||||||||||
Sbjct  1741  CTGGACTCACTGGAGGAGTTGGTAGTGGAGACTTCGGACCTGTCGGCCTTGTGTTTCTAC  1800

Query  1801  GTCCGCCCCTTTGAGAAGCTGTTTGCTCTGACCATGGAGGAGCCGGCCATGCTGCGCTTC  1860
             ||||||||||||||||||||||||||||||||||||||||||||||||||||||||||||
Sbjct  1801  GTCCGCCCCTTTGAGAAGCTGTTTGCTCTGACCATGGAGGAGCCGGCCATGCTGCGCTTC  1860

Query  1861  ACCATCGCCTTCCCGCTTGTCTGCAGCCACTTTGTTCACTGCACCCACTCCATGTGTCCA  1920
             ||||||||||||||||||||||||||||||||||||||||||||||||||||||||||||
Sbjct  1861  ACCATCGCCTTCCCGCTTGTCTGCAGCCACTTTGTTCACTGCACCCACTCCATGTGTCCA  1920

Query  1921  GAGGAGTATCCCCACCTGAAGAGCTGCAGCCTGGGTCTGTGCAACAACTTCCTAGAGGAG  1980
             ||||||||||||||||||||||||||||||||||||||||||||||||||||||||||||
Sbjct  1921  GAGGAGTATCCCCACCTGAAGAGCTGCAGCCTGGGTCTGTGCAACAACTTCCTAGAGGAG  1980

Query  1981  ATCGCCAAGCAGGCTGCCAACTGCATCATGGACGCATGTGCCGAGCAGCGCAAACTCAGT  2040
             ||||||||||||||||||||||||||||||||||||||||||||||||||||||||||||
Sbjct  1981  ATCGCCAAGCAGGCTGCCAACTGCATCATGGACGCATGTGCCGAGCAGCGCAAACTCAGT  2040

Query  2041  GAGCAGCTGCTGCCCAAACATTGCGCCTCCACCATCAGCAAGGCCCGGAACAAGAAGGCC  2100
             ||||||||||||||||||||||||||||||||||||||||||||||||||||||||||||
Sbjct  2041  GAGCAGCTGCTGCCCAAACATTGCGCCTCCACCATCAGCAAGGCCCGGAACAAGAAGGCC  2100

Query  2101  CAGAAGCAGCCCCCCAAGAAGGGGGAGCCGGAGCGGGACAAGCCGGGGGCCGAGAGCCAG  2160
             ||||||||||||||||||||||||||||||||||||||||||||||||||||||||||||
Sbjct  2101  CAGAAGCAGCCCCCCAAGAAGGGGGAGCCGGAGCGGGACAAGCCGGGGGCCGAGAGCCAG  2160

Query  2161  AGGAAGGACCGGATCGTGGTGACCAACATGGACAAGCTGCACCAGATGCTGAGCGAGCTC  2220
             ||||||||||||||||||||||||||||||||||||||||||||||||||||||||||||
Sbjct  2161  AGGAAGGACCGGATCGTGGTGACCAACATGGACAAGCTGCACCAGATGCTGAGCGAGCTC  2220

Query  2221  TCCCTGAGCGTCAACCACATCCCCAGCTTCACCGTGTTCGAGCACATGATCACGCCGGCC  2280
             ||||||||||||||||||||||||||||||||||||||||||||||||||||||||||||
Sbjct  2221  TCCCTGAGCGTCAACCACATCCCCAGCTTCACCGTGTTCGAGCACATGATCACGCCGGCC  2280

Query  2281  GAATACCTCAGCAGCCACCTGGAGACGCGGCTCAACAGATCTTTTGTGTGGATGGCCAAC  2340
             ||||||||||||||||||||||||||||||||||||||||||||||||||||||||||||
Sbjct  2281  GAATACCTCAGCAGCCACCTGGAGACGCGGCTCAACAGATCTTTTGTGTGGATGGCCAAC  2340

Query  2341  TACAACCAGACTACGCAGGAGATTGCACGGCCCTCTGAGGTGCTGGCCAGCCTGCGCTCC  2400
             ||||||||||||||||||||||||||||||||||||||||||||||||||||||||||||
Sbjct  2341  TACAACCAGACTACGCAGGAGATTGCACGGCCCTCTGAGGTGCTGGCCAGCCTGCGCTCC  2400

Query  2401  TACCTCGCCTTCATCCAGTCGTTGGGGCAGTTCACGTGCATGGACTCCAGCCGCATCATC  2460
             ||||||||||||||||||||||||||||||||||||||||||||||||||||||||||||
Sbjct  2401  TACCTCGCCTTCATCCAGTCGTTGGGGCAGTTCACGTGCATGGACTCCAGCCGCATCATC  2460

Query  2461  CGCAACGTCCTGCTTCAGCAGACACAGCCCCTGGACTCCAGCGGGGAGCAGACTCTCACC  2520
             ||||||||||||||||||||||||||||||||||||||||||||||||||||||||||||
Sbjct  2461  CGCAACGTCCTGCTTCAGCAGACACAGCCCCTGGACTCCAGCGGGGAGCAGACTCTCACC  2520

Query  2521  ACCATCTACACCAACTGGTACCTGGAGGCGCTGCTGCGCCAGGCCAGCACTGGGGCCATT  2580
             ||||||||||||||||||||||||||||||||||||||||||||||||||||||||||||
Sbjct  2521  ACCATCTACACCAACTGGTACCTGGAGGCGCTGCTGCGCCAGGCCAGCACTGGGGCCATT  2580

Query  2581  GTGCTGTCCCCCGCCATGCAGGCCTTCATCAGTGTCCCCAGGGAGGGGGAGCAGAGCTTC  2640
             ||||||||||||||||||||||||||||||||||||||||||||||||||||||||||||
Sbjct  2581  GTGCTGTCCCCCGCCATGCAGGCCTTCATCAGTGTCCCCAGGGAGGGGGAGCAGAGCTTC  2640

Query  2641  AGTGCAGAGGAGTTCTCAGACATCTCAGAGATGCGGGCGCTGGCTGAGCTGCTGGGTCCC  2700
             ||||||||||||||||||||||||||||||||||||||||||||||||||||||||||||
Sbjct  2641  AGTGCAGAGGAGTTCTCAGACATCTCAGAGATGCGGGCGCTGGCTGAGCTGCTGGGTCCC  2700

Query  2701  TACGGCATGAAGTTCCTGAGCGACAACCTCATGTGGCACGTCACCTCGCAGGTGGTGGAG  2760
             ||||||||||||||||||||||||||||||||||||||||||||||||||||||||||||
Sbjct  2701  TACGGCATGAAGTTCCTGAGCGACAACCTCATGTGGCACGTCACCTCGCAGGTGGTGGAG  2760

Query  2761  CTGAAGGTAGGAGGCTGGGGCGGGAGGAGCCACTGCTGGCTGCACTCAACTCCCCAGGGT  2820
             ||||||||||||||||||||||||||||||||||||||||||||||||||||||||||||
Sbjct  2761  CTGAAGGTAGGAGGCTGGGGCGGGAGGAGCCACTGCTGGCTGCACTCAACTCCCCAGGGT  2820

Query  2821  TACGGGCAGGCAGCGGGAGTCAGTGCTTAG  2850
             ||||||||||||||||||||||||||||||
Sbjct  2821  TACGGGCAGGCAGCGGGAGTCAGTGCTTAG  2850
```
