# Supplementary material for: Divergent IL18-STAT1 Immune Responses Underlie Differential Susceptibility to Aeromonas hydrophila in Geoclemys hamiltonii and Trachemys scripta: A Comparative Transcriptomic Perspective
Source: Genes (Basel). 2026 Apr 9;17(4):436. doi: 10.3390/genes17040436 (PMC13116093; doi:10.3390/genes17040436)
Supplement: Supplementary file 1 [file genes-17-00436-s001.zip › Figure S2/RRAS2.pdf]

PREDICTED: Trachemys scripta elegans RAS related 2 (RRAS2), mRNA

Sequence ID: XM_034770110.1 **Length:** 2609 **Number of Matches:** 1

Range 1: 1 to 2609 GenBank Graphics ▼ Next Match ▲ Previous Match

Score	Expect	Identities	Gaps	Strand
4819 bits(2609)	0.0	2609/2609(100%)	0/2609(0%)	Plus/Plus

```
Query  1     CCGACGGCCATTTGTGTAGCCGCGCTGGAGCCGCCGCTGCCCTCATTTGGCCCGTGGCCG  60
             ||||||||||||||||||||||||||||||||||||||||||||||||||||||||||||
Sbjct  1     CCGACGGCCATTTGTGTAGCCGCGCTGGAGCCGCCGCTGCCCTCATTTGGCCCGTGGCCG  60

Query  61    CCGCGGTGAGGAGCCGGCTGGGTTGGGGCCGGCAGCGCGGATCTGCTCCCCGCCCCGCGG  120
             ||||||||||||||||||||||||||||||||||||||||||||||||||||||||||||
Sbjct  61    CCGCGGTGAGGAGCCGGCTGGGTTGGGGCCGGCAGCGCGGATCTGCTCCCCGCCCCGCGG  120

Query  121   AGCTCTGGGCGCTGCTACTGCCGCCGCGACCCCGCCCGAGGAGCAGGAGGACCAGGAGCG  180
             ||||||||||||||||||||||||||||||||||||||||||||||||||||||||||||
Sbjct  121   AGCTCTGGGCGCTGCTACTGCCGCCGCGACCCCGCCCGAGGAGCAGGAGGACCAGGAGCG  180

Query  181   GGGCCTGCGGCTTCCAGTccggccggaggcggcggcggcggcgcagggagggacccggac  240
             ||||||||||||||||||||||||||||||||||||||||||||||||||||||||||||
Sbjct  181   GGGCCTGCGGCTTCCAGTCCGGCCGGAGGCGGCGGCGGCGGCGCAGGGAGGGACCCGGAC  240

Query  241   ccggacccggcggcgctgctcggctcggctCCCCGATGGCCGCGGGCTGCAGGGACGGGC  300
             ||||||||||||||||||||||||||||||||||||||||||||||||||||||||||||
Sbjct  241   CCGGACCCGGCGGCGCTGCTCGGCTCGGCTCCCCGATGGCCGCGGGCTGCAGGGACGGGC  300

Query  301   CGGGGCAGGAGAAGTATCGGCTGGTGGTGGTGGGCGGCGGCGGCGTGGGCAAGTCGGCGC  360
             ||||||||||||||||||||||||||||||||||||||||||||||||||||||||||||
Sbjct  301   CGGGGCAGGAGAAGTATCGGCTGGTGGTGGTGGGCGGCGGCGGCGTGGGCAAGTCGGCGC  360

Query  361   TCACCATCCAGTTCATCCAGTCCTACTTTGTCACGGATTATGATCCGACAATTGAAGACT  420
             ||||||||||||||||||||||||||||||||||||||||||||||||||||||||||||
Sbjct  361   TCACCATCCAGTTCATCCAGTCCTACTTTGTCACGGATTATGATCCGACAATTGAAGACT  420

Query  421   CTTACACCAAACAGTGTGTGATAGATGAGAAAGCAGCACGGTTGGACATTCTGGATACAG  480
             ||||||||||||||||||||||||||||||||||||||||||||||||||||||||||||
Sbjct  421   CTTACACCAAACAGTGTGTGATAGATGAGAAAGCAGCACGGTTGGACATTCTGGATACAG  480

Query  481   CAGGACAAGAAGAATTTGGAGCTATGCGAGAACAGTATATGAGGACTGGGGAGGGCTTTC  540
             ||||||||||||||||||||||||||||||||||||||||||||||||||||||||||||
Sbjct  481   CAGGACAAGAAGAATTTGGAGCTATGCGAGAACAGTATATGAGGACTGGGGAGGGCTTTC  540

Query  541   TCCTTGTCTTCTCAGTCACTGATAGAGGAAGTTTTGAAGAAATCTACAAGTTTCAAAGGC  600
             ||||||||||||||||||||||||||||||||||||||||||||||||||||||||||||
Sbjct  541   TCCTTGTCTTCTCAGTCACTGATAGAGGAAGTTTTGAAGAAATCTACAAGTTTCAAAGGC  600

Query  601   AGATACTTAGAGTGAAAGACCGTGATGAATTTCCTATGATTCTAGTTGGTAACAAAGCAG  660
             ||||||||||||||||||||||||||||||||||||||||||||||||||||||||||||
Sbjct  601   AGATACTTAGAGTGAAAGACCGTGATGAATTTCCTATGATTCTAGTTGGTAACAAAGCAG  660

Query  661   ATCTGGATCATCAAAGACAGGTAACACAAGAGGAAGGCCAGCAACTAGCACGACAGCTTA  720
             ||||||||||||||||||||||||||||||||||||||||||||||||||||||||||||
Sbjct  661   ATCTGGATCATCAAAGACAGGTAACACAAGAGGAAGGCCAGCAACTAGCACGACAGCTTA  720

Query  721   AAGTAACTTATATGGAAGCCTCAGCAAAAATACGATTGAATGTAGACCAAGCCTTTCATG  780
             ||||||||||||||||||||||||||||||||||||||||||||||||||||||||||||
Sbjct  721   AAGTAACTTATATGGAAGCCTCAGCAAAAATACGATTGAATGTAGACCAAGCCTTTCATG  780

Query  781   AACTTGTCAGAGTTATAAGAAAATTTCAAGAACAAGAGTGCCCTCCTTCACCAGAACCAA  840
             ||||||||||||||||||||||||||||||||||||||||||||||||||||||||||||
Sbjct  781   AACTTGTCAGAGTTATAAGAAAATTTCAAGAACAAGAGTGCCCTCCTTCACCAGAACCAA  840

Query  841   CACGGAAAGAAAAAGACAAGAAAGGCTGTCATTGTGTCATTTTCTAAGAATCTCATGAAT  900
             ||||||||||||||||||||||||||||||||||||||||||||||||||||||||||||
Sbjct  841   CACGGAAAGAAAAAGACAAGAAAGGCTGTCATTGTGTCATTTTCTAAGAATCTCATGAAT  900

Query  901   CAAGCTATCAACTGCCAGATTAATTTTTCTTTCCACCATTTTGCATCACTTTGGGTATGT  960
             ||||||||||||||||||||||||||||||||||||||||||||||||||||||||||||
Sbjct  901   CAAGCTATCAACTGCCAGATTAATTTTTCTTTCCACCATTTTGCATCACTTTGGGTATGT  960

Query  961   CTAGCCTTTTGTGCCATGTCCTATAAATGGCCACCAAAATAGCCTTAGTCCAAGAAGCTG  1020
             ||||||||||||||||||||||||||||||||||||||||||||||||||||||||||||
Sbjct  961   CTAGCCTTTTGTGCCATGTCCTATAAATGGCCACCAAAATAGCCTTAGTCCAAGAAGCTG  1020

Query  1021  GCTGAATACAGTCCTAGAAGACAACTAGTCATCTTAGGGCAGACTTCAAAGCAAAACACT  1080
             ||||||||||||||||||||||||||||||||||||||||||||||||||||||||||||
Sbjct  1021  GCTGAATACAGTCCTAGAAGACAACTAGTCATCTTAGGGCAGACTTCAAAGCAAAACACT  1080

Query  1081  AAGGCTGCTTCTTTAAAATCACAGTTCCTATTTTTGCTCCCTTTAAGAGCTTGCACCTTG  1140
             ||||||||||||||||||||||||||||||||||||||||||||||||||||||||||||
Sbjct  1081  AAGGCTGCTTCTTTAAAATCACAGTTCCTATTTTTGCTCCCTTTAAGAGCTTGCACCTTG  1140

Query  1141  TGGAATTTTATTCATAAAGGAGATGAAACAAAACCTAAGAGGACAAGGTGTTCAAGTAAA  1200
             ||||||||||||||||||||||||||||||||||||||||||||||||||||||||||||
Sbjct  1141  TGGAATTTTATTCATAAAGGAGATGAAACAAAACCTAAGAGGACAAGGTGTTCAAGTAAA  1200

Query  1201  GCATAAAAGTTGTCTTATGTTAATTTATTTTTCACTTCTGACATTTCATTAGCTACTATC  1260
             ||||||||||||||||||||||||||||||||||||||||||||||||||||||||||||
Sbjct  1201  GCATAAAAGTTGTCTTATGTTAATTTATTTTTCACTTCTGACATTTCATTAGCTACTATC  1260

Query  1261  AAGGAGACCTCTAATCAAAGTGTAAAGTGTATTTAATAAAAAAGTGTATTGGCTTTTTGC  1320
             ||||||||||||||||||||||||||||||||||||||||||||||||||||||||||||
Sbjct  1261  AAGGAGACCTCTAATCAAAGTGTAAAGTGTATTTAATAAAAAAGTGTATTGGCTTTTTGC  1320

Query  1321  CCTTCAGTTAAATTAGAGTTCAGCTAGCCTTAAGAAACCAACACTGAATTTCTTTGTCAG  1380
             ||||||||||||||||||||||||||||||||||||||||||||||||||||||||||||
Sbjct  1321  CCTTCAGTTAAATTAGAGTTCAGCTAGCCTTAAGAAACCAACACTGAATTTCTTTGTCAG  1380

Query  1381  CAAAAATGTTTCAGCTCTTGTTTATGCTTCAGCtttttttCCAGTGGCCAAAAGTCTACT  1440
             ||||||||||||||||||||||||||||||||||||||||||||||||||||||||||||
Sbjct  1381  CAAAAATGTTTCAGCTCTTGTTTATGCTTCAGCTTTTTTTCCAGTGGCCAAAAGTCTACT  1440

Query  1441  TACTGTATTTATTTTATGGATCCAGAATAGCTACAGGATTATTACTACTATTATTATAAT  1500
             ||||||||||||||||||||||||||||||||||||||||||||||||||||||||||||
Sbjct  1441  TACTGTATTTATTTTATGGATCCAGAATAGCTACAGGATTATTACTACTATTATTATAAT  1500

Query  1501  GTGGCCAAATACCTGGGCTCATTCTGGTCTAGCCATTTCAGTTTATTAGAAAAATTTCAC  1560
             ||||||||||||||||||||||||||||||||||||||||||||||||||||||||||||
Sbjct  1501  GTGGCCAAATACCTGGGCTCATTCTGGTCTAGCCATTTCAGTTTATTAGAAAAATTTCAC  1560

Query  1561  ATTGTTgggggggAAAATTCATGTGTCTTCCTTTGATGTATTTCTGGGTAAGGGATTAAA  1620
             ||||||||||||||||||||||||||||||||||||||||||||||||||||||||||||
Sbjct  1561  ATTGTTGGGGGGGAAAATTCATGTGTCTTCCTTTGATGTATTTCTGGGTAAGGGATTAAA  1620

Query  1621  GAAAACTAAAATTGTAGCTGTTTTTATTTCATGTAATATAAAAAGATGGATTTGATCTTT  1680
             ||||||||||||||||||||||||||||||||||||||||||||||||||||||||||||
Sbjct  1621  GAAAACTAAAATTGTAGCTGTTTTTATTTCATGTAATATAAAAAGATGGATTTGATCTTT  1680

Query  1681  CCAATGTCAGAGATGATTAAATGTTTTTGCTATATACTTTTATACATTATTTTCTTATCA  1740
             ||||||||||||||||||||||||||||||||||||||||||||||||||||||||||||
Sbjct  1681  CCAATGTCAGAGATGATTAAATGTTTTTGCTATATACTTTTATACATTATTTTCTTATCA  1740

Query  1741  AACTAGTTAACAAGTATTTTTATATGTTTGTAAGCAAATATGCTTTCACAGCATAACTTG  1800
             ||||||||||||||||||||||||||||||||||||||||||||||||||||||||||||
Sbjct  1741  AACTAGTTAACAAGTATTTTTATATGTTTGTAAGCAAATATGCTTTCACAGCATAACTTG  1800

Query  1801  TGTATATGTAAAGATGAATATTTAATTCACGGTTCACGTTTAACTGACAAAATAATGCGG  1860
             ||||||||||||||||||||||||||||||||||||||||||||||||||||||||||||
Sbjct  1801  TGTATATGTAAAGATGAATATTTAATTCACGGTTCACGTTTAACTGACAAAATAATGCGG  1860

Query  1861  GATAGGCTGAACTGTGGTAGAACTTCTTGCTGGGTACAACCAGAACAGTAATGGCCAATC  1920
             ||||||||||||||||||||||||||||||||||||||||||||||||||||||||||||
Sbjct  1861  GATAGGCTGAACTGTGGTAGAACTTCTTGCTGGGTACAACCAGAACAGTAATGGCCAATC  1920

Query  1921  ATGTGCCGGCCCAACTTACCTATAAAAAGTAAAGTTTGGCATGCTTTCAAGTAAAGTTTA  1980
             ||||||||||||||||||||||||||||||||||||||||||||||||||||||||||||
Sbjct  1921  ATGTGCCGGCCCAACTTACCTATAAAAAGTAAAGTTTGGCATGCTTTCAAGTAAAGTTTA  1980

Query  1981  GTATCCCTTATGAAAGACTGACCATTATGTGAACTATATTAAACTGTAAGACTTTTAAAT  2040
             ||||||||||||||||||||||||||||||||||||||||||||||||||||||||||||
Sbjct  1981  GTATCCCTTATGAAAGACTGACCATTATGTGAACTATATTAAACTGTAAGACTTTTAAAT  2040

Query  2041  GACTGTTTAGTTTAACTGTGGATGGTTTATGAATTTTGAGTTCTGTGGATTGTGTTTAAA  2100
             ||||||||||||||||||||||||||||||||||||||||||||||||||||||||||||
Sbjct  2041  GACTGTTTAGTTTAACTGTGGATGGTTTATGAATTTTGAGTTCTGTGGATTGTGTTTAAA  2100

Query  2101  CAATTCAAGAGTATGTTCCCTGACTCTGAAATACTAAGTGGTATTGCACAGTTGTCACCT  2160
             ||||||||||||||||||||||||||||||||||||||||||||||||||||||||||||
Sbjct  2101  CAATTCAAGAGTATGTTCCCTGACTCTGAAATACTAAGTGGTATTGCACAGTTGTCACCT  2160

Query  2161  TATTGAATGTGTCCAACAGTTCTATGGAGTTTGGTTATTAAGCATACCTTTGTATAACTT  2220
             ||||||||||||||||||||||||||||||||||||||||||||||||||||||||||||
Sbjct  2161  TATTGAATGTGTCCAACAGTTCTATGGAGTTTGGTTATTAAGCATACCTTTGTATAACTT  2220

Query  2221  GAGGTGCTAGAATTAAAGATGATCTAACCATTATCAAGAGGTAAGCACATTGCACCTTTG  2280
             ||||||||||||||||||||||||||||||||||||||||||||||||||||||||||||
Sbjct  2221  GAGGTGCTAGAATTAAAGATGATCTAACCATTATCAAGAGGTAAGCACATTGCACCTTTG  2280

Query  2281  TTTGTCTTTTAAAAAACTGAATCTCTCCTTGGGAGAAAGGGTTAACAATAGCTGGAAGGG  2340
             ||||||||||||||||||||||||||||||||||||||||||||||||||||||||||||
Sbjct  2281  TTTGTCTTTTAAAAAACTGAATCTCTCCTTGGGAGAAAGGGTTAACAATAGCTGGAAGGG  2340

Query  2341  GGGTAGGACAATACAGGCAAACTTTTACATACTTTCAGCCTGACAAGAGCTTGAAATTTG  2400
             ||||||||||||||||||||||||||||||||||||||||||||||||||||||||||||
Sbjct  2341  GGGTAGGACAATACAGGCAAACTTTTACATACTTTCAGCCTGACAAGAGCTTGAAATTTG  2400

Query  2401  GCCTACAGTGATATAACCTGAATGGACTTAAAAGCATCTAAGTAGAAATGGCTTCCTGAT  2460
             ||||||||||||||||||||||||||||||||||||||||||||||||||||||||||||
Sbjct  2401  GCCTACAGTGATATAACCTGAATGGACTTAAAAGCATCTAAGTAGAAATGGCTTCCTGAT  2460

Query  2461  TCAACCGATGCTGTGACCAGCGGCTGACACCCGAGGACTACAGAATGATAGTTCACAAAA  2520
             ||||||||||||||||||||||||||||||||||||||||||||||||||||||||||||
Sbjct  2461  TCAACCGATGCTGTGACCAGCGGCTGACACCCGAGGACTACAGAATGATAGTTCACAAAA  2520

Query  2521  TAGACCATTCAAAATAAACTTCTTGTACTTGGCTATGTTTTGGCATATGCTAATTGTTGA  2580
             ||||||||||||||||||||||||||||||||||||||||||||||||||||||||||||
Sbjct  2521  TAGACCATTCAAAATAAACTTCTTGTACTTGGCTATGTTTTGGCATATGCTAATTGTTGA  2580

Query  2581  TTGCTCTGCCAATaaaaaaaaTTCTATAA  2609
             |||||||||||||||||||||||||||||
Sbjct  2581  TTGCTCTGCCAATAAAAAAAATTCTATAA  2609
```
