# Supplementary material for: Divergent IL18-STAT1 Immune Responses Underlie Differential Susceptibility to Aeromonas hydrophila in Geoclemys hamiltonii and Trachemys scripta: A Comparative Transcriptomic Perspective
Source: Genes (Basel). 2026 Apr 9;17(4):436. doi: 10.3390/genes17040436 (PMC13116093; doi:10.3390/genes17040436)
Supplement: Supplementary file 1 [file genes-17-00436-s001.zip › Figure S2/STAT1.pdf]

PREDICTED: Trachemys scripta elegans signal transducer and activator of transcription 1 (STAT1), transcript variant X2, mRNA

Sequence ID: XM_034785098.1 Length: 3617 Number of Matches: 1

Range 1: 1 to 3617 GenBank Graphics ▼ Next Match ▲ Previous Match

Score	Expect	Identities	Gaps	Strand
6554 bits(3549)	0.0	3617/3644(99%)	27/3644(0%)	Plus/Plus

```
Query  154   ATGACTCAGTGGTATCAGCTGCAGCAACTTGATTCCAAATTCTTGGAGCAAGTACACCAG  213
             ||||||||||||||||||||||||||||||||||||||||||||||||||||||||||||
Sbjct  1     ATGACTCAGTGGTATCAGCTGCAGCAACTTGATTCCAAATTCTTGGAGCAAGTACACCAG  60

Query  214   CTGTATGATGACAGCTTTCCCATGGAAATCAGACAGTATCTGGCACAATGGCTGGAAAAT  273
             ||||||||||||||||||||||||||||||||||||||||||||||||||||||||||||
Sbjct  61    CTGTATGATGACAGCTTTCCCATGGAAATCAGACAGTATCTGGCACAATGGCTGGAAAAT  120

Query  274   CAAGACTGGGAGCATGCTGCAAACAACGTTTCATTTGCTACAGTATTGTTCCATGACCTG  333
             ||||||||||||||||||||||||||||||||||||||||||||||||||||||||||||
Sbjct  121   CAAGACTGGGAGCATGCTGCAAACAACGTTTCATTTGCTACAGTATTGTTCCATGACCTG  180

Query  334   CTGTCACAGCTAGACGATCAGTTCAGTCGGTTTTTAATAGAAAACAACTTTTTGTTGCAA  393
             ||||||||||||||||||||||||||||||||||||||||||||||||||||||||||||
Sbjct  181   CTGTCACAGCTAGACGATCAGTTCAGTCGGTTTTTAATAGAAAACAACTTTTTGTTGCAA  240

Query  394   CACAACATCAGGAAAAGCAAACGTAATCTTCAGGATCATTTTCAAGAGGACCCAATACAA  453
             ||||||||||||||||||||||||||||||||||||||||||||||||||||||||||||
Sbjct  241   CACAACATCAGGAAAAGCAAACGTAATCTTCAGGATCATTTTCAAGAGGACCCAATACAA  300

Query  454   ATGGCAATGACAATCTTCAACTGTCTAAAGGAAGAAAGGAAAATACTGAGCAGCGCCCAG  513
             ||||||||||||||||||||||||||||||||||||||||||||||||||||||||||||
Sbjct  301   ATGGCAATGACAATCTTCAACTGTCTAAAGGAAGAAAGGAAAATACTGAGCAGCGCCCAG  360

Query  514   TTGTCAGACCAGATGCAGGTGGGGAACATACAGAATACTGTAATGCTGGACAAACAGAAG  573
             ||||||||||||||||||||||||||||||||||||||||||||||||||||||||||||
Sbjct  361   TTGTCAGACCAGATGCAGGTGGGGAACATACAGAATACTGTAATGCTGGACAAACAGAAG  420

Query  574   GAGCTGGATATGAAAGTCAGGAGTGTGAAGAACAACGTTGTGGAAGTGGAACAAGACATC  633
             ||||||||||||||||||||||||||||||||||||||||||||||||||||||||||||
Sbjct  421   GAGCTGGATATGAAAGTCAGGAGTGTGAAGAACAACGTTGTGGAAGTGGAACAAGACATC  480

Query  634   AAGACTCTAGAGGATGTGCAAGATGAATATGACTTTAAATGCAAAACCTTACAGAACAGA  693
             ||||||||||||||||||||||||||||||||||||||||||||||||||||||||||||
Sbjct  481   AAGACTCTAGAGGATGTGCAAGATGAATATGACTTTAAATGCAAAACCTTACAGAACAGA  540

Query  694   GAAAATGAGACCAGTGGAGTGGCACAGGATGAATATAAGAAAGAACAGCTTGTCCTCCAA  753
             ||||||||||||||||||||||||||||||||||||||||||||||||||||||||||||
Sbjct  541   GAAAATGAGACCAGTGGAGTGGCACAGGATGAATATAAGAAAGAACAGCTTGTCCTCCAA  600

Query  754   AAGATGTTTCTAACACTTGACCTTAAGCGAAAGGAAGTGGTGAGCAAAATAATAAATCTG  813
             ||||||||||||||||||||||||||||||||||||||||||||||||||||||||||||
Sbjct  601   AAGATGTTTCTAACACTTGACCTTAAGCGAAAGGAAGTGGTGAGCAAAATAATAAATCTG  660

Query  814   TTGAATATATCAGAGCACACACAAAGTGCTTTGATTAATGAAGAGCTTGTTGAATGGAAG  873
             ||||||||||||||||||||||||||||||||||||||||||||||||||||||||||||
Sbjct  661   TTGAATATATCAGAGCACACACAAAGTGCTTTGATTAATGAAGAGCTTGTTGAATGGAAG  720

Query  874   CATAGACAACAAATTGCTTGTATTGGTGGCCCACCCAATGCCTGCCTCGACCAGCTACAA  933
             ||||||||||||||||||||||||||||||||||||||||||||||||||||||||||||
Sbjct  721   CATAGACAACAAATTGCTTGTATTGGTGGCCCACCCAATGCCTGCCTCGACCAGCTACAA  780

Query  934   AACTGGTTCACTATTGTTGCTGAGAGTCTTCAGCAAGTTCGCCAGCAGCTTAAAAAGCTT  993
             ||||||||||||||||||||||||||||||||||||||||||||||||||||||||||||
Sbjct  781   AACTGGTTCACTATTGTTGCTGAGAGTCTTCAGCAAGTTCGCCAGCAGCTTAAAAAGCTT  840

Query  994   GAGGAATTGGAGCAGAAATTTACATATGACCCAGATCCCATTACAAAAAACAAACAAGTC  1053
             ||||||||||||||||||||||||||||||||||||||||||||||||||||||||||||
Sbjct  841   GAGGAATTGGAGCAGAAATTTACATATGACCCAGATCCCATTACAAAAAACAAACAAGTC  900

Query  1054  CTGCAAGACCGCACCTGCAATCTTTTCAAACAACTCATTCAGAGTTCTTTTGTGGTGGAG  1113
             ||||||||||||||||||||||||||||||||||||||||||||||||||||||||||||
Sbjct  901   CTGCAAGACCGCACCTGCAATCTTTTCAAACAACTCATTCAGAGTTCTTTTGTGGTGGAG  960

Query  1114  AGGCAGCCTTGCATGCCAACTCATCCTCAGAGGCCATTGGTCCTGAAGACTGGAGTGCAG  1173
             ||||||||||||||||||||||||||||||||||||||||||||||||||||||||||||
Sbjct  961   AGGCAGCCTTGCATGCCAACTCATCCTCAGAGGCCATTGGTCCTGAAGACTGGAGTGCAG  1020

Query  1174  TTCACAGTGAAACTGAGATTGCTGGTGAAGCTACAGGAACTGAATTATAATTTAAAAGTT  1233
             ||||||||||||||||||||||||||||||||||||||||||||||||||||||||||||
Sbjct  1021  TTCACAGTGAAACTGAGATTGCTGGTGAAGCTACAGGAACTGAATTATAATTTAAAAGTT  1080

Query  1234  AAAGTCTTATTTGATAAGGACGTGAATGAGAAGAACACGGTAAAAGGGTTCAGAAAATTT  1293
             ||||||||||||||||||||||||||||||||||||||||||||||||||||||||||||
Sbjct  1081  AAAGTCTTATTTGATAAGGACGTGAATGAGAAGAACACGGTAAAAGGGTTCAGAAAATTT  1140

Query  1294  AACATTCTGGGAACGAATACAAAAGTAATGAACATGGAGGAATCCACTAATGGAAGCTTA  1353
             ||||||||||||||||||||||||||||||||||||||||||||||||||||||||||||
Sbjct  1141  AACATTCTGGGAACGAATACAAAAGTAATGAACATGGAGGAATCCACTAATGGAAGCTTA  1200

Query  1354  GCAGCAGAGTTCAGGCATCTGCAATTAAAGGAACaaaaaaaTACAGGAAGCAGAACTAAT  1413
             ||||||||||||||||||||||||||||||||||||||||||||||||||||||||||||
Sbjct  1201  GCAGCAGAGTTCAGGCATCTGCAATTAAAGGAACAAAAAAATACAGGAAGCAGAACTAAT  1260

Query  1414  GAGGGTCCTCTCATTGTAACAGAAGAACTTCATTCCCTCAGCTTTGAAACTCAGCTGTGC  1473
             ||||||||||||||||||||||||||||||||||||||||||||||||||||||||||||
Sbjct  1261  GAGGGTCCTCTCATTGTAACAGAAGAACTTCATTCCCTCAGCTTTGAAACTCAGCTGTGC  1320

Query  1474  CAGCCTGGGCTGGTAGTAGATCTAGAGACCACATCCCTTCCCATTGTTGTGATCTCAAAT  1533
             ||||||||||||||||||||||||||||||||||||||||||||||||||||||||||||
Sbjct  1321  CAGCCTGGGCTGGTAGTAGATCTAGAGACCACATCCCTTCCCATTGTTGTGATCTCAAAT  1380

Query  1534  GTGAGCCAGCTTCCAAGTGGATGGGCTTCCATTTTGTGGTACAACATGTTGACTACTGAA  1593
             ||||||||||||||||||||||||||||||||||||||||||||||||||||||||||||
Sbjct  1381  GTGAGCCAGCTTCCAAGTGGATGGGCTTCCATTTTGTGGTACAACATGTTGACTACTGAA  1440

Query  1594  CCGAAGAACTTGTCTTTCTTTCTGAACCCACCTTGTGCAAGATGGTCTCAGCTTTCTGAA  1653
             ||||||||||||||||||||||||||||||||||||||||||||||||||||||||||||
Sbjct  1441  CCGAAGAACTTGTCTTTCTTTCTGAACCCACCTTGTGCAAGATGGTCTCAGCTTTCTGAA  1500

Query  1654  GTGCTGAGTTGGCAGTTCTCTTCCGTAACTAAAAGGGGACTTCATGCAGATCAGTTGAGC  1713
             ||||||||||||||||||||||||||||||||||||||||||||||||||||||||||||
Sbjct  1501  GTGCTGAGTTGGCAGTTCTCTTCCGTAACTAAAAGGGGACTTCATGCAGATCAGTTGAGC  1560

Query  1714  ATGCTGGGGGAGAAACTTCTTGGTCCAACAGGTGGAGGCTCTCTTGATGGCCTTATTCCT  1773
             ||||||||||||||||||||||||||||||||||||||||||||||||||||||||||||
Sbjct  1561  ATGCTGGGGGAGAAACTTCTTGGTCCAACAGGTGGAGGCTCTCTTGATGGCCTTATTCCT  1620

Query  1774  TGGACAAGATTCTGCAAGGAAAATATAAATGATAAAAATTTTCCCTTCTGGCTGTGGATT  1833
             ||||||||||||||||||||||||||||||||||||||||||||||||||||||||||||
Sbjct  1621  TGGACAAGATTCTGCAAGGAAAATATAAATGATAAAAATTTTCCCTTCTGGCTGTGGATT  1680

Query  1834  GAGGGCATCCTTGAACTCATTAAAAAACACCTCTTGTGTCTCTGGAATGATGGCTGTATC  1893
             ||||||||||||||||||||||||||||||||||||||||||||||||||||||||||||
Sbjct  1681  GAGGGCATCCTTGAACTCATTAAAAAACACCTCTTGTGTCTCTGGAATGATGGCTGTATC  1740

Query  1894  ATGGGTTTTATCAGTAAGGAGAGAGAACGTGCTTTGTTAAAGGACCAAAGGCCAGGGACT  1953
             ||||||||||||||||||||||||||||||||||||||||||||||||||||||||||||
Sbjct  1741  ATGGGTTTTATCAGTAAGGAGAGAGAACGTGCTTTGTTAAAGGACCAAAGGCCAGGGACT  1800

Query  1954  TTTTTACTGAGATTTAGTGAAAGTAGCCGAGAAGGAGCCATCACTTTTACCTGGGTAGAG  2013
             ||||||||||||||||||||||||||||||||||||||||||||||||||||||||||||
Sbjct  1801  TTTTTACTGAGATTTAGTGAAAGTAGCCGAGAAGGAGCCATCACTTTTACCTGGGTAGAG  1860

Query  2014  GGATCCCAAAATGAACCTCAGTTCCATTCGGTAGAACCTTATACCAAGAAGGAGCTCTCA  2073
             ||||||||||||||||||||||||||||||||||||||||||||||||||||||||||||
Sbjct  1861  GGATCCCAAAATGAACCTCAGTTCCATTCGGTAGAACCTTATACCAAGAAGGAGCTCTCA  1920

Query  2074  GCTGTTACTTTCCCTGATATCATTCGCAACTACAAAGTGATGGCGGCCGAAAATATTCCT  2133
             ||||||||||||||||||||||||||||||||||||||||||||||||||||||||||||
Sbjct  1921  GCTGTTACTTTCCCTGATATCATTCGCAACTACAAAGTGATGGCGGCCGAAAATATTCCT  1980

Query  2134  GAAAATCCACTGAGCTATCTGTACCCAAATATTCCCAAAGATAATGCCTTTGGGAAATAC  2193
             ||||||||||||||||||||||||||||||||||||||||||||||||||||||||||||
Sbjct  1981  GAAAATCCACTGAGCTATCTGTACCCAAATATTCCCAAAGATAATGCCTTTGGGAAATAC  2040

Query  2194  TACTCCAGACCCAAGGAGACCTCTGAGCCTATGGATTTAGATGGCCCCAAAGGGAATGGA  2253
             ||||||||||||||||||||||||||||||||||||||||||||||||||||||||||||
Sbjct  2041  TACTCCAGACCCAAGGAGACCTCTGAGCCTATGGATTTAGATGGCCCCAAAGGGAATGGA  2100

Query  2254  TACATCAAGACTGAATTAATCTCCGTATCTGAAGTCCACCCTTCCAGACTCCAGACCACA  2313
             ||||||||||||||||||||||||||||||||||||||||||||||||||||||||||||
Sbjct  2101  TACATCAAGACTGAATTAATCTCCGTATCTGAAGTCCACCCTTCCAGACTCCAGACCACA  2160

Query  2314  GAAAACCTCTTACCCATGTCTCCTGAAGATTTTGATGAGGTGTCTCGAATGGTGAGCCCT  2373
             |||||||||||||||||||||||||||||||||||||||||||||||||           
Sbjct  2161  GAAAACCTCTTACCCATGTCTCCTGAAGATTTTGATGAGGTGTCTCGAA-----------  2209

Query  2374  GCAGAAATTGATACGGTGATGTGTTCAGCCTATCCGACTTAAGTGTTATTCATCTCTCTA  2433
                             ||||||||||||||||||||||||||||||||||||||||||||
Sbjct  2210  ----------------TGATGTGTTCAGCCTATCCGACTTAAGTGTTATTCATCTCTCTA  2253

Query  2434  CTATACTGTTTGCACGTGTCCATCCTTAAGTAGCTTGCATTTTTCTGCTTCCCTGTTTCA  2493
             ||||||||||||||||||||||||||||||||||||||||||||||||||||||||||||
Sbjct  2254  CTATACTGTTTGCACGTGTCCATCCTTAAGTAGCTTGCATTTTTCTGCTTCCCTGTTTCA  2313

Query  2494  CAGCCTGCAGGGGTATGCATCAAGGCTGTGTAGTCTGTGAAAAGCTTTCCCTACATTCTG  2553
             ||||||||||||||||||||||||||||||||||||||||||||||||||||||||||||
Sbjct  2314  CAGCCTGCAGGGGTATGCATCAAGGCTGTGTAGTCTGTGAAAAGCTTTCCCTACATTCTG  2373

Query  2554  AAGAAATCCCTTGGACTGTGTGGCACACCAGTGCATCAGACTGGAGCTTCAGTTCAATTA  2613
             ||||||||||||||||||||||||||||||||||||||||||||||||||||||||||||
Sbjct  2374  AAGAAATCCCTTGGACTGTGTGGCACACCAGTGCATCAGACTGGAGCTTCAGTTCAATTA  2433

Query  2614  AGGAATTGAGGTTCTTAATGAGTTCAGTGTGAAAATGAATAATTCCCAGAGTAGCCTGTA  2673
             ||||||||||||||||||||||||||||||||||||||||||||||||||||||||||||
Sbjct  2434  AGGAATTGAGGTTCTTAATGAGTTCAGTGTGAAAATGAATAATTCCCAGAGTAGCCTGTA  2493

Query  2674  TGTTACTGATTGTGATATTACATTCATTTAATTGTAATTTTCAATGAGCTAGAGAATTTT  2733
             ||||||||||||||||||||||||||||||||||||||||||||||||||||||||||||
Sbjct  2494  TGTTACTGATTGTGATATTACATTCATTTAATTGTAATTTTCAATGAGCTAGAGAATTTT  2553

Query  2734  CTGTCACCACTGTGAAACTCCAGCACAGAAGGTAGAGGGGGGAAAAGCTGGAAATCCTAG  2793
             ||||||||||||||||||||||||||||||||||||||||||||||||||||||||||||
Sbjct  2554  CTGTCACCACTGTGAAACTCCAGCACAGAAGGTAGAGGGGGGAAAAGCTGGAAATCCTAG  2613

Query  2794  CAACAGGGTAGAGGACAGATGGGGCTCTTGGTTGGGAGAGCAGTGTGGGATTATGGGAGA  2853
             ||||||||||||||||||||||||||||||||||||||||||||||||||||||||||||
Sbjct  2614  CAACAGGGTAGAGGACAGATGGGGCTCTTGGTTGGGAGAGCAGTGTGGGATTATGGGAGA  2673

Query  2854  AGCCTGTGTTTCTACTAAATTTATCGTAAAGGAAATAGTTATGTTGACATTTACAATGTT  2913
             ||||||||||||||||||||||||||||||||||||||||||||||||||||||||||||
Sbjct  2674  AGCCTGTGTTTCTACTAAATTTATCGTAAAGGAAATAGTTATGTTGACATTTACAATGTT  2733

Query  2914  GGCTCTAGGTTTAAAGTAAAGGTAATTCACACCGCCGAGATTGGTTTCAGGCTGCCTGCA  2973
             ||||||||||||||||||||||||||||||||||||||||||||||||||||||||||||
Sbjct  2734  GGCTCTAGGTTTAAAGTAAAGGTAATTCACACCGCCGAGATTGGTTTCAGGCTGCCTGCA  2793

Query  2974  TAATCAGGGAGACAATAAGCTTCAGTTTGCACTTTGCTCACACTTTGAAGGCTTTCTTCT  3033
             ||||||||||||||||||||||||||||||||||||||||||||||||||||||||||||
Sbjct  2794  TAATCAGGGAGACAATAAGCTTCAGTTTGCACTTTGCTCACACTTTGAAGGCTTTCTTCT  2853

Query  3034  GCAATGAGGAGGGCTGGAATATTTTCTTTTAAATGAAAGCTTAAATACTTTCTCAAAGAA  3093
             ||||||||||||||||||||||||||||||||||||||||||||||||||||||||||||
Sbjct  2854  GCAATGAGGAGGGCTGGAATATTTTCTTTTAAATGAAAGCTTAAATACTTTCTCAAAGAA  2913

Query  3094  TACAGCCACAAACTGCACTGACCTGTGTTTGCTTCTGCTCTGCTAAATCAGTGGCATATC  3153
             ||||||||||||||||||||||||||||||||||||||||||||||||||||||||||||
Sbjct  2914  TACAGCCACAAACTGCACTGACCTGTGTTTGCTTCTGCTCTGCTAAATCAGTGGCATATC  2973

Query  3154  CTTCACTGTAAATAAAAACTAGCATGTTATTAGGGTAAGTGTGAAATCCACAACAGTTAC  3213
             ||||||||||||||||||||||||||||||||||||||||||||||||||||||||||||
Sbjct  2974  CTTCACTGTAAATAAAAACTAGCATGTTATTAGGGTAAGTGTGAAATCCACAACAGTTAC  3033

Query  3214  TGTACTGTTCATTGAGAAAAGAAACACCAATTCAATAGGATGTCTCACAAAATCTTTTTC  3273
             ||||||||||||||||||||||||||||||||||||||||||||||||||||||||||||
Sbjct  3034  TGTACTGTTCATTGAGAAAAGAAACACCAATTCAATAGGATGTCTCACAAAATCTTTTTC  3093

Query  3274  ATGTTGAAAATCCACAAAGGCATCATGTCACATGCTGAACAGTGTTTGAGCATGCGCTGC  3333
             ||||||||||||||||||||||||||||||||||||||||||||||||||||||||||||
Sbjct  3094  ATGTTGAAAATCCACAAAGGCATCATGTCACATGCTGAACAGTGTTTGAGCATGCGCTGC  3153

Query  3334  ACATTCTGCTTTCTATTACGGCGTAAAGGAATTGAAAGGTAGACGTAGAATTACAGCCAG  3393
             ||||||||||||||||||||||||||||||||||||||||||||||||||||||||||||
Sbjct  3154  ACATTCTGCTTTCTATTACGGCGTAAAGGAATTGAAAGGTAGACGTAGAATTACAGCCAG  3213

Query  3394  TACCTTACTGAAGTACAGTTCAATAAAATCTGCGTGAGGCAGCAGAGAGATGTTTTCTCA  3453
             ||||||||||||||||||||||||||||||||||||||||||||||||||||||||||||
Sbjct  3214  TACCTTACTGAAGTACAGTTCAATAAAATCTGCGTGAGGCAGCAGAGAGATGTTTTCTCA  3273

Query  3454  GAGGATCCTTAAGACAAGCACTGCTCTCCCTCCAACCATCACTGCAAGCTGACTGCACAG  3513
             ||||||||||||||||||||||||||||||||||||||||||||||||||||||||||||
Sbjct  3274  GAGGATCCTTAAGACAAGCACTGCTCTCCCTCCAACCATCACTGCAAGCTGACTGCACAG  3333

Query  3514  ACACCAGGTACTACCCCTCTGCCGGTGGTTACTTATGGAGGTGGAAGTGCTTTGATATTG  3573
             ||||||||||||||||||||||||||||||||||||||||||||||||||||||||||||
Sbjct  3334  ACACCAGGTACTACCCCTCTGCCGGTGGTTACTTATGGAGGTGGAAGTGCTTTGATATTG  3393

Query  3574  TCAGACTTATTCATCATTGTCCACTGTGAAAATACATGTGTCCACTAAGTATTCACAAAG  3633
             ||||||||||||||||||||||||||||||||||||||||||||||||||||||||||||
Sbjct  3394  TCAGACTTATTCATCATTGTCCACTGTGAAAATACATGTGTCCACTAAGTATTCACAAAG  3453

Query  3634  CACATAAAAGTTTAATTTTTAAGTAACCAGAACTAAAATACATTGAATGGGGCTCCTTCG  3693
             ||||||||||||||||||||||||||||||||||||||||||||||||||||||||||||
Sbjct  3454  CACATAAAAGTTTAATTTTTAAGTAACCAGAACTAAAATACATTGAATGGGGCTCCTTCG  3513

Query  3694  TTGTGACACACCTGCCAGCCAAACAAAGAGGAAATATTCTAACCCTGCTCCTGGCCAGGG  3753
             ||||||||||||||||||||||||||||||||||||||||||||||||||||||||||||
Sbjct  3514  TTGTGACACACCTGCCAGCCAAACAAAGAGGAAATATTCTAACCCTGCTCCTGGCCAGGG  3573

Query  3754  GATTACGCCTGAAAAGTAAATTGAGAATTGGaaaaaaaaaaaaa  3797
             ||||||||||||||||||||||||||||||||||||||||||||
Sbjct  3574  GATTACGCCTGAAAAGTAAATTGAGAATTGGAAAAAAAAAAAAA  3617
```

Related Information

Gene - associated gene d

Genome Data Viewer - ali

genomic context
