# Supplementary material for: Divergent IL18-STAT1 Immune Responses Underlie Differential Susceptibility to Aeromonas hydrophila in Geoclemys hamiltonii and Trachemys scripta: A Comparative Transcriptomic Perspective
Source: Genes (Basel). 2026 Apr 9;17(4):436. doi: 10.3390/genes17040436 (PMC13116093; doi:10.3390/genes17040436)
Supplement: Supplementary file 1 [file genes-17-00436-s001.zip › Figure S2/TLR5.pdf]

PREDICTED: Trachemys scripta elegans toll like receptor 5 (TLR5), transcript variant X1, mRNA

Sequence ID: XM_034764647.1 Length: 4014 Number of Matches: 2

Range 1: 339 to 4014 GenBank Graphics ▼ Next Match ▲ Previous Match

Score	Expect	Identities	Gaps	Strand
6737 bits(3648)	0.0	3676/3687(99%)	11/3687(0%)	Plus/Plus

```
Query  596    AGCTGCCTTTCATCCTGGTTATTCAACTATCATCTACAGCACCTGCCTGTTTACAGTAAA  655
              ||||||||||||||||||||||||||||||||||||||||||||||||||||||||||||
Sbjct  339    AGCTGCCTTTCATCCTGGTTATTCAACTATCATCTACAGCACCTGCCTGTTTACAGTAAA  398

Query  656    AGCAAGTAAGTTACAGCATCACAATGTTACATCATCTAGTATTTCTCTTAGGAATGTCAC  715
              |||||           ||||||||||||||||||||||||||||||||||||||||||||
Sbjct  399    AGCAA-----------CATCACAATGTTACATCATCTAGTATTTCTCTTAGGAATGTCAC  447

Query  716    TGGTAGCCAAAGAAATATTTGCATTTACAAACTGCTATTCCGATGGCCAAATTGCCAGGT  775
              ||||||||||||||||||||||||||||||||||||||||||||||||||||||||||||
Sbjct  448    TGGTAGCCAAAGAAATATTTGCATTTACAAACTGCTATTCCGATGGCCAAATTGCCAGGT  507

Query  776    ATTATTTTGTAACCTCACCGAGGTTCCACCTGTGCCCAATAATACAGTTATACTCTGGC  835
              ||||||||||||||||||||||||||||||||||||||||||||||||||||||||||||
Sbjct  508    ATTATTTTGTAACCTCACCGAGGTTCCACCTGTGCCCAATAATACAGTTATACTCTGGC  567

Query  836    TAAATTTCAACAAAATCAGGCAAGTGAATGCATCCTCCTTCCCTCTGCTGGAACAGTTGC  895
              ||||||||||||||||||||||||||||||||||||||||||||||||||||||||||||
Sbjct  568    TAAATTTCAACAAAATCAGGCAAGTGAATGCATCCTCCTTCCCTCTGCTGGAACAGTTGC  627

Query  896    AGATTTTGGAAATTGGAACCCAGCTTGTCTCTTCCGTTACCATAGGGAAAGCAGCTTTTA  955
              ||||||||||||||||||||||||||||||||||||||||||||||||||||||||||||
Sbjct  628    AGATTTTGGAAATTGGAACCCAGCTTGTCTCTTCCGTTACCATAGGGAAAGCAGCTTTTA  687

Query  956    GGAACCTGCCAAACCTTCGAAACTTAGATTTAGGAGACAATAAGATACTTCATCTCGATC  1015
              ||||||||||||||||||||||||||||||||||||||||||||||||||||||||||||
Sbjct  688    GGAACCTGCCAAACCTTCGAAACTTAGATTTAGGAGACAATAAGATACTTCATCTCGATC  747

Query  1016   CTGATGCTTTTGTGAAGTTGTCAAATGTACAAATACTCCAGCTATATCACAACAGTCTTG  1075
              ||||||||||||||||||||||||||||||||||||||||||||||||||||||||||||
Sbjct  748    CTGATGCTTTTGTGAAGTTGTCAAATGTACAAATACTCCAGCTATATCACAACAGTCTTG  807

Query  1076   AGGAGTCCATTCTGGAAGAAGACTATCTTCGAGATATGATCTCCTTAGAATATTTGGATC  1135
              ||||||||||||||||||||||||||||||||||||||||||||||||||||||||||||
Sbjct  808    AGGAGTCCATTCTGGAAGAAGACTATCTTCGAGATATGATCTCCTTAGAATATTTGGATC  867

Query  1136   TTTCTGGAAACAAGATCAAAAGCCTTCGCCCTCATCGCTTATTTACCGTCTAAAATCCT  1195
              ||||||||||||||||||||||||||||||||||||||||||||||||||||||||||||
Sbjct  868    TTTCTGGAAACAAGATCAAAAGCCTTCGCCCTCATCGCTTATTTACCGTCTAAAATCCT  927

Query  1196   TGCAAATTGTGAACCTGAAAAACAACTGGATACCCATCTTATGTGAAGGAAACCTTGATA  1255
              ||||||||||||||||||||||||||||||||||||||||||||||||||||||||||||
Sbjct  928    TGCAAATTGTGAACCTGAAAAACAACTGGATACCCATCTTATGTGAAGGAAACCTTGATA  987

Query  1256   GCTTCCAGGGAAAATTCTTCACATTGTTTATTCTCAATTTTAATAAATTATACTACCCAA  1315
              ||||||||||||||||||||||||||||||||||||||||||||||||||||||||||||
Sbjct  988    GCTTCCAGGGAAAATTCTTCACATTGTTTATTCTCAATTTTAATAAATTATACTACCCAA  1047

Query  1316   TTTCTATGGACTGGGCCAAGTGTGGAAATCCTTTCAAAAACATAGCCCTGGACACCCTGG  1375
              ||||||||||||||||||||||||||||||||||||||||||||||||||||||||||||
Sbjct  1048   TTTCTATGGACTGGGCCAAGTGTGGAAATCCTTTCAAAAACATAGCCCTGGACACCCTGG  1107

Query  1376   ATCTTGGTGGCAATGGCTGGGGTGTAGATATAGTGCAACACTTCTGCACAGCTGTGAATG  1435
              ||||||||||||||||||||||||||||||||||||||||||||||||||||||||||||
Sbjct  1108   ATCTTGGTGGCAATGGCTGGGGTGTAGATATAGTGCAACACTTCTGCACAGCTGTGAATG  1167

Query  1436   GGACTTCAATTGtttttttGAAGCTTAGCCATCACATAATGGGTCCAGGATTTGGCTTTA  1495
              ||||||||||||||||||||||||||||||||||||||||||||||||||||||||||||
Sbjct  1168   GGACTTCAATTGTTTTTTTGAAGCTTAGCCATCACATAATGGGTCCAGGATTTGGCTTTA  1227

Query  1496   AGAACTTGAAAGATCCAGACCAAGATACATTTGCAGGGCTAGCAAGAAGTGGCGTTCGCT  1555
              ||||||||||||||||||||||||||||||||||||||||||||||||||||||||||||
Sbjct  1228   AGAACTTGAAAGATCCAGACCAAGATACATTTGCAGGGCTAGCAAGAAGTGGCGTTCGCT  1287

Query  1556   TACTGGATATTTCACATGGTTTTATTTTCTCTCTCAATCCTTATGTATTTCAGAGCCTTG  1615
              ||||||||||||||||||||||||||||||||||||||||||||||||||||||||||||
Sbjct  1288   TACTGGATATTTCACATGGTTTTATTTTCTCTCTCAATCCTTATGTATTTCAGAGCCTTG  1347

Query  1616   GTGATCTGGAATTGCTGGACCTTCACAACAACAAGATAAATCAGATCCAAAAACAAGCAT  1675
              ||||||||||||||||||||||||||||||||||||||||||||||||||||||||||||
Sbjct  1348   GTGATCTGGAATTGCTGGACCTTCACAACAACAAGATAAATCAGATCCAAAAACAAGCAT  1407

Query  1676   TTTTTGGTCTGGGAAACCTAGGAACTCTCAACCTGTCATATAACATTCTGGGGGAGCTGT  1735
              ||||||||||||||||||||||||||||||||||||||||||||||||||||||||||||
Sbjct  1408   TTTTTGGTCTGGGAAACCTAGGAACTCTCAACCTGTCATATAACATTCTGGGGGAGCTGT  1467

Query  1736   ACGATTATACTTTTGAGGGGCTTCAGAATGTGATGCAGATTGATTTGCAACAAAATCATA  1795
              ||||||||||||||||||||||||||||||||||||||||||||||||||||||||||||
Sbjct  1468   ACGATTATACTTTTGAGGGGCTTCAGAATGTGATGCAGATTGATTTGCAACAAAATCATA  1527

Query  1796   TTGGAGTAATTGCTGGGAATTCATTCAGGGATTTAAGAAGGTTAAAGTTGGTGGATCTCC  1855
              ||||||||||||||||||||||||||||||||||||||||||||||||||||||||||||
Sbjct  1528   TTGGAGTAATTGCTGGGAATTCATTCAGGGATTTAAGAAGGTTAAAGTTGGTGGATCTCC  1587

Query  1856   GGGACAATGCCATTAAAACTCTCCCTTCTTTCCCAGCCATGATCACTCTTCATTTAAGTG  1915
              ||||||||||||||||||||||||||||||||||||||||||||||||||||||||||||
Sbjct  1588   GGGACAATGCCATTAAAACTCTCCCTTCTTTCCCAGCCATGATCACTCTTCATTTAAGTG  1647

Query  1916   ACAATAAGCTATTATCTGTAAGTAACCAGAGAATAAATGCAACAATCCTTATCTTGGAAA  1975
              ||||||||||||||||||||||||||||||||||||||||||||||||||||||||||||
Sbjct  1648   ACAATAAGCTATTATCTGTAAGTAACCAGAGAATAAATGCAACAATCCTTATCTTGGAAA  1707

Query  1976   GAAACAGACTGGACAATCTGGGTGATCTTTATATTCTTTTACAAGTTCCAGATGTGAAGT  2035
              ||||||||||||||||||||||||||||||||||||||||||||||||||||||||||||
Sbjct  1708   GAAACAGACTGGACAATCTGGGTGATCTTTATATTCTTTTACAAGTTCCAGATGTGAAGT  1767

Query  2036   ATATCTTGTTAAGACAAAATCGTTTATCTTATTGTGTTAAAAGTGTTGATGTTATAGAAA  2095
              ||||||||||||||||||||||||||||||||||||||||||||||||||||||||||||
Sbjct  1768   ATATCTTGTTAAGACAAAATCGTTTATCTTATTGTGTTAAAAGTGTTGATGTTATAGAAA  1827

Query  2096   ATAACCAGTTAGTCTACTTGGATCTAGGAGAAAACATGTTAAAGCTTGTGTGGGACAGAG  2155
              ||||||||||||||||||||||||||||||||||||||||||||||||||||||||||||
Sbjct  1828   ATAACCAGTTAGTCTACTTGGATCTAGGAGAAAACATGTTAAAGCTTGTGTGGGACAGAG  1887

Query  2156   GTTTATGTTTGGATGTGTTCAGGCGACTTTCCAAACTAGAGGTGCTCCACCTGAATAACA  2215
              ||||||||||||||||||||||||||||||||||||||||||||||||||||||||||||
Sbjct  1888   GTTTATGTTTGGATGTGTTCAGGCGACTTTCCAAACTAGAGGTGCTCCACCTGAATAACA  1947

Query  2216   ACTACCTTACTATCCTTCCACAGGATATTTTTAGTGGTCTAACATCATTAAACAGACTTA  2275
              ||||||||||||||||||||||||||||||||||||||||||||||||||||||||||||
Sbjct  1948   ACTACCTTACTATCCTTCCACAGGATATTTTTAGTGGTCTAACATCATTAAACAGACTTA  2007

Query  2276   ACCTAGCCTCCAACCTGTTGTCTTATCTTTCTCCTGGTGTTTTCCCTGAGAGCCTAAAGA  2335
              ||||||||||||||||||||||||||||||||||||||||||||||||||||||||||||
Sbjct  2008   ACCTAGCCTCCAACCTGTTGTCTTATCTTTCTCCTGGTGTTTTCCCTGAGAGCCTAAAGA  2067

Query  2336   CACTTAATATGTCTGAAAACCAACTTCTTTCACCTGCCCTTGAGCTCTTCATGACTTTGA  2395
              ||||||||||||||||||||||||||||||||||||||||||||||||||||||||||||
Sbjct  2068   CACTTAATATGTCTGAAAACCAACTTCTTTCACCTGCCCTTGAGCTCTTCATGACTTTGA  2127

Query  2396   GTATCCTGGATATAACAAATAACAGGTTTTTCTGTGATTGCAGTTTAAACACCTGGACAG  2455
              ||||||||||||||||||||||||||||||||||||||||||||||||||||||||||||
Sbjct  2128   GTATCCTGGATATAACAAATAACAGGTTTTTCTGTGATTGCAGTTTAAACACCTGGACAG  2187

Query  2456   CACGGTTAAATCAAACCAATGTGACCTTAGCTGGCTCAGAAAATGACACATACTGTATAC  2515
              ||||||||||||||||||||||||||||||||||||||||||||||||||||||||||||
Sbjct  2188   CACGGTTAAATCAAACCAATGTGACCTTAGCTGGCTCAGAAAATGACACATACTGTATAC  2247

Query  2516   TCCCACCTTTTTTAACCAGGGTTCCACTCTCTTCAGTGGCACTTGATGGCTGTAATGAAG  2575
              ||||||||||||||||||||||||||||||||||||||||||||||||||||||||||||
Sbjct  2248   TCCCACCTTTTTTAACCAGGGTTCCACTCTCTTCAGTGGCACTTGATGGCTGTAATGAAG  2307

Query  2576   ACGAACTCCAGAAGCCTCTACAGTTCTCACTGTTCATCTTCACTTCAGTCACTCTGATAA  2635
              ||||||||||||||||||||||||||||||||||||||||||||||||||||||||||||
Sbjct  2308   ACGAACTCCAGAAGCCTCTACAGTTCTCACTGTTCATCTTCACTTCAGTCACTCTGATAA  2367

Query  2636   TGTTCCTAACAGCAGTCATCATTTTTAGTCACTTTCGGGGGACTTGTTTTGTCTGGTATA  2695
              ||||||||||||||||||||||||||||||||||||||||||||||||||||||||||||
Sbjct  2368   TGTTCCTAACAGCAGTCATCATTTTTAGTCACTTTCGGGGGACTTGTTTTGTCTGGTATA  2427

Query  2696   AGACCATCAAAGGTGCTATGCTAAAAGAACGTAAGCAAGCAATAGATACAAGTGCATATC  2755
              ||||||||||||||||||||||||||||||||||||||||||||||||||||||||||||
Sbjct  2428   AGACCATCAAAGGTGCTATGCTAAAAGAACGTAAGCAAGCAATAGATACAAGTGCATATC  2487

Query  2756   AATATGATGCATATTTATGCTACAGCAACAGAGACTTTGAGTGGGTCCAAAATTCATTGA  2815
              ||||||||||||||||||||||||||||||||||||||||||||||||||||||||||||
Sbjct  2488   AATATGATGCATATTTATGCTACAGCAACAGAGACTTTGAGTGGGTCCAAAATTCATTGA  2547

Query  2816   TAAAGCACCTGGACTCTCAGTACTCTGAGAAAAACAGATTTACTTTGTGCTTTGAAGAGA  2875
              ||||||||||||||||||||||||||||||||||||||||||||||||||||||||||||
Sbjct  2548   TAAAGCACCTGGACTCTCAGTACTCTGAGAAAAACAGATTTACTTTGTGCTTTGAAGAGA  2607

Query  2876   GAGATTTCCTGCCTGGGGAGGAACAGATCACCAACATCCGTGATGCCATTTGGAACAGCA  2935
              ||||||||||||||||||||||||||||||||||||||||||||||||||||||||||||
Sbjct  2608   GAGATTTCCTGCCTGGGGAGGAACAGATCACCAACATCCGTGATGCCATTTGGAACAGCA  2667

Query  2936   GGAAGACCATTTGCATTGTGACAAGGCAGTTCCTCAAGGATGGGTGGTGCGTGGAAGCCT  2995
              ||||||||||||||||||||||||||||||||||||||||||||||||||||||||||||
Sbjct  2668   GGAAGACCATTTGCATTGTGACAAGGCAGTTCCTCAAGGATGGGTGGTGCGTGGAAGCCT  2727

Query  2996   TTAATTTTGCCCAGAGCAGATACTTTTGTGACCTGAAAGATGTCCTCATTATGGTTGTGG  3055
              ||||||||||||||||||||||||||||||||||||||||||||||||||||||||||||
Sbjct  2728   TTAATTTTGCCCAGAGCAGATACTTTTGTGACCTGAAAGATGTCCTCATTATGGTTGTGG  2787

Query  3056   TTGGGTCACTTTCTCAGTATCAGTTGATGAAATACAAACCGATTAGAGTCTTTGTGCAAA  3115
              ||||||||||||||||||||||||||||||||||||||||||||||||||||||||||||
Sbjct  2788   TTGGGTCACTTTCTCAGTATCAGTTGATGAAATACAAACCGATTAGAGTCTTTGTGCAAA  2847

Query  3116   GGAGTCAGTACATGCAGTGGCCTGAAGACCATCAAGATGTAGACTGGTTTTTAAATACCC  3175
              ||||||||||||||||||||||||||||||||||||||||||||||||||||||||||||
Sbjct  2848   GGAGTCAGTACATGCAGTGGCCTGAAGACCATCAAGATGTAGACTGGTTTTTAAATACCC  2907

Query  3176   TTTCTCACCAAATTCTGAAAGaaaaaaaaGTGAAAAAGAAATCCAGTGTTATAGAAATGC  3235
              ||||||||||||||||||||||||||||||||||||||||||||||||||||||||||||
Sbjct  2908   TTTCTCACCAAATTCTGAAAGAAAAAAAAGTGAAAAAGAAATCCAGTGTTATAGAAATGC  2967

Query  3236   AAACTGTAAGGACAATCTCGTAGTTGGGAGGGTTGTTCATGGTTCATATTATTAGTGCTA  3295
              ||||||||||||||||||||||||||||||||||||||||||||||||||||||||||||
Sbjct  2968   AAACTGTAAGGACAATCTCGTAGTTGGGAGGGTTGTTCATGGTTCATATTATTAGTGCTA  3027

Query  3296   TAAAACAGATTTTTGTAGTTACATTTTTGCTTGTGGCTTGACAGTTACTTAAATATTCTA  3355
              ||||||||||||||||||||||||||||||||||||||||||||||||||||||||||||
Sbjct  3028   TAAAACAGATTTTTGTAGTTACATTTTTGCTTGTGGCTTGACAGTTACTTAAATATTCTA  3087

Query  3356   AATCCATTTCCACCTGTGGCATGGCAGGAAAGAAGTATGGGCCATGCTTCAGCTTTCCCT  3415
              ||||||||||||||||||||||||||||||||||||||||||||||||||||||||||||
Sbjct  3088   AATCCATTTCCACCTGTGGCATGGCAGGAAAGAAGTATGGGCCATGCTTCAGCTTTCCCT  3147

Query  3416   GACAAATGGATAGAATATAACTTCCTTGGTTTAAGTTTTAATAAATATGGCGTCAGCTTT  3475
              ||||||||||||||||||||||||||||||||||||||||||||||||||||||||||||
Sbjct  3148   GACAAATGGATAGAATATAACTTCCTTGGTTTAAGTTTTAATAAATATGGCGTCAGCTTT  3207

Query  3476   AAAGTCACCAGCAAATGGAATTAACAAAATTCAATCACTCTAGGAACTATGGCCCATATT  3535
              ||||||||||||||||||||||||||||||||||||||||||||||||||||||||||||
Sbjct  3208   AAAGTCACCAGCAAATGGAATTAACAAAATTCAATCACTCTAGGAACTATGGCCCATATT  3267

Query  3536   TTCAAAAGAAGTCACCAAGTATGGGTGTCTCAAATTTTGGGTGTTCAGATTAGGACACCA  3595
              ||||||||||||||||||||||||||||||||||||||||||||||||||||||||||||
Sbjct  3268   TTCAAAAGAAGTCACCAAGTATGGGTGTCTCAAATTTTGGGTGTTCAGATTAGGACACCA  3327

Query  3596   TGGTCTGATTTTCAGAGATGCTGAGTGCTCACAGGTCCCCACGACAATCGAAGGAGTGGG  3655
              ||||||||||||||||||||||||||||||||||||||||||||||||||||||||||||
Sbjct  3328   TGGTCTGATTTTCAGAGATGCTGAGTGCTCACAGGTCCCCACGACAATCGAAGGAGTGGG  3387

Query  3656   TACTCAGCAATTACAAAAAACAGGTGCATGTTATTGCAAGTTAGGCACCAAATTTATAGG  3715
              ||||||||||||||||||||||||||||||||||||||||||||||||||||||||||||
Sbjct  3388   TACTCAGCAATTACAAAAAACAGGTGCATGTTATTGCAAGTTAGGCACCAAATTTATAGG  3447

Query  3716   ATTCTTGAAAATTTGGGTCCATGTGCTGAAAGCCCCCACTAACCAAACTGAATGGGCACA  3775
              ||||||||||||||||||||||||||||||||||||||||||||||||||||||||||||
Sbjct  3448   ATTCTTGAAAATTTGGGTCCATGTGCTGAAAGCCCCCACTAACCAAACTGAATGGGCACA  3507

Query  3776   TTAACTGATGGCAGTGCCACATTAAGGTCTTGCAATGTCTAGTCCTACCATTCACCACCA  3835
              ||||||||||||||||||||||||||||||||||||||||||||||||||||||||||||
Sbjct  3508   TTAACTGATGGCAGTGCCACATTAAGGTCTTGCAATGTCTAGTCCTACCATTCACCACCA  3567

Query  3836   GGTCCCCTACTAAGAGAATGTTTTAGTGGTCATCATCCTTGCTTGTGATGTTGCATGATC  3895
              ||||||||||||||||||||||||||||||||||||||||||||||||||||||||||||
Sbjct  3568   GGTCCCCTACTAAGAGAATGTTTTAGTGGTCATCATCCTTGCTTGTGATGTTGCATGATC  3627

Query  3896   AAGATGGTGTAGGTGTAGGTGTTGGGGAAGTCATGTACATGTCGCTCACATACACCCACA  3955
              ||||||||||||||||||||||||||||||||||||||||||||||||||||||||||||
Sbjct  3628   AAGATGGTGTAGGTGTAGGTGTTGGGGAAGTCATGTACATGTCGCTCACATACACCCACA  3687

Query  3956   CACCCACTATTGGGTACCATTTTTCACAGAACATCATCATTTTGTAAAGCCACACCACAT  4015
              ||||||||||||||||||||||||||||||||||||||||||||||||||||||||||||
Sbjct  3688   CACCCACTATTGGGTACCATTTTTCACAGAACATCATCATTTTGTAAAGCCACACCACAT  3747

Query  4016   AGTATTCtttttttttattattattatttttttttttaAGGGAGGGATTTACCTCTTCCT  4075
              ||||||||||||||||||||||||||||||||||||||||||||||||||||||||||||
Sbjct  3748   AGTATTCTTTTTTTTTATTATTATTATTTTTTTTTTTAAGGGAGGGATTTACCTCTTCCT  3807

Query  4076   TAAAGCCAGGCTTGGAAGGATCACATTTTTATCAGTAAATGTCACTAAACATCAATTTCA  4135
              ||||||||||||||||||||||||||||||||||||||||||||||||||||||||||||
Sbjct  3808   TAAAGCCAGGCTTGGAAGGATCACATTTTTATCAGTAAATGTCACTAAACATCAATTTCA  3867

Query  4136   CCGTACATGTACAAACCAACCCAACATTTCCATAATAATAAATGACATTTAGAAATAGAC  4195
              ||||||||||||||||||||||||||||||||||||||||||||||||||||||||||||
Sbjct  3868   CCGTACATGTACAAACCAACCCAACATTTCCATAATAATAAATGACATTTAGAAATAGAC  3927

Query  4196   CAAGAAAGAAAAATGCTGCTTAAGAACTTAATAGAGTTTGATTTAAGGATATTTACTTTG  4255
              ||||||||||||||||||||||||||||||||||||||||||||||||||||||||||||
Sbjct  3928   CAAGAAAGAAAAATGCTGCTTAAGAACTTAATAGAGTTTGATTTAAGGATATTTACTTTG  3987

Query  4256   TATATTTTGATATGTGATGTTGACAAT  4282
              |||||||||||||||||||||||||||
Sbjct  3988   TATATTTTGATATGTGATGTTGACAAT  4014
```

Range 2: 1 to 340 GenBank Graphics ▼ Next Match ▲ Previous Match ▲ First Match

Score	Expect	Identities	Gaps	Strand
628 bits(340)	0.0	340/340(100%)	0/340(0%)	Plus/Plus

```
Query  146    GGGCTGGGCTAGGCGAGCCCGGCGGAGGGTGTCCCCCGCCCCGCGCCAGTCGAGGCGGAG  205
              ||||||||||||||||||||||||||||||||||||||||||||||||||||||||||||
Sbjct  1      GGGCTGGGCTAGGCGAGCCCGGCGGAGGGTGTCCCCCGCCCCGCGCCAGTCGAGGCGGAG  60

Query  206    CAGGGTTGGGGAACCCGGGCACGGAGGTGAAGCCCTGGCCGAGAATCCCTTCCCTGGCGG  265
              ||||||||||||||||||||||||||||||||||||||||||||||||||||||||||||
Sbjct  61     CAGGGTTGGGGAACCCGGGCACGGAGGTGAAGCCCTGGCCGAGAATCCCTTCCCTGGCGG  120

Query  266    GCGGGCCGAGGCTTCGCGGAGAGGCTGGTGGCCGCCGCCGCCTGCAGCGCAGCCCTTTTC  325
              ||||||||||||||||||||||||||||||||||||||||||||||||||||||||||||
Sbjct  121    GCGGGCCGAGGCTTCGCGGAGAGGCTGGTGGCCGCCGCCGCCTGCAGCGCAGCCCTTTTC  180

Query  326    CAGGGGCTCGCTCGGCTGAGCTGCCCGCGGAGCCGGACCCCGGCGCTGCGGCCCCGGGCG  385
              ||||||||||||||||||||||||||||||||||||||||||||||||||||||||||||
Sbjct  181    CAGGGGCTCGCTCGGCTGAGCTGCCCGCGGAGCCGGACCCCGGCGCTGCGGCCCCGGGCG  240

Query  386    GAGACTGGAAACAGTTAGGTGAACCTTCCTCCTCTCTGCCCCTACTCCTGGGAATGCTGG  445
              ||||||||||||||||||||||||||||||||||||||||||||||||||||||||||||
Sbjct  241    GAGACTGGAAACAGTTAGGTGAACCTTCCTCCTCTCTGCCCCTACTCCTGGGAATGCTGG  300

Query  446    GGCTTTATGTAAAATGGAATCTGGTTTGGAACCAGCTCAG  485
              ||||||||||||||||||||||||||||||||||||||||
Sbjct  301    GGCTTTATGTAAAATGGAATCTGGTTTGGAACCAGCTCAG  340
```
