# Supplementary material for: Divergent IL18-STAT1 Immune Responses Underlie Differential Susceptibility to Aeromonas hydrophila in Geoclemys hamiltonii and Trachemys scripta: A Comparative Transcriptomic Perspective
Source: Genes (Basel). 2026 Apr 9;17(4):436. doi: 10.3390/genes17040436 (PMC13116093; doi:10.3390/genes17040436)
Supplement: Supplementary file 1 [file genes-17-00436-s001.zip › Figure S2/TNFSF13B.pdf]

PREDICTED: Trachemys scripta elegans TNF superfamily member 13b (TNFSF13B), mRNA

Sequence ID: XM_034758181.1 Length: 1544 Number of Matches: 1

Range 1: 1 to 1544 GenBank Graphics ▼ Next Match ▲ Previous Match

Score	Expect	Identities	Gaps	Strand
2852 bits(1544)	0.0	1544/1544(100%)	0/1544(0%)	Plus/Plus

```
Query  1     AAGCTTTTTTCTGTTTTTAGGATACAGGATAATGATAGAAATTCTGTCAGTCCATGCACT  60
             ||||||||||||||||||||||||||||||||||||||||||||||||||||||||||||
Sbjct  1     AAGCTTTTTTCTGTTTTTAGGATACAGGATAATGATAGAAATTCTGTCAGTCCATGCACT  60

Query  61    GACATACATTCCATTTTCTTGAGAACGACTGTATATTTCTATTTTTAGAGACCCCATTAT  120
             ||||||||||||||||||||||||||||||||||||||||||||||||||||||||||||
Sbjct  61    GACATACATTCCATTTTCTTGAGAACGACTGTATATTTCTATTTTTAGAGACCCCATTAT  120

Query  121   GTGTTAGGTTGTGACAGGATACAATACTTTGGCAGGGAGTCTTACATCTCTTCTGCACTA  180
             ||||||||||||||||||||||||||||||||||||||||||||||||||||||||||||
Sbjct  121   GTGTTAGGTTGTGACAGGATACAATACTTTGGCAGGGAGTCTTACATCTCTTCTGCACTA  180

Query  181   TTTCTGAAGATTCAGGAAACAAATAGTATCAGATGTTTTAATCATGTCTGGGTACAAAGA  240
             ||||||||||||||||||||||||||||||||||||||||||||||||||||||||||||
Sbjct  181   TTTCTGAAGATTCAGGAAACAAATAGTATCAGATGTTTTAATCATGTCTGGGTACAAAGA  240

Query  241   TAACAGGAGATGGTAATGGGCAAGTTGCCATAAAAAGTAAATAGCTGAAAGTGAAATGAG  300
             ||||||||||||||||||||||||||||||||||||||||||||||||||||||||||||
Sbjct  241   TAACAGGAGATGGTAATGGGCAAGTTGCCATAAAAAGTAAATAGCTGAAAGTGAAATGAG  300

Query  301   GAAGGTAGATTGAGCAAGCAGCTGAAGAGAGAATAATAATTTGCCAAGATGCAGAGCAGT  360
             ||||||||||||||||||||||||||||||||||||||||||||||||||||||||||||
Sbjct  301   GAAGGTAGATTGAGCAAGCAGCTGAAGAGAGAATAATAATTTGCCAAGATGCAGAGCAGT  360

Query  361   AAGAAGAAAGCTAAGGGAGCCTGCATCTTAGACACTTACCCTGTATATTAAACTTAATGT  420
             ||||||||||||||||||||||||||||||||||||||||||||||||||||||||||||
Sbjct  361   AAGAAGAAAGCTAAGGGAGCCTGCATCTTAGACACTTACCCTGTATATTAAACTTAATGT  420

Query  421   TTGTATCAACAGCTCCATATGACAGGAAATGATCTGGGCGTTTTGGTCAGATATTTTGAC  480
             ||||||||||||||||||||||||||||||||||||||||||||||||||||||||||||
Sbjct  421   TTGTATCAACAGCTCCATATGACAGGAAATGATCTGGGCGTTTTGGTCAGATATTTTGAC  480

Query  481   TGCCCTTAGTTTCAGCCAGCAACTGGCAAAATGGATTACACCCCAGAAGGAGACAGAGCT  540
             ||||||||||||||||||||||||||||||||||||||||||||||||||||||||||||
Sbjct  481   TGCCCTTAGTTTCAGCCAGCAACTGGCAAAATGGATTACACCCCAGAAGGAGACAGAGCT  540

Query  541   GAATGTTCCCTCTGGCTTTAGTAAGGAAAGAGCAGAGATGAAATCCGTGGACTGTGTGCA  600
             ||||||||||||||||||||||||||||||||||||||||||||||||||||||||||||
Sbjct  541   GAATGTTCCCTCTGGCTTTAGTAAGGAAAGAGCAGAGATGAAATCCGTGGACTGTGTGCA  600

Query  601   CGTCATCCAACAGAAGGGTATCTCCTCCTCTCCTCCTGCTCTCCCTGGTGCTGGTTTGGG  660
             ||||||||||||||||||||||||||||||||||||||||||||||||||||||||||||
Sbjct  601   CGTCATCCAACAGAAGGGTATCTCCTCCTCTCCTCCTGCTCTCCCTGGTGCTGGTTTGGG  660

Query  661   CAGGAAAGGACTCCTCTCGGTCACATTCCTGTGGCTTGCAATGCTCCTGTCCTCTTCTCT  720
             ||||||||||||||||||||||||||||||||||||||||||||||||||||||||||||
Sbjct  661   CAGGAAAGGACTCCTCTCGGTCACATTCCTGTGGCTTGCAATGCTCCTGTCCTCTTCTCT  720

Query  721   TGCAGCAGTGTGTCTTTACCACGTTCTCACACTTAAAGCAGAACTAGCAACTCTCCGCAG  780
             ||||||||||||||||||||||||||||||||||||||||||||||||||||||||||||
Sbjct  721   TGCAGCAGTGTGTCTTTACCACGTTCTCACACTTAAAGCAGAACTAGCAACTCTCCGCAG  780

Query  781   CGAGCTGATCTACAGAGTCCAGGCAAGAGCTCCGCTACCCCAGTCCCAGGATAAGAGTAA  840
             ||||||||||||||||||||||||||||||||||||||||||||||||||||||||||||
Sbjct  781   CGAGCTGATCTACAGAGTCCAGGCAAGAGCTCCGCTACCCCAGTCCCAGGATAAGAGTAA  840

Query  841   AGAGGGTACTGTATCCTCTTCTTCCTTGCAGTTATCAGCAGCTGCTGCCAGGCAGGAAAT  900
             ||||||||||||||||||||||||||||||||||||||||||||||||||||||||||||
Sbjct  841   AGAGGGTACTGTATCCTCTTCTTCCTTGCAGTTATCAGCAGCTGCTGCCAGGCAGGAAAT  900

Query  901   CAGGCTGCCTGATTCTGAGGCAGGTGAAGGTGATGAAGAGAGAGGCTGGAATGCAGGCAG  960
             ||||||||||||||||||||||||||||||||||||||||||||||||||||||||||||
Sbjct  901   CAGGCTGCCTGATTCTGAGGCAGGTGAAGGTGATGAAGAGAGAGGCTGGAATGCAGGCAG  960

Query  961   AAGCAGAGGGAGAAGGTCTGCTCTGCCCACAGAAGAAATAATGCTACAGGCCTGTCTGCA  1020
             ||||||||||||||||||||||||||||||||||||||||||||||||||||||||||||
Sbjct  961   AAGCAGAGGGAGAAGGTCTGCTCTGCCCACAGAAGAAATAATGCTACAGGCCTGTCTGCA  1020

Query  1021  ATTGATTGCTGATAGCAAAAGTAACATCCAACAAAAAGATGACTCAAGCATTGTTCCATG  1080
             ||||||||||||||||||||||||||||||||||||||||||||||||||||||||||||
Sbjct  1021  ATTGATTGCTGATAGCAAAAGTAACATCCAACAAAAAGATGACTCAAGCATTGTTCCATG  1080

Query  1081  GCTTCTTAGCTTTAAAAGAGGAACAGCTCTGGAAGAGCAAGGAAATAAAATATTGGTCAA  1140
             ||||||||||||||||||||||||||||||||||||||||||||||||||||||||||||
Sbjct  1081  GCTTCTTAGCTTTAAAAGAGGAACAGCTCTGGAAGAGCAAGGAAATAAAATATTGGTCAA  1140

Query  1141  AGAAGCTGGTTACTTTTTCATATATGGTCAGGTTTTATATACAGATACAACATTTGCTAT  1200
             ||||||||||||||||||||||||||||||||||||||||||||||||||||||||||||
Sbjct  1141  AGAAGCTGGTTACTTTTTCATATATGGTCAGGTTTTATATACAGATACAACATTTGCTAT  1200

Query  1201  GGGACATCTAATACAAAGGAAAAAGGCTCATGTATTTGGTGATGATCTCAGTTTGGTGAC  1260
             ||||||||||||||||||||||||||||||||||||||||||||||||||||||||||||
Sbjct  1201  GGGACATCTAATACAAAGGAAAAAGGCTCATGTATTTGGTGATGATCTCAGTTTGGTGAC  1260

Query  1261  ATTATTTCGTTGTATCCAAAACATGCCTCATTCTTATCCTAATAATTCTTGCTATACCGC  1320
             ||||||||||||||||||||||||||||||||||||||||||||||||||||||||||||
Sbjct  1261  ATTATTTCGTTGTATCCAAAACATGCCTCATTCTTATCCTAATAATTCTTGCTATACCGC  1320

Query  1321  TGGCATTGCAAAATTAGAAGAAGGGGATGAACTTCAACTTACAATACCACGGAGAAGGGC  1380
             ||||||||||||||||||||||||||||||||||||||||||||||||||||||||||||
Sbjct  1321  TGGCATTGCAAAATTAGAAGAAGGGGATGAACTTCAACTTACAATACCACGGAGAAGGGC  1380

Query  1381  CAAAATATCATTGGATGGAGATGGCACtttttttGGTGCAGTTCGACTCCTGTGATACTC  1440
             ||||||||||||||||||||||||||||||||||||||||||||||||||||||||||||
Sbjct  1381  CAAAATATCATTGGATGGAGATGGCACTTTTTTTGGTGCAGTTCGACTCCTGTGATACTC  1440

Query  1441  TACAGTTTTGCATCATGCTTATCTCTGTTCTCtttttttgtttttATGTAATTGaaaaaa  1500
             ||||||||||||||||||||||||||||||||||||||||||||||||||||||||||||
Sbjct  1441  TACAGTTTTGCATCATGCTTATCTCTGTTCTCTTTTTTTGTTTTTATGTAATTGAAAAAA  1500

Query  1501  aaTGGAGATACAGTTAAGCGGCCAATTCAATCtttttttttttt  1544
             ||||||||||||||||||||||||||||||||||||||||||||
Sbjct  1501  AATGGAGATACAGTTAAGCGGCCAATTCAATCTTTTTTTTTTTT  1544
```
