# Supplementary material for: Divergent IL18-STAT1 Immune Responses Underlie Differential Susceptibility to Aeromonas hydrophila in Geoclemys hamiltonii and Trachemys scripta: A Comparative Transcriptomic Perspective
Source: Genes (Basel). 2026 Apr 9;17(4):436. doi: 10.3390/genes17040436 (PMC13116093; doi:10.3390/genes17040436)
Supplement: Supplementary file 1 [file genes-17-00436-s001.zip › Code S1.pdf]

```
setwd("E:\\HC\TH1 vs CKH")
install.packages("readxl")
install.packages("tidyverse")
install.packages("dplyr")
install.packages("openxlsx")
library(readxl)
library(tidyverse)
library(dplyr)
library(openxlsx)

df<- read_excel("TH1 Vs CKH.xlsx")

num_cols <- str_count(df$Genes, ";") + 1
new_col_names <- paste0("Gene", 1:num_cols)
df_separated <- separate(df, Genes, into = new_col_names, sep = ";", fill = "right", extra =
"drop")


data1<-read_excel("Immune disease DOWN.xlsx")
data2<-read_excel("Unigene.xlsx")
result <- inner_join(data1, data2, by = "#Gene id")
write.xlsx(result, file = "Immune disease DOWN merged.xlsx")

data1<-read_excel("Immune system DOWN.xlsx")
data2<-read_excel("Unigene.xlsx")
result <- inner_join(data1, data2, by = "#Gene id")
write.xlsx(result, file = "Immune system DOWN merged.xlsx")

data1<-read_excel("Infectious disease bacterial DOWN.xlsx")
data2<-read_excel("Unigene.xlsx")
result <- inner_join(data1, data2, by = "#Gene id")
write.xlsx(result, file = "Infectious disease bacterial DOWN merged.xlsx")

data1<-read_excel("Infectious disease parasitic DOWN.xlsx")
data2<-read_excel("Unigene.xlsx")
result <- inner_join(data1, data2, by = "#Gene id")
write.xlsx(result, file = "Infectious disease parasitic DOWN merged.xlsx")

data1<-read_excel("Infectious disease viral DOWN.xlsx")
data2<-read_excel("Unigene.xlsx")
result <- inner_join(data1, data2, by = "#Gene id")
write.xlsx(result, file = "Infectious disease viral DOWN merged.xlsx")

data1<-read_excel("Immune disease UP.xlsx")
```

```
data2<-read_excel("Unigene.xlsx")
result <- inner_join(data1, data2, by = "#Gene id")
write.xlsx(result, file = "Immune disease UP merged.xlsx")

data1<-read_excel("Immune system UP.xlsx")
data2<-read_excel("Unigene.xlsx")
result <- inner_join(data1, data2, by = "#Gene id")
write.xlsx(result, file = "Immune system UP merged.xlsx")

data1<-read_excel("Infectious disease bacterial UP.xlsx")
data2<-read_excel("Unigene.xlsx")
result <- inner_join(data1, data2, by =

library(pheatmap)

set.seed(123)
data_matrix <- matrix(rnorm(100), nrow=10,
                      dimnames = list(paste0("Gene",1:10),
                                      paste0("Sample",LETTERS[1:10])))

heatmap_obj <- pheatmap(data_matrix,
                        cluster_cols = FALSE,
                        main = "High-Resolution Heatmap")

png(filename = "highres_heatmap.png",
    width = 3300,
    height = 2550,
    res = 300,
    type = "cairo")

heatmap_obj <- pheatmap(data_matrix,
                        cluster_cols = FALSE,
                        main = "High-Res Heatmap (300dpi)")
dev.off()

pdf(file = "vector_heatmap.pdf",
    width = 11,
    height = 8.5,
    useDingbats = FALSE)

pheatmap(data_matrix,
         cluster_cols = FALSE,
         main = "Vector Heatmap")
dev.off()
```

```
cat("Generated and saved files:\n",
    paste0("PNG file size: ", file.size("highres_heatmap.png")/1024, " KB\n"),
    paste0("PDF file size: ", file.size("vector_heatmap.pdf")/1024, " KB\n"))
```
