# Supplementary figures and images for: Divergent IL18-STAT1 Immune Responses Underlie Differential Susceptibility to Aeromonas hydrophila in Geoclemys hamiltonii and Trachemys scripta: A Comparative Transcriptomic Perspective
Source: Genes (Basel). 2026 Apr 9;17(4):436. doi: 10.3390/genes17040436 (PMC13116093; doi:10.3390/genes17040436)

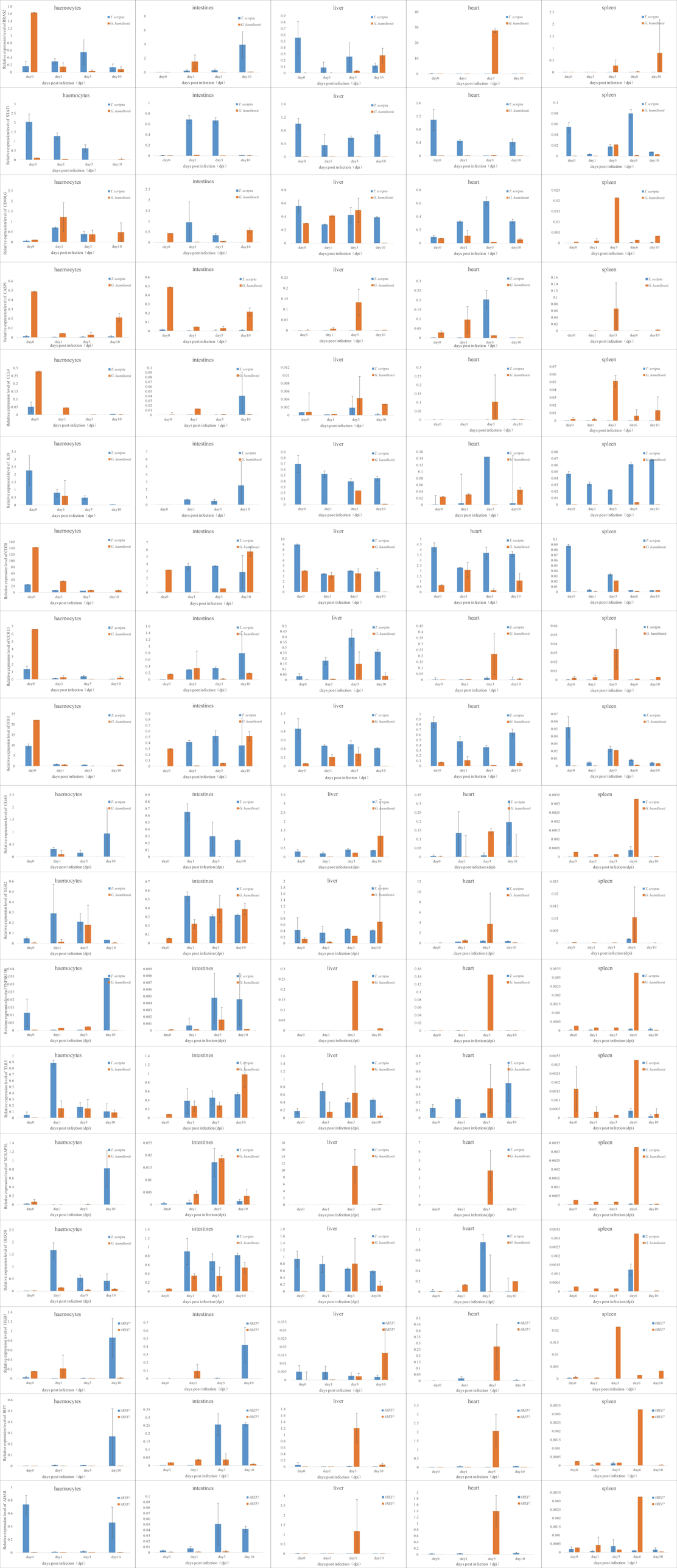

Supplement: Supplementary file 1 [file genes-17-00436-s001.zip › Figure S1.pdf]
