# Supplementary material for: Divergent IL18-STAT1 Immune Responses Underlie Differential Susceptibility to Aeromonas hydrophila in Geoclemys hamiltonii and Trachemys scripta: A Comparative Transcriptomic Perspective
Source: Genes (Basel). 2026 Apr 9;17(4):436. doi: 10.3390/genes17040436 (PMC13116093; doi:10.3390/genes17040436)
Supplement: Supplementary file 1 [file IFIH1.pdf › Figure S2/IFIH1.pdf]

PREDICTED: Trachemys scripta elegans interferon induced with helicase C domain 1 (IFIH1), transcript variant X2, mRNA

Sequence ID: XM_034786312.1 Length: 4020 Number of Matches: 2

Related Information
Gene - associated gene
Genome Data Viewer - a
genomic context

Range 1: 77 to 4020 GenBank Graphics

▼ Next Match ▲ Previous Match

Score	Expect	Identities	Gaps	Strand
7284 bits(3944)	0.0	3944/3944(100%)	0/3944(0%)	Plus/Plus

```
Query  863   GATCTTAGCTGCAACTGAAAATCGTGGAGATAGGGAAGGTGCACGGGAGTTGTTAAGTAG  922
             ||||||||||||||||||||||||||||||||||||||||||||||||||||||||||||
Sbjct  77    GATCTTAGCTGCAACTGAAAATCGTGGAGATAGGGAAGGTGCACGGGAGTTGTTAAGTAG  136

Query  923   AATAATAAAGAAGAAAGATTGGTTCTCTCAATTTTTGGCTGTCCTGCGTGAAACTAAGCA  982
             ||||||||||||||||||||||||||||||||||||||||||||||||||||||||||||
Sbjct  137   AATAATAAAGAAGAAAGATTGGTTCTCTCAATTTTTGGCTGTCCTGCGTGAAACTAAGCA  196

Query  983   TGGAGACCTTGCAGATGAACTAAGTGGAAATGTACCAGAAACAGAAAACAAAGAAAATGG  1042
             ||||||||||||||||||||||||||||||||||||||||||||||||||||||||||||
Sbjct  197   TGGAGACCTTGCAGATGAACTAAGTGGAAATGTACCAGAAACAGAAAACAAAGAAAATGG  256

Query  1043  GATCAAGCAGCCAGCTACTACAGATGAGGCCATGGAAGCTGAAGAGCCATTGGCCTCTGA  1102
             ||||||||||||||||||||||||||||||||||||||||||||||||||||||||||||
Sbjct  257   GATCAAGCAGCCAGCTACTACAGATGAGGCCATGGAAGCTGAAGAGCCATTGGCCTCTGA  316

Query  1103  AGTGGGAGAGGAGCTGAAGCAGATTGATGAAATGAATGATAGTCTGGCCAGTGAGAGCAT  1162
             ||||||||||||||||||||||||||||||||||||||||||||||||||||||||||||
Sbjct  317   AGTGGGAGAGGAGCTGAAGCAGATTGATGAAATGAATGATAGTCTGGCCAGTGAGAGCAT  376

Query  1163  TATATTAGGAACATCTTTTGAAGACGCTTCTGTAGTTTCAGAGTCTGATGTCAGTTTGCC  1222
             ||||||||||||||||||||||||||||||||||||||||||||||||||||||||||||
Sbjct  377   TATATTAGGAACATCTTTTGAAGACGCTTCTGTAGTTTCAGAGTCTGATGTCAGTTTGCC  436

Query  1223  AGATGCAAGTGTCAGTTGCTTAAATGAAAGCATGGGTCACAGTAGCGCCACAAGTGGTGA  1282
             ||||||||||||||||||||||||||||||||||||||||||||||||||||||||||||
Sbjct  437   AGATGCAAGTGTCAGTTGCTTAAATGAAAGCATGGGTCACAGTAGCGCCACAAGTGGTGA  496

Query  1283  TTCAGATGAGGACAATATGGACAAAAGAGCTTCCCCTGAACCAGAGCTGATTCTCAGAGA  1342
             ||||||||||||||||||||||||||||||||||||||||||||||||||||||||||||
Sbjct  497   TTCAGATGAGGACAATATGGACAAAAGAGCTTCCCCTGAACCAGAGCTGATTCTCAGAGA  556

Query  1343  TTACCAGATGGAGGTTGCAAAACCAGCACTGAATGGGGAGAACGTTATAATATGTCTCCC  1402
             ||||||||||||||||||||||||||||||||||||||||||||||||||||||||||||
Sbjct  557   TTACCAGATGGAGGTTGCAAAACCAGCACTGAATGGGGAGAACGTTATAATATGTCTCCC  616

Query  1403  TACAGGCAGTGGTAAAACCAGAGTGGCTGTTTACATTACCAAAGAGCATTTGGATAAGAA  1462
             ||||||||||||||||||||||||||||||||||||||||||||||||||||||||||||
Sbjct  617   TACAGGCAGTGGTAAAACCAGAGTGGCTGTTTACATTACCAAAGAGCATTTGGATAAGAA  676

Query  1463  GAAAAGAGCATCAGAGCCTGGAAAAGTTATAGTACTTGTGAATAAGGTACCACTAGTGGA  1522
             ||||||||||||||||||||||||||||||||||||||||||||||||||||||||||||
Sbjct  677   GAAAAGAGCATCAGAGCCTGGAAAAGTTATAGTACTTGTGAATAAGGTACCACTAGTGGA  736

Query  1523  GCAGCATTTCAGAAAAGAGTTCAACCCATTCCTGAAGCATTGGTATCGGGTTACTGGATT  1582
             ||||||||||||||||||||||||||||||||||||||||||||||||||||||||||||
Sbjct  737   GCAGCATTTCAGAAAAGAGTTCAACCCATTCCTGAAGCATTGGTATCGGGTTACTGGATT  796

Query  1583  AAGTGGTGATTCTCAGCTGAAAATCTCATTTCCTGAAGTTGTAAGAAAGAATGATGTCAT  1642
             ||||||||||||||||||||||||||||||||||||||||||||||||||||||||||||
Sbjct  797   AAGTGGTGATTCTCAGCTGAAAATCTCATTTCCTGAAGTTGTAAGAAAGAATGATGTCAT  856

Query  1643  TATCAGTACTGCCCAGATCCTTGAGAATTCACTGCTGAATGCAACCACAGAAGATGATGA  1702
             ||||||||||||||||||||||||||||||||||||||||||||||||||||||||||||
Sbjct  857   TATCAGTACTGCCCAGATCCTTGAGAATTCACTGCTGAATGCAACCACAGAAGATGATGA  916

Query  1703  AGAAGGTGTCCAGTTATCAGACTTTTCTCTCATTGTTATTGATGAATGTCATCACACCCA  1762
             ||||||||||||||||||||||||||||||||||||||||||||||||||||||||||||
Sbjct  917   AGAAGGTGTCCAGTTATCAGACTTTTCTCTCATTGTTATTGATGAATGTCATCACACCCA  976

Query  1763  GAAGGAAGGCGTCTATAACAATATAATGCGACGTTATTTaaaagaaaagatgaaaaacaa  1822
             ||||||||||||||||||||||||||||||||||||||||||||||||||||||||||||
Sbjct  977   GAAGGAAGGCGTCTATAACAATATAATGCGACGTTATTTAAAAGAAAAGATGAAAAACAA  1036

Query  1823  tagattcagaaaagaaaacaaaCCCCTGATTCCACAGCCTCAGATCCTGGGACTAACTGC  1882
             ||||||||||||||||||||||||||||||||||||||||||||||||||||||||||||
Sbjct  1037  TAGATTCAGAAAAGAAAACAAACCCCTGATTCCACAGCCTCAGATCCTGGGACTAACTGC  1096

Query  1883  TTCACCTGGCGTAGGAGGGGCAAGAACTCATCAGAAAGCGGAAGAACATATTCTGAAAAT  1942
             ||||||||||||||||||||||||||||||||||||||||||||||||||||||||||||
Sbjct  1097  TTCACCTGGCGTAGGAGGGGCAAGAACTCATCAGAAAGCGGAAGAACATATTCTGAAAAT  1156

Query  1943  TTGTGCTAATCTTGATGCATATAGACTCATGACTGTTGAAGAACATGTCTCCCAGCTGAA  2002
             ||||||||||||||||||||||||||||||||||||||||||||||||||||||||||||
Sbjct  1157  TTGTGCTAATCTTGATGCATATAGACTCATGACTGTTGAAGAACATGTCTCCCAGCTGAA  1216

Query  2003  AGATCAGGTGAAGGAACCATATAAGAAGTTTGTGATTGCAGATGACAAAAGAAGGGATCC  2062
             ||||||||||||||||||||||||||||||||||||||||||||||||||||||||||||
Sbjct  1217  AGATCAGGTGAAGGAACCATATAAGAAGTTTGTGATTGCAGATGACAAAAGAAGGGATCC  1276

Query  2063  CTTTAGAGAGAAAATTACAGAAATTATGACAGAAATTCAAAATTATTGTCACCTCAACCC  2122
             ||||||||||||||||||||||||||||||||||||||||||||||||||||||||||||
Sbjct  1277  CTTTAGAGAGAAAATTACAGAAATTATGACAGAAATTCAAAATTATTGTCACCTCAACCC  1336

Query  2123  AACATTTGAATTTGGAACTCAGCCCTACGAACAGTGGGCGGTTAAAGAGGAGAAAAAAGC  2182
             ||||||||||||||||||||||||||||||||||||||||||||||||||||||||||||
Sbjct  1337  AACATTTGAATTTGGAACTCAGCCCTACGAACAGTGGGCGGTTAAAGAGGAGAAAAAAGC  1396

Query  2183  TGCAAAAGAAGAAAACCGTAAAGAACGTGTTTGTGCGGAGCATTTAAAGAAGTATAATGA  2242
             ||||||||||||||||||||||||||||||||||||||||||||||||||||||||||||
Sbjct  1397  TGCAAAAGAAGAAAACCGTAAAGAACGTGTTTGTGCGGAGCATTTAAAGAAGTATAATGA  1456

Query  2243  TGCCCTGCAAATTAATGACACTATCCGAATGATCGATGCATACAACCACCTCAATAACTT  2302
             ||||||||||||||||||||||||||||||||||||||||||||||||||||||||||||
Sbjct  1457  TGCCCTGCAAATTAATGACACTATCCGAATGATCGATGCATACAACCACCTCAATAACTT  1516

Query  2303  CTATAAGGAGGAGAAAAGCAAGAAAACAACAGTAAATGAAGACGACGATGAACCAGTAGC  2362
             ||||||||||||||||||||||||||||||||||||||||||||||||||||||||||||
Sbjct  1517  CTATAAGGAGGAGAAAAGCAAGAAAACAACAGTAAATGAAGACGACGATGAACCAGTAGC  1576

Query  2363  ATCAAAACTAGATGAAACAGATACGTTTCTGATAAAGTTATTTTACGCAAAAAAGAAACA  2422
             ||||||||||||||||||||||||||||||||||||||||||||||||||||||||||||
Sbjct  1577  ATCAAAACTAGATGAAACAGATACGTTTCTGATAAAGTTATTTTACGCAAAAAAGAAACA  1636

Query  2423  GCTGAAAGAGTTGGCTGGAAAGCCAGAATATGAAAATGAGAAGCTGACACAGTTGCGAAA  2482
             ||||||||||||||||||||||||||||||||||||||||||||||||||||||||||||
Sbjct  1637  GCTGAAAGAGTTGGCTGGAAAGCCAGAATATGAAAATGAGAAGCTGACACAGTTGCGAAA  1696

Query  2483  CACCTTAATGGAGGAGTTCACAAAGACTGATGAAGCTAGGGGAATTATCTTCACAAAGAC  2542
             ||||||||||||||||||||||||||||||||||||||||||||||||||||||||||||
Sbjct  1697  CACCTTAATGGAGGAGTTCACAAAGACTGATGAAGCTAGGGGAATTATCTTCACAAAGAC  1756

Query  2543  TCGGCAAAGTGCATTTGCCCTTTTTCAGTGGATTAAGGACAACACAAAATTTGAAGAAGT  2602
             ||||||||||||||||||||||||||||||||||||||||||||||||||||||||||||
Sbjct  1757  TCGGCAAAGTGCATTTGCCCTTTTTCAGTGGATTAAGGACAACACAAAATTTGAAGAAGT  1816

Query  2603  GGGAATTAAAGCTCACTACCTTATTGGAGCTGGGCATAACAGTGAATTTAAACCCATGAC  2662
             ||||||||||||||||||||||||||||||||||||||||||||||||||||||||||||
Sbjct  1817  GGGAATTAAAGCTCACTACCTTATTGGAGCTGGGCATAACAGTGAATTTAAACCCATGAC  1876

Query  2663  TCAGAATGAACAAAGAGAAATAATCAGTAAATTTCGTGCTGGAAACGTCAACCTACTTAT  2722
             ||||||||||||||||||||||||||||||||||||||||||||||||||||||||||||
Sbjct  1877  TCAGAATGAACAAAGAGAAATAATCAGTAAATTTCGTGCTGGAAACGTCAACCTACTTAT  1936

Query  2723  TGCTACTACTGTGGCTGAAGAAGGCCTGGATATCAAAGAGTGTAACATAGTTATCCGCTA  2782
             ||||||||||||||||||||||||||||||||||||||||||||||||||||||||||||
Sbjct  1937  TGCTACTACTGTGGCTGAAGAAGGCCTGGATATCAAAGAGTGTAACATAGTTATCCGCTA  1996

Query  2783  TGGTCTTGTTACCAATGAAATAGCTATGGTGCAGGCCCGGGGTCGAGCTCGAGCTGATGA  2842
             ||||||||||||||||||||||||||||||||||||||||||||||||||||||||||||
Sbjct  1997  TGGTCTTGTTACCAATGAAATAGCTATGGTGCAGGCCCGGGGTCGAGCTCGAGCTGATGA  2056

Query  2843  GAGCACATATGTACTGGTGGCATCTGGTGGATCGGGAGCTGTTGAACGTGAAAATGTTAA  2902
             ||||||||||||||||||||||||||||||||||||||||||||||||||||||||||||
Sbjct  2057  GAGCACATATGTACTGGTGGCATCTGGTGGATCGGGAGCTGTTGAACGTGAAAATGTTAA  2116

Query  2903  TATATTTCGTGAGCAAATGATGTATAAAGCTATTCAACGTGTCCAAAGGATGCGACGGGA  2962
             ||||||||||||||||||||||||||||||||||||||||||||||||||||||||||||
Sbjct  2117  TATATTTCGTGAGCAAATGATGTATAAAGCTATTCAACGTGTCCAAAGGATGCGACGGGA  2176

Query  2963  GGAATATTTAAATAAGATTCAGGGCTTTCAGATGCAAAGTATAATGGaaaaaaaaaTGAA  3022
             ||||||||||||||||||||||||||||||||||||||||||||||||||||||||||||
Sbjct  2177  GGAATATTTAAATAAGATTCAGGGCTTTCAGATGCAAAGTATAATGGAAAAAAAAATGAA  2236

Query  3023  GGCAAAGAGAGATCAGCTTAAGACATACAAGGAAAATCCATCACTAATAACTTTCCTTTG  3082
             ||||||||||||||||||||||||||||||||||||||||||||||||||||||||||||
Sbjct  2237  GGCAAAGAGAGATCAGCTTAAGACATACAAGGAAAATCCATCACTAATAACTTTCCTTTG  2296

Query  3083  CAAAAATTGCCACAAACTGAAATGTTCTGGGGAAGACATACAAGTGATAGAAAATATGCA  3142
             ||||||||||||||||||||||||||||||||||||||||||||||||||||||||||||
Sbjct  2297  CAAAAATTGCCACAAACTGAAATGTTCTGGGGAAGACATACAAGTGATAGAAAATATGCA  2356

Query  3143  TCATGTCAATGTGAAAAAGGAGTTCCAGGACCTTTACAGCATAAGAGAAAATAAAACACT  3202
             ||||||||||||||||||||||||||||||||||||||||||||||||||||||||||||
Sbjct  2357  TCATGTCAATGTGAAAAAGGAGTTCCAGGACCTTTACAGCATAAGAGAAAATAAAACACT  2416

Query  3203  GCAAGAAAAACATGCCGATTACCAAATAAATGGTGAAATTATCTGCAAAGATTGTGGGCA  3262
             ||||||||||||||||||||||||||||||||||||||||||||||||||||||||||||
Sbjct  2417  GCAAGAAAAACATGCCGATTACCAAATAAATGGTGAAATTATCTGCAAAGATTGTGGGCA  2476

Query  3263  AGCTTGGGGAAATATGATGGTGCACCGAGGTGTAGACCTGCCTTGCTTAAAGATTAGAAA  3322
             ||||||||||||||||||||||||||||||||||||||||||||||||||||||||||||
Sbjct  2477  AGCTTGGGGAAATATGATGGTGCACCGAGGTGTAGACCTGCCTTGCTTAAAGATTAGAAA  2536

Query  3323  TTTTGTAGTTGTATTCAAGGATAAGAAAACCTCAAACAAAATTTTCAAAAAATGGGGAGA  3382
             ||||||||||||||||||||||||||||||||||||||||||||||||||||||||||||
Sbjct  2537  TTTTGTAGTTGTATTCAAGGATAAGAAAACCTCAAACAAAATTTTCAAAAAATGGGGAGA  2596

Query  3383  GCTACCAATCAAGTTCCCTGTCTTTGACTATGCAGAGCATTATGTTTCCAGTGACGAAGA  3442
             ||||||||||||||||||||||||||||||||||||||||||||||||||||||||||||
Sbjct  2597  GCTACCAATCAAGTTCCCTGTCTTTGACTATGCAGAGCATTATGTTTCCAGTGACGAAGA  2656

Query  3443  TTAAAAATTCATTCAAGGATGTAAAGAAATGCAACTATTCAGCATTATTTTGACTGCTAT  3502
             ||||||||||||||||||||||||||||||||||||||||||||||||||||||||||||
Sbjct  2657  TTAAAAATTCATTCAAGGATGTAAAGAAATGCAACTATTCAGCATTATTTTGACTGCTAT  2716

Query  3503  AATCAGACTCCTCTTTTGCATACAGAAGGCCAAATTTTGCATCCTACAGACTGTGGAAGG  3562
             ||||||||||||||||||||||||||||||||||||||||||||||||||||||||||||
Sbjct  2717  AATCAGACTCCTCTTTTGCATACAGAAGGCCAAATTTTGCATCCTACAGACTGTGGAAGG  2776

Query  3563  CCTTGAATGCAGTAAAACTATTGTGTGTGAAGAGGAAGGACATGGAGAGGAGAGAGGACT  3622
             ||||||||||||||||||||||||||||||||||||||||||||||||||||||||||||
Sbjct  2777  CCTTGAATGCAGTAAAACTATTGTGTGTGAAGAGGAAGGACATGGAGAGGAGAGAGGACT  2836

Query  3623  GTGCTGTCTGGCAGCTGTTTACACTCCAGCATGCAGTGGAGGTTTGGGATGGGTTGAGGA  3682
             ||||||||||||||||||||||||||||||||||||||||||||||||||||||||||||
Sbjct  2837  GTGCTGTCTGGCAGCTGTTTACACTCCAGCATGCAGTGGAGGTTTGGGATGGGTTGAGGA  2896

Query  3683  AAAAGAAGACCTCATGGAGCCAGGCTTAGTGTGGGTCCACAGAACAAGCCTACTCTACCT  3742
             ||||||||||||||||||||||||||||||||||||||||||||||||||||||||||||
Sbjct  2897  AAAAGAAGACCTCATGGAGCCAGGCTTAGTGTGGGTCCACAGAACAAGCCTACTCTACCT  2956

Query  3743  ACACAGCAGAATCCAGTCAATTATCAAGGTCTCCCTTATAtttttttAATTGCAGCTGTC  3802
             ||||||||||||||||||||||||||||||||||||||||||||||||||||||||||||
Sbjct  2957  ACACAGCAGAATCCAGTCAATTATCAAGGTCTCCCTTATATTTTTTTAATTGCAGCTGTC  3016

Query  3803  ATTTTTATTATTGCAATGAGTGCCTTTCGCTGGCTGAGACTTTCTTCAAATATTTTGGGG  3862
             ||||||||||||||||||||||||||||||||||||||||||||||||||||||||||||
Sbjct  3017  ATTTTTATTATTGCAATGAGTGCCTTTCGCTGGCTGAGACTTTCTTCAAATATTTTGGGG  3076

Query  3863  GTTTTATCTCAAGAGACGCTTTTTAAAAAATAACTTGATTGTAGTTCAAATGTGCTGAAA  3922
             ||||||||||||||||||||||||||||||||||||||||||||||||||||||||||||
Sbjct  3077  GTTTTATCTCAAGAGACGCTTTTTAAAAAATAACTTGATTGTAGTTCAAATGTGCTGAAA  3136

Query  3923  GCAGGGATTGGGAATCAGTTTTTGGTGACACAGCAGCAGAAGTGGAGTACAGGGTATGCA  3982
             ||||||||||||||||||||||||||||||||||||||||||||||||||||||||||||
Sbjct  3137  GCAGGGATTGGGAATCAGTTTTTGGTGACACAGCAGCAGAAGTGGAGTACAGGGTATGCA  3196

Query  3983  AGCATTATCCCAGTGCAAGAGTTGGTATGGGCCACTGACTCCTAGAAGTGGTCTAAGCTG  4042
             ||||||||||||||||||||||||||||||||||||||||||||||||||||||||||||
Sbjct  3197  AGCATTATCCCAGTGCAAGAGTTGGTATGGGCCACTGACTCCTAGAAGTGGTCTAAGCTG  3256

Query  4043  GTCATTTTCCTCTCCTCCTTCCTCATGCTCACATTGCTACTACTCAGAAGCACAGGTGAC  4102
             ||||||||||||||||||||||||||||||||||||||||||||||||||||||||||||
Sbjct  3257  GTCATTTTCCTCTCCTCCTTCCTCATGCTCACATTGCTACTACTCAGAAGCACAGGTGAC  3316

Query  4103  CTTGCTGTCTCTACTTCTACACTATGTCCTGAAAGGGGAGGCCGGTGAGGGGAGTAGATT  4162
             ||||||||||||||||||||||||||||||||||||||||||||||||||||||||||||
Sbjct  3317  CTTGCTGTCTCTACTTCTACACTATGTCCTGAAAGGGGAGGCCGGTGAGGGGAGTAGATT  3376

Query  4163  TACCTAGTGTTGTCTCCCAGAATGCTCTGCTTCTCTCCCTTTATGCCATCAGCTGGCGAC  4222
             ||||||||||||||||||||||||||||||||||||||||||||||||||||||||||||
Sbjct  3377  TACCTAGTGTTGTCTCCCAGAATGCTCTGCTTCTCTCCCTTTATGCCATCAGCTGGCGAC  3436

Query  4223  TCCCAAATGTGTTATGTGGTCAGACTAAAACTAAACTACTATTAAAACATGGTTTGAAAA  4282
             ||||||||||||||||||||||||||||||||||||||||||||||||||||||||||||
Sbjct  3437  TCCCAAATGTGTTATGTGGTCAGACTAAAACTAAACTACTATTAAAACATGGTTTGAAAA  3496

Query  4283  ATCACTGTATTTAAACCCATTTAAATATCCAGCTTCTCAAATTCCAGAACTTCGGTGACA  4342
             ||||||||||||||||||||||||||||||||||||||||||||||||||||||||||||
Sbjct  3497  ATCACTGTATTTAAACCCATTTAAATATCCAGCTTCTCAAATTCCAGAACTTCGGTGACA  3556

Query  4343  AATATAACTTTACCTGTGCAGTAACACGAAAAGACTGTGTAAAATGTTGGAGATAAAATA  4402
             ||||||||||||||||||||||||||||||||||||||||||||||||||||||||||||
Sbjct  3557  AATATAACTTTACCTGTGCAGTAACACGAAAAGACTGTGTAAAATGTTGGAGATAAAATA  3616

Query  4403  ATGTAGCCCATTAATAAATAATCTCTGTAAACTGTGATACAGCTTACTTCCTGTCGCCAT  4462
             ||||||||||||||||||||||||||||||||||||||||||||||||||||||||||||
Sbjct  3617  ATGTAGCCCATTAATAAATAATCTCTGTAAACTGTGATACAGCTTACTTCCTGTCGCCAT  3676

Query  4463  TAGTGCATAGACATTCAGATACGGTAATTGTTGAAGCTTCTCTTCATGACATAttttttt  4522
             ||||||||||||||||||||||||||||||||||||||||||||||||||||||||||||
Sbjct  3677  TAGTGCATAGACATTCAGATACGGTAATTGTTGAAGCTTCTCTTCATGACATATTTTTTT  3736

Query  4523  AAACACGTGGTGTAAAATCCTGGCCCTATTTGAAGTTGATGGCAAAACTCCTATTGACTT  4582
             ||||||||||||||||||||||||||||||||||||||||||||||||||||||||||||
Sbjct  3737  AAACACGTGGTGTAAAATCCTGGCCCTATTTGAAGTTGATGGCAAAACTCCTATTGACTT  3796

Query  4583  CAATAGGGCCAGGATTCCACCCTTGCAATTTAAAGTGCAGCTCTGCGCTAAAAAGTGACT  4642
             ||||||||||||||||||||||||||||||||||||||||||||||||||||||||||||
Sbjct  3797  CAATAGGGCCAGGATTCCACCCTTGCAATTTAAAGTGCAGCTCTGCGCTAAAAAGTGACT  3856

Query  4643  GCAAAAATGGCACCATGTGGTTCCATATTTGTCTCATTGACCAATTACATATTTTCTCAC  4702
             ||||||||||||||||||||||||||||||||||||||||||||||||||||||||||||
Sbjct  3857  GCAAAAATGGCACCATGTGGTTCCATATTTGTCTCATTGACCAATTACATATTTTCTCAC  3916

Query  4703  CTTACAAGTAAGATAACTGTGGTTTTGTCTTGTTTTCCATAATGTCAGTGTTGTAAACTC  4762
             ||||||||||||||||||||||||||||||||||||||||||||||||||||||||||||
Sbjct  3917  CTTACAAGTAAGATAACTGTGGTTTTGTCTTGTTTTCCATAATGTCAGTGTTGTAAACTC  3976

Query  4763  AACCTGGGATCTCTGAAAGTAAAGCTGTGTTCTTTCTGGCTTGA  4806
             ||||||||||||||||||||||||||||||||||||||||||||
Sbjct  3977  AACCTGGGATCTCTGAAAGTAAAGCTGTGTTCTTTCTGGCTTGA  4020
```

Range 2: 1 to 77 GenBank Graphics

▼ Next Match ▲ Previous Match ▲ First Match

Score	Expect	Identities	Gaps	Strand
143 bits(77)	1e-36	77/77(100%)	0/77(0%)	Plus/Plus

```
Query  1   AGAGCAGGCAGGCCGCTGAGCCACTGCGCTAGTGTCAGACCCGGCAGAGGGAGAGGCTGC  60
           ||||||||||||||||||||||||||||||||||||||||||||||||||||||||||||
Sbjct  1   AGAGCAGGCAGGCCGCTGAGCCACTGCGCTAGTGTCAGACCCGGCAGAGGGAGAGGCTGC  60

Query  61  GCTGGGGGATTTGTAAG  77
           |||||||||||||||||
Sbjct  61  GCTGGGGGATTTGTAAG  77
```
